# Epidemiology and clinical features of SARS-CoV-2 infection in children and adolescents in the pre-Omicron era: A global systematic review and meta-analysis

**DOI:** 10.7189/jogh.14.05003

**Published:** 2024-03-01

**Authors:** Durga Kulkarni, Nabihah Farhana Ismail, Fuyu Zhu, Xin Wang, Graciela del Carmen Morales, Amit Srivastava, Kristen E Allen, Julia Spinardi, Ahmed Ehsanur Rahman, Moe H Kyaw, Harish Nair

**Affiliations:** 1 Centre for Global Health, University of Edinburgh, Edinburgh, United Kingdom; 2 Communicable Disease Control Unit, Public Health Department, Johor State, Malaysia; 3 Schol of Public Health, Nanjing Medical University, China; 4 Pfizer, Vaccines, Emerging Markets; 5 Orbital Therapeutics, USA; 6 International Centre for Diarrhoeal Diseases Research, Bangladesh; 7 MRC/Wits Rural Public Health and Health Transitions Research Unit (Agincourt), School of Public Health, Faculty of Health Sciences, University of the Witwatersrand, Johannesburg, South Africa; **Background** Data on paediatric severe acute respiratory syndrome coronavirus 2 (SARS-CoV-2) infections are limited, especially from the early pandemic period. Moreover, children are not considered to be at high risk for severe outcomes of coronavirus disease 2019 (COVID-19). In this systematic review and meta-analysis, we estimated the SAR-CoV-2 positivity rate, identified risk factors, and described the severity and mortality outcomes of SARS-CoV-2 infections in children aged ≤18 years until December 2021, prior to the Omicron era.

## Abstract

**Methods:**

We searched MEDLINE, Embase, Global Health, CINAHL, China National Knowledge Infrastructure, Wanfang, CQvip, and the World Health Organization (WHO) COVID-19 global literature databases for primary studies recruiting children aged ≤18 years with a diagnosis of SARS-CoV-2 infection confirmed either by molecular or antigen tests. We used the Joanna Briggs Institute critical appraisal tools to appraise the study quality and conducted meta-analyses using the random effects model for all outcomes except for race/ethnicity as risk factors of SARS-CoV-2 infection.

**Results:**

We included 237 studies, each reporting at least one of the study outcomes. Based on data from 117 studies, the pooled SARS-CoV-2 positivity rate was 9.30% (95% confidence interval (CI) = 7.15–11.73). Having a comorbidity was identified as a risk factor for SARS-CoV-2 infection (risk ratio (RR) = 1.33; 95% CI = 1.04–1.71) based on data from 49 studies. Most cases in this review presented with mild disease (n = 50; 52.47% (95% CI = 44.03–60.84)). However, 20.70% of paediatric SARS-CoV-2 infections were hospitalised (67 studies), 7.19% required oxygen support (57 studies), 4.26% required intensive care (93 studies), and 2.92% required assisted ventilation (63 studies). The case fatality ratio (n = 119) was 0.87% (95% CI = 0.54–1.28), which included in-hospital and out-of-hospital deaths.

**Conclusions:**

Our data showed that children were at risk for SARS-CoV-2 infections and severe outcomes in the pre-Omicron era. These findings underscore the need for effective vaccination strategies for the paediatric population to protect against the acute and long-term sequelae of COVID-19.

**Registration:**

PROSPERO: CRD42022327680.

The World Health Organization (WHO) declared the coronavirus disease 2019 (COVID-19) outbreak a global pandemic on 11 March 2020 [1]. WHO estimates suggest that there have been over 663 million confirmed COVID-19 cases, including at least 6 million reported deaths as of 19 January 2023 [2]. Since the emergence of the first case in Wuhan, China in late 2019, numerous SARS-CoV-2 variants have emerged and have been circulating globally. The Omicron variant, (also called variant B.1.1.529), was first reported to WHO on 24 November 2021 and has been circulating ever since [3]. There is a large volume of data on the epidemiology and economic impact of SARS-CoV-2 infections; unfortunately, comprehensive data on the burden of SARS-CoV-2 infections in children and adolescents are limited, particularly in the pre-Omicron era.

The United Nations (UN) defines children as persons under the age of 18 years [4] and adolescents as persons between the ages of 10 and 19 years [5]. Evidence suggests that individuals under 20 years of age represented 33% of the 2020 global population and contributed to 21% of all reported COVID-19 cases since the beginning of the pandemic until the end of 2022 [6]. Although these numbers indicate that the incidence of COVID-19 is low in this compared to other age groups, a lack of data from this population has been identified as a challenge for developing precise estimates [6]. Moreover, the case numbers represent just one aspect of the disease burden. A comprehensive determination of the burden of COVID-19 is incomplete without estimating the disease severity and highest level of management or care required by cases. Furthermore, it is important to identify vulnerable groups within this population to prioritise protection and prevention measures, access to health care, or even treatment.

Therefore, we aimed to estimate the burden of SARS-CoV-2 infections (in terms of test positivity, severity of clinical presentation, and the level of care required) in children and adolescents aged ≤18 years from published literature reporting data from the pre-Omicron era, and to report data on risk factors for SARS-CoV-2 infections and related mortality in this population. This systematic review of existing evidence and synthesis of knowledge will help improve our understanding of the burden of SARS-CoV-2 infections in children and adolescents globally and help shape and inform protection and prevention policies and management guidelines.

## METHODS

We registered the protocol for this systematic review in PROSPERO (CRD42022327680) and followed the PRISMA-P 2020 guidelines in reporting our findings [7] (Table S12–13 in the **Online Supplementary Document**). We did not seek formal ethical approval as this review was based on data from open-access published primary studies.

### Literature search

We searched MEDLINE, Embase, Global Health, CINAHL, and the WHO COVID-19 global literature databases on 27 February 2022 using pre-developed search strategies to identify studies reporting incidence, risk factors, severity, and outcomes of COVID-19 infection in children and adolescents aged ≤18 years. We also searched three Chinese literature databases (CNKI, Wanfang, and CQvip) on 21 March 2022 (Text S2 in the in the **Online Supplementary Document**).

### Literature selection

Following deduplication, we uploaded the references retrieved from the searches in English language databases into Covidence (Veritas Health Innovation, Melbourne, Australia). A single reviewer (DK) then screened their titles and abstracts. During the screening, we observed that many studies defined children as those aged ≤18 years rather than those aged <18 years. Therefore, the review population included children and adolescents, i.e. those aged ≤18 years, as data were very commonly reported for this age group. A single reviewer screened the full text of the studies based on pre-defined inclusion criteria, depending on the study language (English: DK, Chinese: FZ or XW, Portuguese: JS, Spanish: KA). We included observational studies with a sample size of at least 100 children; presenting data on people aged ≤18 years; reporting on SARS-CoV-2 infection confirmed by polymerase chain reaction (PCR) or antigen tests; and reporting test positivity, severity, risk factors, or mortality due to SARS-CoV-2 infection or the differential impacts of SARS-CoV-2 variants. We set no criteria regarding settings (community, educational institutions, health care settings, etc.) (Text S1 in the **Online Supplementary Document**).

### Data extraction

We designed a data extraction form in Microsoft Access to capture characteristics and outcomes for studies included in this systematic review and meta-analysis (see variables in Text S3 in the **Online Supplementary Document**). A single reviewer extracted the data into Microsoft Access (DK, JS, or KA) or Microsoft Excel (FZ), while a second reviewer cross-checked the extractions in MS Excel (English-language studies: NFI, Chinese-language studies: XW). Any disagreements between the two reviewers were resolved through discussion or by consulting a third reviewer (HN) if necessary.

### Quality assessment

A single reviewer (DK, KA, or FZ) assessed the quality of the included studies using the modified Joanna Briggs Institute quality assessment checklists, according to each study’s design [8] (Text S4 in the **Online Supplementary Document**). English language studies marked as good quality or poor quality by the first reviewer were re-assessed by the second reviewer (NFI). Studies were deemed to be of good quality if they had a score of 7–8 (cross-sectional studies), 8–10 (case-control studies and quasi-experimental studies), 9–11 (cohort studies), or 8–10 (diagnostic test accuracy studies); of fair quality if they had a score of 4–6 (cross-sectional studies), 4–7 (case-control studies and quasi-experimental studies), 6 and 8 (cohort studies), or 4–7 (diagnostic test accuracy studies); and of poor quality if they had a score of 0–3 (cross-sectional, case-control, quasi-experimental, and diagnostic test accuracy studies) or 0–5 (cohort studies). Any discrepancies were resolved by discussion or by consulting a third reviewer (HN). We used the quality scores to conduct sensitivity analysis.

### Statistical analysis

We conducted all statistical analyses in *R*, version 4.2.1 (R Core Team, Vienna, Austria). Since we anticipated significant heterogeneity, we adopted the DerSimonian and Laird random effects model [9]. We applied the Freeman-Tukey double arcsine transformation for all analyses except for the analysis on race/ethnicity as a risk factor for SARS-CoV-2 infections, which we synthesised narratively due to a lack of standardised data. The Freeman-Tukey double arcsine transformation enabled the inclusion of zero event estimates. The transformation also standardises the variance, which was appropriate to our analyses, as there was substantial heterogeneity across study estimates. We derived the pooled proportion as back-transformed values as the weighted mean of the transformed proportions to report all the pooled estimates with their 95% confidence intervals (CIs). We assessed heterogeneity by calculating the *I*^2^ and τ^2^ values. For risk factors, we calculated the pooled risk ratio (RR) with their 95% CIs. We developed forest plots to display results from individual studies along with the pooled estimates.

#### Test positivity estimates

We generated a pooled estimate for the proportion of children and adolescents testing positive for SARS-CoV-2 either by antigen tests or PCR among all children and adolescents that were tested in each study. All nucleic acid amplification tests were coded as PCR, as we estimated this to be the most widely used nucleic acid test across settings. We then performed a leave-one-out meta-analysis to investigate the influence of each study on the overall estimate and to identify influential studies, and also conducted a sensitivity analysis to determine the pooled estimate for the proportion of positive cases by including only good-quality studies. We conducted subgroup analyses to report the test positivity by setting (community, health care, educational institution, or other), age groups, type of diagnostic test, and geographical location.

##### By age group

We also performed subgroup analyses by two age groups (<5 and 5 to ≤18 years), in which we included any studies that reported age-stratified data for these two groups. We then further split these age groups into two narrower age bands (<5-year-old group into those aged <1 and 1 to <5 years; 5 to ≤18-year-old group into those aged 5 to <11 and 11 to 18 years). If a study reported data for groups that overlapped over subgroups within a group, we classified the data in a way that they fell in the group where most of their sample can be expected to fall in (for example, those <3 years old were included in the 1 to <5-year-old group) to maximise the inclusion of data in the analysis. Age groups that could not be classified into either of our groups (because they fell exactly in between) were placed in the higher age group (for example, the <2-year-old group was placed in the 1 to <5-year-old group).

##### By regions

To assess regional differences, we conducted subgroup analysis by WHO regions [10] and World Bank income levels [11].

##### By SARS-CoV-2 variants

To investigate the differential impact of SARS-CoV-2 variants on the proportion of positive cases, we utilised open-access data [12]. These data represented the proportion of the total number of sequences (not cases), over time, that fell into the defined variant groups. Countries were displayed if they had at least 70 sequences for any variant being tracked, over at least 4 weeks. We allotted the most dominant variant, i.e. the one with the highest proportion of the total number of sequences occurring at the mid-study period of each study (month and year), which we calculated in Microsoft Excel.

##### By study settings

We classified settings as community, health care institution, educational institution, or other. Health facility setting included all studies reporting data from primary, secondary, or tertiary health care facilities including hospitals, COVID-19 isolation/quarantine centres, general practitioner clinics, paediatric clinics including well child visits, intensive care units (ICUs), paediatric intensive care units (PICUs), and health centres delivering specialised care (such as burns centres, oncology centres, etc.). If the setting was mixed or unclear, we classified it as ‘other.’

##### By testing method

We conducted a subgroup analysis by testing method, i.e. PCR (including any nucleic acid tests), PCR or antigen tests, and antigen tests.

#### Risk factor analyses

We performed pooled analyses using risk factor data if at least two studies reported data on a particular risk factor. The pooled RRs were calculated with the 95% CI for each factor. Available data enabled us to conduct pooled analysis for age, sex, ethnicity, comorbidities, and pregnancy as risk factors for COVID-19 in people aged ≤18 years. We reported risk ratios for SARS-CoV-2 infection in four age groups (>1 year, 1 to <5 years, 5 to <10 years, and 10 to ≤18 years). However, the data reported in the studies were unstandardised; due to this heterogeneity, we classified the data from overlapping age bands so that they fell in the group where most of their sample can be expected to fall in. We compared the risk of SARS-CoV-2 infection in each of the groups to the other ages in the ≤18 years group. Some included studies also conducted adjusted analyses for several risk factors; in such cases, we were unable to pool the estimates of these adjusted analyses due to a lack of standardised data. Moreover, four studies reported on race or ethnicity; as we were unable to conduct a meta-analysis owing to the nature of these data (i.e. different groups of ethnicities/races reported in each study depending on their populations), we undertook a narrative synthesis for race or ethnicity as a risk for SARS-CoV-2 infection. We calculated the risk of SARS-CoV-2 infection in people having any of the following nine comorbidities: obesity, diabetes, immunosuppression, cardiovascular disease, asthma, epilepsy, renal disease, neurological condition, and congenital cardiac conditions.

#### Severity of disease

We assessed the severity of COVID-19 in children in two ways. We estimated the clinical management requirements (hospitalisation, ICU admissions, supplemental oxygen, and assisted ventilation) and provided a pooled estimate for the proportion of children (among those testing positive for SARS-CoV-2) requiring each of these. ICU admissions included ICU, PICU, and high dependency unit (HDU) admissions. Oxygen supplementation included all types of supplemental oxygen, including assisted ventilation, if details were unavailable. Assisted ventilation included all types of non-invasive and invasive mechanical ventilation, including continuous positive airway pressure, bilevel positive airway pressure, and tracheal intubation. If studies reported only on assisted ventilation without data on other types of supplemental oxygenation, we included them only for the analysis of assisted ventilation and not in the oxygen supplementation analysis. We included only studies that followed up at least 100 SARS-CoV-2 positive children and adolescents. We excluded studies that recruited only hospitalised children from the analysis of the proportion of children requiring hospitalisation. We could not assess the criteria for hospitalisation, ICU admission, and other requirements in individual studies, which is why we did not conduct further investigations and subgroup analyses based on WHO region or country income levels. As there were limited data on dominant variants and therefore, we did not conduct any statistical tests to explore differences between the effects on severity by variants.

We also assessed severity based on the clinical presentation (asymptomatic, mild, moderate, and severe/ critical disease). When studies combined two groups, we included the children from the combined group in the higher level of severity of the two groups (for example, moderate-severe disease cases were included in severe disease). We combined severe and critical COVID-19 disease as a single group due to overlapping definitions across studies and limited data on critical disease. The definitions of asymptomatic SARS-CoV-2 infections (without any clinical signs or symptoms), mild COVID-19 disease (upper respiratory infections), and moderate COVID-19 disease (non-severe pneumonia) were reasonably consistent across studies. If study authors did not report on the definitions used, these definitions were assumed.

#### Case fatality ratio

Lastly, we assessed the mortality outcomes in SARS-CoV-2 positive children. We calculated a pooled estimate of the proportion of SARS-CoV-2 positive children with fatal outcomes. The estimate likely included in-hospital and out-of-hospital deaths, as these details were rarely available in individual studies. To increase the confidence in our estimates, we only included studies that followed-up at least 100 SARS-CoV-2 children until the end of the study period or discharge or death (whichever occurred first). If studies differentiated between deaths caused due to COVID-19 and deaths due to other medical reasons in children and adolescents with SARS-CoV-2 infections, we extracted the data for deaths attributable to COVID-19 disease.

## RESULTS

**Figure 1:**
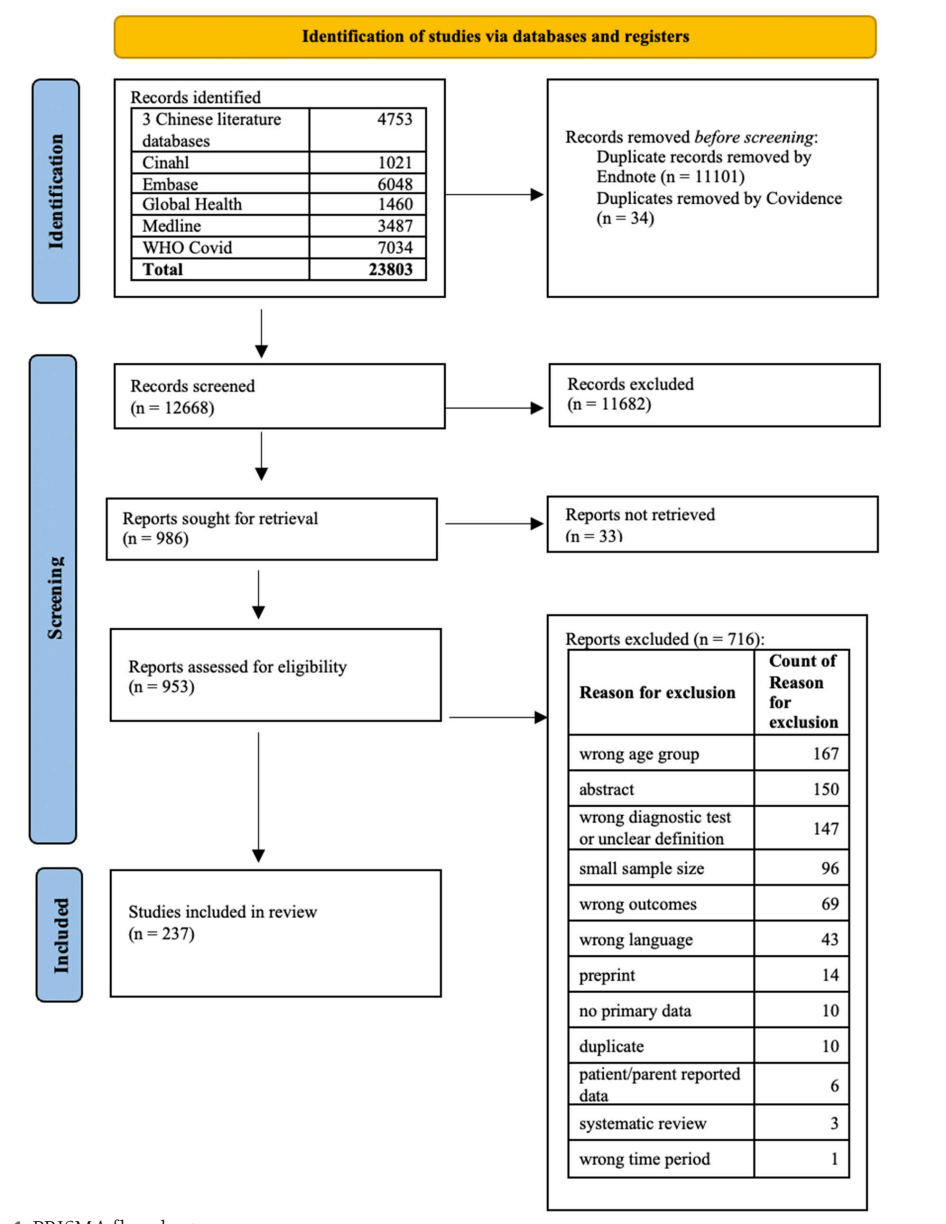
PRISMA flowchart.

We identified 19 050 records through our searches in eight (including three Chinese) academic literature databases. Following the screening process, we included 237 studies in this systematic review [13-249] (**Figure 1**; Table S11 in the **Online Supplementary Document**). Our searches did not yield any studies that were entirely conducted in the Omicron era or after Omicron became the dominant variant. (Table S9 in the **Online Supplementary Document**).

### Test positivity estimates

Based on 117 studies, we found the test positivity of SARS-CoV-2 to be 9.30% (95% CI = 7.15−11.73). The *I*^2^ and τ^2^ values suggested there was significant heterogeneity across the included study estimates. Three studies [55,88,241] were found to be influential in the estimates. The pooled SARS-CoV-2 test positivity after the exclusion of these studies was calculated as 8.13% (95% CI = 6.45−9.98, *I*^2^ = 100%, τ^2^ = 0.0300). When the analysis was restricted to good-quality studies only, the pooled SARS-CoV-2 test positivity was 9.04% (95% CI = 4.46−15.01, *I*^2^ = 99%; τ^2^ = 0.0172) (Figure S2 and Table S10 in the **Online Supplementary Document**).

#### Subgroup analysis of proportion positive estimates

**Figure 2:**
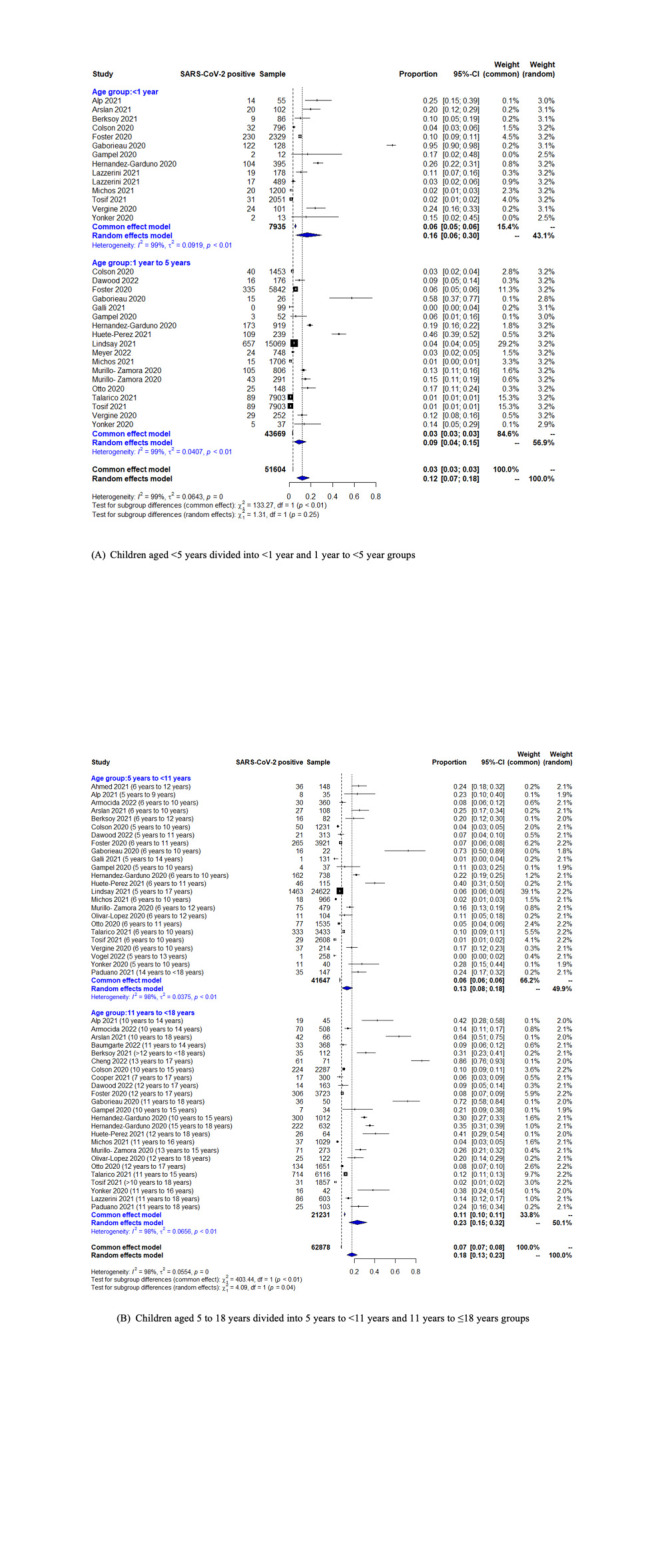
Forest plot for subgroup analysis of SARS-CoV-2 proportion positive in people aged ≤18 years. **Panel A.** Children aged <5 years divided into <1-year and 1 to <5-year-old groups. **Panel B.** Children aged 5 to 18 years divided into 5 to <11-year and 11 to ≤18-year-old groups.

**Figure 2** illustrates the findings of the subgroup analyses according to age groups. The results of the subgroup analysis of SARS-CoV-2 proportion positive in children and adolescents by the WHO regions, country income groups, study setting, and testing method are summarised in **Table 1** and the plots available in Figures S3−6 in the supplementary materials **Online Supplementary Document**. The estimates were highest in the African region, middle income countries, health facility settings and in studies employing PCR. The estimates were lowest in the Western Pacific Region, high income countries, educational institutions and studies employing antigen testing.

**Table 1:** Summary of results of subgroup analysis of SARS-CoV-2 test positivity in people aged ≤18 y

	Number of studies	Pooled estimate, (95% CI)	τ^2^
**WHO regions**			
European region	62	7.83 (5.14−11.01)	0.0468
Western Pacific region	11	4.57 (0.62−11.71)	0.0499
Region of the Americas	28	13.28 (7.92−19.76)	0.0551
Eastern Mediterranean region	4	9.04 (3.08−17.57)	0.0192
African region	4	14.39 (14.27−14.51)	0.0000
Southeast Asian region	6	12.67 (7.64−18.73)	0.0104
Mixed*	1	31.03 (30.15−31.93)	-
**Country income groups**			
High income	77	5.95 (3.89−8.41)	0.0443
Upper middle-income	28	17.15 (12.53−22.31)	0.0300
Lower middle-income	10	15.84 (9.70−23.13)	0. 0213
Mixed†	2	32.72 (28.94−36.61)	0.0007
**Study setting**			
Health facility	86	10.01 (7.24−13.17)	0.0532
Other setting‡	20	9.58 (5.85−14.11)	0.0255
Community	2	9.76 (6.17−14.07)	0.0019
Educational institution	9	3.38 (0.57−8.20)	0.0246
**Testing method**			
PCR	106	9.67 (7.30−12.34)*	0.0490
PCR or antigen test	10	6.57 (3.02−11.33)	0.0182
Antigen test	1	3.38 (2.75−4.08)	-

CI – confidence interval, PCR – polymerase chain reaction, WHO – World Health Organization

*There was one multicentre study [87] which was conducted across more than one WHO region. It was put in the ‘mixed’ group.

†There were two studies [87,232] reporting findings from more than one country and those were from different income groups. They formed a separate ‘mixed’ group.

‡Other setting included those in which children were testing was unclear or it could be expected to have data from more than one setting (health facility, educational institution, and community) This included studies that reported surveillance data or data from testing laboratories. However, the setting in which the children were tested was, however, unclear. Such studies were classified as conducted in other settings [64,65,68,75,89,97,110,129,137,162,171,175,192,196,197,215,218,220,225].

We were unable to assess the effect of different SARS-CoV-2 variants, as there were limited data on the dominant variant in each country at the mid-study period. Most of the studies included in this review were conducted during the early pandemic period. Consequently, we could not provide reliable proportion positive estimates by SARS-CoV-2 variant subgroups (Figure S7 in the **Online Supplementary Document**.

### Risk factors of SARS-CoV-2 infection in children aged ≤18 years

#### Age

We found the risk of SARS-CoV-2 infection to be lowest in the 1 to <5-year-old group (RR = 0.64; 95% CI = 0.49−0.83) compared with the remaining age groups. This was followed by the 5 to <10-year-old age group (RR = 0.82; 95% CI = 0.69−0.98) and the <1 year age group (RR = 0.88; 95% CI = 0.59−1.31). The risk was highest in those aged between 10 years and ≤18 years (RR = 1.60; 95% CI = 1.23−2.08). Eleven studies [14,22,34,38,50,103,121,143,152,155,200] reported the mean or median age or age range in SARS-CoV-2 positive and SARS-CoV-2 negative children and adolescents. However, the *P*-value was not reported in all studies. Five studies [33,38,79,134,159] controlled for confounders and reported adjusted risk ratios (Tables S1−3 in the **Online Supplementary Document**).

#### Sex

Twenty-one studies [14,22,33,34,50,63,79,103,107,109,121,130,134,143,152,154,155,159,166,175,200] reported data on sex as a risk factor for SARS-CoV-2 infection in children. The results of this meta-analysis showed no evidence of an association between sex and SARS-CoV-2 infection in children aged ≤18 years. Of these 21 studies, four [33,34,79,159] conducted multivariate analyses to estimate the association between SARS-CoV-2 infection and sex after adjusting for other factors (Figure S8 and Table S4 in the **Online Supplementary Document**).

#### Ethnicity or race

Six studies [38,63,143,152,200,246] reported data on ethnicity or race as a risk factor for SARS-CoV-2 infection in the ≤18 years population. The findings for non-Hispanic White, Black, and Hispanic groups were variable across studies. The association between being Asian and the risk of SARS-CoV-2 infection was found to be statistically insignificant in all studies reporting data on Asians. However, the RR estimated varied across studies and ranged between 1.15 and 5.10. Some race/ethnicity groups (Secular Jews, Arabs, Ultraorthodox Jews, multiracial non-Latino) were reported by single studies. Three studies were conducted in the United States [38,200,246], and one in Israel [63], Brazil [143] and Spain [152] each (Table S5 in the **Online Supplementary Document**).

#### Comorbidities

The findings of the meta-analysis of comorbidities as a risk factor for SARS-CoV-2 infection are presented in **Table 2**. The results of another analysis that included combined data on at least one of the following comorbidities – obesity, diabetes, immunosuppression, cardiovascular disease, asthma, epilepsy, renal disease, neurologic condition, or congenital heart disease – showed that having any of these comorbidities was a risk factor for SARS-CoV-2 infection (RR = 1.33; 95% CI = 1. −1.71, *P* < 0.001).

**Table 2:** Comorbidities as a risk for SARS-CoV-2 infection

Comorbidity	Case definitions used	Number of studies included	Total sample size	RR (95% CI)	Direction of association
Obesity	Obesity as a comorbidity or obesity based on BMI	7	13 325	2.91 (1.68−5.04)	Risk increase.
Diabetes	Diabetes, diabetes mellitus type 1	6	13 416	2.30 (1.23−4.28)	Risk increase.
Immunosuppression	Immunosuppressed, immunodeficiency, primary immunodeficiencies, immunosuppressant use, immune thrombocytopenia, and common variable immune deficiency	6	13 509	1.14 (0.57−2.30)	No evidence of association.
Cardiovascular disease	Disease, cardiovascular conditions, and cardiac disease	5	13 538	1.07 (0.54−2.13)	No evidence of association.
Asthma	Includes asthma, asthma or reactive airway disease, and asthma or chronic lung disease	7	300 402	0.73 (0.46−1.17)	No evidence of association.
Epilepsy		3	608	1.90 (1.14−3.17)	Risk increase.
Renal disease	Chronic renal insufficiency, chronic renal failure, end-stage renal disease, renal disease, kidney-related comorbidity	4	11 285	1.04 (0.77−1.40)	No evidence of association.
Neurologic condition	Malformation, disabilities, neuromuscular diseases, neurological diseases except for epilepsy, neurologic condition, neuromuscular damage, neurological comorbidity	6	3885	0.79 (0.41−1.52)	No evidence of association.
Congenital cardiac disease	Any congenital cardiac disease	3	704	0.71 (0.34−1.49)	No evidence of association.

BMI – body mass index, CI – confidence interval, RR – risk ratio

The results of adjusted analyses for some comorbidities were reported by six studies [63,79,103,134,159,200] (Table S6 in the **Online Supplementary Document**).

#### Pregnancy

Based on two studies conducted in Mexico with a total sample size of 8297 [31,103] meta-analysis did not show an association of pregnancy with SARS-CoV-2 infection in people aged ≤18 years (RR = 1.19; 95% CI = 0.73−1.92; *P* = 0.4807).

### Severity of SARS-CoV-2 infection in children and adolescents

#### Severity according to clinical presentation

**Figure 3:**
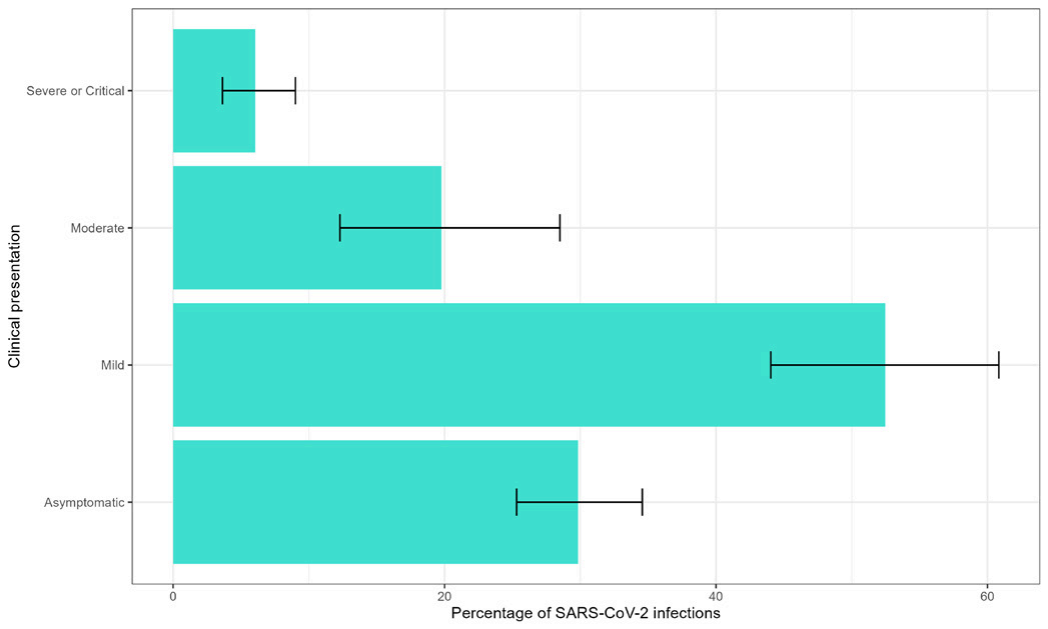
Severity of SARS-CoV-2 infection in children aged ≤18 years.

Mild COVID-19 disease was the most common presentation of SARS-CoV-2 infection in those aged ≤18 years (52.47%). Meanwhile, 29.83% children with SARS-CoV-2 infection remained asymptomatic, followed by those presenting with moderate disease (19.77%) and lastly with severe/critical disease (6.05%) (**Figure 3**). Not all studies reported number of cases with each of these four presentations (asymptomatic, mild, moderate, and severe/critical disease). Therefore, the denominator populations for each of these outcomes are not identical (Figures S9−12, Tables S7−8 in the **Online Supplementary Document**).

#### Severity according to management requirements

Among those testing positive, the percentage of children requiring hospitalisation was 20.70% (95% CI = 15.04−26.99). In view of oxygen supplementation, our analysis showed that about 7.19% (95% CI = 4.66−10.20) of those testing positive for SARS-CoV-2 required some kind of oxygen supplementation for the management of clinical symptoms. For ICU admission, 4.26% (95% CI = 2.90−5.58) of children and adolescents with SARS-CoV-2 infection required ICU or PICU or HDU admission. Finally, the pooled percentage of children and adolescents with SARS-CoV-2 infections requiring assisted ventilation was 2.92% (95% CI = 1.79−4.30) (Figures S13−16 in the **Online Supplementary Document**).

### Mortality outcomes

The pooled case fatality ratio was 0.87% (95% CI = 0.54−1.28) (Figure S17 in the **Online Supplementary Document**). Although most of these deaths can be considered as in-hospital deaths, these details, including the follow-up period, were unclear in most studies.

## DISCUSSION

We undertook this systematic review and meta-analysis to assess the burden and epidemiology of SARS-CoV-2 infections in people aged ≤18 years to help guide prevention policies and treatment recommendations. Our findings suggest that about 9.30% of children and adolescents tested positive for SARS-CoV-2 infection by PCR or antigen tests in the pre-Omicron era, while around 29.83% of infections were asymptomatic. Likewise, 21 in 100 people who were aged ≤18 years and tested positive for SARS-CoV-2 required hospitalisation, 4 in 100 required ICU admission, and 1 in 100 resulted in death.

Our study showed that the pooled proportion positive estimates were slightly higher in studies only using PCR for diagnosis (9.67%) when compared with all included studies (9.30%). Well-designed large studies and surveillance systems are needed to better understand the true burden of COVID-19 in the paediatric population. Additionally, laboratory testing of SARS-CoV-2 should not be limited to those with symptoms of COVID-19 since at least about one-third of SARS-CoV-2 infected persons remain asymptomatic [250,251]. Without robust data on a representative sample for each country, we will not understand the true magnitude of COVID-19 and transmission patterns at the local and global levels. Importantly, early diagnosis and confirmation of infection will allow individuals to seek health care, therefore reducing disease severity and death.

Variability in sampling strategy and testing policies may have contributed to significant heterogeneity across studies. For example, three influential studies providing unusually high proportions of SARS-CoV-2 positives enrolled individuals within two weeks of exposure to a laboratory-confirmed COVID-19 household contact into the Household Exposure and Respiratory Virus Transmission and Immunity Study (HEARTS) with a convenient recruitment strategy [55] and evaluated a sample of children with a suspicion [241] or high suspicion of COVID-19, respectively [88]. Most studies conducted testing of all children visiting the health centre or educational institution, or individuals having symptoms, or a known contact with a COVID-19 case, or having residence in a geographical area of high COVID-19 incidence.

Our meta-analysis showed that the RR of SARS-CoV-2 positive test was higher in those aged 10 to ≤18 years compared to those <10 years. The number of COVID-19 cases and population by age-groups reported by the UN reflects a similar trend [6]. Globally, males have been shown to be at higher risk of severe COVID-19 disease and adverse outcomes compared to females, yet there does not seem to be strong evidence that suggests that test positivity is significantly higher in males compared to females in individuals of all ages [252]. We also did not detect an association of sex with SARS-CoV-2 test positivity (RR = 0.96; 95% CI = 0.87−1.05) in children and adolescents. Evidence on race/ethnicity as a risk factor for SARS-CoV-2 infections in this population was limited, with wide CIs, while the studies were reported from different countries and thus may not be directly comparable. Moreover, the findings must be interpreted with a caveat, since we did not adjust for demographic factors such as age, sex, deprivation, household size, and underlying health conditions in this analysis. Existing evidence, not limited to the paediatric and adolescent population, indicates that some ethnic communities are disproportionately affected by higher SARS-CoV-2 infection rates and adverse outcomes, and these disparities can often be attributed to pre-existing social inequalities [253]. Adults and children with certain underlying medical conditions are at higher risk of COVID-19 [254]. We found that having at least one of the following comorbidities – obesity, diabetes, immunosuppression, cardiovascular disease, asthma, epilepsy, renal disease, neurologic condition, or congenital heart disease – increased SARS-CoV-2 infection risk by 33% in people aged ≤18 years. In our meta-analysis, we did not observe a statistically significant association between immunosuppression and the risk of SARS-CoV-2 infection. It is plausible that this patient population was likely to have taken more precautions and preventive measures such as shielding, which may have confounded this association. Pregnancy and other comorbidities, like neurological disease, congenital cardiac disease, and renal disease, did not show a statistically significant association with SARS-CoV-2 infection. Like those immunosuppressed patients, pregnant women and patients living with these conditions can be expected to have had a heightened degree of precaution and better compliance to non-pharmacological interventions, particularly in the early pandemic period, when most of the data were collected. Thus, population behaviour factors that were beyond the scope of this systematic review may have confounded this association.

We estimated that about 20.70% of people aged ≤18 years who were positive for SARS-CoV-2 required admission to the hospital, but only 6.05% had severe or critical disease. This suggests that the criteria for hospitalisation were likely influenced by several other factors apart from the severity of COVID-19 disease. Importantly, criteria for hospitalisation were highly heterogeneous in different countries and changed over time. This discrepancy could be attributed to the fact that most countries adopted a very cautious approach at the beginning of the pandemic and set guidelines to hospitalise patients for monitoring and control of infection (isolation) rather than as a requirement for the management of the disease. Besides, we did not explore if the necessity for hospitalisation, ICU admission, oxygen supplementation, and mechanical ventilation was primarily driven by COVID-19 disease or other factors such as underlying health conditions. Therefore, these estimates should not be interpreted as hospitalisation, ICU admission, oxygen supplementation, and mechanical ventilation attributable to COVID-19 disease severity and are likely overestimates. Our findings suggest that about 0.87% of SARS-CoV-2 positive children had fatal outcomes. Children and adolescents with severe and critical disease are more likely to die than those with asymptomatic infection or mild and moderate COVID-19 disease. Only 35 studies reported the number of children and adolescents progressing to critical disease, and it may be expected that these studies were conducted in tertiary health facilities that have equipment and manpower to manage critically ill patients. For studies conducted in other settings, like communities, educational institutions, or primary health facilities, the case fatality ratio, requirement of oxygen supplementation, ICU admission, and assisted ventilation can be expected to be lower, as it is unlikely to have people with critical COVID-19 in these settings. It remains to be seen how the case fatality ratio changes according to disease severity and it would be of particular interest to see the disease severity estimates and death in children with specific underlying conditions. Again, most studies reported the case fatality ratios rather than the disease-attributable case fatality ratios. The estimate is, therefore, likely an overestimate of the true mortality estimate.

Our test-positive estimate of 9.30% is higher than the estimate provided by Jain et al. [255] for influenza in the same population (7.00%) between 2010 and 2012. Their estimates for respiratory syncytial virus, meanwhile, were higher (28.00%) compared to our estimates for SARS-Cov-2 in children aged ≤18 years. However, their study population was restricted to hospitalised children with a specific case definition of acute respiratory infections with radiologic evidence of pneumonia. One would expect higher chances of detection of respiratory virus in such populations presenting with acute respiratory illnesses and evidence of pneumonia as compared to our study population, which also included asymptomatic people who may or may not have had contact with a SARS-CoV-2 positive individual. Our study population was not restricted to those with acute respiratory illnesses, as we also wanted to estimate the proportion of cases with asymptomatic and mild disease presentation. The pooled proportion positive for influenza among hospitalised children and adolescents aged ≤18 years with respiratory illness was reported to be 9.50% (95% CI = 8.10−11.00) between 1982 and 2012 in another study [256]. Although we did not conduct a subgroup analysis for hospitalised patients, our subgroup analysis by study setting showed comparable estimates (10.01%; 95% CI = 7.24−13.17) in studies conducted in health facility settings (which also included outpatients). Considering the risk of COVID-19 is comparable to or higher than influenza, routine COVID-19 vaccination may need to be considered in children and adolescents, at risk of severe illness, as has been done in the case of influenza; moreover, COVID-19 vaccines such as mRNA vaccines have been shown to reduce both infection and severe outcomes such as hospitalisation and death in the paediatric population and have been demonstrated to be safe [257].

This is, to the best of our knowledge, the first systematic review to assess the global data on SARS-CoV-2 infections in children and adolescents. The study was guided by PRISMA-P guidelines and followed a predetermined protocol. We adopted a sensitive search strategy to retrieve relevant publications. However, our review has some limitations. We adopted a single reviewer screening approach that can lead to bias and increase the chance of human error. The differences in testing criteria and selection for inclusion of participants in individual studies led to very high heterogeneity in the included study estimates (*I*^2^ = 100%, τ^2^ = 0 · 0460 for the test positivity estimates). We were unable to provide reliable estimates for subgroup analysis by SARS-CoV-2 variant, as most studies were conducted before reliable and large-scale genomic sequencing data became available. We did not explore the effect of non-pharmaceutical interventions or COVID-19 vaccination in children on our estimates. The vaccination policies were, again, highly variable across countries, including the type of vaccine, age criteria and the time of start of vaccination programmes. Lastly, we included no studies conducted in low-income countries in the proportion positive analysis. The two studies that were conducted across multiple countries also reported data either from high- or middle-income countries. This paucity of data also translates into vaccine coverage in low-income countries, as 69.20% of the global population had received at least one dose of the COVID-19 vaccine vs only 25.90% of people living in low-income countries as of 18 January 2023 [258]. Investment in research and surveillance activities in low-income countries is, therefore, warranted to help inform vaccination and other prevention policies and management strategies.

There were limited and unstandardised data on the long-term sequelae or complications of COVID-19 disease, so we did not undertake a synthesis for these outcomes. The portrayal of the burden of disease is incomplete without estimating the long-term sequelae or the incidence of long COVID-19 in this age group which seems to be more common than previously believed. This is a critical factor in comparing the risks and benefits of vaccinations, particularly because presentation with mild disease is very common in this age group [259]. Our analysis does not consider the impact of vaccination policies or non-pharmacological interventions and other preventive measures that were implemented or relaxed by different countries at different times and that had a likely impact on the burden of disease.

## CONCLUSIONS

The detection of SARS-CoV-2 in children is not uncommon. Although many of the paediatric cases presented with asymptomatic or mild forms of disease with complete recovery, there is a considerable proportion of those requiring hospitalisation, oxygen support, and ICU admission. Moreover, it is essential to systematically investigate the long-term sequelae or incidence of long COVID-19 in this population and assess the differential impact of SARS-CoV-2 variants. Given the importance of COVID-19 in this population, more investment in research, diagnostics, treatment, and vaccines for this population is warranted.

## Additional material


Online Supplementary Document

## References

[1] Cucinotta D Vanelli M WHO Declares COVID-19 a Pandemic. Acta Biomed. 2020;91:157–60. 32191675 10.23750/abm.v91i1.9397 PMC7569573

[2] World Health Organization. WHO Coronavirus (COVID-19) Dashboard. 2021. Available: https://covid19.who.int/. Accessed: 5 February 2024.

[3] World Health Organization. Coronavirus disease (COVID-19): Variants of SARS-COV-2. 2021. Available: https://www.who.int/emergencies/diseases/novel-coronavirus-2019/question-and-answers-hub/q-a-detail/coronavirus-disease-%28covid-19%29-variants-of-sars-cov-2?gclid=EAIaIQobChMI7LeX1Y_P_AIVR4BQBh02QA1VEAAYASAAEgI2rfD_BwE. Accessed: 5 February 2024.

[4] United Nations Children’s Fund. Convention on the Rights of the Child. 1989. Available: https://www.ohchr.org/en/instruments-mechanisms/instruments/convention-rights-child#:~:text=PART%20I-,Article%201,child%2C%20majority%20is%20attained%20earlier. Accessed: 5 February 2024.

[5] United Nations Children’s Fund. Investing in a safe, healthy and productive transition from childhood to adulthood is critical. 2022. Available: https://data.unicef.org/topic/adolescents/overview/. Accessed: 5 February 2024.

[6] United Nations Children’s Fund. COVID-19 confirmed cases and deaths, age- and sex-disaggregated data. 2022. Available: https://data.unicef.org/resources/covid-19-confirmed-cases-and-deaths-dashboard/. Accessed: 5 February 2024.

[7] Page MJ McKenzie JE Bossuyt PM Boutron I Hoffmann TC Mulrow CD The PRISMA 2020 statement: an updated guideline for reporting systematic reviews. Syst Rev. 2021;10:89. 10.1186/s13643-021-01626-4 33781348 PMC8008539

[8] Joanna Briggs Institute. Critical appraisal tools. Available: https://jbi.global/critical-appraisal-tools. Accessed: 5 February 2024.

[9] DerSimonian R Kacker R Random-effects model for meta-analysis of clinical trials: an update. Contemp Clin Trials. 2007;28:105–14. 10.1016/j.cct.2006.04.004 16807131

[10] World Health OrganizationCountries. 2023. Available: https://www.who.int/countries. Accessed: 5 February 2024.

[11] The World Bank. World Bank Country and Lending Groups. 2022. Available: https://datahelpdesk.worldbank.org/knowledgebase/articles/906519-world-bank-country-and-lending-groups. Accessed: 5 February 2024.

[12] Hodcroft EB. Overview of Variants in Countries. 2021. Available: https://covariants.org/per-country. Accessed: 5 February 2024.

[13] Abo YN Clifford V Lee LY Costa AM Crawford N Wurzel D COVID-19 public health measures and respiratory viruses in children in Melbourne. J Paediatr Child Health. 2021;57:1886–92. 10.1111/jpc.15601 34080245 PMC8242487

[14] Ahmed GK Elbeh K Gomaa HM Soliman S Does COVID-19 infection have an impact on children’s psychological problems? Middle East Current Psychiatry. 2021;28:77. 10.1186/s43045-021-00155-z

[15] Aizawa Y Shobugawa Y Tomiyama N Nakayama H Takahashi M Yanagiya J Coronavirus Disease 2019 Cluster Originating in a Primary School Teachers’ Room in Japan. Pediatr Infect Dis J. 2021;40:e418. 10.1097/INF.0000000000003292 34561385 PMC8505149

[16] Akkoç G Akgün Ö Kızılırmak C Yıldız F Duru HNS Elevli M Demographic and Clinical Characteristics of COVID-19 in Children and the Effect of Household Tobacco Smoke Exposure on COVID-19. Turk Arch Pediatr. 2021;56:322. 10.5152/TurkArchPediatr.2021.20226 35005725 PMC8655958

[17] Alattas B Azaz A Rawat D Miqdady M Bitar R Clinical manifestations and outcome in children with COVID-19 infection in Abu Dhabi: a retrospective single-centre study. BMJ Paediatr Open. 2021;5:e001219. 10.1136/bmjpo-2021-001219 34841090 PMC8609498

[18] AlGhamdi A Al Talhi Y Al Najjar A Sobhi A Al Juaid A Ibrahim A Epidemiology, clinical characteristics and risk factors of COVID-19 among children in Saudi Arabia: a multicenter chart review study. BMC Pediatr. 2022;22:86. 10.1186/s12887-021-02959-8 35151286 PMC8840071

[19] Alharbi M Kazzaz YM Hameed T Alqanatish J Alkhalaf H Alsadoon A SARS-CoV-2 infection in children, clinical characteristics, diagnostic findings and therapeutic interventions at a tertiary care center in Riyadh, Saudi Arabia. J Infect Public Health. 2021;14:446–53. 10.1016/j.jiph.2020.12.034 33765595 PMC7833958

[20] Almuzaini Y Alsohime F Al Subaie S Temsah MH Alsofayan Y Alamri F Clinical profiles associated with SARS-CoV-2 infection and complications from coronavirus disease-2019 in children from a national registry in Saudi Arabia. Ann Thorac Med. 2021;16:280. 10.4103/atm.atm_709_20 34484444 PMC8388572

[21] Alonso GT Ebekozien O Gallagher MP Rompicherla S Lyons SK Choudhary A Diabetic ketoacidosis drives COVID-19 related hospitalizations in children with type 1 diabetes. J Diabetes. 2021;13:681–7. 10.1111/1753-0407.13184 33855813 PMC8251108

[22] Alp EE Dalgic N Yilmaz V Altuntas Y Ozdemir HM Evaluation of patients with suspicion of COVID-19 in pediatric emergency department. The Medical Bulletin of Sisli Etfal Hospital. 2021;55:179. 34349593 10.14744/SEMB.2021.03360 PMC8298069

[23] Alqayoudhi A Al Manji A Al khalili S Al Maani A Alkindi H Alyaquobi F The role of children and adolescents in the transmission of SARS-CoV-2 virus within family clusters: A large population study from Oman. J Infect Public Health. 2021;14:1590–4. 10.1016/j.jiph.2021.09.008 34627056 PMC8442293

[24] Alsharrah D Alhaddad F Alyaseen M Aljamaan S Almutairi N Ayed M Clinical characteristics of pediatric SARS-CoV-2 infection and coronavirus disease 2019 (COVID-19) in Kuwait. J Med Virol. 2021;93:3246–50. 10.1002/jmv.26684 33219559 PMC7753270

[25] Alshengeti A Alahmadi H Barnawi A Alfuraydi N Alawfi A Al-Ahmadi A Epidemiology, clinical features, and outcomes of coronavirus disease among children in Al-Madinah, Saudi Arabia: A retrospective study. Int J Pediatr Adolesc Med. 2022;9:136–42. 10.1016/j.ijpam.2021.11.001 35663790 PMC9152574

[26] Alshukairi AN Doar H Al-Sagheir A Bahasan MA Sultan AA Al Hroub MK Outcome of COVID19 in patients with Osteogenesis Imperfecta: A retrospective multicenter study in Saudi Arabia. Front Endocrinol (Lausanne). 2022;12:800376. 10.3389/fendo.2021.800376 35095767 PMC8792853

[27] Ana Laura GO Abraham Josué NR Briceida LM Israel PO Tania AF Nancy MR Sensitivity of the Molecular Test in Saliva for Detection of COVID-19 in Pediatric Patients With Concurrent Conditions. Front Pediatr. 2021;9:642781. 10.3389/fped.2021.642781 33912522 PMC8071854

[28] Antúnez-Montes OY Escamilla MI Figueroa-Uribe AF Arteaga-Menchaca E Lavariega-Saráchaga M Salcedo-Lozada P COVID-19 and multisystem inflammatory syndrome in Latin American children: a multinational study. Pediatr Infect Dis J. 2021;40:e1–6. 10.1097/INF.0000000000002949 33055501

[29] Anugulruengkitt S Teeraananchai S Chantasrisawad N Promsena P Jantarabenjakul W Puthanakit T Clinical outcomes of pediatric COVID-19 in a tertiary care center in Bangkok, Thailand. IJID Reg. 2021;1:159–62. 10.1016/j.ijregi.2021.11.003 35721777 PMC8600753

[30] Aprea V Debaisi GE Guedes V Guglielmo MC Miño L Stabilito LA Out-of-hospital care setting in a Febrile Emergency Unit of suspected Covid 19 patients. Andes Pediatr. 2021;92:677–82. 10.32641/andespediatr.v92i5.3422 35319573

[31] Arellano-Llamas AA Hernández-Caballero Á [COVID-19 in Mexican children and adolescents until May 10th, 2020. A focus on patients with diabetes]. Rev Mex Endocrinol Metab Nutr. 2020;7:80–6. Spanish.

[32] Arivoli Kaliyan SP Prasanna S Thirumalaikumarasamy S Ethirajan N Thandavarayan M Jeganathan S Clinical and Epidemiological Profile of Children with COVID-19 in a Tertiary Care Centre in Tamil Nadu, India. J Clin Diagn Res. 2021;15:SC10–4.

[33] Armocida B Zamagni G Magni E Monasta L Comar M Zanotta N Clinical, anamnestic, and sociodemographic predictors of positive SARS-CoV-2 testing in children: A cross sectional study in a tertiary hospital in Italy. PLoS One. 2022;17:e0262923. 10.1371/journal.pone.0262923 35077483 PMC8789147

[34] Arslan G Aktürk H Duman M Clinical characteristics of pediatric coronavirus disease 2019 and predictors of polymerase chain reaction positivity. Pediatr Int. 2021;63:1055–61. 10.1111/ped.14602 33426754 PMC8013810

[35] Asseri AA Alzaydani I Al-Jarie A Albishri A Alsabaani A Almaghrabi MK Clinical characteristics and laboratory abnormalities of hospitalized and critically ill children with coronavirus disease 2019: a Retrospective Study from Saudi Arabia. Int J Gen Med. 2021;14:1949. 10.2147/IJGM.S311831 34040426 PMC8141390

[36] Ayed A Embaireeg A Benawadh A Al-Fouzan W Hammoud M Al-Hathal M Maternal and perinatal characteristics and outcomes of pregnancies complicated with COVID-19 in Kuwait. BMC Pregnancy Childbirth. 2020;20:754. 10.1186/s12884-020-03461-2 33267785 PMC7709095

[37] Bai Y Gao L Wang X Zhong L Li J Ding S Epidemiological characteristics and clinical manifestations of pediatric patients with COVID-19 in China: A multicenter retrospective study. Pediatr Investig. 2021;5:203–10. 10.1002/ped4.12282 34540320 PMC8441896

[38] Bandi S Nevid MZ Mahdavinia M African American children are at higher risk of COVID-19 infection. Pediatr Allergy Immunol. 2020;31:861–4. 10.1111/pai.13298 32469426 PMC7283894

[39] Barrera CM Hazell M Chamberlain AT Gandhi NR Onwubiko U Liu CY Retrospective cohort study of COVID-19 among children in Fulton County, Georgia, March 2020–June 2021. BMJ Paediatr Open. 2021;5:e001223. 10.1136/bmjpo-2021-001223 35471855 PMC8671844

[40] Baumgarte S Hartkopf F Hölzer M von Kleist M Neitz S Kriegel M Investigation of a Limited but Explosive COVID-19 Outbreak in a German Secondary School. Viruses. 2022;14:87. 10.3390/v14010087 35062291 PMC8780098

[41] Bayesheva D Boranbayeva R Turdalina B Fakhradiyev I Saliev T Tanabayeva S COVID-19 in the paediatric population of Kazakhstan. Paediatr Int Child Health. 2021;41:76–82. 10.1080/20469047.2020.1857101 33315538

[42] Bellino S Punzo O Rota MC Del Manso M Urdiales AM Andrianou X COVID-19 disease severity risk factors for pediatric patients in Italy. Pediatrics. 2020;146:e2020009399. 10.1542/peds.2020-009399 32665373

[43] Berksoy E Kanik A Cicek A Bardak Ş Elibol P Demir G Clinical and laboratory characteristics of children with SARS-CoV-2 infection. Pediatr Pulmonol. 2021;56:3674–81. 10.1002/ppul.25654 34516721 PMC8661911

[44] Besli GE Demir SÖ Girit S Arman T Duyu M Arslanoglu S COVID-19 in children: a single center experience from Istanbul, Turkey. Medical Journal of Bakirkoy. 2021;17:64–71.

[45] Biko DM Ramirez-Suarez KI Barrera CA Banerjee A Matsubara D Kaplan SL Imaging of children with COVID-19: experience from a tertiary children’s hospital in the United States. Pediatr Radiol. 2021;51:239–47. 10.1007/s00247-020-04830-x 32945888 PMC7498743

[46] Bolaños-Almeida CE Espitia Segura OM Clinical and Epidemiologic Analysis of COVID-19 Children Cases in Colombia PEDIACOVID. Pediatr Infect Dis J. 2021;40:e7–11. 10.1097/INF.0000000000002952 33093428

[47] Brotons P Jordan I Bassat Q Henares D Fernandez de Sevilla M Ajanovic S The positive rhinovirus/enterovirus detection and SARS-CoV-2 persistence beyond the acute infection phase: an intra-household surveillance study. Viruses. 2021;13:1598. 10.3390/v13081598 34452462 PMC8402816

[48] Buonsenso D Munblit D De Rose C Sinatti D Ricchiuto A Carfi A Preliminary evidence on long COVID in children. Acta Paediatr. 2021;110:2208–11. 10.1111/apa.15870 33835507 PMC8251440

[49] Buta G, Cojocaru S, Costru T, Puia R, Abdusa-Ganea D, Ungureanu A. Clinical-Epidemiological Characteristics of Children Hospitalized with COVID-19 in the Republic of Moldova. In: Tignyanu I, Sontea V, Railean S, editors. 5th International Conference on Nanotechnologies and Biomedical Engineering. Proceedings of ICNBME-2021; 2021 November 3-5. Chisinau, Moldova. Cham: Springer International Publishing; 2022. p. 706-711.

[50] Calvani M Cantiello G Cavani M Lacorte E Mariani B Panetta V Reasons for SARS-CoV-2 infection in children and their role in the transmission of infection according to age: a case-control study. Ital J Pediatr. 2021;47:193. 10.1186/s13052-021-01141-1 34579754 PMC8474731

[51] Capozza M Salvatore S Baldassarre ME Inting S Panza R Fanelli M Perinatal Transmission and Outcome of Neonates Born to SARS-CoV-2-Positive Mothers: The Experience of 2 Highly Endemic Italian Regions. Neonatology. 2021;118:665–71. 10.1159/000518060 34628414 PMC8678243

[52] Capra DR Ucha G Antoni E Malfetano A Wolfsteiner N Arias P Experience at the Department of Pediatrics of a private facility in the Metropolitan Area of Buenos Aires during the COVID-19 pandemic. Arch Argent Pediatr. 2021;119:310–6. 34569738 10.5546/aap.2021.eng.310

[53] Carrasco I Muñoz-Chapuli M Vigil-Vázquez S Aguilera-Alonso D Hernández C Sánchez-Sánchez C SARS-COV-2 infection in pregnant women and newborns in a Spanish cohort (GESNEO-COVID) during the first wave. BMC Pregnancy Childbirth. 2021;21:326. 10.1186/s12884-021-03784-8 33902483 PMC8072086

[54] Çelik B Doğan M İnan DB Sunkak S Saatçi E Tubaş F Demographic and laboratory findings of symptomatic and asymptomatic COVID-19 in children. Guncel Pediatri. 2021;19:280–4. 10.4274/jcp.2021.47640

[55] Cheng WA Turner L Marentes Ruiz CJ Tanaka ML Congrave-Wilson Z Lee Y Clinical manifestations of COVID-19 differ by age and obesity status. Influenza Other Respir Viruses. 2022;16:255–64. 10.1111/irv.12918 34668322 PMC8652925

[56] Chiang C-Y Ellwood P Ellwood E García-Marcos L Masekela R Asher I Infection with SARS-CoV-2 among children with asthma: evidence from Global Asthma Network. Pediatr Allergy Immunol. 2022;33:e13709. 10.1111/pai.13709 34856034

[57] Chopra S Saha A Kumar V Thakur A Pemde H Kapoor D Acute Kidney Injury in Hospitalized Children with COVID19. J Trop Pediatr. 2021;67:fmab037. 10.1093/tropej/fmab037 34080011 PMC8195178

[58] Chowdhoury SR Biswas HS Raut S Bhakta S Roy A Banerjee S Pediatric Oncology Patients and COVID-19: An Experience from the Tertiary COVID Care Facility in Eastern India: A Prospective Observational Study. Indian J Med Paediatr Oncol. 2021;42:130–4. 10.1055/s-0041-1732814

[59] Chua GT Wong JSC Lam I Ho PPK Chan WH Yau FYS Clinical Characteristics and Transmission of COVID-19 in Children and Youths During 3 Waves of Outbreaks in Hong Kong. JAMA Netw Open. 2021;4:e218824. 10.1001/jamanetworkopen.2021.8824 33938934 PMC8094012

[60] Ciofi Degli Atti ML Campana A Muda AO Concato C Ravà L Ricotta L Facing SARS-CoV-2 Pandemic at a COVID-19 Regional Children’s Hospital in Italy. Pediatr Infect Dis J. 2020;39:e221–5. 10.1097/INF.0000000000002811 32639459

[61] Cloete J Kruger A Masha M du Plessis NM Mawela D Tshukudu M Paediatric hospitalisations due to COVID-19 during the first SARS-CoV-2 omicron (B. 1.1. 529) variant wave in South Africa: a multicentre observational study. Lancet Child Adolesc Health. 2022;6:294–302. 10.1016/S2352-4642(22)00027-X 35189083 PMC8856663

[62] Cofré F Mackenney J Poli C Riquelme M Carvajal C Álvarez P [Clinical manifestations of SARS-CoV-2 infection in children in the middle of pandemic season in a pediatric tertiary center. Report of local COVID Clinical Committee, Hospital de Niños Roberto del Río, Santiago Chile]. Rev Chilena Infectol. 2020;37:756–61. Spanish. 33844817 10.4067/S0716-10182020000600756

[63] Cohen HA Gerstein M Yaniv N Richenberg Y Jacobson E Marton S Attention-Deficit/Hyperactivity Disorder as a Risk Factor for COVID-19 Infection. J Atten Disord. 2022;26:985–90. 10.1177/10870547211044217 34668429

[64] Colson P Esteves-Vieira V Giraud-Gatineau A Zandotti C Filosa V Chaudet H Temporal and age distributions of SARS-CoV-2 and other coronaviruses, southeastern France. Int J Infect Dis. 2020;101:121–5. 10.1016/j.ijid.2020.09.1417 32976991 PMC7511210

[65] Colson P Tissot-Dupont H Morand A Boschi C Ninove L Esteves-Vieira V Children account for a small proportion of diagnoses of SARS-CoV-2 infection and do not exhibit greater viral loads than adults. Eur J Clin Microbiol Infect Dis. 2020;39:1983–7. 10.1007/s10096-020-03900-0 32845413 PMC7447969

[66] Cooper DM Zulu MZ Jankeel A Ibraim IC Ardo J Kasper K SARS-CoV-2 acquisition and immune pathogenesis among school-aged learners in four diverse schools. Pediatr Res. 2021;90:1073–80. 10.1038/s41390-021-01660-x 34304252 PMC8308070

[67] Corso MCM Soares VJ Amorim AMP Cipolotti R Magalhães IMQ Lins MM SARS-CoV-2 in children with cancer in Brazil: Results of a multicenter national registry. Pediatr Blood Cancer. 2021;68:e29223. 10.1002/pbc.29223 34288386 PMC8441618

[68] Dash GC Subhadra S Turuk J Parai D Rout UK Rath S COVID-19 in children in Odisha state, India: a retrospective review. BMJ Paediatr Open. 2021;5:e001284. 10.1136/bmjpo-2021-001284 34754950 PMC8568531

[69] Dawood FS Porucznik CA Veguilla V Stanford JB Duque J Rolfes MA Incidence Rates, Household Infection Risk, and Clinical Characteristics of SARS-CoV-2 Infection Among Children and Adults in Utah and New York City, New York. JAMA Pediatr. 2022;176:59–67. 10.1001/jamapediatrics.2021.4217 34623377 PMC8501415

[70] de Lusignan S Dorward J Correa A Jones N Akinyemi O Amirthalingam G Risk factors for SARS-CoV-2 among patients in the Oxford Royal College of General Practitioners Research and Surveillance Centre primary care network: a cross-sectional study. Lancet Infect Dis. 2020;20:1034–42. 10.1016/S1473-3099(20)30371-6 32422204 PMC7228715

[71] Devrim İ Böncüoğlu E Kıymet E Şahinkaya Ş Çelebi MY Cem E Comparison of the pediatric hospitalizations due to COVID-19 and H1N1pdm09 virus infections during the pandemic period. J Med Virol. 2022;94:2055–9. 10.1002/jmv.27589 35023188 PMC9015540

[72] Dilber B Aydın ZGG Yeşilbaş O Sağ E Aksoy NK Gündoğmuş F Neurological Manifestations of Pediatric Acute COVID Infections: A Single Center Experience. J Trop Pediatr. 2021;67:fmab062. 10.1093/tropej/fmab062 34254129 PMC8344731

[73] Domínguez Rojas J Estupiñan Vigil M Garcés-Ghilardi R Alvarado-Gamarra G Del Águila O Lope Tenorio AF [Cross-sectional study of the clinical characteristics and outcomes of children hospitalized with COVID-19 in Lima, Peru]. Medwave. 2021;21:e8107. Spanish. 10.5867/medwave.2021.01.8107 33617519

[74] Domínguez-Rodríguez S Villaverde S Sanz-Santaeufemia FJ Grasa C Soriano-Arandes A Saavedra-Lozano J A Bayesian Model to Predict COVID-19 Severity in Children. Pediatr Infect Dis J. 2021;40:e287–93. 10.1097/INF.0000000000003204 34250967

[75] Dong Y Mo X Hu Y Qi X Jiang F Jiang Z Epidemiology of COVID-19 Among Children in China. Pediatrics. 2020;145:e20200702. 10.1542/peds.2020-0702 32179660

[76] Du H Dong X Zhang J-j Cao Y-y Akdis M Huang P-q Clinical characteristics of 182 pediatric COVID-19 patients with different severities and allergic status. Allergy. 2021;76:510–32. 10.1111/all.14452 32524611 PMC7307120

[77] Eleftheriou I Dasoula F Dimopoulou D Lebessi E Serafi E Spyridis N Real-life evaluation of a COVID-19 rapid antigen detection test in hospitalized children. J Med Virol. 2021;93:6040–4. 10.1002/jmv.27149 34156112 PMC8427014

[78] Elghoudi A Aldhanhani H Ghatasheh G Sharif E Narchi H Covid-19 in Children and Young Adolescents in Al Ain, United Arab Emirates- a Retrospective Cross-Sectional Study. Front Pediatr. 2021;8:603741. 10.3389/fped.2020.603741 33537264 PMC7848192

[79] Söbü E Karaaslan A Çetin C Yasemin A Vitamin D levels of COVID-19 positive sypmtomatic pediatric cases. Güncel Pediatri. 2021;19:9–14. 10.4274/jcp.2021.0002

[80] Engels G Sack J Weissbrich B Hartmann K Knies K Härtel C Very Low Incidence of SARS-CoV-2, Influenza and RSV but High Incidence of Rhino-, Adeno- and Endemic Coronaviruses in Children With Acute Respiratory Infection in Primary Care Pediatric Practices During the Second and Third Wave of the SARS-CoV-2 Pandemic. Pediatr Infect Dis J. 2022;41:e146–8. 10.1097/INF.0000000000003460 35175993 PMC8919947

[81] Ennab F ElSaban M Khalaf E Tabatabaei H Khamis AH Devi BR Clinical Characteristics of Children With COVID-19 in the United Arab Emirates: Cross-sectional Multicenter Study. JMIR Pediatr Parent. 2021;4:e29049. 10.2196/29049 34643535 PMC8575012

[82] Ergenc Z Kepenekli E Çetin E Ersoy A Korkmaz B Selçik R Incidence of multisystem inflammatory syndrome in children and the comorbidity scores in pediatric coronavirus disease 2019 cases. Pediatr Int. 2022;64:e15084. 10.1111/ped.15084 34863003

[83] Erturk A Demir S Oztorun Cİ Erten EE Guney D Bostanci SA Management of a Pediatric Burn Center During the Covid-19 Pandemic. J Burn Care Res. 2022;43:468–73. 10.1093/jbcr/irab137 34313735 PMC8344618

[84] Ferraro D Arias AP Pérez G Gómez S Deschutter V Highton E [Epidemiological characteristics according to the progress of SARS-CoV-2 pandemic in a highly complex pediatric hospital in Argentina: a descriptive study]. Rev Chilena Infectol. 2021;38:506–11. Spanish. 10.4067/S0716-10182021000400506 34652396

[85] Forster J Streng A Rudolph P Rücker V Wallstabe J Timme S Feasibility of SARS-CoV-2 Surveillance Testing Among Children and Childcare Workers at German Day Care Centers: A Nonrandomized Controlled Trial. JAMA Netw Open. 2022;5:e2142057. 10.1001/jamanetworkopen.2021.42057 34982157 PMC8728621

[86] Foster CE Marquez L Davis AL Tocco E Koy TH Dunn J A Surge in Pediatric Coronavirus Disease 2019 Cases: The Experience of Texas Children’s Hospital From March to June 2020. J Pediatric Infect Dis Soc. 2021;10:593–8. 10.1093/jpids/piaa164 33301595 PMC7798952

[87] Funk AL Florin TA Kuppermann N Tancredi DJ Xie J Kim K Outcomes of SARS-CoV-2–Positive Youths Tested in Emergency Departments: The Global PERN–COVID-19 Study. JAMA Netw Open. 2022;5:e2142322. 10.1001/jamanetworkopen.2021.42322 35015063 PMC8753506

[88] Gaborieau L Delestrain C Bensaid P Vizeneux A Blanc P Garraffo A Epidemiology and Clinical Presentation of Children Hospitalized with SARS-CoV-2 Infection in Suburbs of Paris. J Clin Med. 2020;9:2227. 10.3390/jcm9072227 32674306 PMC7408757

[89] Galli C Pellegrinelli L Bubba L Primache V Anselmi G Delbue S When the COVID-19 Pandemic Surges during Influenza Season: Lessons Learnt from the Sentinel Laboratory-Based Surveillance of Influenza-Like Illness in Lombardy during the 2019–2020 Season. Viruses. 2021;13:695. 10.3390/v13040695 33923819 PMC8073979

[90] Gampel B Troullioud Lucas AG Broglie L Gartrell-Corrado RD Lee MT Levine J COVID-19 disease in New York City pediatric hematology and oncology patients. Pediatr Blood Cancer. 2020;67:e28420. 10.1002/pbc.28420 32588957 PMC7361160

[91] Garazzino S Montagnani C Donà D Meini A Felici E Vergine G Multicentre Italian study of SARS-CoV-2 infection in children and adolescents, preliminary data as at 10 April 2020. Euro Surveill. 2020;25:2000600. 10.2807/1560-7917.ES.2020.25.18.2000600 32400362 PMC7219028

[92] Gavriliu L-C Murariu C Potop V Spătaru R Characteristics of the pediatric patients diagnosed with SARS-CoV-2 infection in a Romanian children’s hospital: a retrospective study. PeerJ. 2021;9:e11560. 10.7717/peerj.11560 34141491 PMC8183429

[93] Ghosh UK Sultana A Ghosh NK Akram A Ahmed E Rana IH Clinico-demographic profile of Coronavirus infection among Bangladeshi children: A tertiary care hospital study. Bangladesh Journal of Infectious Diseases. 2020;7:S16–S21. 10.3329/bjid.v7i00.50157

[94] Göktuğ A Güngör A Öz FN Akelma Z Güneylioğlu MM Yaradılmış RM Evaluation of Epidemiological, Demographic, Clinical Characteristics and Laboratory Findings of COVID-19 in the Pediatric Emergency Department. J Trop Pediatr. 2021;67:fmab066. 10.1093/tropej/fmab066 34471922 PMC8499923

[95] Gomes NTN Haslett MIC Percio J Duarte MMS Malta JMAS Carvalho FC Retrospective cohort of children and adolescents hospitalized by COVID-19 in Brazil from the beginning of the pandemic to August 1st, 2020. Rev Bras Epidemiol. 2021;24:e210026. 10.1590/1980-549720200026 34378752

[96] Götzinger F Santiago-García B Noguera-Julián A Lanaspa M Lancella L Carducci FIC COVID-19 in children and adolescents in Europe: a multinational, multicentre cohort study. Lancet Child Adolesc Health. 2020;4:653–61. 10.1016/S2352-4642(20)30177-2 32593339 PMC7316447

[97] Gudbjartsson DF Helgason A Jonsson H Magnusson OT Melsted P Norddahl GL Spread of SARS-CoV-2 in the Icelandic population. N Engl J Med. 2020;382:2302–15. 10.1056/NEJMoa2006100 32289214 PMC7175425

[98] Gujar N Tambe M Parande M Salunke N Jagdale G Anderson SG A case control study to assess effectiveness of measles containing vaccines in preventing severe acute respiratory syndrome coronavirus 2 (SARS-CoV-2) infection in children. Hum Vaccin Immunother. 2021;17:3316–21. 10.1080/21645515.2021.1930471 34128766 PMC8220437

[99] Gumus H Ozcan Y Kazanasmaz H Demir A Guzelcicek A. Clinical Characteristics of COVID-19 Infection in the Pediatric Age Group. Electron J Gen Med. 2021;18:em308. 10.29333/ejgm/11019

[100] Guzman BV Elbel B Jay M Messito MJ Curado S Age-dependent association of obesity with COVID-19 severity in paediatric patients. Pediatr Obes. 2022;17:e12856. 10.1111/ijpo.12856 34581027 PMC8646488

[101] Hedberg P Karlsson Valik J van der Werff S Tanushi H Requena Mendez A Granath F Clinical phenotypes and outcomes of SARS-CoV-2, influenza, RSV and seven other respiratory viruses: a retrospective study using complete hospital data. Thorax. 2022;77:154. 10.1136/thoraxjnl-2021-216949 34226206 PMC8260304

[102] Hendler JV do Lago PM Müller GC Santana JC Piva JP Daudt LE Risk factors for severe COVID-19 infection in Brazilian children. Braz J Infect Dis. 2021;25:101650. 10.1016/j.bjid.2021.101650 34774486 PMC8578000

[103] Hernández-Garduño E Comorbidities that predict acute respiratory syndrome coronavirus 2 test positivity in Mexican Children: A case-control study. Pediatr Obes. 2021;16:e12740. 10.1111/ijpo.12740 33040467

[104] Hijazi LO Alaraifi AK Alsaab F Otolaryngology manifestations of COVID-19 in pediatric patients. Int J Pediatr Otorhinolaryngol. 2021;144:110701. 10.1016/j.ijporl.2021.110701 33845420 PMC8011029

[105] Hobbs CV Woodworth K Young CC Jackson AM Newhams MM Dapul H Frequency, Characteristics and Complications of COVID-19 in Hospitalized Infants. Pediatr Infect Dis J. 2022;41:e81–6. 10.1097/INF.0000000000003435 34955519 PMC8828316

[106] Howard LM Garguilo K Gillon J LeBlanc K Seegmiller AC Schmitz JE The first 1000 symptomatic pediatric SARS-CoV-2 infections in an integrated health care system: a prospective cohort study. BMC Pediatr. 2021;21:403. 10.1186/s12887-021-02863-1 34517879 PMC8435399

[107] Huete-Pérez JA Ernst KC Cabezas-Robelo C Páiz-Medina L Silva S Huete A Prevalence and risk factors for SARS-CoV-2 infection in children with and without symptoms seeking care in Managua, Nicaragua: results of a cross-sectional survey. BMJ Open. 2021;11:e051836. 10.1136/bmjopen-2021-051836 34548362 PMC8457995

[108] Ibrahim LF Tham D Chong V Corden M Craig S Buntine P The characteristics of SARS-CoV-2-positive children who presented to Australian hospitals during 2020: a PREDICT network study. Med J Aust. 2021;215:217–21. 10.5694/mja2.51207 34389995 PMC8447363

[109] Ibrahim LF Tosif S McNab S Hall S Lee HJ Lewena S SARS-CoV-2 testing and outcomes in the first 30 days after the first case of COVID-19 at an Australian children’s hospital. Emerg Med Australas. 2020;32:801–8. 10.1111/1742-6723.13550 32390285 PMC7273066

[110] Imamura T Saito M Ko YK Imamura T Otani K Akaba H Roles of children and adolescents in COVID-19 transmission in the community: a retrospective analysis of nationwide data in Japan. Front Pediatr. 2021;9:705882. 10.3389/fped.2021.705882 34447727 PMC8382948

[111] Indriyani SAK Dewi NE Kartasasmita CB Characteristics and Outcomes of Children With COVID-19: Evidence From West Nusa Tenggara Province, Indonesia. Archives of Pediatric Infectionus Diseases. 2021;9:e111762. 10.5812/pedinfect.111762

[112] Isoldi S Mallardo S Marcellino A Bloise S Dilillo A Iorfida D The comprehensive clinic, laboratory, and instrumental evaluation of children with COVID-19: A 6-months prospective study. J Med Virol. 2021;93:3122–32. 10.1002/jmv.26871 33570199 PMC8014060

[113] Jang J Hwang MJ Kim YY Park SY Yoo M Kim SS Epidemiological Characteristics and Transmission Patterns of COVID-19 Cases Among Children and Adolescents Aged 0-18 Years in South Korea. Risk Manag Healthc Policy. 2022;15:219–27. 10.2147/RMHP.S338121 35173498 PMC8841663

[114] Ji SQ Zhang M Zhang Y Xia K Chen Y Chu Q Characteristics of immune and inflammatory responses among different age groups of pediatric patients with COVID-19 in China. World J Pediatr. 2021;17:375–84. 10.1007/s12519-021-00440-1 34341947 PMC8328122

[115] Jimenez-García R Nogueira J Retuerta-Oliva A Sainz T Cano-Fernández J Flores-Pérez P Pneumonia in Hospitalized Children During SARS-CoV-2 Pandemic. Is it All COVID-19? Comparison Between COVID and Non-COVID Pneumonia. Pediatr Infect Dis J. 2021;40:e111–3. 10.1097/INF.0000000000003008 33264212

[116] Özge K Yanartaş MS Törün SH Alakbarova K Bayramoğlu Z Mustafa Ö Experience in children in the COVID-19 pandemic of a tertiary center, in Istanbul. Journal of Istanbul Faculty of Medicine. 2021;84:293–300.

[117] Kanthimathinathan HK Dhesi A Hartshorn S Ali SH Kirk J Nagakumar P COVID-19: a UK children’s hospital experience. Hosp Pediatr. 2020;10:802–5. 10.1542/hpeds.2020-000208 32518091

[118] Kapoor D Kumar V Pemde H Singh P Impact of Comorbidities on Outcome in Children With COVID-19 at a Tertiary Care Pediatric Hospital. Indian Pediatr. 2021;58:572–5. 10.1007/s13312-021-2244-0 34176797 PMC8253676

[119] Kara AA Böncüoğlu E Kıymet E Arıkan KÖ Şahinkaya Ş Düzgöl M Evaluation of predictors of severe-moderate COVID-19 infections at children: A review of 292 children. J Med Virol. 2021;93:6634–40. 10.1002/jmv.27237 34314067 PMC8426728

[120] Karaaslan A Çetin C Akın Y Tekol SD Söbü E Demirhan R Coinfection in SARS-CoV-2 infected children patients. J Infect Dev Ctries. 2021;15:761–5. 10.3855/jidc.14314 34242183

[121] Karaci M Güven Ş Boğa A Varol F Çalışkan S Sayman EN Should We Perform Laboratory and Radiographic Evaluations for All Children with COVID-19?: A Single-Center Experience. Journal of Child Science. 2021;11:e93–9. 10.1055/s-0041-1729630

[122] Karbuz A Akkoc G Bedir Demirdag T Yilmaz Ciftdogan D Ozer A Cakir D Epidemiological, Clinical, and Laboratory Features of Children With COVID-19 in Turkey. Front Pediatr. 2021;9:631547. 10.3389/fped.2021.631547 34055680 PMC8161543

[123] Kavanagh FG James DL Brinkman D Cornyn S Murphy C O’Neill S Safety of elective paediatric surgery during the coronavirus disease 2019 pandemic. Int J Pediatr Otorhinolaryngol. 2021;150:110861. 10.1016/j.ijporl.2021.110861 34583300 PMC8349430

[124] Kepenekli E Yakut N Ergenc Z Aydıner Ö Sarınoğlu RC Karahasan A COVID-19 disease characteristics in different pediatric age groups. J Infect Dev Ctries. 2022;16:16–24. 10.3855/jidc.15353 35192517

[125] Kim L Whitaker M O’Halloran A Kambhampati A Chai SJ Reingold A Hospitalization rates and characteristics of children aged< 18 years hospitalized with laboratory-confirmed COVID-19—COVID-NET, 14 states, March 1–July 25, 2020. MMWR Morb Mortal Wkly Rep. 2020;69:1081. 10.15585/mmwr.mm6932e3 32790664 PMC7440125

[126] Krithika A Vindhiya K Prithviraj S Manasvini S An observational study of clinical presentation of covid 19 among children in India. Current Pediatric Research. 2021;25:674–6.

[127] Kuchar E Załęski A Wronowski M Krankowska D Podsiadły E Brodaczewska K Children were less frequently infected with SARS-CoV-2 than adults during 2020 COVID-19 pandemic in Warsaw, Poland. Eur J Clin Microbiol Infect Dis. 2021;40:541–7. 10.1007/s10096-020-04038-9 32986153 PMC7520378

[128] Kuczborska K Książyk J Prevalence and Course of SARS-CoV-2 Infection among Immunocompromised Children Hospitalised in the Tertiary Referral Hospital in Poland. J Clin Med. 2021;10:4556. 10.3390/jcm10194556 34640570 PMC8509507

[129] Kufa T Jassat W Cohen C Tempia S Masha M Wolter N Epidemiology of SARS-CoV-2 infection and SARS-CoV-2 positive hospital admissions among children in South Africa. Influenza Other Respir Viruses. 2022;16:34–47. 10.1111/irv.12916 34796674 PMC9664941

[130] More K Chawla D Murki S Tandur B Deorari AK Kumar P Outcomes of neonates born to mothers with coronavirus disease 2019 (COVID-19)—National Neonatology Forum (NNF) India COVID-19 Registry. Indian Pediatr. 2021;58:525–31. 10.1007/s13312-021-2234-2 33742609 PMC8253678

[131] Kushner LE Schroeder AR Kim J Mathew R “For COVID” or “With COVID”: Classification of SARS-CoV-2 Hospitalizations in Children. Hosp Pediatr. 2021;11:e151–6. 10.1542/hpeds.2021-006001 34011566

[132] Ladhani SN Ireland G Baawuah F Beckmann J Okike IO Ahmad S SARS-CoV-2 infection, antibody positivity and seroconversion rates in staff and students following full reopening of secondary schools in England: A prospective cohort study, September–December 2020. EClinicalMedicine. 2021;37:100948. 10.1016/j.eclinm.2021.100948 34386740 PMC8343251

[133] Lanari M Biserni GB Pavoni M Borgatti EC Leone M Corsini I Feasibility and Effectiveness Assessment of SARS-CoV-2 Antigenic Tests in Mass Screening of a Pediatric Population and Correlation with the Kinetics of Viral Loads. Viruses. 2021;13:2071. 10.3390/v13102071 34696501 PMC8537025

[134] Lazzerini M Sforzi I Trapani S Biban P Silvagni D Villa G Characteristics and risk factors for SARS-CoV-2 in children tested in the early phase of the pandemic: a cross-sectional study, Italy, 23 February to 24 May 2020. Euro Surveill. 2021;26:2001248. 10.2807/1560-7917.ES.2021.26.14.2001248 33834960 PMC8034058

[135] Lee H Choi S Park JY Jo DS Choi UY Lee H Analysis of Critical COVID-19 Cases Among Children in Korea. J Korean Med Sci. 2022;37:e13. 10.3346/jkms.2022.37.e13 34981683 PMC8723896

[136] Leidman E Duca LM Omura JD Proia K Stephens JW Sauber-Schatz EK COVID-19 trends among persons aged 0–24 years—United States, March 1–December 12, 2020. MMWR Morb Mortal Wkly Rep. 2021;70:88. 10.15585/mmwr.mm7003e1 33476314 PMC7821770

[137] Levy C Basmaci R Bensaid P Bru CB Coinde E Dessioux E Changes in Reverse Transcription Polymerase Chain Reaction–positive Severe Acute Respiratory Syndrome Coronavirus 2 Rates in Adults and Children According to the Epidemic Stages. Pediatr Infect Dis J. 2020;39:e369–72. 10.1097/INF.0000000000002861 32868745

[138] Lindsay L Secrest MH Rizzo S Keebler DS Yang F Tsai L Factors associated with COVID-19 viral and antibody test positivity and assessment of test concordance: a retrospective cohort study using electronic health records from the USA. BMJ Open. 2021;11:e051707. 10.1136/bmjopen-2021-051707 34598988 PMC8488284

[139] Liu T Liang W Zhong H He J Chen Z He G Risk factors associated with COVID-19 infection: a retrospective cohort study based on contacts tracing. Emerg Microbes Infect. 2020;9:1546–53. 10.1080/22221751.2020.1787799 32608325 PMC7473290

[140] Liu W Zhang Q Chen J Xiang R Song H Shu S Detection of Covid-19 in children in early January 2020 in Wuhan, China. N Engl J Med. 2020;382:1370–1. 10.1056/NEJMc2003717 32163697 PMC7121643

[141] Loconsole D Caselli D Centrone F Morcavallo C Campanella S Aricò M SARS-CoV-2 Infection in Children in Southern Italy: A Descriptive Case Series. Int J Environ Res Public Health. 2020;17:6080. 10.3390/ijerph17176080 32825563 PMC7504571

[142] López-Aguilar E Cárdenas-Navarrete R Simental-Toba A Pacheco-Rosas D Thomé-Ortiz P Soto-Pérez G Children with cancer during COVID-19 pandemic: Early experience in Mexico. Pediatr Blood Cancer. 2021;68:e28660. 10.1002/pbc.28660 32902133

[143] Lorenzo VB Nascimento-Carvalho CM Differences between children with severe acute lower respiratory infection with or without SARS-Cov-2 infection. J Infect. 2021;83:e1–3. 10.1016/j.jinf.2021.05.038 34090916 PMC8197553

[144] Lu X Zhang L Du H Zhang J Li YY Qu J SARS-CoV-2 infection in children. N Engl J Med. 2020;382:1663–5. 10.1056/NEJMc2005073 32187458 PMC7121177

[145] Lu Y Li Y Deng W Liu M He Y Huang L Symptomatic Infection is Associated with Prolonged Duration of Viral Shedding in Mild Coronavirus Disease 2019: A Retrospective Study of 110 Children in Wuhan. Pediatr Infect Dis J. 2020;39:e95–9. 10.1097/INF.0000000000002729 32379191 PMC7279058

[146] Lynam K Twomey J Mahony M O'Mahony E Ahmed I Murphy A Low prevalence of SARS-CoV-2 detected in symptomatic children admitted to hospital. Irish Medical Journal. 2021;114:1.

[147] Madani S Shahin S Yoosefi M Ahmadi N Ghasemi E Koolaji S Red flags of poor prognosis in pediatric cases of COVID-19: the first 6610 hospitalized children in Iran. BMC Pediatr. 2021;21:563. 10.1186/s12887-021-03030-2 34893036 PMC8660655

[148] Maltezou HC Magaziotou I Dedoukou X Eleftheriou E Raftopoulos V Michos A Children and Adolescents With SARS-CoV-2 Infection: Epidemiology, Clinical Course and Viral Loads. Pediatr Infect Dis J. 2020;39:e388–92. 10.1097/INF.0000000000002899 33031141

[149] Mania A Mazur-Melewska K Lubarski K Kuczma-Napierała J Mazurek J Jończyk-Potoczna K Wide spectrum of clinical picture of COVID-19 in children—From mild to severe disease. J Infect Public Health. 2021;14:374–9. 10.1016/j.jiph.2020.12.029 33621800 PMC7833883

[150] Mania A Pokorska-Śpiewak M Figlerowicz M Pawłowska M Mazur-Melewska K Faltin K Pneumonia, gastrointestinal symptoms, comorbidities, and coinfections as factors related to a lengthier hospital stay in children with COVID-19—analysis of a paediatric part of Polish register SARSTer. Infect Dis (Lond). 2022;54:196–204. 10.1080/23744235.2021.1995628 34711132 PMC8567279

[151] Matteudi T Luciani L Fabre A Minodier P Boucekine M Bosdure E Clinical characteristics of paediatric COVID-19 patients followed for up to 13 months. Acta Paediatr. 2021;110:3331–3. 10.1111/apa.16071 34403523 PMC8444774

[152] Melé M Henares D Pino R Asenjo S Matamoros R Fumadó V Low impact of SARS-CoV-2 infection among paediatric acute respiratory disease hospitalizations. J Infect. 2021;82:414–51. 10.1016/j.jinf.2020.10.013 33098956 PMC7577222

[153] Messiah SE Xie L Mathew MS Delclos GL Kohl HW Kahn JS Results of COVID-19 Surveillance in a Large United States Pediatric Healthcare System over One Year. Children (Basel). 2021;8:752. 10.3390/children8090752 34572184 PMC8468442

[154] Meyer M Ruebsteck E Eifinger F Klein F Oberthuer A van Koningsbruggen-Rietschel S Morbidity of Respiratory Syncytial Virus (RSV) Infections: RSV Compared With Severe Acute Respiratory Syndrome Coronavirus 2 Infections in Children Aged 0-4 Years in Cologne, Germany. J Infect Dis. 2022;226:2050–3. 10.1093/infdis/jiac052 35172330 PMC8903412

[155] Meyer M Ruebsteck E Gruell H Klein F Lehmann C Wendt S COVID-19 study found that 0.4% of 5730 asymptomatic children aged 0–18 years tested positive for virus before hospital procedures or admission. Acta Paediatr. 2021;110:2584–5. 10.1111/apa.15884 33894011 PMC8251235

[156] Michos A Savvidou P Syridou G Eleftheriou E Iosifidis E Grivea I SARS-CoV-2 molecular testing in Greek hospital paediatric departments: a nationwide study. Epidemiol Infect. 2021;149:e70. 10.1017/S0950268821000455 33622430 PMC7985887

[157] Morban DAH Hidalgo MEC Jorge MM Antonio EP Clinical and epidemiological characteristics of COVID-19 in Pediatrics in Dominican Republic. Rev Cubana Pediatr. 2021;93:1–19.

[158] Moreno-Noguez M Rivas-Ruiz R Roy-García IA Pacheco-Rosas DO Moreno-Espinosa S Flores-Pulido AA Risk factors associated with SARS-CoV-2 pneumonia in the pediatric population. Bol Med Hosp Infant Mex. 2021;78:251–8. 10.24875/BMHIM.20000263 34351892

[159] Murillo-Zamora E Aguilar-Sollano F Delgado-Enciso I Hernandez-Suarez CM Predictors of laboratory-positive COVID-19 in children and teenagers. Public Health. 2020;189:153–7. 10.1016/j.puhe.2020.10.012 33246302 PMC7584439

[160] Musa OAH Chivese T Bansal D Abdulmajeed J Ameen O Islam N Prevalence and determinants of symptomatic COVID-19 infection among children and adolescents in Qatar: a cross-sectional analysis of 11 445 individuals. Epidemiol Infect. 2021;149:e193. 10.1017/S0950268821001515 34210371 PMC8387684

[161] Mveang Nzoghe A Padzys GS Maloupazoa Siawaya AC Kandet Yattara M Leboueny M Avome Houechenou RM Dynamic and features of SARS-CoV-2 infection in Gabon. Sci Rep. 2021;11:9672. 10.1038/s41598-021-87043-y 33958601 PMC8102484

[162] Navarro-Olivos E Padilla-Raygoza N Flores-Vargas G Gallardo-Luna MJ León-Verdín MG Lara-Lona E COVID-19-Associated Case Fatality Rate in Subjects Under 18 Years Old in Mexico, up to December 31, 2020. Front Pediatr. 2021;9:696425. 10.3389/fped.2021.696425 34660475 PMC8517252

[163] Ng DC Tan KK Chin L Ali MM Lee ML Mahmood FM Clinical and epidemiological characteristics of children with COVID-19 in Negeri Sembilan, Malaysia. Int J Infect Dis. 2021;108:347–52. 10.1016/j.ijid.2021.05.073 34087485 PMC8168297

[164] Odeleye E Friar S Bate J Successful Implementation of Routine SARS-CoV-2 Screening in Children With Cancer and Their Parents During the Pandemic in the United Kingdom. J Pediatr Hematol Oncol. 2021;43:e1046–7. 10.1097/MPH.0000000000002145 33769389

[165] Okur DS Clinical impact of COVID-19 on Turkish children with neurological and neuromuscular diseases: One center experience. Medicine (Baltimore). 2021;100:e28401. 10.1097/MD.0000000000028401 34941179 PMC8702014

[166] Olivar-López V Leyva-Barrera A López-Martínez B Parra-Ortega I Márquez-González H Clinical risk profile associated with SARS-CoV-2 infection and complications in the emergency area of a pediatric COVID-19 center. Bol Med Hosp Infant Mex. 2020;77:221–7. 10.24875/BMHIM.20000198 33064676

[167] Oliveira EA Colosimo EA Simões e Silva AC Mak RH Martelli DB Silva LR Clinical characteristics and risk factors for death among hospitalised children and adolescents with COVID-19 in Brazil: an analysis of a nationwide database. Lancet Child Adolesc Health. 2021;5:559–68. 10.1016/S2352-4642(21)00134-6 34119027 PMC8192298

[168] Ollier Q Pillet S Mory O Gagnaire J Thuiller C Annino N Prospective evaluation of the point-of-care use of a rapid antigenic SARS-CoV-2 immunochromatographic test in a paediatric emergency department. Clin Microbiol Infect. 2022;28:734.e1–6. 10.1016/j.cmi.2021.12.019 35065265 PMC8769904

[169] Olson SM Newhams MM Halasa NB Price AM Boom JA Sahni LC Effectiveness of BNT162b2 vaccine against critical Covid-19 in adolescents. N Engl J Med. 2022;386:713–23. 10.1056/NEJMoa2117995 35021004 PMC8781318

[170] Omrani AS Almaslamani MA Daghfal J Alattar RA Elgara M Shaar SH The first consecutive 5000 patients with Coronavirus Disease 2019 from Qatar; a nation-wide cohort study. BMC Infect Dis. 2020;20:777. 10.1186/s12879-020-05511-8 33076848 PMC7570422

[171] Ortiz-Pinto MA de Miguel-García S Ortiz-Marrón H Ortega-Torres A Cabañas G Gutiérrez–Torres LF Childhood obesity and risk of SARS-CoV-2 infection. Int J Obes (Lond). 2022;46:1155–9. 10.1038/s41366-022-01094-3 35173279 PMC8853122

[172] Osmanov IM Spiridonova E Bobkova P Gamirova A Shikhaleva A Andreeva M Risk factors for post-COVID-19 condition in previously hospitalised children using the ISARIC Global follow-up protocol: a prospective cohort study. Eur Respir J. 2022;59:2101341. 10.1183/13993003.01341-2021 34210789 PMC8576804

[173] Otto WR Geoghegan S Posch LC Bell LM Coffin SE Sammons JS The Epidemiology of Severe Acute Respiratory Syndrome Coronavirus 2 in a Pediatric Healthcare Network in the United States. J Pediatric Infect Dis Soc. 2020;9:523–9. 10.1093/jpids/piaa074 32559282 PMC7337783

[174] Özlü SG Aydın Z Bozelli BN Avcı B İnözü M Çaycı FŞ Can microalbuminuria be an ındicator of renal ınvolvement in pediatric Covid 19 patients? Infection. 2022;50:719–24. 10.1007/s15010-021-01745-z 35094314 PMC8800829

[175] Paduano S Facchini MC Greco A Borsari L Mingrone VM Tancredi S Characteristics and risk factors of isolated and quarantined children and adolescents during the first wave of SARS-CoV-2 pandemic: A cross-sectional study in Modena, Northern Italy: SARS-CoV-2 in Modena children. Acta Biomed. 2021;92:e2021449. 34739471 10.23750/abm.v92iS6.12225 PMC8850999

[176] Pande N Save S Kondekar A Sawant V Rathi S Malik S Clinical profile of children with sars-cov-2 infection from a dedicated covid-19 hospital in India. Current Pediatric Research. 2021;25:697–703.

[177] Pandey M Sisodia S Bandi S Roland D Incidence of spread of clinically relevant SARS-CoV2 infection between children in a tertiary emergency department: An evaluation. J Infect. 2020;81:979–97. 10.1016/j.jinf.2020.09.020 32961252 PMC7834124

[178] Parambil BC Moulik NR Dhamne C Dhariwal N Narula G Vora T COVID-19 in Children with Cancer and Continuation of Cancer-Directed Therapy During the Infection. Indian J Pediatr. 2022;89:445–51. 10.1007/s12098-021-03894-3 34378149 PMC8354680

[179] Parcha V Booker KS Kalra R Kuranz S Berra L Arora G A retrospective cohort study of 12,306 pediatric COVID-19 patients in the United States. Sci Rep. 2021;11:10231. 10.1038/s41598-021-89553-1 33986390 PMC8119690

[180] Parri N Lenge M Cantoni B Arrighini A Romanengo M Urbino A COVID-19 in 17 Italian Pediatric Emergency Departments. Pediatrics. 2020;146:e20201235. 10.1542/peds.2020-1235 32968031

[181] Peaper DR Murdzek C Oliveira CR Murray TS Severe Acute Respiratory Syndrome Coronavirus 2 Testing in Children in a Large Regional US Health System During the Coronavirus Disease 2019 Pandemic. Pediatr Infect Dis J. 2021;40:175–81. 10.1097/INF.0000000000003024 33399431 PMC8852689

[182] Peng X Guo Y Xiao H Xia W Zhai A Zhu B Overview of chest involvement at computed tomography in children with coronavirus disease 2019 (COVID-19). Pediatr Radiol. 2021;51:222–30. 10.1007/s00247-020-04826-7 33084963 PMC7576110

[183] Perramon A Soriano-Arandes A Pino D Lazcano U Andrés C Català M Schools as a Framework for COVID-19 Epidemiological Surveillance of Children in Catalonia, Spain: A Population-Based Study. Front Pediatr. 2021;9:754744. 10.3389/fped.2021.754744 34568244 PMC8457047

[184] Pokorska-Śpiewak M Talarek E Mania A Pawłowska M Popielska J Zawadka K Clinical and Epidemiological Characteristics of 1283 Pediatric Patients with Coronavirus Disease 2019 during the First and Second Waves of the Pandemic—Results of the Pediatric Part of a Multicenter Polish Register SARSTer. J Clin Med. 2021;10:5098. 10.3390/jcm10215098 34768620 PMC8585006

[185] Pokorska-Śpiewak M Talarek E Popielska J Nowicka K Ołdakowska A Zawadka K Comparison of clinical severity and epidemiological spectrum between coronavirus disease 2019 and influenza in children. Sci Rep. 2021;11:5760. 10.1038/s41598-021-85340-0 33707568 PMC7952543

[186] Pudjiadi AH Putri ND Sjakti HA Yanuarso PB Gunardi H Roeslani RD Pediatric COVID-19: Report From Indonesian Pediatric Society Data Registry. Front Pediatr. 2021;9:716898. 10.3389/fped.2021.716898 34631619 PMC8495320

[187] Qian G Zhang Y Xu Y Hu W Hall IP Yue J Reduced inflammatory responses to SARS-CoV-2 infection in children presenting to hospital with COVID-19 in China. EClinicalMedicine. 2021;34:100831. 10.1016/j.eclinm.2021.100831 33880437 PMC8049192

[188] Rabha AC Fernandes FR Solé D Bacharier LB Wandalsen GF Asthma is associated with lower respiratory tract involvement and worse clinical score in children with COVID-19. Pediatr Allergy Immunol. 2021;32:1577–80. 10.1111/pai.13536 33966294 PMC8236910

[189] Rabha AC Oliveira Junior FI Oliveira TA Cesar RG Fongaro G Mariano RF Clinical manifestations of children and adolescents with COVID-19: report of the first 115 cases from Sabará Hospital Infantil. Rev Paul Pediatr. 2020;39:e2020305. 10.1590/1984-0462/2021/39/2020305 33263697 PMC7695045

[190] Reis BY Barda N Leshchinsky M Kepten E Hernán MA Lipsitch M Effectiveness of BNT162b2 vaccine against delta variant in adolescents. N Engl J Med. 2021;385:2101–3. 10.1056/NEJMc2114290 34670036 PMC8552532

[191] Rha B Lively JY Englund JA Staat MA Weinberg GA Selvarangan R Severe Acute Respiratory Syndrome Coronavirus 2 Infections in Children: Multicenter Surveillance, United States, January–March 2020. J Pediatric Infect Dis Soc. 2020;9:609–12. 10.1093/jpids/piaa075 32556327 PMC7337823

[192] Rivas-Ruiz R Roy-García IA Ureña-Wong KR Aguilar-Ituarte F Vázquez-de Anda GF Gutiérrez-Castrellón P Factors associated with death in children with COVID-19 in Mexico. Gac Med Mex. 2020;156:516–22. 33877105 10.24875/GMM.M21000478

[193] Rizzo C Loconsole D Pandolfi E Ciofi Degli Atti ML Van Summeren J Paget J Sars-Cov2 not detected in a pediatric population with acute respiratory infection in primary care in Central and Southern Italy from November 2019 to Early March 2020. Front Pediatr. 2021;9:620598. 10.3389/fped.2021.620598 34046372 PMC8147864

[194] Rodriguez Velásquez S Jacques L Dalal J Sestito P Habibi Z Venkatasubramanian A The toll of COVID-19 on African children: a descriptive analysis on COVID-19-related morbidity and mortality among the pediatric population in sub-Saharan Africa. Int J Infect Dis. 2021;110:457–65. 10.1016/j.ijid.2021.07.060 34332088 PMC8457828

[195] De Rose DU Auriti C Landolfo F Capolupo I Salvatori G Ranno S Reshaping neonatal intensive care units (NICUs) to avoid the spread of severe acute respiratory coronavirus virus 2 (SARS-CoV-2) to high-risk infants. Infect Control Hosp Epidemiol. 2021;42:632–3. 10.1017/ice.2020.310 32576333 PMC7360948

[196] Sahni LC Avadhanula V Ortiz CS Feliz KE John RE Brown CA Comparison of Mid-Turbinate and Nasopharyngeal Specimens for Molecular Detection of SARS-CoV-2 Among Symptomatic Outpatients at a Pediatric Drive-Through Testing Site. J Pediatric Infect Dis Soc. 2021;10:872–9. 10.1093/jpids/piab046 34173660

[197] Salako A Odubela O Musari-Martins T Ezemelue P Gbaja-Biamila T Opaneye B Prevalence and Presentation of Paediatric Coronavirus Disease 2019 in Lagos, Nigeria. Int J Pediatr. 2021;2021:2185161. 10.1155/2021/2185161 34659422 PMC8514970

[198] Saleh NY Aboelghar HM Salem SS Ibrahem RA Khalil FO Abdelgawad AS The severity and atypical presentations of COVID-19 infection in pediatrics. BMC Pediatr. 2021;21:144. 10.1186/s12887-021-02614-2 33765980 PMC7992820

[199] Sananez I Raiden SC Algieri SC Uranga M Grisolía NA Filippo D A poor and delayed anti-SARS-CoV2 IgG response is associated to severe COVID-19 in children. EBioMedicine. 2021;72:103615. 10.1016/j.ebiom.2021.103615 34649078 PMC8502533

[200] Schneider JG Relich RF Datta D Bond C Goings M Hall D Identifying Risk Factors That Distinguish Symptomatic Severe Acute Respiratory Syndrome Coronavirus 2 Infection From Common Upper Respiratory Infections in Children. Cureus. 2021;13:e13266. 10.7759/cureus.13266 33728202 PMC7948314

[201] Sedighi I Fahimzad A Pak N Khalili M Shokrollahi MR Heydari H A multicenter retrospective study of clinical features, laboratory characteristics, and outcomes of 166 hospitalized children with coronavirus disease 2019 (COVID-19): A preliminary report from Iranian Network for Research in Viral Diseases (INRVD). Pediatr Pulmonol. 2022;57:498–507. 10.1002/ppul.25756 34779156 PMC8661970

[202] See KC Tan LP Ong LT Lee PY Clinical and epidemiological characteristics of children with COVID-19 in Selangor, Malaysia. IJID Reg. 2022;2:63–9. 10.1016/j.ijregi.2021.11.012 35721419 PMC8645283

[203] Sena GR Lima TPF Vidal SA Duarte MDCMB Bezerra PGM Fonseca Lima EJ Clinical Characteristics and Mortality Profile of COVID-19 Patients Aged less than 20 years Old in Pernambuco – Brazil. Am J Trop Med Hyg. 2021;104:1507–12. 10.4269/ajtmh.20-1368 33606669 PMC8045659

[204] Shahid S Raza M Junejo S Maqsood S Clinical features and outcome of COVID-19 positive children from a tertiary healthcare hospital in Karachi. J Med Virol. 2021;93:5988–97. 10.1002/jmv.27178 34228363 PMC8427002

[205] Shapiro Ben David S Rahamim-Cohen D Tasher D Geva A Azuri J Ash N COVID-19 in children and the effect of schools reopening on potential transmission to household members. Acta Paediatr. 2021;110:2567–73. 10.1111/apa.15962 34053108 PMC8222890

[206] Sharma AG Kumar V Sodani R Sapre A Singh P Saha A Predictors of mortality in children admitted with SARS-CoV-2 infection to a tertiary care hospital in North India. J Paediatr Child Health. 2022;58:432–9. 10.1111/jpc.15737 34546612 PMC8661990

[207] Sharma AK Chapagain RH Bista KP Bohara R Chand B Chaudhary NK Epidemiological and clinical profile of COVID-19 in Nepali children: An initial experience. J Nepal Paediatr Soc. 2020;40:202–9. 10.3126/jnps.v40i3.32438

[208] Shi T Pan J Katikireddi SV McCowan C Kerr S Agrawal U Risk of COVID-19 hospital admission among children aged 5–17 years with asthma in Scotland: a national incident cohort study. Lancet Respir Med. 2022;10:191–8. 10.1016/S2213-2600(21)00491-4 34861180 PMC8631918

[209] Shoji K Akiyama T Tsuzuki S Matsunaga N Asai Y Suzuki S Clinical Characteristics of Hospitalized COVID-19 in Children: Report From the COVID-19 Registry in Japan. J Pediatric Infect Dis Soc. 2021;10:1097–100. 10.1093/jpids/piab085 34487185 PMC8522383

[210] Shoji K Akiyama T Tsuzuki S Matsunaga N Asai Y Suzuki S Comparison of the clinical characteristics and outcomes of COVID-19 in children before and after the emergence of Delta variant of concern in Japan. J Infect Chemother. 2022;28:591–4. 10.1016/j.jiac.2022.01.009 35074258 PMC8769918

[211] Singh P Attri K Mahto D Kumar V Kapoor D Seth A Clinical Profile of COVID-19 Illness in Children—Experience from a Tertiary Care Hospital. Indian J Pediatr. 2022;89:45–51. 10.1007/s12098-021-03822-5 34313946 PMC8313877

[212] Sola AM David AP Rosbe KW Baba A Ramirez-Avila L Chan DK Prevalence of SARS-CoV-2 Infection in Children Without Symptoms of Coronavirus Disease 2019. JAMA Pediatr. 2021;175:198–201. 10.1001/jamapediatrics.2020.4095 32840605 PMC7851725

[213] Somekh I Stein M Karakis I Simões EAF Somekh E Characteristics of SARS-CoV-2 Infections in Israeli Children During the Circulation of Different SARS-CoV-2 Variants. JAMA Netw Open. 2021;4:e2124343. 10.1001/jamanetworkopen.2021.24343 34491353 PMC8424472

[214] Song X Delaney M Shah RK Campos JM Wessel DL DeBiasi RL Common seasonal respiratory viral infections in children before and during the coronavirus disease 2019 (COVID-19) pandemic. Infect Control Hosp Epidemiol. 2022;43:1454–8. 10.1017/ice.2021.430 34607617

[215] Soriano-Arandes A Gatell A Serrano P Biosca M Campillo F Capdevila R Household Severe Acute Respiratory Syndrome Coronavirus 2 Transmission and Children: A Network Prospective Study. Clin Infect Dis. 2021;73:e1261–9. 10.1093/cid/ciab228 33709135 PMC7989526

[216] Sousa BLA Brentani A Costa Ribeiro CC Dolhnikoff M Grisi SJFE Ferrer APS Non-communicable diseases, sociodemographic vulnerability and the risk of mortality in hospitalised children and adolescents with COVID-19 in Brazil: a cross-sectional observational study. BMJ Open. 2021;11:e050724. 10.1136/bmjopen-2021-050724 34489291 PMC8423513

[217] Stokes EK Zambrano LD Anderson KN Marder EP Raz KM El Burai Felix S Coronavirus Disease 2019 Case Surveillance - United States, January 22-May 30, 2020. MMWR Morb Mortal Wkly Rep. 2020;69:759–65. 10.15585/mmwr.mm6924e2 32555134 PMC7302472

[218] Talarico V Roppa K Alcaro T Vinci M Minchella P Raiola G Epidemiological analysis on children and adolescents with COVID-19 in a central area of Calabria region: one year of pandemia. Acta Biomed. 2021;92:e2021381. 34738568 10.23750/abm.v92i5.11769 PMC8689322

[219] Tang F Luo W Wang X Li H Mei H Shao J Clinical features and follow-up of pediatric patients hospitalized for COVID-19. Pediatr Pulmonol. 2021;56:1967–75. 10.1002/ppul.25407 33852775 PMC8250880

[220] Tosif S Ibrahim LF Hughes RR Cheng D Wurzel D Overmars I Characteristics and outcomes of SARS-CoV-2 infection in Victorian children at a tertiary paediatric hospital. J Paediatr Child Health. 2022;58:618–23. 10.1111/jpc.15786 34693586 PMC8662161

[221] Uka A Buettcher M Bernhard-Stirnemann S Fougère Y Moussaoui D Kottanattu L Factors associated with hospital and intensive care admission in paediatric SARS-CoV-2 infection: a prospective nationwide observational cohort study. Eur J Pediatr. 2022;181:1245–55. 10.1007/s00431-021-04276-9 34845526 PMC8628837

[222] Ustundag G Yilmaz-Ciftdogan D Kara-Aksay A Sahin A Ekemen-Keles Y Orsdemir-Hortu H Coronavirus disease 2019 in healthy children: What is the effect of household contact? Pediatr Int. 2022;64:e14890. 10.1111/ped.14890 34145691 PMC8447341

[223] van der Zalm MM Lishman J Verhagen LM Redfern A Smit L Barday M Clinical Experience With Severe Acute Respiratory Syndrome Coronavirus 2–Related Illness in Children: Hospital Experience in Cape Town, South Africa. Clin Infect Dis. 2021;72:e938–44. 10.1093/cid/ciaa1666 33170927 PMC7717210

[224] Verd S Ramakers J Vinuela I Martin-Delgado MI Prohens A Díez R Does breastfeeding protect children from COVID-19? An observational study from pediatric services in Majorca, Spain. Int Breastfeed J. 2021;16:83. 10.1186/s13006-021-00430-z 34663389 PMC8521512

[225] Vergine G Fantini M Marchetti F Stella M Valletta E Biasucci G Home management of children with COVID-19 in the Emilia-Romagna Region, Italy. Front Pediatr. 2020;8:575290. 10.3389/fped.2020.575290 33194906 PMC7644844

[226] Vogel S von Both U Nowak E Ludwig J Köhler A Lee N SARS-CoV-2 Saliva Mass Screening in Primary Schools: A 10-Week Sentinel Surveillance Study in Munich, Germany. Diagnostics (Basel). 2022;12:162. 10.3390/diagnostics12010162 35054329 PMC8774979

[227] Wang SM Tao F Hou Y Zhang A Xiong H Sun JJ Screening of SARS-CoV-2 in 299 Hospitalized Children with Hemato-oncological Diseases: A Multicenter Survey in Hubei, China. Curr Med Sci. 2020;40:642–5. 10.1007/s11596-020-2228-7 32767262 PMC7412776

[228] Wang X Chen X Tang F Luo W Fang J Qi C Be aware of acute kidney injury in critically ill children with COVID-19. Pediatr Nephrol. 2021;36:163–9. 10.1007/s00467-020-04715-z 32844290 PMC7447530

[229] Wanga V Gerdes ME Shi DS Choudhary R Dulski TM Hsu S Characteristics and clinical outcomes of children and adolescents aged< 18 years hospitalized with COVID-19—six hospitals, United States, July–August 2021. MMWR Morb Mortal Wkly Rep. 2021;70:1766. 10.15585/mmwr.mm705152a3 34968374 PMC8736272

[230] Ward JL Harwood R Smith C Kenny S Clark M Davis PJ Risk factors for PICU admission and death among children and young people hospitalized with COVID-19 and PIMS-TS in England during the first pandemic year. Nat Med. 2022;28:193–200. 10.1038/s41591-021-01627-9 34931076

[231] Węcławek-Tompol J Zakrzewska Z Gryniewicz-Kwiatkowska O Pierlejewski F Bień E Zaucha-Prażmo A COVID-19 in pediatric cancer patients is associated with treatment interruptions but not with short-term mortality: a Polish national study. J Hematol Oncol. 2021;14:163. 10.1186/s13045-021-01181-4 34635137 PMC8503711

[232] Wong JJM Gan CS Kaushal SH Chuah SL Sultana R Tan NWH Pediatric COVID-19 Risk Factors in Southeast Asia-Singapore and Malaysia: A Test-Negative Case–Control Study. Am J Trop Med Hyg. 2022;106:1113. 10.4269/ajtmh.21-1000 35168193 PMC8991357

[233] Wong-Chew RM Noyola DE Villa AR Características clínicas y factores de riesgo de mortalidad en menores de 18 años con COVID-19 en México y Ciudad de México [Clinical characteristics and mortality risk factors in patients aged less than 18 years with COVID-19 in Mexico and Mexico City]. An Pediatr (Barc). 2022;97:119–28. 10.1016/j.anpedi.2021.07.014 34603458 PMC8469217

[234] Xiong X Chua GT Chi S Kwan MYW Sang Wong WH Zhou A A Comparison Between Chinese Children Infected with Coronavirus Disease-2019 and with Severe Acute Respiratory Syndrome 2003. J Pediatr. 2020;224:30–6. 10.1016/j.jpeds.2020.06.041 32565097 PMC7301144

[235] Cura Yayla BC Özsürekçi Y Aykaç K Oygar PD Gürlevik SL Ilbay S Characteristics and management of children with COVID-19 in Turkey. Balkan Med J. 2020;37:341. 10.4274/balkanmedj.galenos.2020.2020.7.52 32865382 PMC7590545

[236] Yılmaz Çelebi M Kıymet E Böncüoğlu E Şahinkaya Ş Cem E Düzgöl M Evaluation of childhood hospitalization rates and degree of severity of SARS-CoV-2 variants, including B.1.1.7 (Alpha), B.1.315/P.1 (Beta/Gamma), and B.1.617.2 (Delta). J Med Virol. 2022;94:2050–4. 10.1002/jmv.27587 35018660 PMC9015582

[237] Yılmaz K Gozupirinççioğlu A Aktar F Akın A Karabel M Yolbas I Evaluation of the novel coronavirus disease in Turkish children: Preliminary outcomes. Pediatr Pulmonol. 2020;55:3587–94. 10.1002/ppul.25095 32991038 PMC7536995

[238] Yılmaz K Şen V Aktar F Onder C Yılmaz ED Yılmaz Z Does Covid-19 in children have a milder course than Influenza? Int J Clin Pract. 2021;75:e14466. 10.1111/ijcp.14466 34107134 PMC8237020

[239] Yonker LM Neilan AM Bartsch Y Patel AB Regan J Arya P Pediatric Severe Acute Respiratory Syndrome Coronavirus 2 (SARS-CoV-2): Clinical Presentation, Infectivity, and Immune Responses. J Pediatr. 2020;227:45–52.e5. 10.1016/j.jpeds.2020.08.037 32827525 PMC7438214

[240] Yoon Y Choi G-J Kim JY Kim K-R Park H Chun JK Childcare exposure to severe acute respiratory syndrome coronavirus 2 for 4-year-old presymptomatic child, South Korea. Emerg Infect Dis. 2021;27:341. 10.3201/eid2702.203189 33252327 PMC7853589

[241] Nunziata F Bruzzese E Poeta M Pierri L Catzola A Ciccarelli GP Health-care organization for the management and surveillance of SARS-CoV-2 infection in children during pandemic in Campania region, Italy. Ital J Pediatr. 2020;46:170. 10.1186/s13052-020-00928-y 33198780 PMC7667478

[242] Delahoy MJ Ujamaa D Whitaker M O’Halloran A Anglin O Burns E Hospitalizations Associated with COVID-19 Among Children and Adolescents - COVID-NET, 14 States, March 1, 2020-August 14, 2021. MMWR Morb Mortal Wkly Rep. 2021;70:1255–60. 10.15585/mmwr.mm7036e2 34499627 PMC8437052

[243] Chua GT Xiong X Choi EH Han MS Chang SH Jin BL COVID-19 in children across three Asian cosmopolitan regions. Emerg Microbes Infect. 2020;9:2588–96. 10.1080/22221751.2020.1846462 33138739 PMC7723019

[244] Haeusler GM Ammann RA Carlesse F Groll AH Averbuch D Castagnola E SARS-CoV-2 in children with cancer or after haematopoietic stem cell transplant: an analysis of 131 patients. Eur J Cancer. 2021;159:78–86. 10.1016/j.ejca.2021.09.027 34736044 PMC8501219

[245] Okonkwo IN Howie A Parry C Shelton C Cobley S Craig R The safety of paediatric surgery between COVID-19 surges: an observational study. Anaesthesia. 2020;75:1605–13. 10.1111/anae.15264 32955100 PMC7537528

[246] Otto WR Grundmeier RW Montoya-Williams D Njoroge WF Wallis KE Gerber JS Association between Preferred Language and Risk of Severe Acute Respiratory Syndrome Coronavirus 2 Infection in Children in the United States. Am J Trop Med Hyg. 2021;105:1261. 10.4269/ajtmh.21-0779 34469330 PMC8592181

[247] Shayganmehr A Dorosti AA Saboktakin L Abbasi M Khaiatzadeh S Khoshmaram N Clinical pediatric screening for COVID-19. Iran J Pediatr. 2021;31:e107780. 10.5812/ijp.107780

[248] DU ZS Xu H Liu MC [A retrospective analysis of medication in children with SARS-CoV-2 infection in Wuhan, China]. Zhongguo Dang Dai Er Ke Za Zhi. 2021;23:61–6. Chinese. 33476539 10.7499/j.issn.1008-8830.2007212 PMC7818160

[249] Daoju J Jun S Haomei Y Yan H Jing S Peiqing L [Clinical Characteristics Analysis of 129 Suspected Cases of 2019 Novel Coronavirus Infection in Children in the Guangzhou Region]. Guangzhou Med J (Ft Sam Houst, Tex). 2022;53:12–9. Chinese.

[250] Oran DP Topol EJ The proportion of SARS-CoV-2 infections that are asymptomatic: a systematic review. Ann Intern Med. 2021;174:655–62. 10.7326/M20-6976 33481642 PMC7839426

[251] Syangtan G Bista S Dawadi P Rayamajhee B Shrestha LB Tuladhar R Asymptomatic SARS-CoV-2 carriers: a systematic review and meta-analysis. Front Public Health. 2021;8:587374. 10.3389/fpubh.2020.587374 33553089 PMC7855302

[252] Global Health-50/50. The COVID-19 sex-disaggregated data tracker. 2022. Available: https://globalhealth5050.org/the-sex-gender-and-covid-19-project/the-data-tracker/. Accessed: 5 February 2024.

[253] Yaya S Yeboah H Charles CH Otu A Labonte R Ethnic and racial disparities in COVID-19-related deaths: counting the trees, hiding the forest. BMJ Glob Health. 2020;5:e002913. 10.1136/bmjgh-2020-002913 32513864 PMC7298686

[254] National Health Service. Who is at high risk from coronavirus (COVID-19). 2022. Available: https://www.nhs.uk/conditions/coronavirus-covid-19/people-at-higher-risk/who-is-at-high-risk-from-coronavirus/. Accessed: 5 February 2024.

[255] Jain S Williams DJ Arnold SR Ampofo K Bramley AM Reed C Community-Acquired Pneumonia Requiring Hospitalization among U.S. Children. N Engl J Med. 2015;372:835–45. 10.1056/NEJMoa1405870 25714161 PMC4697461

[256] Lafond KE Nair H Rasooly MH Valente F Booy R Rahman M Global Role and Burden of Influenza in Pediatric Respiratory Hospitalizations, 1982-2012: A Systematic Analysis. PLoS Med. 2016;13:e1001977. 10.1371/journal.pmed.1001977 27011229 PMC4807087

[257] Tian F Yang R Chen Z Safety and efficacy of COVID-19 vaccines in children and adolescents: A systematic review of randomized controlled trials. J Med Virol. 2022;94:4644–53. 10.1002/jmv.27940 35705969 PMC9350282

[258] Mathieu E, Ritche H, Rodés-Guirao L, Appel C, Davrilov D, Giattino C, et al. Coronavirus Pandemic (COVID-19). 2020. Available: https://ourworldindata.org/coronavirus. Accessed: 5 February 2024.

[259] Zimmermann P Pittet LF Curtis N Long covid in children and adolescents. BMJ. 2022;376:o143. 10.1136/bmj.o143 35058281

