# Supplementary material for: Epidemiology and clinical features of SARS-CoV-2 infection in children and adolescents in the pre-Omicron era: A global systematic review and meta-analysis
Source: J Glob Health. 2024 Mar 1;14:05003. doi: 10.7189/jogh.14.05003 (PMC10902805; doi:10.7189/jogh.14.05003)

Contents

Table S1: Age as a risk factor of SARS-CoV-2 infection in people aged ≤18 years 3

Table S2: Summary estimates (mean, median or range) of age in SARS-CoV-2 positive and SARS-CoV-2 negative children and adolescents 4

Table S3: Results of adjusted analyses for age as a risk factor of SARS-CoV-2 infection in people aged ≤18 years 5

Table S4: Results of adjusted analyses for sex as a risk factor of SARS-CoV-2 infection in people aged ≤18 years 6

Table S5: Results of adjusted and unadjusted analyses in individual studies for race/ ethnicity as a risk factor of SARS-CoV-2 infection in people aged ≤18 years 7

Table S6: Results of adjusted analyses in individual studies for comorbidities as a risk factor of SARS-CoV-2 infection in people ≤18 years 9

Table S7: Number of SARS-CoV-2 infections presenting with severe COVID-19 disease in people aged ≤18 years 11

Table S8: Number of SARS-CoV-2 infections presenting with critical COVID-19 disease in people aged ≤18 years 13

Table S9: Study characteristics of included studies 15

Table S10: Risk of bias assessment of individual studies 26

Table S11: List of studies excluded during full text review with reasons for exclusion 38

Table S12: PRISMA checklist 130

Table S13: PRISMA abstract checklist 133

Table S14: Outcomes reported by each study 134

Text S1: Selection criteria for inclusion of studies in the review 151

Inclusion criteria 151

Exclusion criteria 151

Text S2: Search strategies 153

Text S3: List of variables for which data were extracted 159

Text S4: Checklists used for quality appraisal of included studies (according to study design) 161

Figure S1: Forest plot: Pooled proportion of positive SARS-CoV-2 tests in people aged ≤18 years 163

Figure S2: Influential plots for test positivity analysis 164

Figure S3: Subgroup analysis: Plot of subgroup analysis according to WHO region 165

Figure S4: Subgroup analysis: Plot of subgroup analysis according to country income level 166

Figure S5: Subgroup analysis: Plot of subgroup analysis according to study setting 167

Figure S6: Subgroup analysis: Plot of subgroup analysis according to testing method 168

Figure S7: Subgroup analysis: Plot of subgroup analysis according to SARS-CoV-2 dominant variant in the country at the mid study time 169

Figure S8: Forest plot: Pooled relative risk of male sex as risk factor of SARS-CoV-2 infection in people aged ≤18 years 170

Figure S9: Forest plot: Pooled proportion of people aged ≤18 years presenting with asymptomatic SARS-CoV-2 infection 171
Figure S10: Forest plot: Pooled proportion of people aged ≤18 years presenting with mild COVID-19 disease 172
Figure S11: Forest plot: Pooled proportion of people aged ≤18 years presenting with moderate severity of COVID-19 disease 173
Figure S12: Forest plot: Pooled proportion of people aged ≤18 years presenting with severe or critical COVID-19 disease 174
Figure S13: Forest plot: Pooled proportion of SARS-CoV-2 infections in people aged ≤18 years requiring hospital admission 175
Figure S14: Forest plot: Pooled proportion of SARS-CoV-2 infections in people aged ≤18 years requiring oxygen supplementation. 176
Figure S15: Forest plot: Pooled proportion of SARS-CoV-2 infections in people aged ≤18 years requiring ICU admission 177
Figure S16: Forest plot: Pooled proportion of SARS-CoV-2 infections in people aged ≤18 years requiring assisted ventilation 178
Figure S17: Forest plot: Pooled case fatality rate of SARS-CoV-2 infections in people aged ≤18 years 179

Table S1: Age as a risk factor of SARS-CoV-2 infection in people aged ≤18 years

**Study**	**Age group**	**Total sample**	**a**	**b**	**c**	**d**
Hernandez-Garduno 2020	<1 year	3696	104	291	857	2444
Lazzerini 2021	<6 months	2148	19	159	140	1830
Lazzerini 2021	6 months to < 2years	2148	17	472	142	1517
Lorenzo 2021	<1 year	210	18	47	44	101
Michos 2021	<1 year	4901	20	1180	70	3631
Alp 2021	28 days to 4 years	135	14	41	27	53
Armocida 2022	<6 years	1484	27	589	100	768
Calvani 2021	0 to 5 years	2837	28	772	68	1969
Hernandez-Garduno 2020	1 to 5 years	3696	173	746	788	1989
Michos 2021	1 to 5 years	4901	15	1691	75	3120
Olivar-Lopez 2020	2.1 years to 6 years	510	16	118	63	313
Paduano 2021	<6 years	349	18	81	60	190
Alp 2021	5 to 9 years	135	8	27	33	67
Armocida 2022	6 to 10 years	1484	30	330	97	1027
Calvani 2021	6 to 10 years	2837	38	1023	58	1718
Cheng 2022	≤12 years	239	148	20	61	10
Cohen 2021	≤12 years	64409	2939	31553	3268	26649
Galli 2021	5 to 14 years	631	1	130	65	435
Hernandez-Garduno 2020	6 to 10 years	3696	162	576	799	2159
Lazzerini 2021	2 to 9 years	2148	37	836	122	1153
Michos 2021	6 to 10 years	4901	18	948	72	3863
Olivar-Lopez 2020	6.1 to 12 years	510	11	93	68	338
Paduano 2021	6 to 13 years	349	35	112	43	159
Alp 2021	10 to 14 years	135	6	13	35	81
Alp 2021	15 to 18 years	135	13	13	28	81
Armocida 2022	10 to 14 years	1484	70	438	57	919
Calvani 2021	11 to 18 years	2837	30	946	66	1795
Hernandez-Garduno 2020	11 to 15 years	3696	300	712	661	2023
Hernandez-Garduno 2020	16 to 17 years	3696	222	410	739	2325
Lazzerini 2021	10 to 18 years	2148	86	517	73	1472
Michos 2021	11 to 16 years	4901	37	992	53	3819
Olivar-Lopez 2020	12.1 to <18 years	510	25	97	54	334
Paduano 2021	14 or more years	349	25	78	53	193

a = people belonging to the group mentioned in the age group column and testing positive for SARS-CoV-2 infection
b = people belonging to the group mentioned in the age group column and testing negative for SARS-CoV-2 infection
c = people aged ≤18 years but not belonging to the group mentioned in the age group column and testing positive for SARS-CoV-2 infection
d = people aged ≤18 years but not belonging to the group mentioned in the age group column and testing negative for SARS-CoV-2 infection

Table S2: Summary estimates (mean, median or range) of age in SARS-CoV-2 positive and SARS-CoV-2 negative children and adolescents

Study	Risk	Total sample size for continuous variable analysis	SARS-CoV-2 positive group	SARS-CoV-2 negative group	p-value for comparison between continuous variables
Ahmed 2021	mean age in years (S.D.)	148	9.11 (1.9)	8.13 (1.7)	0.001
Alp 2021	mean age in years (S.D.)	135	8.8 (6.4)	7.0 (5.5)	0.144
Arslan 2021	median age in months (IQR)		79 (34 to 149)	30.5 (9 to 82)	
Bandi 2020	mean age in years (S.D.)	474	9.72 (7.13)	4.85 (5.65)	
Calvani 2021	mean age in years (S.D.)	2837	8.52 (4.52)	8,6 (4.5)	0.798
Hernandez-Garduno 2020	median age in years (IQR)		11 (4 to 15)	9 (3 to 14)	<0.0001
Karaci 2021	median age in years (IQR)	581	123 (59 to 177.2)	118 (51-185)	0.84
Lorenzo 2021	median age in years (IQR)	210	2.8 (0.6 to 6.1)	2.6 (0.5 to 6.3)	0.7
Lorenzo 2021	age range in years	210	0 to 14.7	0 to 16.4	
Mele 2021	median age in years (IQR)	110	16.8 (11.7 to 17.6)	3.5 (0.9 to 7.5)	0.004
Meyer 2021	median age in years (IQR)	5730	12 (0.8 to 17.4)	5.2 (0 to 17.9)	
Schneider 2021	median age in years (IQR)		2.78 (0.10 to 14.02)	2.21 (0.62 to 8.79)	0.72

Table S3: Results of adjusted analyses for age as a risk factor of SARS-CoV-2 infection in people aged ≤18 years

Study	Risk	Total sample size for adjusted Odds Ratio analysis	Adjusted Odds Ratio	Adjusted 95% lower CI	Adjusted 95% upper CI	p-value for adjusted Odds R atioanalysis	Factors adjusted for
Armocida 2022	<6 years	1484	1.00				contact with COVID-19 case, symptoms
Armocida 2022	6 to 10 years	1484	3.20	1.70	6.10	<0.001	contact with COVID-19 case, symptoms
Armocida 2022	10 to 14 years	1484	4.80	2.70	8.80	<0.001	contact with COVID-19 case, symptoms
Bandi 2020	mean age in years (S.D.)	474	1.09	1.06	1.78		logistic regression adjusting for all demographics in one model.
Elif 2021	Age	112	1.09	0.97	1.22	0.161	vitamin D, BMI, gender
Lazzerini 2021	<6 months	2148	1.12	0.60	2.11	0.725	comorbidity, contact with COVID-19 case, relatives with respiratory symptoms
Lazzerini 2021	6 months to < 2years	2148	0.43	0.15	1.20	0.107	comorbidity, contact with COVID-19 case, relatives with respiratory symptoms
Lazzerini 2021	2 to 9 years	2148	0.33	0.22	0.50	<0.0001	comorbidity, contact with COVID-19 case, relatives with respiratory symptoms
Lazzerini 2021	10 to 18 years	2148	1.00				comorbidity, contact with COVID-19 case, relatives with respiratory symptoms
Murillo-Zamora 2020	< 3 years	1849	1.00				sex, population by place of residence, household contact with case, sudden onset symptoms, disease severity, obesity, asthma, immunosuppression
Murillo-Zamora 2020	3 to 5 years	1849	1.04	0.70	1.55	0.841	sex, population by place of residence, household contact with case, sudden onset symptoms, disease severity, obesity, asthma, immunosuppression
Murillo-Zamora 2020	6 to 12 years	1849	1.10	0.78	1.53	0.592	sex, population by place of residence, household contact with case, sudden onset symptoms, disease severity, obesity, asthma, immunosuppression
Murillo-Zamora 2020	13 to 15 years	1849	2.08	1.46	2.96	<0.001	sex, population by place of residence, household contact with case, sudden onset symptoms, disease severity, obesity, asthma, immunosuppression

Table S4: Results of adjusted analyses for sex as a risk factor of SARS-CoV-2 infection in people aged ≤18 years

Author (year)	Risk group	Total sample size for adjusted Odds Ratio analysis	Adjusted Odds Ratio	Adjusted 95% lower CI	Adjusted 95% upper CI	p-value	Factors adjusted for
Armocida 2022	Female	1484	1.49	1.00	2.30	0.073	cohabitation with a case, age, fevers, chills, headache, ageusia, sore throat, earache, rhinorrhoea, diarrhoea
Arslan 2021	Male	404	1.70	0.69	4.01	0.253	cough, shortness of breath, sore throat, rhinorrhea, smell-taste loss, positive contact, and fever > 38 °C.
Elif 2021	Male	112	0.87	0.32	2.34	0.784	age, BMI standard deviation score, vitamin D
Murillo-Zamora 2020	Male	1849	1.08	0.83	1.40	0.56	age, population by place of residence, household contact with case, sudden onset symptoms, disease severity, obesity, asthma, immunosuppression

Table S5: Results of adjusted and unadjusted analyses in individual studies for race/ ethnicity as a risk factor of SARS-CoV-2 infection in people aged ≤18 years

Study	Race/ethnicity	Total sample size	Adjusted Odds ratio	Adjusted 95% lower CI	Adjusted 95% upper CI	p-value for adjusted analysis	Factors adjusted for	Unadjusted Risk ratio	Unadjusted 95% lower CI	Unadjusted 95% upper CI
Bandi 2020	Non-hispanic white	474	1.00				logistic regression adjusting for all demographics in one model.	0.26	0.06	1.08
Bandi 2020	Black	474	3.10	1.23	5.34			1.67	0.77	3.6
Bandi 2020	Hispanic	474	2.10	0.97	4.67			1.44	0.64	3.24
Bandi 2020	Asian	474						1.15	0.08	17.24
Bandi 2020	Other	474	1.30	0.56	4.34			0.75	0.11	5.31
Cohen 2021	not available	64409						1.56	1.44	1.69
Cohen 2021	Secular Jew	64409						0.65	0.62	0.69
Cohen 2021	Arabs	64409						0.89	0.74	1.08
Cohen 2021	Ultraorthodox Jew	64409						1.52	1.41	1.64
Lorenzo 2021	Mixed (self- reported)	210						1.18	0.21	6.55
Mele 2021	Caucasian	110						1.25	0.76	2.05
Schneider 2021	White non-Hispanic	263						0.11	0.03	0.46
Schneider 2021	Black non-Hispanic	263						1.59	0.62	4.06
Schneider 2021	Hispanic or Latino	263	6.30	2.00	19.70	0.001		5.77	2.46	13.55
Schneider 2021	Asian or Pacific Islander	263						5.10	0.96	26.95
Schneider 2021	Other	263						7.68	1.78	33.06
Schneider 2021	Unknown	263						0.28	0.02	4.57

Otto 2021	White non- Latino	10138						0.77	0.68	0.87
Otto 2021	Black non-Latino	10138						1.17	1.06	1.30
Otto 2021	Hispanic/ Latino	10138						1.32	1.14	1.54
Otto 2021	Asian non-Latino	10138						1.15	0.91	1.47
Otto 2021	Multiracial non-Latino	10138						0.69	0.48	0.99
Otto 2021	Other/ unknown	10138						0.77	0.60	0.99

Table S6: Results of adjusted analyses in individual studies for comorbidities as a risk factor of SARS-CoV-2 infection in people ≤18 years

Study	Comorbidity	Total sample size	Adjusted Odds Ratio	Adjusted 95% lower CI	Adjusted 95% upper CI	p-value	Factors adjusted for
Cohen 2020	ADHD treated	13300	0.64	0.31	1.31	0.218	age, sex, ethnicity
Cohen 2020	ADHD untreated	13300	1.43	1.07	1.91	0.016	age, sex, ethnicity
Cohen 2020	non-ADHD	13300	1.00				age, sex, ethnicity
Murillo- Zamora 2020	Asthma	1849	0.55	0.29	1.06	0.075	sex, age, population by place of residence, sudden onset symptoms, household contact with case, disease severity, obesity, immunosuppression
Hernandez-Garduno 2020	Diabetes		3.02	1.25	7.32	0.014	age, gender, hospitalisation status, contact with COVID-19 case, current smoking status, pneumonia, underlying disease, pregnancy
Lazzerini 2021	Cardiac disease	2148	3.1	1.19	5.02	<0.0001	age, contact with COVID-19 case, relatives with respiratory symptoms
Schneider 2020	History of hypoxic ischaemic encephalopathy infection	261	16.5	2.5	108	0.004	demographic and clinical factors significantly associated with SARS-CoV-2 infection in univariate analysis
Hernandez-Garduno 2020	Hypertension		3.95	1.32	11.97	0.015	age, gender, hospitalisation status, contact with COVID-19 case, current smoking status, pneumonia, underlying disease, pregnancy
Murillo- Zamora 2020	Immunosuppression*	1849	1.22	0.79	1.91	0.387	sex, age, population by place of residence, sudden onset symptoms, household contact with case, disease severity, obesity, asthma
Hernandez-Garduno 2020	Immunosuppression**		1.59	1.07	2.37	0.022	age, gender, hospitalisation status, contact with COVID-19 case, current smoking status, pneumonia, underlying disease, pregnancy
Murillo- Zamora 2020	Obesity	1849	2.05	1.11	3.79	0.022	sex, age, population by place of residence, sudden onset symptoms, household contact with case, disease severity, asthma, immunosuppression
Schneider 2020	Obesity	263	23.8	1.10	531	0.05	demographic and clinical factors significantly associated with SARS-CoV-2 infection in univariate analysis
Hernandez-Garduno 2020	Obesity		5.11	2.78	9.39	<0.001	age, gender, hospitalisation status, contact with COVID-19 case, current smoking status, pneumonia, underlying disease, pregnancy
Hernandez-Garduno 2020	Boys only and obesity		3.5	1.67	7.34	0.0009	

Elif 2021	BMI- standard deviation score		1.02	0.62	1.69	0.936	
Hernandez-Garduno 2020	Girls only and obesity	112	5.13	2	13.15	0.0007	

*Any identified cause of the related deficiency except for the personal history of diabetes mellitus, human virus immunodeficiency infection, chronic kidney disease, or asthma.
** Without specification

Table S7: Number of SARS-CoV-2 infections presenting with severe COVID-19 disease in people aged ≤18 years

Study	Total SARS-CoV-2 infections	Severe COVID-19 disease
Ozlu 2022	100	0
Pande 2021	100	27
Sharma 2021	100	44
Yilmaz 2020	105	3
Alshengeti 2022	106	4
Ennab 2021	111	0
Ennab 2021	111	32
Kushner 2021	117	9
Maltezou 2020	118	1
Sharma 2020	121	1
Tang 2021	127	3
Qian 2021	127	7
Jiang 2022	129	0
Alsharrah 2020	134	0
Shahid 2021	141	17
Indriyani 2021	146	43
Capra 2021	152	0
Lazzerini 2021	159	5
Loconsole 2020	166	3
Sedighi 2021	166	70
Chiang 2021	169	4
Parri 2020	170	1
Ustundag 2021	173	4
Arslan 2021	176	0
Alattas 2021	180	3
Du 2021	182	1

Hobbs 2022	206	104
Bai 2021	209	2
Karaci 2021	222	9
Ji 2021	241	6
Singh 2022	255	42
Gujar 2021	274	0
Ferraro D 2021	280	16
Hendler 2021	288	196
Kara 2021	292	12
Murillo- Zamora 2020	294	78
Wong 2022	326	0
Saleh 2021	398	103
Krithika 2021	400	3
Anugulruengkitt 2021	416	12
Oslon 2022	445	196
Sananez 2021	550	8
Rabha 2021	588	43
Goktug 2021	621	5
Bayesheva 2021	650	4
Buta 2022	701	59
Dong 2020	726	18
Karbuz 2021	1156	16
See 2021	1489	4
Reis 2021	2089	0
Bellino 2020	3836	79

Table S8: Number of SARS-CoV-2 infections presenting with critical COVID-19 disease in people aged ≤18 years

Study	Total SARS-CoV-2 infections	Critical COVID-19 disease
Pande 2021	100	10
Besli 2021	104	4
Yilmaz 2020	105	0
Alshengeti 2022	106	1
Rabha 2021	115	0
Kushner 2021	117	15
Kapoor 2021	120	36
Sharma 2020	121	0
Jiang 2022	129	0
Indriyani 2021	146	6
Capra 2021	152	0
Lazzerini 2021	159	2
Parri 2020	170	1
Arslan 2021	176	1
Corso 2021	179	41
Du 2021	182	3
Bai 2021	209	1
Guzman 2021	213	20
Karaci 2021	222	1
Ji 2021	241	8
Singh 2022	255	31
Gujar 2021	274	0
Ferraro D 2021	280	20
Hendler 2021	288	92
Wong 2022	326	3
Anugulruengkitt 2021	416	0
AlGhamdi 2022	567	0
Rabha 2021	588	18
Goktug 2021	621	4
Buta 2022	701	0
Dong 2020	726	3
See 2021	1489	0
Bellino 2020	3836	7

Jang 2022	14967	1
Lee 2021	39146	8

Table S9: Study characteristics of included studies

Study ID	Covidence	Author name	Published year	Datacollection start month	Datacollection start year	Datacollection end month	Datacollection end year	Country
110	13198	Abo	2021	March	2020	November	2020	Australia
176	13791	Ahmed	2021	February	2020	May	2021	Egypt
37	12524	Aizawa	2021	November	2020	December	2020	Japan
47	13565	Akkoc	2021	March	2020	May	2020	Turkey
65	12118	Alattas	2021	March	2020	June	2020	United Arab Emirates
174	14215	AlGhamdi	2022	March	2020	July	2020	Saudi Arabia
87	18667	Alharbi	2021	April	2020	July	2020	Saudi Arabia
86	12167	Almuzaini	2021	March	2020	April	2020	Saudi Arabia
243	13654	Alonso	2021	April	2020	January	2021	United States of America
15	14329	Alp	2021	March	2020	May	2020	Turkey
22	18474	Alqayoudhi	2021	February	2020	May	2020	Oman
43	12046	Alsharrah	2020	February	2020	April	2020	Kuwait
45	14219	Alshengeti	2022	May	2020	July	2020	Saudi Arabia
80	17026	Alshukairi	2021	March	2020	August	2021	Saudi Arabia
94	18847	Ana Laura	2021	May	2020	August	2020	Mexico
181	12731	Antúnez-Montes	2021	July	2020	August	2020	Mexico, Colombia,
68	12143	Anugulruengkitt	2021	April	2021	July	2021	Thailand
150	17010	Aprea	2021	April	2020	June	2020	Argentina

220	20057	Arellano-Llamas	2020	February	2020	May	2020	Mexico
61	12209	Armocida	2022	September	2020	December	2020	Italy
60	12043	Arslan	2021	March	2020	May	2020	Turkey
93	11993	Asseri	2021	May	2020	October	2020	Saudi Arabia
85	20144	Ayed	2020	March	2020	May	2020	Kuwait
34	14146	Bai	2021	January	2020	February	2020	China
197	10899	Bandi	2020	March	2020	April	2020	e Principe, Sierra Leone, and Uganda
185	18317	Barrera	2021	March	2020	June	2021	Mexico
148	15768	Baumgarte	2022	September	2020	September/ October	2020	Germany
34	14146	Bayesheva	2021	March	2020	June	2020	Kazakhstan
90	12845	Bellino	2020	February	2020	May	2020	Italy
1	11967	Berksoy	2021	March	2020	July	2020	Turkey
58	12938	Besli	2021	March	2020	July	2020	Turkey
29	15039	Biko	2020	March	2020	May	2020	United States of America
194	11943	Bolanos-Almeida	2021	March	2020	June	2020	Colombia
107	17624	Brotons	2021	April	2020	June	2020	Spain
229	17729	Buonsenso	2021	March	2020	October	2020	Italy
179	12204	Buta	2022	March	2020	June	2021	Moldova
113	18120	Calvani	2021	October	2020	December	2020	Italy
72	17455	Capozza	2021	April	2020	December	2020	Italy
151	14391	Capra	2021	March	2020	August	2020	Argentina
30	18681	Carrasco	2021	March	2020	July	2020	Spain

53	20080	Celik	2021	March	2020	June	2020	Turkey
95	12127	Cheng	2022	June	2020	January	2021	United States of America
17	15615	Chiang	2021	September	2020	November	2020	Argentina, Belarus, Brazil, Greece, Guatemala, Iran, Kyrgyzstan, the Kingdom of Saudi Arabia, Spain, and Sudan
10	10777	Chopra	2021	March	2020	November	2020	India
169	17381	Chowdhoury	2021	May	2020	September	2020	India
144	12010	Chua	2021	January	2020	December	2020	Hong Kong
254	12887	Chua	2020	January	2020	May	2020	Republic of Korea, China, and Hong Kong SAR
137	14451	Ciofi Degli Atti	2020	January	2020	April	2020	Italy
139	17094	Cloete	2022	October	2021	December	2021	South Africa
218	12132	Cofre	2020	March	2020	June	2020	Chile
207	11196	Cohen	2021	February	2020	October	2020	Israel
119	11841	Colson	2020	January	2020	May	2020	France
141	11841	Colson	2020	February	2020	March	2020	France
101	18590	Cooper	2021	September	2020	March	2020	United States of America
173	18622	Corso	2021	April	2020	March	2021	Brazil
193	12906	Dash	2021	March	2020	August	2021	India
149	15547	Dawood	2022	September	2020	August	2021	United States of America
235	14905	Delahoy	2021	March	2020	August	2021	United States of America

63	18417	deLusignan	2020	January	2020	April	2020	England
20	12361	Devrim	2022	March	2021	August	2021	Turkey
154	16765	Dilber	2021	March	2020	January	2021	Turkey
217	13432	Dominguez Rojas	2021	March	2020	August	2020	Peru
147	11258	Dominguez-Rodriguez	2021	March	2020	July	2020	Spain
239	14191	Dong	2020	January	2020	February	2020	China
249		Du	2021	January	2020	March	2020	China
125	12015	Du	2021	January	2020	February	2020	China
71	18117	Eleftheriou	2021	September	2020	February	2021	Greece
153	12898	Elghoudi	2020	March	2020	May	2020	United Arab Emirates
26	19860	Elif	2021	March	2020	April	2020	Turkey
35	19823	Engels	2022	November	2020	April	2021	Germany
11	12021	Ennab	2021	March	2020	June	2020	United Arab Emirates
111	15528	Ergenc	2021	March	2020	December	2020	Turkey
170	16095	Erturk	2021	May	2020	November	2020	Turkey
214	14144	Ferraro D	2022	April	2020	September	2020	Argentina
234	14530	Forster	2022	October	2020	March	2021	Germany
51	19329	Foster	2020	March	2020	June	2020	United States of America
213	17042	Funk	2022	March	2020	June	2021	Argentina, Canada, Costa Rica, Italy, Paraguay, Singapore, Spain, United States of America
24	14176	Gaborieau	2020	March	2020	May	2020	France
109	19930	Galli	2021	February	2020	April	2020	Italy

38	12844	Gampel	2020	March	2020	April	2020	United States of America
129	16369	Garazzino	2020	March	2020	April	2020	Italy
67	11758	Gavriliu	2021	April	2020	October	2020	Romania
2	12220	Ghosh	2020	April	2020	August	2020	Bangladesh
46	14321	Goktug	2021	March	2020	December	2020	Turkey
237	18315	Gomes	2021	March	2020	August	2020	Brazil
180	12888	Gotzinger	2020	April	2020	April	2020	Austria, Belgium, Bulgaria, Croatia, Denmark,
231	19181	Gudbjartsson	2020	March	2020	April	2020	Iceland
89	11470	Gujar	2021	February	2020	July	2020	India
171	12032	Gumus	2021	March	2020	December	2020	Turkey
91	10914	Guzman	2021	March	2020	January	2021	United States of America
257	18623	Haeusler	2021	unclear	unclear	February	2021	Austria, Germany, Italy, Switzerland, United Kingdom, Brazil, Canada, Russia, Israel, Australia,
102	12147	Hedberg	2022	February	2020	April	2020	Sweden
191	18426	Hendler	2021	March	2020	December	2020	Brazil
208	12292	Hernandez-Garduno	2020	March	2020	July	2020	Mexico
92	17005	Hijazi	2021	January	2020	July	2020	Saudi Arabia
200	14625	Hobbs	2022	March	2020	August	2020	United States of America
159	14562	Howard	2021	March	2020	September	2020	United States of America
162	17773	Huete-Perez	2021	October	2020	November	2020	Nicaragua
105	11754	Ibrahim	2021	February	2020	September	2020	Australia

230	18738	Ibrahim	2020	March	2020	April	2020	Australia
177	18509	Imamura	2021	January	2020	October	2020	Japan
116	11703	Indriyani	2021	March	2020	July	2020	Indonesia
25	12383	Isoldi	2021	April	2020	June	2020	Italy
205	14149	Jang	2022	January	2020	June	2021	South Korea
99	11737	Ji	2021	January	2020	April	2020	China
248		Jiang	2022	January	2020	February	2020	China
54	17583	Jimenez-Garcia	2021	March	2020	May	2020	Spain
64	14392	Kaba	2021	March	2020	June	2020	Turkey
201	11962	Kaliyan	2021	March	2020	June	2021	India
7	13359	Kanthimathinathan	2020	March	2020	April	2020	United Kingdom
178	15108	Kapoor	2021	March	2020	December	2020	India
33	14331	Kara	2021	March	2020	December	2020	Turkey
79	12245	Karaaslan	2021	March	2020	May	2020	Turkey
73	19023	Karaci	2021	March	2020	April	2020	Turkey
55	14170	Karbuz	2021	March	2020	June	2020	Turkey
75	18565	Kavanagh	2021	September	2020	December	2020	Ireland
28	12840	Kepenekli	2022	unclear	unclear	unclear	unclear	Turkey
225	14904	Kim	2020	March	2020	July	2020	United States of America
161	16921	Krithika	2021	March	2020	May	2021	India
32	11883	Kuchar	2021	March	2020	May	2020	Poland
78	17757	Kuczborska	2021	November	2020	April	2021	Poland
142	14210	Kufa	2022	February	2020	September	2020	South Africa
124	17040	Kumar	2021	April	2020	unclear	unclear	India
175	14601	Kushner	2021	May	2020	February	2021	United States of America
232	18693	Ladhani	2021	September	2020	December	2020	England
236	14522	Lanari	2021	November	2020	April	2021	Italy

132	11715	Lazzerini	2021	February	2020	May	2020	Italy
157	10983	Lee	2022	January	2021	October	2021	South Korea
228	13257	Leidman	2021	March	2020	December	2020	United States of America
206	11659	Levy	2020	March	2020	May	2020	France
233	14462	Lindsay	2021	February	2020	July	2020	United States of America
143	18392	Liu	2020	January	2020	March	2020	China
146	13612	Liu	2020	January	2020	January	2020	China
42	18661	Loconsole	2020	March	2020	June	2020	Italy
184	11887	Lopez-Aguilar	2020	March	2020	June	2020	United States of America
121	13694	Lorenzo	2021	April	2020	April	2021	Brazil
27	19361	Lu	2020	January	2020	March	2020	China
103	18653	Lu	2020	January	2020	February	2020	China
12	16059	Lynam	2021	February	2020	June	2020	Ireland
192	18165	Madani	2021	February	2020	November	2020	Iran
39	11847	Maltezou	2020	February	2020	June	2020	Greece
13	19960	Mania	2021	February	2020	May	2020	Poland
134	17584	Mania	2022	March	2020	December	2020	Poland
41	12041	Matteudi	2021	February	2020	May	2020	France
57	16052	Mele	2021	March	2020	May	2020	Spain
126	18300	Messiah	2021	March	2020	March	2021	Singapore and Malaysia
59	13238	Meyer	2021	March	2020	December	2020	Germany
88	16311	Meyer	2022	March	2020	November	2021	Germany
81	18704	Michos	2021	March	2020	June	2020	Greece
222	11954	Morban	2021	March	2020	July	2020	Dominican Republic
202	18395	Moreno-Noguez	2021	March	2020	May	2020	Mexico
48	17716	Murillo- Zamora	2020	February	2020	August	2020	Mexico
130	17759	Musa	2021	March	2020	July	2020	Qatar

163	13825	MveangNzoghe	2021	March	2020	August	2020	Gabon
190	13292	Navarro-Olivos	2021	March	2020	December	2020	Mexico
70	11947	Ng	2021	February	2020	December	2020	Malaysia
156	14784	Nunziata	2020	March	2020	September	2020	Italy
69	19294	Odeleye	2021	April	2020	July	2020	United Kingdom
256	18568	Okonkwo	2020	March	2020	July	2020	United Kingdom
108	12113	Okur	2021	March	2020	January	2021	Turkey
21	12169	Olivar-Lopez	2020	March	2020	June	2020	Mexico
187	12008	Oliveira	2021	February	2020	January	2021	United Kingdom
98	17910	Ollier	2022	January	2021	May	2021	France
244	13982	Olson	2022	May	2021	October	2021	United States of America
106	14571	Omrani	2020	May	2020	June	2020	Qatar
182	11835	Ortiz- Pinto	2022	March	2020	May	2021	Peru, Costa Rica and Brazil
199	18407	Osmanov	2022	April	2020	August	2020	Russia
255	11134	Otto	2021	March	2020	February	2021	United States of America
183	14212	Otto	2020	March	2020	June	2020	Spain
97	11364	Ozlu	2022	April	2020	July	2020	Turkey
168	11716	Paduano	2021	February	2020	June	2020	Italy
152	12162	Pande	2021	April	2020	August	2020	India
186	15539	Pandey	2020	March	2020	July	2020	United States of America
77	12915	Parambil	2021	unclear	unclear	September	2020	India
211	18316	Parcha	2021	April	2020	October	2020	United States of America
114	12864	Parri	2020	March	2020	May	2020	Italy
224	18904	Peaper	2021	March	2020	September	2020	United States of America
52	17051	Peng	2021	January	2020	March	2020	China

135	18798	Perramon	2021	September	2020	May	2021	Spain
56	12338	Pokorska-Spiewak	2021	February	2020	April	2020	Poland
128	11945	Pokorska-Spiewak	2021	March	2020	December	2020	Poland
209	17273	Pudjiadi	2021	March	2020	December	2020	Indonesia
120	18170	Qian	2021	January	2020	March	2020	China
172	12124	Rabha	2021	March	2020	June	2020	Brazil
210	11176	Rabha	2021	March	2020	January	2021	Brazil
242	13983	Reis	2021	June	2021	September	2021	Israel
4	18898	Rha	2020	January	2020	March	2020	United States of America
189	14463	Rivas-Ruiz	2020	February	2020	May	2020	Germany
117	18779	Rizzo	2021	November	2019	March	2020	Italy
195	19524	Rodriguez Velasquez	2021	April	2020	September	2020	Botswana, Burkina Faso, Chad, Congo, Eswatini, Guinea,
96	18260	Rose	2021	March	2020	May	2020	Italy
84	12348	Sahni	2021	April	2020	July	2020	United Kingdom
160	17768	Salako	2021	March	2020	June	2020	Nigeria
82	18966	Saleh	2021	March	2020	November	2020	Egypt
117	17604	Sananez	2021	May	2020	January	2021	Argentina
122	15025	Schneider	2021	March	2020	May	2020	United States of America
115	16363	Sedighi	2021	March	2020	May	2020	Iran
104	11948	See	2021	January	2020	December	2020	Malaysia
198	11996	Sena	2021	February	2020	June	2020	Brazil
50	12084	Shahid	2021	April	2020	July	2020	Pakistan
131	12894	Shapiro Ben David	2021	February	2020	July	2020	Israel
3	14140	Sharma	2020	January	2020	August	2020	Nepal
6	17720	Sharma	2021	April	2020	October	2020	India

253	12146	Shayganmehr	2021	March	2020	April	2020	Iran
238	18439	Shi	2022	March	2020	July	2021	Scotland
158	12033	Shoji	2021	January	2020	February	2021	Japan
252	12357	Shoji	2022	October	2020	October	2021	Japan
166	12163	Singh	2022	April	2020	October	2020	India
196	17820	Sola	2021	March	2020	May	2020	Liberia, Mauritius, Mozambique, Namibia, Niger, Rwanda, São Tomé
241	11753	Somekh	2021	October	2020	February	2021	Israel
165	12277	Song	2021	March	2020	December	2020	United States of America
136	14919	Soriano-Arandes	2021	July	2020	October	2020	Spain
167	16835	Sousa	2021	January	2020	December	2020	Brazil
226	12523	Stokes	2020	January	2020	May	2020	United States of America
44	14125	Talarico	2021	March	2020	February	2021	Italy
245	12080	Tang	2021	January	2020	March	2020	China
16	11710	Tosif	2021	March	2020	September	2020	Australia
164	14465	Uka	2021	March	2020	October	2020	Switzerland
40	12974	Ustundag	2021	March	2020	September	2020	Turkey
138	12076	van der Zalm	2021	April	2020	July	2020	South Africa
76	13788	Verd	2021	August	2020	December	2020	Spain
5	14883	Vergine	2020	March	2020	April	2020	Italy
188	18725	Vogel	2022	March	2021	May	2021	Brazil
8	11261	Wang	2020	January	2020	March	2020	China
9	18807	Wang	2020	January	2020	April	2020	China
227	11691	Wanga	2021	July	2021	August	2021	United States of America
203	18408	Ward	2022	February	2020	January	2021	England
74	12998	Węcławek-Tompol	2021	March	2020	February	2021	Poland

126	17266	Wong	2022	January	2020	March	2021	United States of America
219	11997	Wong-Chew	2021	February	2020	September	2020	Mexico
14	12319	Xiong	2020	January	2020	March	2020	China
18	11696	Yayla	2020	March	2020	June	2020	Turkey
31	14344	Yilmaz	2020	April	2020	June	2020	Turkey
66	13790	Yilmaz	2021	March	2020	November	2020	Turkey
145	14312	Yilmaz Celebi	2022	March	2021	August	2021	Turkey
36	17403	Yonker	2020	unclear	unclear	unclear	unclear	United States of America
100	11820	Yoon	2021	February	2020	unclear	unclear	South Korea

Table S10: Risk of bias assessment of individual studies

Cohort studies

Author name	Published year	Covidence number	Study ID	Were the two groups similar and recruited from the same population?	Were the exposures measured similarly to assign people to both exposed and unexposed groups?	Was the exposure measured in a valid and reliable way?	Were confounding factors identified?	Were strategies to deal with confounding factors stated?	Were the groups/participants free of the outcome at the start of the study (or at the moment of exposure)?	Were the outcomes measured in a valid and reliable way?	Was the follow up time reported and sufficient to be long enough for outcomes to occur?	Was follow up complete, and if not, were the reasons to loss to follow up described and explored?	Were strategies to address incomplete follow up utilized?	Was appropriate statistical analysis used?	Total score
Ahmed	2021	13791	176	0	1	1	1	0	0	1	0	1	not applicable	1	6
Aizawa	2021	12524	37	1	0	1	1	0	1	0	0	1	not applicable	1	6
Akkoc	2021	13565	47	0	1	1	0	0	0	1	0	0	0	1	4
Alattas	2021		65	0	1	1	1	0	0	1	0	1	not applicable	1	6
AlGhamdi	2022	14215	174	0	1	1	1	0	0	1	0	1	not applicable	1	6
Alharbi	2021		87	1	1	1	1	0	0	1	0	1	not applicable	1	7
Almuzaini	2021	12167	86	1	1	1	1	0	0	0	0	1	not applicable	1	6
Alonso	2021		243	0	1	1	1	1	0	1	0	1	not applicable	1	7
Alp	2021	14329	15	1	1	1	1	0	1	1	0	1	not applicable	1	8
Alsharrah	2020	12046	43	0	1	1	1	1	0	1	0	1	not applicable	1	7
Alshengeti	2022	14219	45	0	1	1	1	0	0	1	0	1	not applicable	1	6
Alshukairi	2021	17026	80	0	1	1	1	not applicable	0	1	0	1	not applicable	0	5
Antúnez-Montes	2021	12731	181	0	1	1	1	0	0	1	0	1	not applicable	1	6
Anugulruengkitt	2021	12143	68	0	1	1	1	1	0	1	0	1	not applicable	1	7

Arslan	2021	12043	60	1	1	1	1	1	0	1	0	1	not applicable	1	8
Asseri	2021	11993	93	0	1	1	1	0	0	1	0	1	not applicable	1	6
Ayed	2020	20144	85	1	1	1	1	0	0	1	0	1	not applicable	1	7
Bai	2021	14146	34	1	1	1	1	1	1	1	0	1	not applicable	1	9
Bandi	2020	10899	197	0	1	1	1	1	0	1	0	1	not applicable	1	7
Barrera	2021	18317	185	0	1	1	1	0	0	1	0	1	not applicable	1	6
Baumgarte	2022	15768	148	1	1	1	0	0	0	1	not applicable	not applicable	not applicable	1	5
Bayasheva	2021	13036	240	0	1	1	1	1	0	1	0	1	not applicable	1	7
Bellino	2020	12845	90	1	1	1	1	1	0	1	0	0	0	1	7
Berksoy	2021	11967	1	0	1	1	1	0	0	1	0	1	not applicable	1	6
Besli	2021	12938	58	1	1	1	1	0	0	1	1	1	not applicable	1	8
Biko	2020	15039	29	0	1	1	1	0	1	1	0	1	not applicable	1	7
Bolanos-Almeida	2021	11943	194	0	1	1	1	0	0	1	0	1	not applicable	1	6
Buonsenso	2021		229	0	1	1	0	0	0	1	1	1	not applicable	1	6
Buta	2022	12204	179	0	1	1	1	0	0	1	0	1	not applicable	1	6
Capozza	2021	17455	72	1	1	1	1	0	0	1	0	0	not applicable	1	6
Carrasco	2021	18681	30	1	1	1	0	0	1	1	1	0	0	1	7
Celik	2021	20080	53	0	1	1	1	0	0	1	0	1	not applicable	1	6
Cheng	2022	12127	95	0	1	1	1	1	1	1	0	1	not applicable	1	8
Chiang	2021	15615	17	1	1	1	1	1	1	1	0	1	not applicable	1	9
Chopra	2021	10777	10	1	0	1	1	1	1	1	0	0	0	1	7
Chowdhoury	2021	17481	169	0	1	1	0	0	0	1	0	0	not applicable	1	4
Chua	2021	12010	144	1	1	1	0	0	0	1	0	1	not applicable	1	6
Chua	2020		254	1	1	1	1	0	0	1	1	1	not applicable	1	8

Ciofi Degli Atti	2020	14451	137	1	1	1	1	0	0	1	1	1	not applicable	1	8
Cloete	2022	17094	139	0	1	1	1	0	0	0	0	0	0	1	4
Cohen	2021	11196	207	1	1	1	1	1	0	1	0	1	not applicable	1	8
Cooper	2021	18590	101	1	1	1	1	0	0	1	not applicable	not applicable	not applicable	1	6
Corso	2021	18622	173	0	1	1	1	0	0	1	0	1	not applicable	1	6
Dash	2021	12906	193	0	1	1	0	0	0	1	0	1	not applicable	1	5
Delahoy	2021	14905	235	1	0	1	0	0	0	1	1	1	not applicable	1	6
deLusignan	2020	18417	63	0	1	1	1	1	0	1	not applicable	not applicable	not applicable	1	6
Dilber	2021	16765	154	1	1	1	0	0	0	1	0	1	not applicable	1	6
Dominguez-Rodriguez	2021	11258	147	0	1	1	1	1	0	1	0	1	not applicable	1	7
Dong	2020	14191	239	1	1	1	1	0	0	1	0	1	not applicable	1	7
Du	2021	12015	125	0	1	1	1	0	0	1	1	1	not applicable	1	7
Elghoudi	2020	12898	153	1	1	1	1	0	0	0	0	1	not applicable	1	6
Ennab	2021	12021	11	1	1	1	1	0	0	1	1	1	not applicable	1	8
Ergenc	2021	15528	111	0	1	1	0	1	1	1	0	1	not applicable	1	7
Erturk	2021	16095	170	1	1	1	0	0	0	1	0	1	not applicable	1	6
Foster	2020	19329	51	0	1	1	1	1	0	1	0	1	not applicable	1	7
Funk	2022	17042	213	0	1	1	1	1	0	1	1	0	not applicable	1	7
Gaborieau	2020	14176	24	1	1	1	1	0	1	1	0	1	not applicable	1	8
Gampel	2020	12844	38	0	1	1	1	1	0	1	0	1	not applicable	1	7
Garazzino	2020	16369	129	0	1	1	1	0	0	1	0	1	not applicable	1	6
Gavriliu	2021	11758	67	0	1	1	1	1	0	1	0	1	not applicable	1	7
Ghosh	2020	12220	2	0	1	1	1	0	0	1	0	1	not applicable	1	6
Goktug	2021	14321	46	1	1	1	1	1	1	0	0	1	not applicable	1	8

Gomes	2021		237	1	1	1	1	1	0	1	1	1	not applicable	1	9
Gotzinger	2020	12888	180	0	1	1	1	0	0	1	1	1	not applicable	1	7
Gumus	2021	12032	171	0	1	1	0	0	0	0	0	1	not applicable	1	4
Guzman	2021	10914	91	0	1	1	1	1	0	1	0	1	not applicable	1	7
Haeusler	2021			1	1	1	1	0	0	1	1	1	not applicable	1	8
Hedberg	2022	12147	102	1	1	1	1	0	1	1	1	0	1	1	9
Hendler	2021	18426	191	1	1	1	1	1	0	1	0	1	not applicable	1	8
Hijazi	2021	17005	92	0	1	1	1	0	0	0	1	1	not applicable	1	6
Hobbs	2020	14625	200	1	1	1	1	0	0	1	0	1	not applicable	1	7
Howard	2021	14562	159	0	1	1	1	1	0	0	1	1	not applicable	1	7
Huete-Perez	2021	17773	162	1	1	1	1	0	0	1	1	0	0	1	7
Ibrahim	2021	11754	105	0	1	1	1	0	0	1	0	1	not applicable	1	6
Ibrahim	2020		230	0	1	1	1	0	0	1	0	1	not applicable	1	6
Indiriyani	2021	11703	116	1	1	1	1	0	0	0	0	1	not applicable	1	6
Isoldi	2021	12383	25	1	1	1	1	0	1	1	1	0	0	1	8
Jang	2022	14149	205	1	1	1	0	0	0	1	0	1	not applicable	1	6
Ji	2021	11737	99	0	1	1	1	0	0	1	0	1	not applicable	1	6
Jimenez-García	2021	17583	54	1	1	1	1	1	0	1	0	1	not applicable	1	8
Kaba	2021	14392	64	1	1	1	1	0	0	1	0	1	not applicable	1	7
Kaliyan	2021	11962	201	0	1	1	1	0	0	1	0	1	not applicable	1	6
Kanthimathinathan	2020	13359	7	0	1	1	1	not applicable	1	1	0	1	not applicable	1	7
Kapoor	2021	15108	178	0	1	1	1	0	0	1	0	1	not applicable	1	6
Kara	2021	14331	33	0	1	1	1	1	1	1	0	1	not applicable	1	8
Karaaslan	2021	12245	79	0	1	1	0	0	0	1	0	0	not applicable	1	4

Karaci	2021	19023	73	0	1	1	1	1	0	1	0	1	not applicable	1	7
Karbuz	2021	14170	55	0	1	1	1	1	0	1	0	0	0	1	6
Kavanagh	2021		75	1	1	1	0	0	0	1	1	0	0	1	6
Kepenekli	2022	12840	28	0	1	1	1	0	0	1	0	1	not applicable	1	6
Kim	2020	14904	225	0	1	1	1	0	0	1	1	0	0	1	6
Krithika	2021	16921	161	0	1	1	1	0	0	0	0	1	not applicable	1	5
Kuchar	2021	11883	32	1	1	1	0	0	0	1	0	1	not applicable	1	6
Kuczborska	2021	17757	78	0	1	1	1	0	1	1	0	0	0	1	6
Kufa	2022	14210	142	0	1	1	1	0	0	1	0	0	not applicable	1	5
Kumar	2021	17040	124	1	1	1	1	1	0	1	1	0	0	1	8
Kushner	2021	14601	175	1	1	1	1	0	1	1	0	1	not applicable	1	8
Ladhani	2021	18693	232	1	1	1	1	1	0	1	1	0	1	1	9
Lazzarini	2021	11715	132	0	1	1	1	1	0	1	0	1	not applicable	1	7
Lee	2022	10983	157	0	1	1	1	0	0	1	1	1	not applicable	1	7
Leidman	2021	12357	228	1	1	1	1	1	0	1	0	0	0	1	7
Liu	2020	13612	146	0	1	1	0	0	0	1	0	1	not applicable	1	5
Liu	2020	18392	143	1	1	1	0	0	0	1	not applicable	1	not applicable	1	6
Loconsole	2020	18661	42	0	1	1	1	0	0	1	0	1	not applicable	1	6
**Lopez-Aguilar**	2020	11887	184	1	1	1	1	0	0	1	0	1	not applicable	1	7
Lorenzo	2021	13694	121	1	1	1	1	0	0	1	0	1	not applicable	1	7
Lu	2020	18653	103	1	1	1	1	0	0	1	0	1	not applicable	1	7
Lu	2020	19361	27	1	1	1	1	1	1	0	0	1	not applicable	1	8
Lynam	2021	16059	12	0	1	1	0	0	1	1	0	1	not applicable	1	6
Madani	2021	18165	192	0	1	1	1	1	0	1	0	1	not applicable	1	7
Maltezou	2020	11847	39	0	1	1	1	1	1	1	0	1	not applicable	1	8

Mania	2022	17584	134	0	1	1	1	1	0	0	0	1	not applicable	1	6
Mania	2021	19960	13	1	1	1	0	0	1	1	0	1	not applicable	1	7
Matteudi	2021	12041	41	0	1	1	0	0	0	0	1	0	0	1	4
Mele	2021	16052	57	1	1	1	1	1	0	1	0	1	not applicable	1	8
Messiah	2021	18300	127	1	0	1	1	1	0	1	0	1	not applicable	1	7
Meyer	2021	13238	59	1	1	1	1	1	0	1	not applicable	not applicable	not applicable	0	6
Michos	2021	18704	81	1	1	1	0	0	0	1	0	1	not applicable	1	6
Moreno-Noguez	2021	18395	202	0	1	1	1	1	0	1	0	1	not applicable	1	7
Murillo-Zamora	2020	17716	48	0	1	1	1	1	0	1	0	1	not applicable	1	7
Musa	2021	17759	130	1	1	1	1	0	0	0	0	1	not applicable	1	6
MveangNzoghe	2021	13825	163	0	1	1	1	0	0	1	0	1	not applicable	1	6
Navarro-Olivos	2021	13292	190	0	0	1	1	1	0	1	0	1	not applicable	1	6
Ng	2021	11947	70	1	1	1	1	0	0	1	0	1	not applicable	1	7
Nunziata	2020	14784	156	1	1	1	1	0	0	1	0	1	not applicable	1	7
Odeleye	2021	19294	69	1	1	1	0	0	0	1	not applicable	not applicable	not applicable	1	5
Okur	2021	12113	108	0	1	1	0	0	0	1	0	1	not applicable	1	5
Olivar-Lopez	2020	12169	21	0	1	1	1	1	1	1	0	1	not applicable	1	8
Oliveira	2021	12008	187	1	1	1	1	1	0	1	1	1	not applicable	1	9
Omrani	2021		106	0	1	1	1	1	0	1	1	1	not applicable	1	8
Ortiz-Pinto	2022	11835	182	0	1	1	1	0	0	1	1	1	not applicable	1	7
Osmanov	2022	18407	199	0	1	1	1	1	0	1	1	0	0	1	7
Otto	2020	14212	183	0	1	1	1	0	1	1	0	1	not applicable	1	7
Ozlu	2022	11364	97	0	1	1	1	0	0	1	0	1	not applicable	1	6
Paduano	2021	11716	168	1	1	1	1	0	0	1	0	1	not applicable	1	7

Pande	2021	12162	152	0	1	1	1	1	0	1	0	1	not applicable	1	7
Pandey	2020	15539	186	1	1	1	0	0	0	1	0	1	not applicable	1	6
Parambil	2021	12915	77	1	1	1	1	0	0	1	1	1	not applicable	1	8
Parcha	2021	18316	211	0	1	1	1	1	0	1	0	1	not applicable	1	7
Parri	2020	12864	114	0	1	1	0	0	0	1	0	0	0	1	4
Peng	2021	17051	52	0	1	1	1	0	0	0	0	1	not applicable	1	5
Pokorska-Spiewak	2021	11945	128	0	1	1	1	0	0	1	0	1	not applicable	1	6
Pokorska-Spiewak	2021	12338	56	1	1	1	1	1	0	1	0	1	not applicable	1	8
Pudjiadi	2021	17273	209	0	1	1	0	0	0	1	0	1	not applicable	1	5
Qian	2021	18170	120	0	1	1	1	1	0	1	0	1	not applicable	1	7
Rabha	2021	12124	172	0	1	1	1	0	0	1	0	1	not applicable	1	6
Rabha	2021	11176	210	0	1	1	1	1	0	1	0	1	not applicable	1	7
Reis	2021	13983	242	0	1	1	1	1	0	1	1	1	1	1	9
Rha	2020	18898	4	1	1	1	0	0	0	1	0	1	not applicable	1	6
Rivas-Ruiz	2020	14463	189	0	1	1	1	1	0	1	0	1	not applicable	1	7
Rodriguez Velasquez	2021	19524	195	0	1	1	0	0	0	1	0	0	0	1	4
Rose	2021	18260	96	1	1	1	0	0	0	1	not applicable	not applicable	not applicable	0	4
Salako	2021	17768	160	0	1	1	1	1	0	1	0	1	not applicable	1	7
Saleh	2021	18966	82	1	1	1	1	0	0	1	0	1	not applicable	1	7
Sananez	2021	17604	118	0	1	1	1	1	0	1	0	1	not applicable	1	7
Schneider	2021	15025	122	0	1	1	1	1	0	1	0	0	0	1	6
Sedighi	2021	16363	115	0	1	1	1	0	0	1	0	1	not applicable	1	6
See	2021	11948	104	0	1	1	1	1	0	1	1	1	0	1	8
Sena	2021	11996	198	0	1	1	1	1	0	1	0	1	not applicable	1	7

Shahid	2021	12084	50	0	1	1	1	1	0	1	0	1	not applicable	1	7
Shapiro Ben David	2021	12894	131	0	1	1	1	0	0	1	1	1	not applicable	1	7
Sharma	2020	14140	3	0	1	1	1	0	0	1	1	1	not applicable	1	7
Sharma	2021	17720	6	1	0	1	1	1	1	1	1	1	not applicable	1	9
Shi	2022		238	1	1	1	1	1	0	1	1	0	1	1	9
Shoji	2022	12357	252	0	0	1	1	1	0	1	0	1	not applicable	1	6
Shoji	2021	12033	158	0	0	1	1	0	0	1	0	1	not applicable	1	5
Singh	2022	12163	166	1	1	1	1	0	0	1	0	1	not applicable	1	7
Somekh	2021	11753	241	0	1	1	0	0	0	1	0	0	0	1	4
Song	2021	12277	165	1	1	1	0	0	0	1	0	1	not applicable	1	6
Soriano-Arandes	2021	14919	136	0	0	1	1	1	0	1	0	1	not applicable	1	6
Sousa	2021	16835	167	0	1	1	1	1	0	1	0	1	not applicable	1	7
Stokes	2020	12523	226	0	1	1	1	1	0	1	0	1	not applicable	1	7
Talarico	2021	14125	44	1	1	1	0	0	0	0	0	1	not applicable	1	5
Tang	2021	12080	245	0	1	1	1	0	0	1	1	0	not applicable	1	6
Tosif	2021	11710	16	0	1	1	1	0	0	1	0	1	not applicable	1	6
Uka	2021	14465	164	0	1	1	1	0	0	1	0	1	not applicable	1	6
Ustundag	2021	12974	40	0	1	1	1	1	0	1	0	1	not applicable	1	7
van der Zalm	2021		138	0	1	1	0	1	0	1	0	1	not applicable	1	6
Verd	2022	13788	76	1	1	1	1	0	0	1	0	1	not applicable	1	7
Vergine	2020	14883	5	0	1	1	1	0	0	1	0	1	not applicable	1	6
Vogel	2022	18725	188	1	1	1	0	0	0	1	1	1	0	1	7
Wang	2020	11261	8	1	1	1	0	0	1	1	0	1	not applicable	1	7
Wang	2020	18807	9	1	1	1	0	0	1	1	1	1	not applicable	1	8

Wanga	2021	11691	227	0	0	1	1	1	0	1	1	0	0	1	6
Ward	2022	18408	203	0	1	1	1	1	0	1	1	1	not applicable	1	8
Wecławek-Tompol	2021	12998	74	0	0	1	1	1	0	1	1	1	not applicable	1	7
Wong	2022	17266	126	1	0	1	1	1	0	1	0	1	not applicable	1	7
Wong-Chew RM	2021	11997		0	0	1	1	1	1	1	1	1	0	1	8
Xiong	2020	12319	14	1	1	1	0	0	1	1	0	1	not applicable	1	7
Yayla	2020	11696	18	0	1	1	1	1	0	1	0	1	not applicable	1	7
Yilmaz	2022	14312	145	1	1	1	1	0	0	0	0	1	not applicable	1	6
Yilmaz	2020	14344	31	0	1	1	1	0	1	1	0	1	not applicable	1	7
Yonker	2020	14703	36	1	1	1	1	1	0	1	0	not applicable	not applicable	1	7
Yoon	2021	11820	100	1	1	1	0	0	0	1	not applicable	not applicable	not applicable	1	5

Cross-sectional studies

**Author name**	**Published year**	**Covidence number**	**Study ID**	**Were the criteria for inclusion in the sample clearly defined?**	**Were the study subjects and the setting described in detail?**	**Was the exposure measured in a valid and reliable way?**	**Were objective, standard criteria used for measurement of the condition?**	**Were confounding factors identified?**	**Were strategies to deal with confounding factors stated?**	**Were the outcomes measured in a valid and reliable way?**	**Was appropriate statistical analysis used?**	**Total score**
Abo	2021	13198	110	1	1	1	1	0	0	1	1	6
Alqayoudhi	2021	18474	22	1	1	1	1	0	0	1	1	6
Aprea	2021	17010	150	1	1	1	1	1	0	1	1	7
Arellano-Llamas, AA	2020	20057		1	1	1	1	0	0	1	1	6
Armocida	2022	12209	61	1	1	0	1	1	1	1	1	7
Brotons	2021	17624	107	1	1	1	1	1	1	1	1	8
Capra	2021	14391	151	1	1	1	1	1	0	1	1	7
Cofre, F	2020	12132		1	1	1	1	0	0	1	1	6
Colson	2021	11841	119	0	1	1	1	0	0	1	1	5

Colson	2020	11841	141	0	1	1	1	0	0	1	1	5
Dawood	2022	15547	149	1	1	1	1	1	0	1	1	7
Dominguez Rojas, J	2021	13432		1	1	1	1	1	0	1	1	7
Du	2021	1111	107	0	1	1	1	0	0	1	1	5
Engels	2022		35	1	1	1	1	0	0	1	1	7
Ferraro, D	2021	14144		1	1	1	1	0	0	1	1	6
Galli	2021	19930	109	1	1	1	1	0	0	1	0	5
Gudbjartsson	2020	19181	231	1	1	1	1	0	0	1	1	6
Imamura	2021	18509	177	1	0	1	1	0	1	1	1	6
Jiang	2022	1111	104	0	1	1	1	0	0	1	1	5
Levy	2020	11659	206	0	1	1	1	0	0	1	1	5
Lindsay	2021	14462	233	0	1	0	1	1	1	1	1	6
Morban DAH	2021	11954		1	1	1	1	1	0	1	1	7
Okonkwo	2020			1	1	1	1	0	0	1	1	6
Otto	2021		255	1	1	1	1	1	1	1	1	8
Peaper	2021	18904	224	0	1	1	1	1	0	1	1	6
Perramon	2021	18798	135	0	1	1	1	0	0	1	1	5
Rizzo	2021	18779	117	1	1	1	1	1	0	1	0	6
**Shayganmehr**	2021		253	1	1	1	1	0	0	1	1	6
Sola	2021	17820	196	1	0	1	1	0	0	1	1	5

Case-control studies

Author name	Published year	Covidence number	Study ID	Were the groups comparable other than the presence of disease in cases or the absence of disease in controls?	Were cases and controls matched appropriately?	Were the same criteria used for identification of cases and controls?	Was exposure measured in a standard, valid and reliable way?	Was exposure measured in the same way for cases and controls?	Were confounding factors identified?	Were strategies to deal with confounding factors stated?	Were outcomes assessed in a standard, valid and reliable way for cases and controls?	Was the exposure period of interest long enough to be meaningful?	Was appropriate statistical analysis used?	Total score
Calvani	2021	18120	113	1	1	1	0	1	0	0	1	not applicable	1	6
Devrim	2022	12361	20	1	0	1	1	1	0	0	1	not applicable	1	6
Elif	2021	19860	26	1	0	1	1	1	1	0	1	not applicable	0	6
Gujar	2021	11470	89	0	0	1	1	1	1	1	1	0	1	7
Hernandez-Garduno	2020	12292	208	0	0	1	1	1	1	1	1	not applicable	1	7
Meyer	2022	16311	88	1	1	1	1	1	0	0	1	not applicable	1	7
Olson	2022	13982	244	0	0	1	1	1	1	0	0	1	1	6
Yilmaz	2021	13790	66	1	1	1	1	1	1	0	1	not applicable	1	8

Diagnostic test accuracy studies

**Author name**	**Published year**	**Covidence number**	**Study ID**	**Was a consecutive or random sample of patients enrolled?**	**Was a case control design avoided?**	**Did the study avoid inappropriate exclusions?**	**Were the index test results interpreted without knowledge of the results of the reference standard?**	**If a threshold was used was it pre-specified?**	**Is the reference standard likely to correctly classify the target condition?**	**Were the reference standard results interpreted without knowledge of the results of the index test?**	**Was there an appropriate interval between index test and reference standard?**	**Did all patients receive the same reference standard?**	**Were all patients included in the analysis?**	**Total score**
Ana Laura	2021	18847	94	1	1	0	0	not applicable	1	0	1	1	1	6
Eleftheriou	2021	18117	71	0	1	0	1	not applicable	1	1	1	1	1	7
Lanari	2021	14522	236	1	1	0	0	1	1	0	1	1	1	7
Ollier	2022	17910	98	0	1	0	0	not applicable	1	0	1	1	1	5
Sahni	2021	12348	84	0	1	0	0	not applicable	1	0	1	1	1	5

Quasi-experimental studies

Author name	Published year	Covidence number	Study ID	**Is it clear in the study what is the 'cause' and what is the 'effect' (i.e. there is no confusion about which variable comes first)?**	**Were the participants included in any comparisons similar?**	**Were the participants included in any comparisons receiving similar treatment/care, other than the exposure or intervention of interest?**	**Was there a control group**	**Were there multiple measurements of the outcome both pre and post the intervention/exposure?**	**Was follow up complete and if not, were differences between groups in terms of their follow up adequately described and analyzed?**	**Were the outcomes of participants included in any comparisons measured in the same way?**	**Were outcomes measured in a reliable way?**	**Was appropriate statistical analysis used?**	**Total score**
Forster	2022	14530	234	1	0	0	1	1	not applicable	1	1	1	6

Table S11: List of studies excluded during full text review with reasons for exclusion

Study Title	Author names	Year	Reason for exclusion
Critical paediatric COVID-19: varied presentations but good outcomes	Lanyon, N.; du Pre, P.; Thiruchelvam, T.; Ray, S.; Johnson, M.; Peters, M. J.	2021	abstract
SARS-CoV-2 infection in ambulatory and hospitalised Spanish children	de Ceano-Vivas, M.; Martin-Espin, I.; Del Rosal, T.; Bueno-Barriocanal, M.; Plata-Gallardo, M.; Ruiz-Dominguez, J. A.; Lopez-Lopez, R.; Molina-Gutierrez, M. A.; Bote-Gascon, P.; Gonzalez-Bertolin, I.; Garcia-Sanchez, P.; Martin-Sanchez, J.; de Miguel-Lavisier, B.; Sainz, T.; Baquero-Artigao, F.; Mendez-Echevarria, A.; Calvo, C.	2020	abstract
Hospitalization and case fatality in individuals with sickle cell disease and COVID-19 infection	Mucalo, L.; Brandow, A. M.; Mason, S. F.; Singh, A.; Taylor, B. W.; Woods, K. J.; Yusuf, F. I.; Panepinto, J.	2020	abstract
Factors associated with hospitalization in youths and young adults with type 1 diabetes and COVID-19 infection	Tallon, E.; Ebekozien, O.; Sanchez, J.; Staggs, V.; Ferro, D.; Shyu, C. R.; Clements, M.	2021	abstract
Implementation of Telephone Screening for Timely Diagnosis of SARS-Cov2 Infection in Children with Cancer in a Low-Middle Income Country	Aguiar, G.; Bastidas, D.; Ruiz, R.; Tamayo, G.; Martinez, A.; Ornelas, M.; Rivera-Gomez, R.; Aristizabal, P.	2021	abstract
Implications of incidence of COVID-19 at rush university hospital pediatric oncology clinic	Aguina, M.; Gliksberg, A.; Kent, P.	2021	abstract
Neither inflammatory bowel disease nor immunosuppressants are associated with an increased risk for severe COVID-19. An observational dutch cohort-study	Gilissen, L.; Heinen, S.; Rijpma, L.; Schoon, E. J.; Schreuder, R. M.; Wensing, A. M.; Van Der Ende, M.; Bloemen, J.; Stapelbroek, J.; Stronkhorst, A.	2021	abstract
Experience with COVID-19 in german paediatric rheumatology centres	Klein, A.; Windschall, D.; Emminger, W.; Berendes, R.; Kuemmerle-Deschner, J.; Trauzeddel, R.; Rietschel, C.; Kuhn, A.; Hufnagel, M.; Sailer-Hoeck, M.; Hospach, T.; Haller, M.; Mrusek, S.; Sengler, C.; Minden, K.; Horneff, G.	2021	abstract

**Study Title**	**Author names**	**Year**	**Reason for exclusion**
Evaluation of the negative predictive value of the SARS-CoV-2 PCR respiratory assays in asymptomatic children undergoing surgery	Rao, S.; Ambroggio, L.; Asturias, E. J.; Bajaj, L.; Corrado, M.; Inge, T.; Jung, S.; Morrissey, T.; Osborne, C. M.; Searns, J. B.; Whitney, G.; Dominguez, S.	2020	abstract
Impact of SARS-CoV-2 Delta Variant on the Spectrum of Pediatric COVID-19 Disease in Arkansas	Romero, Jose R.; Warden, Donald E.; Cima, Michael	2021	abstract
COVID 19 Infection in Children in Colombia, Experience from a Nationwide Network (CORONARED)	Mesa-Monsalve, Juan Gonzalo Tobar Ivan Felipe GutiÃ©rrez Diaz Alejandro Diaz Calle-Giraldo Juan Pablo Chaucanez-Bastidas Yamile K.; LÃ³pez-Cubillos, Juan Francisco Mendoza-Rosado Laura Sarmiento-Wilches Patrik Eliana Sosa-Ã• vila Luis M.; MejÃa-Rivera, Luis Fernando Rojas Hernandez Juan P.; Arango-ferreira, Catalina Hoyos-Orrego Ã• lvaro DarÃo Ortiz-MarÃn Diana Cristina Vivas-Trochez Rosalba Jaramillo-Arango Catalina Garces carlos Medina Eduardo LÃ³pez Vinasco-SÃ¡nchez Luis Gabriel Araque-MuÃ±oz Paula Londono-Ruiz Juan Pablo BeltrÃ¡n-Arroyave Claudia Patricia Hurtado-Palacios Isabel C.	2021	abstract
COVID-19 and the gastrointestinal tract; initial manifestations and liver inflammation in children	Bitar, R.; Alattas, B.; Rawat, D.; Azaz, A.; Miqdady, M.	2021	abstract
Covid-19 among Children with Cancer in Greece (2020): Results from the Nationwide Registry of Childhood Hematological Malignancies and Solid Tumors (NARECHEM-ST)	Kourti, M.; Michos, A.; Narechem-St, G.; Ntzani, E.; Themistocleous, M.; Petridou, E.	2021	abstract
COVID-19 pandemic highlights health disparities in latinos in San Diego	Quiroz, E.; Hermel, D. J.; Bagsic, S. R.; Costantini, C. L.; Mahindra, A.; Saven, A.; Gahvari, Z.; Nagler, E.	2020	abstract
Correlation Between SARS CoV 2 Viral Load and Clinical Evolution of Patients Under 15 Years of Age with COVID 19 in a General Hospital in the Province of Buenos Aires, Argentina	Brizuela, Martin GoÃ±i Sandra Cardama Georgina Sommese Leandro Farina Hernan	2021	abstract

Study Title	Author names	Year	Reason for exclusion
Investigating the clinical characteristics of paediatric COVID-19 in Cape Town, South Africa: Initial results from the University of Cape Town (UCT), Department of Paediatrics and Child Health , COVID-19 paediatric repository	Zuhlke, L.; Stander, R.; Joachim, A.; Aldersley, T.; Dawood, A.; Hendricks, C.; Soni, K.; Abrams, J.; Morrow, B.; Zar, H.; Webb, K.; Donald, K.	2021	abstract
Hematologic and hemostatic derangements in children with COVID-19: The Miami experience	Leeman, R.; Shoag, J.; Borchetta, M.; Mitchell, C.; Davis, J.; Corrales-Medina, F.	2021	abstract
Impact of Covid-19 on the Children with Cancer in 6 Pediatric Oncology Units of Pakistan-A Multi-Center Study	Raza, M.; Maqsood, S.; Rana, Z.; Hamid, H.; Yasmeen, N.; Rehman, M.; Iqbal, R.; Shamvil Ashraf, M.	2021	abstract
The impact of National Containment Measures on a Pediatric Italian regional Hub for COVID-19, an observational study	Crea, F.; Panfili, F. M.; Amodeo, M. E.; Fintini, D.; Rossi, F. P.; Trenta, I.; Menichella, A.; Ossella, C.; Deidda, A.; Lidano, R.; Macchiarulo, G.; Lambiase, C.; Barbieri, M. A.; Raponi, M.	2021	abstract
582. Risk Factors for Progression to Hospitalization in Adolescents Presenting with Mild or Moderate COVID-19	Dubois, Melanie Campbell Jeffrey Lamb Gabriella S.; Lamb, Gabriella S.; Nakamura, Mari M.	2021	abstract
Epidemiology and disease burden of hospitalized children with paediatric multisystem inflammatory syndrome temporally associated with SARS-CoV-2 infection in Canada: A canadian pediatric surveillance program national prospective study	El Tal, T.; Morin, M. P.; Morris, S.; Berard, R.; Farrar, D.; Kakkar, F.; Moore-Hepburn, C.; Haddad, E.; Scuccimarri, R.; Yeung, R.	2021	abstract
Covid-19 Infection in children with Cancer after the First Wave in Turkey: A Study of the Turkish Pediatric Oncology (TPOG) and Hematology (TPHD) Societies	Kebudi, R.; Kurucu, N.; Tugcu, D.; Eker, N.; Ince, D.; Tokuc, G.; Cecen, R. E.; Sevinir, B.; Vural, O.; Erdem, M.; Demirdag, T.; Koc, A.; Kara, B.; Uzel, H.; Tuncel, D.; Citak, C.; Kartal, I.; Canpolat, C.; Ozguven, A.; Elli, M.; Acipayam, C.; Toret, E.; Karakas, Z.; Turkkan, E.; Kocak, U.; Tufekci, O.; Bay Buyukkapu, S.; Orhan, M.; Albayrak, C.; Albayrak, D.; Sen, H.; Bicakci, Z.; Ozbek, N.; Somer, A.; Kara, A.	2021	abstract

**Study Title**	**Author names**	**Year**	**Reason for exclusion**
Universal screening for SARS-COV-2 in children undergoingsurgery: A multicenter report	Fazal, F. Z.; Lin, E. E.; Blumberg, T. J.; Adler, A. C.; Talwar, D.; Ellingsen, K.; Shah, A. S.	2021	abstract
O3â€…The UK paediatric liver transplant programme during the COVID-19 pandemic	Grammatikopoulos, Tassos Taylor Rhiannon Whitney Julie Hartley Jane Attia Magdy Mirza Darius Rajwal Sanjay Watson Sarah Isaac John Manas Derek Heaton Nigel Dhawan Anil Forsythe John Thorburn Douglas	2021	abstract
Diagnostic accuracy of presenting symptoms in predicting SARS-CoV-2-positivity	Lim, Z. N.; Mills, R.; Van Geyzel, L.; Cummins, C.; Darren, A.; Jyothish, D.	2021	abstract
Mild COVID-19 disease among children with inflammatory bowel disease: An update from the secure-IBD registry	Brenner, E.; Zhang, X.; Ruemmele, F.; Turner, D.; Agrawal, M.; Ungaro, R.; Colombel, J. F.; Kappelman, M.	2021	abstract
Hospital mortality in patients with rare diseases during the COVID-19 and SARS pandemics: results from a 7.5 million population	Yan Chung, Claudia Ching Wilfred Hing Sang Wong Brian Hon Yin Chung	2021	abstract
The incidence of COVID-19 among childrenwith rheumatic diseases receiving biological agents	Tsurikova, N.; Ligostaeva, E.; Avdeenko, V.; Kobzeva, N.; Tsiganok, I.; Skorobogatova, K.; Motkina, A.	2021	abstract
GCS-neuro-COVID: Neurologic manifestations and outcomes in children with and without comorbidities	Whited, A.; Robertson, C.; Yun, J.; Wainwright, M.; Roa, J.; Schober, M.; Fink, E.	2022	abstract
56 Factors Predicting Admission in Pediatric and Young Adults With COVID-19	Dhillon, R. S.; Weise, M.; Wrotniak, B.; Cross, K.	2021	abstract
Multisystem inflammatory syndrome in children: results of a multicenter Italian survey during SARS-COV2 pandemic	Zunica, F.; Della Paolera, S.; Giangreco, M.; La Torre, F.; Bracaglia, C.; Filocamo, G.; Montin, D.; Villani, A.; Cimaz, R.; Ravelli, A.; Cattalini, M.; Taddio, A.	2021	abstract
History of Pain is Associated with Hospitalization and Severe Course of COVID-19 in Children with Sickle Cell Disease	Mucalo, L.; Brandow, A.; Dasgupta, M.; Mason, S.; Simpson, P.; Singh, A.; Taylor, B.; Woods, K.; Yusuf, F.; Panepinto, J.	2021	abstract

**Study Title**	**Author names**	**Year**	**Reason for exclusion**
Description of moderate-severe cases of covid-19 in pediatric cancer at the unidad nacional de oncologia pediatrica, Guatemala	Escobar, S. M. G.; Herrera, T. B. V.; Rosado, R. E.; Cojulun, A. C.; Toledo, M. A. M.; Klussmann, F. A.	2021	abstract
SARS-COV-2 infection in children; an analysis of two distinct clinical phenotypes caused by the same virus	Cantor, A.; Perez, A.; Miller, J.; Margolis, K. G.; Rudolph, B.; Kogan-Liberman, D.; DaSilva, B.; Gao, Q.; Ovchinsky, N.; Martinez, M.	2020	abstract
Investigating correlations between risk factors and outcomes in patients with COVID-19 in the PICU	Sachdeva, R.; Rice, T.; Soto-Campos, G.; Palumbo, J.; Wetzel, R.	2021	abstract
Impact of the COVID-19 pandemic on pediatric inpatient servicesin a new york city community hospital	Assudani, N.; Christian, L. N.; Adebayo, A.; Pierre, L.; Kondamudi, N. P.	2021	abstract
Acute liver injury in COVID-19: Risk factors in a large pediatric cohort	Perez, A.; Cantor, A.; Miller, J.; Kogan-Liberman, D.; Rudolph, B.; Margolis, K. G.; Gao, Q.; DaSilva, B.; Martinez, M.; Ovchinsky, N.	2020	abstract
SARS-CoV-2 in pediatric patients with pulmonary disorders: A single center experience	Johnson, B.; Bhandari, A.; Planet, P. J.; Cheng, P.	2021	abstract
SARS-CoV-2 diagnosis and point prevalence in a non-cohorted tertiary care center	Schrank, S.; McAleese, K.; Spence, A. B.; Natarajan, M.; Timpone, J.; Chang, J.; Balba, G. P.; Kumar, P.	2020	abstract
The Impact of COVID-19 in Children Post Hematopoietic Stem Cell Transplantation: Experiencefrom a Pediatric Transplant Unit in India	Varla, H.; Kumarmeena, S.; Rumeshchandar,; Vellaichamyswaminathan, V.; Ramyauppuluri,; Indirajayakumar,; Raj, R.	2021	abstract
Pediatric obstructive sleep apnea (OSA) and COVID-19-related adverse clinical outcomes	Shah, V.; Foldvary-Schaefer, N.; Wang, L.; Jehi, L.; Obrea, C. P.; Milinovich, A.; Mehra, R.	2021	abstract
Clinical course of COVID-19 in children with rheumatic disease under biologic therapy from Turkey	Sozeri, B.; Ulu, K.; Kaya, U.; Haslak, F.; Kisaarslan, A. P.; Otar Yener, G.; Baba, O.; Altug Gucenmez, O.; Sahin, N.; Baglan, E.; Sonmez, H. E.; Cakmak, F.; Ozturk, K.; Yildirim, D. G.; Sener, S.; Barut, K.; Batu, E. D.; Yildiz, M.; Basaran, O.; Adrovic, A.; Sahin, S.; Ozdel, S.; Bilginer, Y.; Poyrazoglu, H.; Coskuner, T.; Caglayan, S.;	2021	abstract

Study Title	Author names	Year	Reason for exclusion
	Demir, F.; Yuksel, S.; Kalyoncu, M.; Kasapcopur, O.; Ozen, S.; Aktay Ayaz, N.		
SARS-COV-2 Versus other viral respiratory infection: A comparative-analytical study of clinical spectrum in children	Saleh, M.	2021	abstract
Covid-19 Pediatric Patients in the United States; African American and Hispanic among Most Hospitalized Patients with Persistent Gi Manifestations	Ashktorab, Y.; Brim, A.; Pizuorno, A.; Nikdel, S.; Gayam, V.; Brim, H.	2021	abstract
Factors Predicting Admission in Pediatric and Young Adults With COVID-19	Dhillon, R. S.; Weise, M.; Wrotniak, B.; Cross, K.	2021	abstract
Short term outcomes in multisystem inflammatory syndrome in children (MIS-C) related to COVID-19	Riollano, M.; L. Marshall C; Kowalsky, S.; Tosi, M.; Posada, R.; Trachtman, R.; Paniz-Mondolfi, A.; Sordillo, E.	2020	abstract
Paediatric SARS-COV2 infections in Switzerland	Uka, A.; Zimmermann, P.; Ritz, N.	2021	abstract
Outcomes of COVID-19 infection among children and young people with pre-existing rheumatic and musculoskeletal diseases	Kearsley-Fleet, L.; Lawson-Tovey, S.; Costello, R. E.; Belot, A.; Aeschlimann, F.; Melki, I.; Kone-Paut, I.; Clemente, D.; Pinedo Gago, M. C.; Svestkova, N.; Vinsova, N.; Hamad Saied, M.; Berkun, Y.; Wulffraat, N.; Eulert, S.; Scire, C. A.; Strangfeld, A.; Mateus, E.; Machado, P.; Uziel, Y.; Hyrich, K.	2021	abstract
A Multi-center Study to Describe Obese Pediatric Patients with COVID-19 Across the United States	Hammoud, Roukaya Al Murphy James Del Bianco Gabriela P.; Heresi, Gloria Chang Michael L.	2021	abstract
Factors differentiating active COVID-19 infection from multisystem inflammatory syndrome in children	Gupta, N.; Talathi, S.	2022	abstract
Covid-19 in pediatric hematology and oncology patients in New York City	Gampel, B.; Troullioud Lucas, A.; Broglie, L.; Gartrell-Corrado, R.; Lee, M.; Levine, J.; Orjuela-Grimm, M.; Satwani, P.; Glade Bender, J.; Roberts, S.	2020	abstract

Study Title	Author names	Year	Reason for exclusion
Managing Covid-19 Amongst Pediatric Heart Transplant Recipients in Sao Paulo, Brazil	Azeka, E.; Arshad, A.; Jatene, M. B.	2020	abstract
COVID-19 infection in pediatric hematology oncology patients in Louisiana	Prasad, P.; LeBlanc, Z.; Velez, M.; LeBlanc, D.; Gardner, R.; Morrison, C.; Finger, L.; Charbonnet, V.	2021	abstract
COVID-19 infection among pediatric rheumatology patients: A single center experience	Kok, E.; Curry, M.; Ramirez, A.; Muscal, E.; DeGuzman, M.	2021	abstract
Juvenile idiopathic arthritis in the context of the coronavirus disease 19 pandemic: Impact on the decrease in treatment and the return to school	Quere, B.; Lemelle, I.; Lohse, A.; Pillet, P.; Molimard, J.; Richer, O.; Sordet, C.; Despert, V.; Rossi-Semerano, L.; Borocco, C.; Kone-Paut, I.; Gervais, E.; Guellec, D.; Devauchelle-Pensec, V.	2021	abstract
ALL-098: COVID-19 in Children with Cancer: What We Learned from the First Wave	Ebeid, F.; Adly, A.; Makkeyah, S.; Mostafa, S.; Ragab, I.	2021	abstract
13 Comorbid Conditions With COVID-19 in Hospitalized Pediatric Patients: A Multi-Center Analysis	Heyming, T.; Spiegelman, L.; Marano, R.; Taraman, S.; Feaster, W.; Keskinocak, P.; Ehwerhemuepha, L.	2021	abstract
Biochemical and cardiovascular predictors of PIMS risk in children after COVID-19 recovery -the results of the LATE COVID Kids study	Jatczak-Pawlik, I.; Lewek, J.; Czkwianianc, E.; Zeman, K.; Jankowski, P.; Banach, M.	2021	abstract
482. SARS-CoV-2 Prevalence in Feces of Very Young Children, A Longitudinal Study	Nashed, Lydia M.; Mani, Jyoti Hazrati Sahel Richards Tiana Nerikar Naya Ravi Shreeya Mattei Lisa Levy Shira Maxwell George Hourigan Suchitra	2021	abstract
Using the Educational Method B-Learning among Primary Caregivers to Reduce Sars-Cov-2 Infections in Children with Cancer in Limited Resource Setting	Escobedo-Melendez, G.; Gonzalez-Roldan, M.	2021	abstract
Unlocking pediatric oncology services during COVID lock down-difficult but not impossible	Sneha, D.; Scott, J.; Jayaraman, D.; Thiruvengadam Kothandan, B.; Arul, J.	2021	abstract

**Study Title**	**Author names**	**Year**	**Reason for exclusion**
Sociodemographic and clinical features of children and adolescents with SARS-CoV-2 infection in Nashville, Tennessee	Howard, Leigh Garguilo Kathryn Gillon Jessica Webber Steven Halasa Natasha B.; Banerjee, Ritu Banerjee Ritu	2020	abstract
A short audit of the impact of public health measures on respiratory viral presentations to our paediatric assessment unit during a winter of COVID-19	Jeremie, Juan	2021	abstract
Clinical presenting characteristics of pediatric COVID-19 infection in a tertiary care Children's Hospital in Detroit	Ang, J. Y.; Kannikeswaran, N.; Asmar, B.	2020	abstract
Clinical manifestations of COVID-19 and its impact on pediatric patients with rheumatic disease	Yi, B.; Mohandas, S.; Cidon, M.	2021	abstract
Clinical Course of Sars-Cov-2 in Children with Cancer in the South URAL Region	Kovalenko, S.; Spitchak, I.; Volkova, K.; Zub, N.; Solovieva, O.	2021	abstract
Admission rates for respiratory diseases during the covid-19 pandemic: The experience of the pediatric clinic in Pavia	Votto, M.; De Filippo, M.; Iozzi, L.; Castagnoli, R.; Manganelli, N. P.; De Amici, M.; Testa, G.; Licari, A.; Marseglia, G. L.	2021	abstract
A subset of children who have had COVID-19 and/or multisystem inflammatory syndrome in children (MIS-C) develop functional gastrointestinal disorders (FGID)	Qu, V.; Ringel, R.; Da Silva, B.; Pinker, E.; Margolis, K.; Khlevner, J.	2021	abstract
SARS-CoV-2 infection rates in children with cardiorespiratory disease	Du Berry, C.; Saunders, T.; Tosif, S.; Crawford, N.; Wurzel, D.	2021	abstract
Risk of Complications in Children With Adrenal Insufficiency and Covid-19	Raisingani, Manish Gope	2021	abstract
Risk Factors for Progression to Hospitalization in Adolescents Presenting with Mild or Moderate COVID-19	Dubois, Melanie Campbell Jeffrey Lamb Gabriella S.; Lamb, Gabriella S.; Nakamura, Mari M.	2021	abstract
Trend of critical bronchiolitis following easing of SARS-CoV-2 precautions	Cardenas, J.; Pringle, C.; Avery, L.	2022	abstract

Study Title	Author names	Year	Reason for exclusion
Pediatric ischemic stroke and severe acute respiratory syndrome Coronavirus 2	Beslow, L. A.; Linds, A. B.; Fox, C. K.; Kossorotoff, M.; Zuniga Zambrano, Y. C.; Hernandez-Chavez, M.; Hassanein, S. M. A.; Byrne, S.; Lim, M.; Maduaka, N.; Zafeiriou, D.; Dowling, M. M.; Felling, R. J.; Rafay, M. F.; Lehman, L. L.; Noetzel, M. J.; Bernard, T. J.; Dlamini, N.	2021	abstract
Vitamin D status and guidelines in Paediatric Inflammatory Multisystem Syndrome Temporarily associated with SARS-CoV-2 (PIMS-TS)	Brighouse, J. R.; Wan, M.; Duncan, E.; Handforth, J.; Kenny, J.; Mughal, M. Z.; Riphagen, S.; Theocharis, P.; Cheung, M. S.	2021	abstract
ERN-EuroBloodNet European Registry of Patients Affected by Red Blood Cell Disorders and COVID-19	Velasco, P.; Longo, F.; Piolatto, A.; Bardon-Cancho, E. J.; Ponce-Salas, B.; Flevari, P.; Voskaridou, E.; Biemond, B. J.; Nur, E.; Delaporta, P.; Besse-Hammer, T.; Ruiz-Llobet, A.; Raso, S.; Spasiano, A.; Guerzoni, M. E.; Beneitez-Pastor, D.; Dedeken, L.; Pepe, A.; Rosso, R.; Kunz, J. B.; de Montalembert, M.; Campisi, S.; Glenthoj, A.; Gonzalez Urdiales, P.; Benghiat, F. S.; Azerad, M. A.; Saunders, C. J.; Ferreira Faria, T.; Casini, T.; Bagnato, S.; Van de Velde, A.; Labarque, V.; Bertoni, E.; Van Damme, A.; Diamantidis, M. D.; Russo, R.; Stiakaki, E.; Quota, A.; Christou, S.; Teles, M. J.; Lafiatis, I.; Kerkhoffs, J. L.; Arguello Marina, M.; Lorite, M.; Rodriguez, A.; Iolascon, A.; Taher, A. T.; Colombatti, R.; Roy, N.; Manu Pereira, M. D. M.	2021	abstract
Asymptomatic pediatric COVID-19 positive patients captured through pre-operative screening program for ambulatory surgeries at a tertiary care hospital	Hilow, E.; Bryson, P. C.; Foster, C.; Markakis, D.; Kay, M.	2021	abstract
Are outcomes of living donor liver transplantation better during the COVID 19 pandemic	Menon, J.; Hakeem, A.; Rammohan, A.; Sundaramoorthy, S.; Kanagavelu, R.; Shanmugam, N.; Reddy, M. S.; Rela, M.	2021	abstract
Acute kidney injury and SARS-CoV-19 in children: Data from the national COVID cohort collaborative	Brennan, M.; Martin, S.; DeWitt, P.; Bennett, T.; Basu, R.; Dziorny, A.	2022	abstract

**Study Title**	**Author names**	**Year**	**Reason for exclusion**
SARS-CoV-2 Surveillance Testing Patterns among Hospitalized Pediatric Patients in a Single Academic Medical Center	Bukhari, Areej Seidelman Jessica Smith Becky A.; Lewis, Sarah S.; Smith, Michael J.; Moehring, Rebekah W.; Anderson, Deverick J.; Akinboyo, Ibukunoluwa	2021	abstract
Pediatric Inflammatory Multisystemic Syndrome in Brazil: Sociodemographic characteristics and risk factors to death	Da Silva Oliveira, V.; Soares, M. B.; De Morais, W. J.; Almeida, J. P.; Dias, L. A.; Barros, L. A. F.; De Aquino, E. C.; Pinto, R. M.	2021	abstract
Patterns of COVID-19 illness in hospitalized children	Nadiger, M.; Hassor, S.; Etinger, V.; Laufer, M.; Melnick, S.; Totapally, B.	2021	abstract
Outcome of Covid-19 among Pediatric Cancer Patients of a Tertiary Care Hospital in Pakistan	Raza, M.; Maqsood, S.; Syed, Y.; Muhammad, S.; Shamvil Ashraf, M.	2021	abstract
The magnitude of COVID-19 in children with rheumatic/ autoinflammatory diseases on immunomodulatory agents: A single center experience	Krepis, P.; Dasoula, F.; Papadaki, C.; Syggelou, A.; Maritsi, D. N.	2021	abstract
Longitudinal Plasma Cytokine Profiles Differentiating COVID-19 Severity Groups	Green, Amanda M.; Souquette, Aisha Agrawal Mona Wolf Joshua Wolf Joshua Gaur Aditya Allison Kim J.; Estepp, Jeremie Allen Emma Thomas Paul Smallwood Heather	2021	abstract
How common is COVID-19 in children?, young people and adults with rheumatic diseases results from the international covid-19 european patient registry	Shoop-Worrall, S.; Verstappen, S.; Costello, W.; Angevare, S.; Uziel, Y.; Wouters, C.; Wulffraat, N.; Beesley, R.	2021	abstract
Hematologic complications in covid-19: Study from Kerala	Das, S.; Mathew, S. K.; Mathew, R.	2020	abstract
Health perceptions and utilization in neurogastroenterology clinics during the COVID pandemic	Beinvogl, B.; Cohen, A.; Di Filippo, C.; Kane, M.; Nurko, S.; Rosen, R.	2021	abstract
Glycaemic control in T1D children and adolescents during Portuguese COVID-19 first lockdown: A family's survey report	Galhardo, J.; Dinis, I.; Limbert, C.	2021	abstract
Epidemiology of COVID-19 in children	Ali, A. K.	2021	abstract

Study Title	Author names	Year	Reason for exclusion
Severe COVID-19 in children and young adults in the Washington DC metropolitan region	DeBiasi, R. L.; Song, X.; Ansusinha, E.; Smith, K. L.; Bell, M.; Pershad, J.; Hahn, A.; Hamdy, R.; Hanisch, B.; Harik, N.; Jantausch, B. A.; Koay, A.; Campos, J. M.; Delaney, M.; Simpson, J.; Cora-Bramble, D.; Wessel, D.	2020	abstract
A comparison between epidemiological trend and curve of childbirth from women with covid infection in western Sicily: A cross-sectional study	Notarbartolo, V.; Marino, C.; Tumminello, M.; Di Pasquale, L.; Amato, L.; Giardina, F.	2021	abstract
Clinical course of COVID-19 in children with rheumatic disease under biologic therapy	Demir, F.; Ulu, K.; Caglayan, S.; Coskuner, T.; Sozeri, B.	2021	abstract
Children and COVID-19 in Colorado: The children's hospital Colorado experience	Graff, K. E.; Silveira, L.; Jarjour, J.; Curran-Hays, S.; Carpenter, L.; Pickard, K.; Mattiucci, M.; Smith-Anderson, C.; Abuogi, L.	2020	abstract
Chest imaging in COVID-19 infection in children: A diagnostic cross-sectional study	Bishay, M.; Gagen, R.; Lim, Z.; Van Geyzel, L.; Jyothish, D.; McGuirk, S.	2021	abstract
Characterization of pediatric oncology patients affected by COVID-19 using the SCCM virus registry	Balakumar, N.; Nadiger, M.; Keshavamurthy, P. R. S.; Totapally, B.; Bhalala, U. S.	2022	abstract
Characteristics Associated with SARS-CoV-2 Infection in Children	Curtis Sudbury, F.; Williams, Amanda Kwon Michelle Musser Leah Gavigan Patrick Ericson Jessica E.	2021	abstract
The COVID-19 pandemic and new onset pediatric type 1 diabetes at children's national hospital	Marks, B.; Khilnani, A.; Meyers, A.; Estrada, E.; Boughton, J.; Streisand, R.; Monaghan, M.	2021	abstract
COVID-19 is associated with increased mortality and acute hyperglycemic emergencies in patients with type 2 diabetes	Borella, N.; Wang, L.; Ross, J.; Stone, S.; Baldridge, M.	2021	abstract
Covid-19 Infection in Children with Cancer: Single-Center Experience from Turkey	Eker, N.; Tokuc, A. G.; Tufan, B.; Aras, S.	2021	abstract

**Study Title**	**Author names**	**Year**	**Reason for exclusion**
The Clinical Impact of Covid-19 on Cystic Fibrosis Patients in New York	Simonson, J.; Esposito, C.; Frantzen, T.; Henthorne, K.; Ramdeo, R.; Trentacoste, J.; Tsang, D.; La Vecchia, G.; Abdullah, R.; Berdella, M.; Bonitz, L.; Condos, R.; Constantinescu, A.; DeCelie-Germana, J.; Di Mango, E.; Giusti, R.; Keating, C.; Kier, C.; Lennox, A.; Sadeghi, H.; Walker, P.; Wang, J.	2021	abstract
Risk of Complications in Children With Type 1 Diabetes and Covid-19	Raisingani, Manish Gope	2021	abstract
COVID-19 in Children, Adolescents and Young Adults with Hematological Malignancies	Parker, R. S.; Malvar, J.; Doan, A.; Aguayo-Hiraldo, P.; Parekh, C.	2021	abstract
Epidemiological characteristics of 2143 pediatric patients with 2019 coronavirus disease in China: Dong Y, Mo X, Hu Y, et al. Pediatrics. 2020; doi: 10.1542/peds.2020-0702	Eastin, C.; Eastin, T.	2020	abstract
186: FACTORS DIFFERENTIATING ACTIVE COVID-19 INFECTION FROM MULTISYSTEM INFLAMMATORY SYNDROME IN CHILDREN	Gupta, Neha; Talathi, Saurabh	2022	abstract
Child mortality in the pandemic	Stoianova, S.	2021	abstract
Characteristics of new-onset paediatric Type 1 diabetes in the COVID-19 pandemic â€“ a multicentre perspective	Ponmani, Caroline Sakka Sophia Wickramarachchi Chandu Ajzensztejn Michal Shankar Kanumakala Redpath Yvette Hulse Tony Koshy Sherin	2021	abstract
Characteristics and outcomes of 728,047 children in the national COVID cohort collaborative (N3C)	Martin, B.; DeWitt, P.; Russell, S.; Dziorny, A.; Chute, C.; Haendel, M.; Moffitt, R.; Bennett, T.	2022	abstract
Burn care during six months covid-19 pandemic; Report of a single center	Aydogan, C.; Ayvazoglu Soy, E. H.; Turk, E.; Yabanoglu, H.; Avci, T.; Gedik, E.; Haberal, M.	2021	abstract
Association of elevated D-dimers with severity and outcome of COVID-19 infection in children	Saqlain, N.; Mazher, N.; Bari, A.; Ch, A.; Farhan, S.; Ahmed, N.	2021	abstract

Study Title	Author names	Year	Reason for exclusion
Respiratory findings in children post-COVID-19 infection	Leftin Dobkin, S. C.	2021	abstract
Characterization of chest-X-ray in young children and adolescents with PCR confirmed SARS-CoV-2 infection	Aguilar, H. M.; Molto, J.; Zember, J.; Sanchez-Jacob, R.; Diez, C.; Weinstock, J. J.; Xu Chen, X.; Kahanowitch, R.; Arroyo Morr, M. A.; Linguraru, M. G.; Nino, G. R.	2021	abstract
Predictors of COVID-19 symptom severity in NYC children: A prospective cohort study	Gourari, I.; Worgall, S.; Permaul, P.	2021	abstract
Effective strategies to combat COVID-19 pandemic in pediatric cancer care-a multi-institutional study from Pakistan	Rahil Khan, M.; Raza, M.; Jabbar, N.; Rana, Z. A.; Nuzhat, Y.; Faheem Ur Rehman, M.; Afia, A.; Shamvil Ashraf, M.	2020	abstract
Distribution of Respiratory Viral Pathogens in Infants Across Different Clinical Settings from December 2019 to April 2020	Haddadin, Zaid Rankin Danielle A.; lipworth, loren Fryzek Jon Suh Mina Shepard Donald S.; McHenry, Rendie Varjabedian Rebekkah Fernandez Kailee N.; Nelson, Christopher Halasa Natasha B.	2020	abstract
Children and adolescent patients with pre-existing type 1 diabetes and additional comorbidities have an increased risk of hospitalization from COVID-19; data from the T1D exchange COVID registry	Rompicherla, S.; Noor, N.; Edelen, R.; Gallagher, M. P.; Alonso, G. T.; Daniels, M.; Simmons, J.; Ebekozien, O.	2021	abstract
COVID-19 in children and young adults with sickle cell disease in the province of Quebec	Dakhallah, N.; Colaiacovo, M. L.; Castonguay, M.; Jimenez-Cortes, C.; Souza, A.; Ah-Yan, C.; Vincent, A. M.; Naessens, V.; Brossard, J.; Abish, S. S.; Santiago, R.; Soulieres, D.; Forte, S.; Pastore, Y.; Tran, T. H.	2021	abstract
Corona virus disease 2019 (Covid-19) incidence in pediatric oncology patient: Does routine screening affect the risk for the transmission in the hospital	Adrizain, R.; Sari, N.	2021	abstract
SARS-CoV-2 testing, infections, and hospital admissions with COVID-19 in children and young people in Scotland: a birth cohort study	Favarato, G.; Wijlaars, L.; Clemens, T.; Cunningham, S.; De Stavola, B.; Dibben, C.; Fenton, L.; Macfarlane, A.; McMenamin, J.; Milojevic, A.; Taylor, J.; Wood, R.; Hardelid, P.	2021	abstract

**Study Title**	**Author names**	**Year**	**Reason for exclusion**
SARS-CoV-2 surveillance in households with and without asthmatic/allergic children: The Human Epidemiology and Response to SARS-CoV-2 study (HEROS)	Seibold, M.; Moore, C.; Everman, J.; Williams, B.; Nolin, J.; Fairbanks-Mahnke, A.; Plender, E.; Patel, B.; Arbes, S.; Bacharier, L.; Bendixsen, C.; Calatroni, A.; Camargo, C.; Dupont, W.; Furuta, G.; Gebretsadik, T.; Gruchalla, R.; Gupta, R.; Hershey, G. K.; Murrison, L.; Jackson, D.; Johnson, C.; Kattan, M.; Liu, A.; Lussier, S.; O'Connor, G.; River-Spoljaric, K.; Phipatanakul, W.; Rothenberg, M.; Seroogy, C.; Teach, S.; Zoratti, E.; Togias, A.; Fulkerson, P.; Hartert, T.	2022	abstract
SARS-coV-2 infection in asthma/ recurrent wheezing-the reality of a pediatric hospital during 2020	Goncalves, T.; Lobato, M.; Pita, J. S.; Romeira, A. M.; Brito, M. J.; Matos, V.; Pinto, P. L.	2021	abstract
Profile and predictors of outcome in children with covid-19 disease: A prospective cohort study	Veena Sudeepthi, S.; Jindal, A.	2021	abstract
Prevalence and pattern of thromboembolic disease in pediatric patients with COVID-19	Royall, I.; Nguyen, H.; Sammer, M.	2021	abstract
Presentation and Outcome of Pediatric Multisystem Inflammatory Syndrome Temporally Associated with Sars-cov-2 Pandemic: An International Survey	Bautista, C.; Sanchez-De-Toledo, J.; Bradley, C. C.; Herberg, J.; Bajolle, F.; Randanne, P. C.; Salas-Mera, D.; Foldvari, S.; Chowdhury, D.; Bianco, F.; Singh, Y.; Levin, M.; Bonnet, D.; Fraisse, A.	2020	abstract
Potential predictors of requirement for mechanical ventilation in cases of COVID-19 related multisystem inflammatory syndrome in children (MIS-C): Results of a hospital-based cohort study from South India	Tiwari, A.; Balan, S.; Rauf, A.; Kappanayil, M.; Kesavan, S.; Sivadas, S.; Chickermane, P.; Vijayan, A.; Raj, M.; Anilkumar, V.; Sudhakar, A.	2021	abstract
Pediatric COVID-19: A report from viral infection and respiratory illness universal study (VIRUS)	Bhalala, U.; Gist, K.; Tripathi, S.; Chiotos, K.; Dapul, H.; Gharpure, V.; Bansal, V.; Kumar, V.; Boman, K.; Retford, L.; Kashyap, R.	2021	abstract
Outcomes of COVID-19 infection among children and young people with pre-	Kearsley-Fleet, L.; Lawson-Tovey, S.; Costello, R. E.; Belot, A.; Aeschlimann, F.; Melki, I.; Kone-Paut, I.; Eulert, S.; Svestkova, N.; Fingerhutova, S.; Clemente, D.; Berkun, Y.; Uziel, Y.; Wulffraat, N.	2021	abstract

Study Title	Author names	Year	Reason for exclusion
existing rheumatic and musculoskeletal diseases	M.; Raffeiner, B.; Oliveira-Ramos, F.; Dackhammar, C.; Strangfeld, A.; Mateua, E. F.; Machado, P. M.; Hyrich, K. L.		
Incidence and seasonality of respiratory viruses in pediatric admissions: What was the impact of school closure?	Oliveira, J.; Monteiro, S.; Salazar, L.; Souto, M.; Morais, L.; Ramos, A.; Ferreira-Magalhaes, M.	2021	abstract
RISK FACTORS FOR SEVERE COVID-19 ILLNESS IN CHILDREN: ANALYSIS OF THE VIRUS: COVID-19 REGISTRY	Tripathi, S.; Gist, K.; Chiotos, K.; Dapul, H.; Gharpure, V.; Bansal, V.; Kumar, V.; Boman, K.; Retford, L.; Kashyap, R.; Bhalala, U.	2021	abstract
Incidence and Characteristics of Type 1 and Type 2 Diabetes among Youths in the COVID-19 Pandemic	Nip, Angel Ahmad Tariq	2021	abstract
Impact of COVID-19 infection in patients with chronic intestinal failure. a national experience	Sayed, C.; Damas, V.; Charpentier, C.; Quilliot, D.; Piquet, M. A.; Dechelotte, P.; Billiauws, L.; Joly, F.	2020	abstract
Cardiac involvement in a paediatric cohort with COVID-19	Simoes, J. F.; Lemos, A. P.; Valsassina, R.; Garcia, A. M.; Silva, T. M.; Gouveia, C.; Laranjo, S.; Trigo, C.; Pinto, F.; Brito, M. J.	2021	abstract
Prevalence of SARS-CoV-2 positivity in pediatric surgical patients amid the first wave of the COVID-19 pandemic in New York City	Price, J. C.; Lee, J. J.; Ing, C.; Li, G.; Narula, J.; Clark, M. K.; Stylianos, S.; Whittington, R. A.; Levy, R. J.; Sun, L. S.	2022	abstract
Clinical characteristics of SARS-CoV-2 among confirmed and suspected cases in Tennessee	Talj, R.; Rankin, D. A.; Nicotera, J.; Howard, L.; Halasa, N. B.	2020	abstract
Clinical presentation of and differences between COVID-19 RT-PCR-positive and RT-PCR-negative pediatric patients	Saleh, M.	2021	abstract
Clinical characteristics of pediatric patients hospitalized with COVID-19 in an electronic health record database	Chomistek, A. K.; Doherty, M. C.; Gately, R. V.; Ogilvie, R. P.; Liang, C.; Seeger, J. D.; Wang, F.	2021	abstract
Clinical characteristics of infants with severe acute respiratory syndrome coronavirus 2 infection	Valentine, B.; Cooper, P.; Kouzoukas, B.; Ganbote, T.; Sreerama, P.; Jacobs, N.; Havalad, V.; Gharpure, V.	2022	abstract

**Study Title**	**Author names**	**Year**	**Reason for exclusion**
Children are at risk of COVID-19 infection: A profile from a UK Hospital	Khurram, M.; Chowdhury, A.	2021	abstract
Virtual pediatric systems: AKI in pediatric COVID-19 among north american intensive care units	Raina, R.; Chakraborty, R.; Singh, S. S.; Mahesh, S.	2021	abstract
The association between Covid-19 and obesity in children	Timofeeva, D.; Badretdinova, A.; Kamalova, A.; Sadykova, D.; Khusnutdinova, L.	2021	abstract
A pilot study of PCR community mass testing for COVID-19: who attends and who tests positive?	Levin, Kate Beatrix von Wissmann	2021	abstract
Clinical syndromes caused by covid-19 and a bayesian model to predict severity	Tagarro, A.; Dominguez-Rodriguez, S.; Villaverde, S.; Serna-Pascual, M.; Sanz-Santaeufemia, F. J.; Grasa, C.; Soriano-Arandes, A.; Saavedra-Lozano, J.; Fumado, V.; Epalza, C.; Alonso, J. A.; Rodriguez-Molino, P.; Pujol, J. M.; Moraleda, C.	2021	abstract
Comorbid Conditions With COVID-19 in Hospitalized Pediatric Patients: A Multi-Center Analysis	Heyming, T.; Spiegelman, L.; Marano, R.; Taraman, S.; Feaster, W.; Keskinocak, P.; Ehwerhemuepha, L.	2021	abstract
MIS-C is a risk factor for thrombotic events	McHugh, Jessica	2021	abstract
Preliminary assessment of acute kidney injury in critically ill children associated with SARS-CoV-2 infection: A multicenter cross-sectional analysis	Bjornstad, E. C.; Krallman, K. A.; Askenazi, D.; Zappitelli, M.; Goldstein, S. L.; Basu, R. K.	2021	abstract
Comparison of COVID-19 Infection in Children During the First and Second Wave	Krishnamurthy, S.; Kar, S. S.; Dhodapkar, R.; Parameswaran, N.	2022	abstract
Pediatric Hospitalizations for Respiratory Infections: Before and after SARS-CoV-2	Monteiro, S.; Salazar, L.; Oliveira, J.; Souto, M.; Morais, L.; Ramos, A.; Ferreira-Magalhaes, M.	2021	abstract
SARS-CoV-2 Infection in Hospitalized Children: An Elevated Body Mass Index is a Marker of Increased Risk of Acute Respiratory Failure	Foster, Catherine Kumar Shelley Tocco Elizabeth Holzmann-Pazgal Galit Campbell Judith R.; Marquez, Lucila Dutta Ankhi	2021	abstract

Study Title	Author names	Year	Reason for exclusion
Disease Severity and Clinical Manifestations of SARS-CoV-2 Infection Among Infants Over the First Year of the Pandemic in Canada	Piché-Renaud, Pierre-Philippe Panetta Luc Farrar Daniel Hepburn Charlotte Moore Drouin Olivier Kakkar Fatima Morris Shaun	2021	abstract
Racial-ethnic disparities in outcomes of children hospitalized for COVID-19: A virus registry report	Dapul, H.; Tripathi, S.; Kuehne, J.; Ramirez, M.; Rajagopalan, L.; Salameh, M.; Tolopka, T.; Garcia, M.; Boman, K.; Kumar, V.; Dreyer, B.; Bhalala, U. S.	2022	abstract
Incidence of spread of clinically relevant SARS-COV2 infection between children in a tertiary emergency department: An evaluation	Pandey, M.; Sisodia, S.; Bandi, S.; Roland, D.	2021	abstract
Low incidence of COVID-19 in children and adolescent post-liver transplant at a Latin American reference center	Tannuri, U.; Tannuri, A. C. A.; Cordon, M. N. A.; Miyatani, H. T.	2020	no primary data
Critically ill children in paediatric intensive care unit are no less susceptible to infectious diseases amid the COVID-19 pandemic	Leung, K. K. Y.; Hon, K. L.; Ip, P.; Chan, R. W. Y.	2021	no primary data
Care of Pediatric Patients with Diabetes During the Coronavirus Disease 2019 (COVID-19) Pandemic	Buggs-Saxton, C.	2021	no primary data
Letter to the Editor: THE IMPACT OF THE COVID-19 PANDEMIC ON SCHIZOPHRENIA PATIENTS	Hosgelen, E. I.; Alptekin, K.	2021	no primary data
COVID-19 Case Age Distribution: Correction for Differential Testing by Age	Fisman, D. N.; Greer, A. L.; Brankston, G.; Hillmer, M.; O'Brien, S. F.; Drews, S. J.; Tuite, A. R.	2021	no primary data
Deaths in children and young people in England after SARS-CoV-2 infection during the first pandemic year	Smith, C.; Odd, D.; Harwood, R.; Ward, J.; Linney, M.; Clark, M.; Hargreaves, D.; Ladhani, S. N.; Draper, E.; Davis, P. J.; Kenny, S. E.; Whittaker, E.; Luyt, K.; Viner, R.; Fraser, L. K.	2022	no primary data
Women and children first: The need for ringfencing during the COVID-19 pandemic	Grunebaum, A.; Dudenhausen, J.; McCullough, L. B.; Chervenak, F. A.	2020	no primary data

**Study Title**	**Author names**	**Year**	**Reason for exclusion**
COVID-19, Australia: Epidemiology Report 17 (Fortnightly reporting period ending 24 May 2020)	Covid- National Incident Room Surveillance, Team	2020	no primary data
How lethal is SARS-CoV-2 pneumonia when compared with respiratory syncytial virus and influenza in young children?	Wei, J. S.	2020	no primary data
Clinical and transmission dynamics characteristics of 406 children with Coronavirus disease 2019 in China: a review	Yang, ZhenDong; Zhou, GaoJun; Jin, RunMing; Liu, ZhiSheng; Dong, ZongQi; Xie, Xiong; Song, GuoWei	2020	no primary data
CaractÃ©ristiques des patients drÃ©panocytaires infectÃ©s par le SARS-CoV-2Â en fonction de leur prise en charge ambulatoire ou en hospitalisationÂ : Ã©tude nationale chez 536Â patients	Arlet, J. B.; De Luna, G.; Khimoud, D.; Cheminet, G.; Garou, A.; Cannas, G.; Cougoul, P.; Guitton, C.; Holvoet, L.; OdiÃ¨vre, M. H.; Joseph, L.; Santin, A.; De Montalembert, M.; Bartolucci, P.; Bernit, E.; Lionnet, F.	2021	non-English study
Epidemiological features of severe acute respiratory syndrome coronavirus 2 infection in children in Shijiazhuang, China: An analysis of 133 cases. [Chinese]	Su-Kun, L.; Bo, N.; Li-Li, J.; Jian-Hua, L.; Jin-Feng, S.	2021	non-English study
[Clinical features of asymptomatic or subclinical COVID-19 in children]	Liu, You-Jing; Chen, Peng; Liu, Zhi-Sheng; Li, Ying; Du, Hui; Xu, Jia-Li	2020	non-English study
Clinical features of asymptomatic or subclinical COVID-19 in children. [Chinese]	Liu, Y. J.; Chen, P.; Liu, Z. S.; Li, Y.; Du, H.; Xu, J. L.	2020	non-English study
Characteristics of hospitalizations in Canada of children with acute SARS-CoV-2 infection in 2020	Drouin, O.; Hepburn, C. M.; Farrar, D. S.; Baerg, K.; Chan, K.; Cyr, C.; Donner, E. J.; Embree, J. E.; Farrell, C.; Forgie, S.; Giroux, R.; Kang, K. T.; King, M.; Laffin, M.; Luu, T. M.; Orkin, J.; Papenburg, J.; Pound, C. M.; Price, V. E.; Purewal, R.; Sadarangani, M.; Salvadori, M. I.; Top, K. A.; Viel-Theriault, I.; Kakkar, F.; Morris, S. K.; pour l'equipe de l'etude sur la, Covid-du Programme canadien de surveillance pediatrique	2021	non-English study

Study Title	Author names	Year	Reason for exclusion
Epidemiological and clinical profile of children with coronavirus disease (Covid-19) at the center for the treatment of epidemics and infection prevention (cteip) of the university hospital of donka in conakry. [French]	Camara, E.; Barry, I. K.; Diallo, F. B.; Diallo, M. L.; Diop, M. M.; Cherif, M. S.; Diallo, I. S.; Kouyate, M.; Bangoura, M. A.; Barry, A.; Barry, M. C.; Ngadande, I. H.; Kaba, O.; Kolie, O. O.; Camara, D. D.; Diallo, S. B.; Dia, H.	2020	non-English study
First epidemic wave of SARS-CoV-2 in nouvelle-Aquitaine: descriptive analysis of control measures, care pathways and compliance with barrier measures, March-April 2020	Castor, C.; Gault, G.; Larrieu, S.; Evain, S.; Siguier, A.; Ramel, V.; Darigol, M. V.; Herteau, A.; Trouvain, K.; Filleul, L.	2022	non-English study
CaractÃ©ristiques des hospitalisations au Canada d'enfants ayant contractÃ© une infection aiguÃ« par le SRAS-CoV-2 en 2020	Drouin, Olivier; Hepburn, Charlotte Moore; Farrar, Daniel S.; Baerg, Krista; Chan, Kevin; Cyr, Claude; Donner, Elizabeth J.; Embree, Joanne E.; Farrell, Catherine; Forgie, Sarah; Giroux, Ryan; Kang, Kristopher T.; King, Melanie; Laffin, Melanie; Luu, Thuy Mai; Orkin, Julia; Papenburg, Jesse; Pound, Catherine M.; Price, Victoria E.; Purewal, Rupeena; Sadarangani, Manish; Salvadori, Marina I.; Top, Karina A.; Viel-ThÃ©riault, Isabelle; Kakkar, Fatima; Morris, Shaun K.	2021	non-English study
[Children and COVID-19-Data from mandatory reporting and results of contact person testing in daycare centers and schools in Frankfurt am Main, Germany, August-December 2020]	Heudorf, Ursel; Steul, Katrin; Walczok, Antoni; Gottschalk, Rene	2021	non-English study
[SARS-CoV-2 incidence, transmission, and containment measures in daycare centers during the COVID-19 pandemic-findings from the Corona Daycare Study]	Loss, Julika; Kuger, Susanne; Buchholz, Udo; Lehfeld, Ann-Sophie; Varnaccia, Gianni; Haas, Walter; Jordan, Susanne; Kalicki, Bernhard; Schienkiewitz, Anja; Rauschenbach, Thomas	2021	non-English study
[Children in the COVID-19 pandemic and the public health service (OGD) : Data and reflections from Frankfurt am Main, Germany]	Heudorf, U.; Gottschalk, R.; Walczok, A.; Tinnemann, P.; Steul, K.	2021	non-English study

Study Title	Author names	Year	Reason for exclusion
Prevalence of SARS-CoV-2 in children from a cohort of 2192 patients. [German]	Meyer, M.; Rubsteck, E.; Lehmann, C.; Klein, F.; Gruell, H.; Hunseler, C.; Weber, L. T.	2021	non-English study
[Challenges of the COVID-19 Pandemic for Schools in Mecklenburg-Vorpommern - First Results of a Prospective Case Study]	LÃ¼cker, Petra; Ehmke, Manja; Rabes, Anne; Emmerich, Anna-Sabina; KÃ¤stner, Anika; Reisinger, Emil C.; Hoffmann, Wolfgang; Sombetzki, Martina	2021	non-English study
[COVID-19 in obstetric anesthesia : Prospective surveillance of peripartum infections with SARS-CoV-2 and peripartum course of disease in affected women]	Sitter, Magdalena; Schlesinger, Tobias; Reinhold, Ann-Kristin; Scholler, Axel; von Heymann, Christian; Welfle, Sabine; Bartmann, Catharina; WÃ¶ckel, Achim; Kleinschmidt, Stefan; Schneider, Sven; Gottschalk, AndrÃ©; Greve, Susanne; Wermelt, Julius Z.; Wiener, Roland; Schulz, Frank; Chappell, Daniel; Brunner, Maya; Neumann, Claudia; Meybohm, Patrick; Kranke, Peter	2021	non-English study
COVID-19 pada anak dan langkah pencegahan yang perlu dilakukan di sekolah Kota Tanjungpinang tahun 2021	Martias, Indra	2021	non-English study
[The management of confirmed cases of Covid-19 infection in schools: the experience of the prevention Department of the Azienda Sanitaria Friuli occidentale in the Pordenone area]	Candela, G.; Del Bianco, F.; Lo Giudice, A.; Bolzonello, C.; Bomben, L.; Sumelli, C.; Biasotto, E.; Pilan, S.	2021	non-English study
Epidemiological and clinical characteristics of 5,628 patients with coronavirus disease 2019 in South Korea: A nationwide multicenter study	Lee, S. W.; Moon, S. Y.; Yon, D. K.	2021	non-English study
Multisystem inflammatory syndrome in children associated with covid-19: Results of a multicenter study. [Russian]	Novikova, Y. Yu; Ovsyannikov, D. Yu; Glazyrina, A. A.; Zvereva, N. N.; Peters, S. S.; Abdullaev, A. N.; Afukov, I. I.; Bogdan, P. I.; Vakhlova, I. V.; Gorev, V. V.; Degtyareva, E. A.; Zhdanova, O. I.; Zorina, M. A.; Kantemirova, M. G.; Karpenko, M. A.; Kolganova, N. I.; Kryshova, E. S.; Kurbanova, S. Kh; Nikolishin, A. N.; Pastukhov, P. A.; Petryaykina, E. E.; Rtishchev, A. Yu; Saifullin, M. A.; Sergeev, D. A.; Sokotova, T. V.; Kharkin, A. V.; Chagirev, V. N.; Shchederkina, I. O.	2021	non-English study

**Study Title**	**Author names**	**Year**	**Reason for exclusion**
Features of a new Soronavirus infection in children of different ages. [Russian]	Shakmaeva, M. A.; Chernova, T. M.; Timchenko, V. N.; Nachinkina, T. A.; Tetyushin, K. V.; Kaplina, T. A.; Subbotina, M. D.; Bulina, O. V.; Afanasyeva, O. I.	2021	non-English study
MULTISYSTEM INFLAMMATORY SYNDROME IN CHILDREN ASSOCIATED WITH COVID-19: RESULTS OF A MULTICENTER STUDY	Ð• Ð¾Ð²Ð¸ÐºÐ¾Ð²Ð°, Ð® Ð® ÐžÐ²Ñ• Ñ• Ð½Ð½Ð¸ÐºÐ¾Ð² Ð” Ð® Ð“Ð»Ð°Ð·Ñ‹Ñ€Ð¸Ð½Ð° Ð• Ð• Ð—Ð²ÐµÑ€ÐµÐ²Ð° Ð• Ð• ÐŸÐµÑ‚ÐµÑ€Ñ• Ð¡ Ð¡ Ð• Ð±Ð´ÑƒÐ»Ð»Ð°ÐµÐ² Ð• Ð• Ð• Ñ„ÑƒÐºÐ¾Ð² Ð˜ Ð˜ Ð‘Ð¾Ð³Ð´Ð°Ð½ ÐŸ Ð˜ Ð’Ð°Ñ…Ð»Ð¾Ð²Ð° Ð˜ Ð' Ð“Ð¾Ñ€ÐµÐ² Ð' Ð' Ð”ÐµÐ³Ñ‚Ñ• Ñ€ÐµÐ²Ð° Ð• Ð• Ð–Ð´Ð°Ð½Ð¾Ð²Ð° Ðž Ð˜ Ð— Ð¾Ñ€Ð¸Ð½Ð° Ðœ Ð• ÐšÐ°Ð½Ñ‚ÐµÐ¼Ð¸Ñ€Ð¾Ð²Ð° Ðœ Ð“ ÐšÐ°Ñ€Ð¿ÐµÐ½ÐºÐ¾ Ðœ Ð• ÐšÐ¾Ð»Ð³Ð°Ð½Ð¾Ð²Ð° Ð• Ð˜ ÐšÑ€Ñ‹ÑˆÐ¾Ð²Ð° Ð• Ð¡ ÐšÑƒÑ€Ð±Ð°Ð½Ð¾Ð²Ð° Ð¡ Ð¥ Ð• Ð¸ÐºÐ¾Ð»Ð¸ÑˆÐ¸Ð½ Ð• Ð• ÐŸÐ°Ñ• Ñ‚ÑƒÑ…Ð¾Ð² ÐŸ Ð•	2021	non-English study
Clinical features of the course of covid-19 in hospitalized children of various ages. [Russian]	Bychkova, S. V.; Malgina, G. B.; Plyusnina, N. N.; Volhin, E. V.; Dyakova, M. M.	2021	non-English study
Diagnostics of a new coronavirus infection in children admitted to a multidisciplinary hospital. [Russian]	Nikolaeva, S. V.; Feklisova, L. V.; Ponezheva Zh, B.; Belyaeva, T. Yu; Titova, T. V.; Khadisova, M. K.; Gorelov, A. V.	2021	non-English study
Soronavirus infection COVID-19 in children in the Russian federation. [Russian]	Gorelov, A. V.; Nikolaeva, S. V.; Akimkin, V. G.	2020	non-English study
Clinical characteristics of novel coronavirus infection and efficacy of intranasal interferon alpha in pregnant women with COVID-19 and in newborns with perinatal exposure. [Russian]	Romanovskaya, A. V.; Malyugina, T. N.; Mikhaylova, E. V.; Malinina, N. V.; Zheleznikov, P. A.; Chudakova, T. K.; Serdyukov, A. Y.; Raskina, E. E.; Burova, O. S.; Denisyuk, N. E.	2021	non-English study
Clinical and epidemiological features of the course of the new coronavirus infection COVID-19 in children during periods of an increase in the incidence in Moscow in 2020-2021. [Russian]	Mazankova, L. N.; Samitova, E. R.; Osmanov, I. M.; Afukov, I. I.; Dracheva, N. A.; Malakhov, A. B.; Gutyrchik, T. A.; Akimkin, V. G.; Ploskireva, A. A.; Taranov, L. O.	2021	non-English study

**Study Title**	**Author names**	**Year**	**Reason for exclusion**
COVID-19 in children with bronchial asthma: Clinical manifestations, course options, approaches to therapy. [Russian]	Geppe, N. A.; Kolosova, N. G.; Shakhnazarova, M. D.; Malakhov, A. B.; Timofeev Yu, S.; Samartseva, V. G.; Glazachev, O. S.; Grebeneva, I. V.; Romantseva, E. V.; Kalinovskaya, I. I.; Odinaeva, N. D.	2021	non-English study
New coronavirus infection (COVID-19) in children: Clinical, laboratory and diagnostic aspects. [Russian]	Nosyreva, S. Yu; Pankov, A. S.; Korneev, A. G.	2021	non-English study
Epidemic process of covid-19 in the russian federation: Interim results. 1th report. [Russian]	Pshenichnaya, N. Yu; Lizinfeld, I. A.; Zhuravlev, G. Yu; Ploskireva, A. A.; Akimkin, V. G.	2020	non-English study
Characteristics of CoVID-19 in children: The first experience in the hospital of st. Petersburg. [Russian]	Dondurey, E. A.; Isankina, L. N.; Afanasyeva, O. I.; Titeva, A. V.; Vishnevskaya, T. V.; Kondratev, V. A.; Gryaznova, I. A.; Berezina, M. V.; Zolotova, M. A.; Volzhanin, V. M.	2020	non-English study
Maternal and perinatal outcomes during Covid-19 pandemia in Far East and Siberian federal region of Russia	Đ‘Đ•Đ›ĐžĐšĐ Đ˜Đ• Đ˜Đ¦ĐšĐ• Đ¯, Đ¢ Đ• Đ• Đ Đ¢Đ«ĐœĐ£Đš Đ• Đ’ Đ¤Đ˜Đ›Đ˜ĐŸĐŸĐžĐ’ Đž Đ¡ Đ¤Đ ĐžĐ›ĐžĐ’Đ• Đ• Đ˜	2021	non-English study
Herd immunity to SARS-CoV-2 among the population in Saint-Petersburg during the COVID-19 epidemic	Popova, A. Yu Ezhlova E. B.; Melâ€™nikova, A. A.; Bashketova, N. S.; Fridman, R. K.; Lyalina, L. V.; Smirnov, V. S.; Chkhindzheriya, I. G.; Grechaninova, T. A.; Agapov, K. A.; Arsentâ€™eva, N. A.; Bazhenova, N. A.; Batsunov, O. K.; Danilova, E. M.; Zueva, E. V.; Komkova, D. V.; Kuznetsova, R. N.; Lyubimova, N. E.; Markova, A. N.; Khamitova, I. V.; Lomonosova, V. I.; Vetrov, V. V.; Milichkina, A. M.; Dedkov, V. G.; Totolyan, A. A.	2020	non-English study
Outbreaks of covid-19 in medical organizations	Sisin, Y. I.; Golubkova, A. A.; Kozlova, I. I.; Ostapenko, N. A.	2021	non-English study
Course of a new coronavirus infection in children: some aspects of monitoring and analysis of mortality	Uskov, A. N.; Lobzin, Yu V.; Rychkova, S. V.; Babachenko, I. V.; Fedorov, V. V.; Ulukhanova, L. U.; Pochinyaeva, L. M.	2020	non-English study
Predictors of death in severe new coronavirus infection in children	Aleksandrovich, Y. U. S.; Prometnoy, D. V.; Mironov, P. I.; Pshenisnov, K. V.; Anchutin, P. E.; Teplyakova, E. D.	2021	non-English study
COVID-19 in Russia. Spring-Summer Period of the Pandemic 2020	Karpova, L. S.; Lioznov, D. A.; Stolyarov, K. A.; Popovtseva, N. M.; Stolyarova, T. P.	2020	non-English study

Study Title	Author names	Year	Reason for exclusion
Clinical manifestations of new coronavirus infection (COVID-19) in children admitted to hospital	Sadykova, D. I.; Khaliullina, S. V.; Anokhin, V. A.; Ziatdinov, A. I.; Senek, S. A.; Samoylova, N. V.; Makarova, T. P.; Melnikova, Yu S.; Khusnutdinova, L. R.	2021	non-English study
Coronavirus infection (COVID-19) in children with cystic fibrosis	Kondratyeva, Elena Ivanovna Sherman V. D.; Zhekaite, E. K.; Simonova, O. I.; Gorinova, Y. V.; Boitsova, E. V.; Mukhina, M. A.; Kashirskaya, N. Yu Malakhov A. B.	2020	non-English study
Epidemic process of COVID-19 in the Russian Federation: interim results. 1th report	Pshenichnaya, N. Yu Lizinfeld Irina A.; Zhuravlev, Grigory Yu Ploskireva Antonina A.; Akimkin, Vasily G.	2020	non-English study
Characteristics of laboratory-confirmed positive cases in the first year of the COVID-19 pandemic in a university hospital	Ã‡iÃ§ek, C.; Soylu, M.; Ã–zarslan, M. A.; Mert, M.; BaÅŸkir, M. B.; SertÃ¶z, Åž R.; ZeytinoÄŸlu, A.; Karbek Akarca, F.; Ersel, M.; UlaÅŸ Saz, E.; TaÅŸbakan, M.; PullukÃ§u, H.; TaÅŸbakan, M. S.; ErsayoÄŸlu,; Iacute,; Ã‡ankayali,; Iacute,; AydoÄŸan, T. G.; Polat, F.; Aydin Uysal, A.; Akkul, B.; AkkuÅŸ, G.; Åžahin,; Iacute,; N,; Noyan, A.	2021	non-English study
Features of the coronavirus disease course in young children	Kosmynina, N. S.; Avramenko, I. Y.	2021	non-English study
Multisystem inflammatory syndrome in children: a cross-sectional study of cases and factors associated with deaths during the COVID-19 pandemic in Brazil, 2020	Relvas-Brandt, L. A.; Gava, C.; Camelo, F. S.; Porto, V. B. G.; Alves, R. F. S.; Costa, M. S. C. D.; Carvalho, S. M. D.; Carmo, G. M. I. D.; Fantinato, F. F. S. T.; Wada, M. Y.; Mendes, Y. M. M. B. E.; Vieira, M. G.; Steenhouwer, R. B.; Branco, K. M. P. C.; Santos, M. V. C. D.; Rivera, I. R.; Nicoloso, L. H.; Safadi, M. A. P.; Assis, D. M.	2021	non-English study
[Evaluation of two COVID-19 antigenic diagnostic tests: <i>BIOSYNEX^{Â®} COVID-19 Ag BSS</i> and <i>BIOSYNEX^{Â®} COVID-19 Ag + BSS</i> compared to <i>AmpliQuick^{Â®} SARS-CoV-2 PCR</i>]	Ngaba, Guy Pascal; Kalla, Ginette Claude Mireille; Assob, Jules ClÃ©ment Nguedia; Njouendou, Abdel Jelil; Jembe, Christian Nelly; Mboudou, Emile TÃ©lesphore; Mbopi-Keou, FranÃ§ois-Xavier	2021	non-English study
Coronavirus infection COVID-19 in children in the Russian Federation	Gorelov, Aleksandr V.; Nikolaeva, Svetlana V.; Akimkin, Vasily G	2020	non-English study

Study Title	Author names	Year	Reason for exclusion
Immunocompromised children and young people are at no increased risk of severe COVID-19	Chappell, H.; Patel, R.; Driessens, C.; Tarr, A. W.; Irving, W. L.; Tighe, P. J.; Jackson, H. J.; Harvey-Cowlishaw, T.; Mills, L.; Shaunak, M.; Gbesemete, D.; Leahy, A.; Lucas, J. S.; Faust, S. N.; de Graaf, H.	2022	patient/parent reported data
COVID-19 symptoms and diagnoses among a sociodemographically diverse cohort of children from New York city: lessons from the first wave, spring 2020	Kahn, L. G.; Ghassabian, A.; Jacobson, M. H.; Yu, Keunhyung; Trasande, L.	2021	patient/parent reported data
Covid-19 pandemic in Egyptian children with liver diseases: Incidence and impact on health care service delivery in a low/middle income country	Abdullatif, H.; Elakel, W.; Baroudy, S.; El-Karaksy, H.; Mogahed, E. A.	2022	patient/parent reported data
Prevalence of COVID-19 in Italian Children With Celiac Disease: A Cross-Sectional Study	Lionetti, E.; Fabbrizi, A.; Catassi, C.	2021	patient/parent reported data
COVID-19: A New Horizon in Congenital Heart Diseases	Aghaei Moghadam, E.; Mohammadzadeh, S.; Sattarzadeh Badkoubeh, R.; Ghamari, A.; Rabbani, A.; Mohebbi, A.; Zeinaloo, A.; Ashrafi, M.; Kamran, N.; Masoominasab, P.; Mahmoudi, Z.; Zamani Mehryan, A.; Mirzaaghayan, M. R.	2021	patient/parent reported data
Household transmission of SARS-CoV-2 from children and adolescents	Chu, V. T.; Yousaf, A. R.; Chang, K.; Schwartz, N. G.; McDaniel, C. J.; Lee, S. H.; Szablewski, C. M.; Brown, M.; Drenzek, C. L.; Dirlikov, E.; Rose, D. A.; Villanueva, J.; Fry, A. M.; Hall, A. J.; Kirking, H. L.; Tate, J. E.; Lanzieri, T. M.; Stewart, R. J.	2021	patient/parent reported data
Multisystem inflammatory syndrome in European White children â€“ study of 274 cases	Ludwikowska, Kamila Maria Okarska-NapieraÅ‚a Magdalena Dudek Natalia Tracewski PaweÅ‚ Kusa Jacek PiwoÅ„ski Krzysztof Afelt Aneta Cysewski Dominik Biela Mateusz Werner BoÅ¼ena Jackowska Teresa Suski Catherine Kursa Miron Bartosz Kuchar Ernest Szenborn Leszek	2021	preprint
SARS-CoV-2 surveillance (09/2020 - 03/2021) in elementary schools and daycare facilities in Bavaria	Anna, Kern; Pia, H. Kuhlmann; Stefan, Matl; Markus, Ege; Nicole, Maison; Jana, Eckert; Ulrich von, Both; Uta, Behrends; Melanie, Anger; Michael, C. FrÃ¼hwald; Michael, Gerstlauer; Joachim, Woelfle; Antje, Neubert; Michael, Melter; Johannes, Liese; David,	2022	preprint

Study Title	Author names	Year	Reason for exclusion
	Goettler; Andreas, Sing; Bernhard, Liebl; Johannes, HÃ¼bner; Christoph, Klein; Covid Kids Bavaria, Consortium		
CORONAVIRUS DISEASE 2019 IN A TERTIARY PEDIATRIC CENTER IN PORTUGAL	Tiago Milheiro, Silva; Ana Margarida, Garcia; Catarina, Gouveia; Flora, Candeias; Maria Joao, Brito	2021	preprint
Asthma and the Risk of SARS-CoV-2 Infection Among Children and Adolescents	Saahithi, Rao; Jillian, H. Hurst; Congwen, Zhao; Benjamin, A. Goldstein; Laine, Thomas; Jason, E. Lang; Matthew, S. Kelly	2021	preprint
Risk of SARS-CoV-2 testing, PCR-confirmed infections and COVID-19-related hospital admissions in children and young people: birth cohort study	Hardelid, P.; Favarato, G.; Wijlaars, L.; Fenton, L.; McMenamin, J.; Clemens, T.; Dibben, C.; Milojevic, A.; Macfarlane, A.; Taylor, J.; Cunningham, S.; Wood, R.	2021	preprint
A Comprehensive Clinical Description of Pediatric SARS-CoV-2 Infection in Western Pennsylvania	Freeman, M. C.; Gaietto, K.; DiCicco, L. A.; Rauenswinter, S.; Squire, J. R.; Aldewereld, Z.; Rapsinski, G.; Iagnemma, J.; Campfield, B. T.; Wolfson, D.; Kazmerski, T. M.; Forno, E.	2020	preprint
Coronavirus (COVID-19) infection in children at a specialist centre: outcome and implications of underlying high-risk comorbidities in a paediatric population	Richard, Issitt; John, Booth; William, Bryant; Anastasia, Spiridou; Andrew, Taylor; Pascale, DuPre; Padmanabhan, Ramnarayan; John, Hartley; Mario Cortino, Borja; Karyn, Moshal; Helen, Dunn; Harry, Hemingway; Neil, Sebire	2020	preprint
Comparison of outcomes from COVID infection in pediatric and adult patients before and after the emergence of Omicron	Wang, L.; Berger, N. A.; Kaelber, D. C.; Davis, P. B.; Volkow, N. D.; Xu, R.	2022	preprint
Screening for SARS-CoV-2 infections in daycare facilities for children in a large city in Germany	Nadine, Luebke; Anna-Kathrin, Schupp; Renate, Bredahl; Ursula, Kraus; Sandra, Hauka; Marcel, Andree; Lutz, Ehlkes; Thomas, Klein; Alexandra, Graupner; Johannes, Horn; Ralph, Brinks; Klaus, Goebels; Ortwin, Adams; Joerg, Timm	2021	preprint
Risk factors for SARS-CoV-2 infection and hospitalisation in children and adolescents in Norway: A nationwide population-based study	Ketil, Stordal; Paz Lopez-Doriga, Ruiz; Margrethe, Greve-Isdahl; Pal, Suren; Per Kristian, Knudsen; Hanne Lovdal, Gulseth; German, Tapia	2021	preprint
Baseline characteristics, management, and outcomes of 55,270 children and	Duarte-Salles, T.; Vizcaya, D.; Pistillo, A.; Casajust, P.; Sena, A. G.; Lai, L. Y. H.; Prats-Uribe, A.; Ahmed, W. U.; Alshammari, T. M.;	2020	preprint

Study Title	Author names	Year	Reason for exclusion
adolescents diagnosed with COVID-19 and 1,952,693 with influenza in France, Germany, Spain, South Korea and the United States: an international network cohort study	Alghoul, H.; Alser, O.; Burn, E.; You, S. C.; Areia, C.; Blacketer, C.; DuVall, S.; Falconer, T.; Fernandez-Bertolin, S.; Fortin, S.; Golozar, A.; Gong, M.; Tan, E. H.; Huser, V.; Iveli, P.; Morales, D. R.; Nyberg, F.; Posada, J. D.; Recalde, M.; Roel, E.; Schilling, L. M.; Shah, N. H.; Shah, K.; Suchard, M. A.; Zhang, L.; Williams, A. E.; Reich, C. G.; Kostka, K.; Prieto-Alhambra, D.		
Age-Specific Changes in Virulence Associated with SARS-CoV-2 Variants of Concern	David, Fisman; Ashleigh, Tuite	2021	preprint
Post-Acute COVID-19 Outcomes In Children Requiring Hospitalisation (preprint)	Bossley, Cara Kavaliunaite Ema Harman Katharine Cook James Ruiz Gary Gupta Atul	2021	preprint
Clinical Course and Characteristics of COVID-19 in Patients With Inborn Errors of Immunity: A Retrospective Multicenter Experience From Iran	Karimi, Abdollah Shokri Youssef Jamee Mahnaz Heidari Atefh Nazarpack Fatemeh Fallahi Mazdak Shiari Reza Sharifinejad Niusha Mesdaghi Mehrnaz Khalili Mitra Armin Shahnaz Ghanaie Roxana Mansour Tabatabaei Sedigheh Rafiei Fahimzad Seyed Alireza Eslami Narges Sharafian Samin Mahdaviani Seyed Alireza Nabavizadeh Seyed Hesamedin Esmaeilzadeh Hossein Kanannejad Zahra Babaei Maryam Mansouri Mahboubeh Babaei Delara Fallah Shahrzad Fard Nasrin Khakbazan Hashemimoghaddam Seyedeh Atefeh Alyasin Soheila Chavoshzadeh Zahra	2021	preprint
Safety and efficacy of oral lopinavir/ritonavir in pediatric patients with coronavirus disease: A nationwide comparative analysis	Lu, J. M.; Zhou, A. F.; Zhang, X. B.; Wang, X. F.; Ye, Q. F.; Shang, F. N.; He, Y. L.; Ma, S. L.; Cui, Y. X.; Chen, R. J.; Li, X. Y.; Zhai, X. W.; Xu, H.; Li, Z. P.	2021	small sample size
海南省69例新型冠状病毒肺炎确诊病例的流行病学与临床特征分析.	Chen	2020	small sample size
某县儿童感染新型冠状病毒的流行病学分析.	Gao	2020	small sample size

Study Title	Author names	Year	Reason for exclusion
亳州市新冠肺炎患者一般资料特征学分析.	Han	2020	small sample size
新型冠状病毒肺炎合并心血管疾病患者的胸部ct特点.	Jin	2020	small sample size
福建省新型冠状病毒肺炎流行病学特征分析.	Ou	2020	small sample size
新冠肺炎合并呼吸道病原体感染的相关性研究.	Tang	2021	small sample size
儿童新型冠状病毒肺炎的ct表现.	Xiong	2020	small sample size
云南省瑞丽市新型冠状病毒感染/肺炎成人和儿童本土病例的流行病学及临床特征.	Yang	2021	small sample size
108例住院患者2019新型冠状病毒核酸检测结果分析.	Yu	2020	small sample size
四川省新型冠状病毒肺炎聚集性疫情流行特征分析.	Zhou	2020	small sample size
儿童新型冠状病毒自然感染流行特征分析.	Zou	2021	small sample size
Immunocompromised seroprevalence and course of illness of SARS-CoV-2 in one pediatric quaternary care center	Freeman, M. C.; Rapsinski, G. J.; Zilla, M. L.; Wheeler, S. E.	2020	small sample size
Clinical profile, hospital course and outcome of children with COVID-19	Karthi, Nallasamy; Angurana, S. K.; Muralidharan, Jayashree; Mathew, J. L.; Arun, Bansal; Singh, M. P.; Ishani, Bora; Laxmi, P. V. M.; Sanjay, Verma; Naveen, Sankhyan; Vikas, Suri; Guru, R. R.; Puri, G. D.	2021	small sample size
Clinical characteristics and mortality associated with COVID-19 in Jakarta,	Surendra, Henry; Elyazar, Iqbal Rf; Djaafara, Bimandra A.; Ekawati, Lenny L.; Saraswati, Kartika; Adrian, Verry; Oktavia, Dwi; Salama,	2021	small sample size

Study Title	Author names	Year	Reason for exclusion
Indonesia: A hospital-based retrospective cohort study	Ngabila; Lina, Rosa N.; Andrianto, Adhi; Lestari, Karina D.; Burhan, Erlina; Shankar, Anuraj H.; Thwaites, Guy; Baird, J. Kevin; Hamers, Raph L.		
Characteristics and predictors of outcomes of critically Ill children with SARS-CoV-2 infection - the PICU experience	Kazi, M. A.; Roychowdhury, S.; Ghosh, S.; Mahapatra, M. K.; Bhakta, S.; Konar, M. C.; Sarkar, M.	2022	small sample size
Clinical and Therapeutic Approach to Hospitalized COVID-19 Patients: A Pediatric Cohort in Portugal	Saraiva, B. M.; Garcia, A. M.; Silva, T. M.; Gouveia, C.; Brito, M. J.	2021	small sample size
Maintenance of Elective Patient Care at Berlin University Children's Hospital During the COVID-19 Pandemic	Terliesner, N.; Rosen, A.; Kaindl, A. M.; Reuter, U.; Lippold, K.; Mall, M. A.; Bernuth, H. V.; Gratopp, A.	2021	small sample size
Comparison of clinical characteristics and outcomes of pediatric and adult patients with coronavirus disease 2019 in Shenzhen, China	Wang, Fang; Lai, ChangXiang; Huang, PengYu; Liu, JiaMing; Wang, XianFeng; Tang, QiYuan; Zhou, Xuan; Xian, WenJie; Chen, RuiKun; Li, Xuan; Li, ZhiYu; Liao, LiQun; He, Qing; Liu, Lei	2020	small sample size
Clinical characteristics of pediatric patients with COVID-19 in an emergency department	Morilla, L.; Morel, Z.; Pavlicich, V.	2020	small sample size
Clinical, epidemiological and cardiovascular aspects in children convalescents of covid-19 in In villa Clara province, Cuba	Vega, L. L.; Marrero, F. E. P.; Del GonzÃ¡lez, L. R. L.; Machado, M. D. N.; Ygualada, J. A. S.; Chang, Y. A.	2021	small sample size
Seroprevalence and Clinical Outcomes of SARS-CoV-2 in Paediatric Patients with Rheumatic Disease	Walters, H. M.; Mian, Z.; Thomas, L.; Cerise, J.; Eberhard, B. A.; Pagano, E.; Gottlieb, B. S.; Steigerwald, K.; Hui-Yuen, J. S.	2021	small sample size
Detection of Respiratory Pathogens Does Not Predict Risks After Outpatient Adenotonsillectomy	Vickers, D. M.; Reddy, A.; Akmyradov, C.; Brown, K. M.; Boyanton, B. L.; Wright, H. D.; Taylor, J. A.; Childress, S. H.; Hartzell, L. D.; Johnson, A. B.; Key, J. M.; Nolder, A. R.; Richter, G. T.; Wineland, A. M.; Strub, G. M.	2021	small sample size
"Second Wave" of COVID-19 Pandemic: Admittance on Pediatric Emergency	Mata Zubillaga, D.; Gonzalez Garcia, L. G.; Garcia Aparicio, C.; Laso Alonso, A. E.; Rodriguez Manchon, S.; Corral Hospital, S.	2021	small sample size

Study Title	Author names	Year	Reason for exclusion
Department of a Regional Hospital From North of Spain During State of Alarm			
Incidence of COVID-19 in a cohort of adult and paediatric patients with rheumatic diseases treated with targeted biologic and synthetic disease-modifying anti-rheumatic drugs	Michelena, X.; Borrell, H.; Lopez-Corbeto, M.; Lopez-Lasanta, M.; Moreno, E.; Pascual-Pastor, M.; Erra, A.; Serrat, M.; Espartal, E.; Anton, S.; Anez, G. A.; Caparros-Ruiz, R.; Pluma, A.; Trallero-Araguas, E.; Barcelo-Bru, M.; Almirall, M.; De Agustin, J. J.; Llados, J.; Julia, A.; Marsal, S.	2020	small sample size
How we deal with the COVID-19 epidemic in an Italian paediatric onco-haematology clinic located in a region with a high density of cases	Sainati, L.; Biffi, A.	2020	small sample size
Acute kidney injury in pediatric patients hospitalized with acute COVID-19 and multisystem inflammatory syndrome in children associated with COVID-19	Basalely, A.; Gurusinghe, S.; Schneider, J.; Shah, S. S.; Siegel, L. B.; Pollack, G.; Singer, P.; Castellanos-Reyes, L. J.; Fishbane, S.; Jhaveri, K. D.; Mitchell, E.; Merchant, K.; Capone, C.; Gefen, A. M.; Steinberg, J.; Sethna, C. B.	2021	small sample size
Favourable perioperative outcomes for children with SARS-CoV-2	Nepogodiev, D.	2020	small sample size
Paediatric COVID-19 admissions in a region with open schools during the two first months of the pandemic	Hildenwall, H.; Luthander, J.; Rhedin, S.; Hertting, O.; Olsson-Akefeldt, S.; Melen, E.; Alfven, T.; Herlenius, E.; Rinder, M. R.	2020	small sample size
Observations about symptomatic and asymptomatic infections of 494 patients with COVID-19 in Shanghai, China	Mei, X.; Zhang, Y.; Zhu, H.; Ling, Y.; Zou, Y.; Zhang, Z.; Guo, H.; Liu, Y.; Cheng, X.; Liu, M.; Huang, W.; Wang, J.; Yi, Z.; Qian, Z.; Lu, H.	2020	small sample size
Paediatric COVID-19: milder presentation - a silver lining in dark cloud	Sharmila, Ramteke; Rajesh, Tikkas; Manjusha, Goel; Shipra, Mandraha; Jyotsna, Shrivastava	2020	small sample size
A Preliminary Report of COVID-19 in Children in India	Banerjee, S.; Guha, A.; Das, A.; Nandi, M.; Mondal, R.	2020	small sample size
Pediatric COVID-19 and Appendicitis: A Gut Reaction to SARS-CoV-2?	Malhotra, A.; Sturgill, M.; Whitley-Williams, P.; Lee, Y. H.; Esochaghi, C.; Rajasekhar, H.; Olson, B.; Gaur, S.	2021	small sample size
Severe clinical spectrum with high mortality in pediatric patients with COVID-19 and multisystem inflammatory syndrome	Pereira, M. F. B.; Litvinov, N.; Farhat, S. C. L.; Eisencraft, A. P.; Gibelli, Mabc; Carvalho, W. B.; Fernandes, V. R.; Fink, T. T.; Framil, J. V. S.; Galleti, K. V.; Fante, A. L.; Fonseca, M. F. M.; Watanabe, A.; Paula, C. S. Y.; Palandri, G. G.; Leal, G. N.; Diniz, M. F. R.; Pinho, J.	2020	small sample size

Study Title	Author names	Year	Reason for exclusion
	R. R.; Silva, C. A.; Marques, H. H. S.; Pediatric, Covid H. C. Fmusp Study Group; Rossi Junior, A.; Delgado, A. F.; Andrade, A. P. M.; Schvartsman, C.; Sabino, E. C.; Rocha, M. C.; Kanunfre, K. A.; Okay, T. S.; Carneiro-Sampaio, M. M. S.; Jorge, P. P. D.		
Could the covid-19 infection have a better prognosis than expected in pediatric hematology oncology and bone marrow transplant patients?	Oner, O. B.; Aksoy, B. A.; Yaman, A.; Sutcu, M.; Cipe, F.; Atca, A. O.; Bozkurt, C.; Fisgin, T.	2021	small sample size
The epidemiological and clinical profile of covid-19 in children: Moroccan experience of the cheikh khalifa university center	Chekhlabi, N.; Kettani, C. E.; Haoudar, A.; Bahlaoui, A.; Mahi, M.; Ettair, S.; Dini, N.	2020	small sample size
Management of Children with Severe COVID-19 in a Pediatrics Unit in Istanbul: A Retrospective Study	Karaaslan, AyÅŸe Ã‡etin Ceren AkÄ±n Yasemin Bal Cem Murat Demirhan Recep	2021	small sample size
Evaluation of COVID-19 patients in the pediatric critical care unit	Durak, C.; Aygun, F.; Aygun, D.; Kilinc, A. A.; Onal, P.	2021	small sample size
COVID-19 in cystic fibrosis patients	Kondratâ€™eva, E. I.; Krasovskiy, S. A.; Kashirskaya, N. Yu Amelina E. L.; Zhekayte, E. K.; Sherman, V. D.; Simonova, O. I.; Gorinova, Y. V.; Boitsova, E. V.; Mukhina, M. A.; Butyugina, I. N.; Makarova, M. A.; Malakhov, A. B.	2020	small sample size
COVID-19 in cancer patients on active systemic therapy - outcomes from LMIC scenario with an emphasis on need for active treatment	Anant, Ramaswamy; Lingaraj, Nayak; Moulik, N. R.; Manju, Sengar; Girish, Chinnaswamy; Kunal, Jobanputra; Shah, M. J.; Akhil, Kapoor; Amit, Joshi; Amit, Kumar; Anant, Gokarn; Avinash, Bonda; Parambil, B. C.; Maya, Prasad; Bhausaheb, Bagal; Chetan, Dhamne; Gaurav, Narula; Hasmukh, Jain; Jaya, Ghosh; Jayashree, Thorat; Jyoti, Bajpai; Nandini, Menon; Navin, Khattry; Prabhat, Bhargava; Sachin, Punatar; Seema, Gulia; Shripad, Banavali; Sudeep, Gupta; Sujay, Srinivas; Sushmita, Rath; Tushar, Vora; Vanita, Noronha; Patil, V. M.; Vikas, Ostwal; Kumar, Prabhash	2020	small sample size
Infectivity, susceptibility, and risk factors associated with SARS-CoV-2	Hu, Shixiong Wang Wei Wang Yan Litvinova Maria Luo Kaiwei Ren Lingshuang Sun Qianlai Chen Xinghui Zeng Ge Li Jing Liang Lu Deng Zhihong Zheng Wen Li Mei Yang Hao Guo Jinxin Wang Kai	2020	small sample size

Study Title	Author names	Year	Reason for exclusion
transmission under intensive contact tracing in Hunan, China	Chen Xinhua Liu Ziyan Yan Han Shi Huilin Chen Zhiyuan Zhou Yonghong Sun Kaiyuan Vespignani Alessandro Viboud CÃ©cile Gao Lidong Ajelli Marco Yu Hongjie		
Symptomatology and outcome of acute COVID-19 illness in children at Faridabad, India	Gupta, Priyanka Bhinder Onkar Gupta Vipul Ahuja Abhinav Pandey Anil Ravi R. Mandal	2021	small sample size
Clinical Characteristics and Outcomes of COVID-19 in Children in Northern Iran	Shahbaznejad, L.; Rouhanizadeh, H.; Navaeifar, M. R.; Hosseinzadeh, F.; Movahedi, F. S.; Rezai, M. S.	2021	small sample size
The Wide Spectrum of COVID-19 Clinical Presentation in Children	Nathan, N.; Prevost, B.; Sileo, C.; Richard, N.; Berdah, L.; Thouvenin, G.; Aubertin, G.; Lecarpentier, T.; Schnuriger, A.; Jegard, J.; Guellec, I.; Taytard, J.; Corvol, H.	2020	small sample size
Is vitamin D deficiency a risk factor for COVID-19 in children?	Yilmaz, K.; Sen, V.	2020	small sample size
Pediatric Asthma Exacerbation in Children with Suspected and Confirmed Coronavirus Disease 2019 (COVID-19): An Observational Study from Saudi Arabia	Asseri, A. A.	2021	small sample size
Novel Coronavirus Infection in Hospitalized Infants Under 1 Year of Age in China	Wei, Min Yuan; Jingping Liu, Yu Fu Tao Yu Xue Zhang Zhi-Jiang	2020	small sample size
Clinical Spectrum of COVID-19 in a Mexican Pediatric Population	Bustos-Cordova, E.; Castillo-Garcia, D.; Ceron-Rodriguez, M.; Soler-Quinones, N.	2021	small sample size
Coronavirus disease in children: a multicentre study from the Kingdom of Saudi Arabia	Kari, J. A.; Shalaby, M. A.; Albanna, A. S.; Alahmadi, T. S.; Sukkar, S. A.; MohamedNur, H. A. H.; Al-Ghamdi, M. S.; Basri, A. H.; Shagal, R. A.; Abeer, Alnajar; Mazen, Badawi; Safdar, O. Y.; Zaher, Z. F.; Mohamad-Hani, Temsah; Alhasan, K. A.	2021	small sample size
Coronavirus disease 2019 in children: Characteristics, antimicrobial treatment, and outcomes	Peng, H.; Gao, P.; Xu, Q.; Liu, M.; Peng, J.; Wang, Y.; Xu, H.	2020	small sample size
Pediatric patients with COVID-19 admitted to intensive care units in Brazil: a prospective multicenter study	Prata-Barbosa, A.; Lima-Setta, F.; Santos, G. R. dos; Lanziotti, V. S.; Castro, R. E. V. de; Souza, D. C. de; Raymundo, C. E.; Oliveira, F. R. C. de; Lima, L. F. P. de; Tonial, C. T.; Colleti, J., Jr.; Bellinat, A. P.	2020	small sample size

**Study Title**	**Author names**	**Year**	**Reason for exclusion**
	N.; Lorenzo, V. B.; Zeitel, R. de S.; Pulcheri, L.; Costa, F. C. M. da; Torre, F. P. F. la; Figueiredo, E. A. D. N.; Silva, T. P. da; Riveiro, P. M.; Mota, I. C. F. da; Brandao, I. B.; Azevedo, Z. M. A. de; Gregory, S. C.; Boedo, F. R. O.; Carvalho, R. N. de; Castro, N. A. de A. S. R.; Genu, D. H. S.; Foronda, F. A. K.; Cunha, A. J. L. A.; Magalhaes-Barbosa, M. C. de		
COVID-19 diagnosis and testing in pediatric heart transplant recipients	Bock, M. J.; Kuhn, M. A.; Chinnock, R. E.	2021	small sample size
Evaluation of children followed up for Covid-19 in a tertiary hospital	Ozkan, E. A.; Erdeniz, E. H.	2021	small sample size
SARS-CoV-2 infections in children and adolescents with rheumatic musculoskeletal diseases â€“ data from the National Pediatric Rheumatology Database in Germany	Sengler, Claudia Eulert Sascha Niewerth Martina Minden Kirsten Horneff Gerd Kuemmerle-Deschner Jasmin Siemer Caroline Berendes Rainer Girschick Hermann HÃ¼hn Regina Borte Michael Hospach Anton Emminger Wolfgang Armann Jakob Klein Ariane Kallinich Tilmann	2021	small sample size
Case Series of Variable Acute Appendicitis in Children with SARS-CoV-2 Infection	Engelis, Arnis; Smane, Liene; Pavare, Jana; Zviedre, Astra; Zurmutai, Timurs; Berezovska, Marisa M.; Bormotovs, Jurijs; Kakar, Mohit; Saxena, Amulya K.; Petersons, Aigars	2021	small sample size
Children Hospitalized With Severe COVID-19 in Wuhan	Wang, Y.; Zhu, F.; Wang, C.; Wu, J.; Liu, J.; Chen, X.; Xiao, H.; Liu, Z.; Wu, Z.; Lu, X.; Ma, J.; Zeng, Y.; Peng, H.; Sun, D.	2020	small sample size
COVID-19 and recurrent respiratory infections in children of Kazakhstan	Zhamankulov, A.; Rozenson, R.; Morenko, M.; Shnayder, K.; Akhmetova, U.; Tyo, A.	2021	small sample size
Infectivity, susceptibility, and risk factors associated with SARS-CoV-2 transmission under intensive contact tracing in Hunan, China	Hu, S.; Wang, W.; Wang, Y.; Litvinova, M.; Luo, K.; Ren, L.; Sun, Q.; Chen, X.; Zeng, G.; Li, J.; Liang, L.; Deng, Z.; Zheng, W.; Li, M.; Yang, H.; Guo, J.; Wang, K.; Chen, X.; Liu, Z.; Yan, H.; Shi, H.; Chen, Z.; Zhou, Y.; Sun, K.; Vespignani, A.; Viboud, C.; Gao, L.; Ajelli, M.; Yu, H.	2021	small sample size
Symptoms and transmission of SARS-CoV-2 among children - Utah and Wisconsin, March-May 2020	Laws, R. L.; Chancey, R. J.; Rabold, E. M.; Chu, V. T.; Lewis, N. M.; Fajans, M.; Reses, H. E.; Duca, L. M.; Dawson, P.; Conners, E. E.; Gharpure, R.; Yin, S.; Buono, S.; Pomeroy, M.; Yousaf, A. R.; Owusu, D.; Wadhwa, A.; Pevzner, E.; Battey, K. A.; Njuguna, H.; Fields, V. L.; Salvatore, P.; O'Hegarty, M.; Vuong, J.; Gregory, C. J.; Banks, M.; Rispens, J.; Dietrich, E.; Marcenac, P.; Matanock, A.;	2021	small sample size

Study Title	Author names	Year	Reason for exclusion
	Pray, I.; Westergaard, R.; Dasu, T.; Bhattacharyya, S.; Christiansen, A.; Page, L.; Dunn, A.; Atkinson-Dunn, R.; Christensen, K.; Kiphibane, T.; Willardson, S.; Fox, G.; Ye, D.; Nabity, S. A.; Binder, A.; Freeman, B. D.; Lester, S.; Mills, L.; Thornburg, N.; Hall, A. J.; Fry, A. M.; Tate, J. E.; Tran, C. H.; Kirking, H. L.		
Clinical and epidemiological characteristics of children with SARS-CoV-2 infection admitted in a Peruvian hospital	Rodriguez-Portilla, R.; Llaque-Quiroz, P.; Guerra-Rios, C.; Cieza-Yamunaque, L. P.; Coila-Paricahua, E. J.; Baique-Sanchez, P. M.; Pinedo-Torres, I.	2021	small sample size
COVID-19 and Congenital Heart Disease: Results from a Nationwide Survey	Sabatino, J.; Ferrero, P.; Chessa, M.; Bianco, F.; Ciliberti, P.; Secinaro, A.; Oreto, L.; Avesani, M.; Bucciarelli, V.; Calcaterra, G.; Calabro, M. P.; Russo, M. G.; Bassareo, P. P.; Guccione, P.; Indolfi, C.; Di Salvo, G.	2020	small sample size
Analytical statistical study on various aspects of covid-19 among pediatric age group in Kirkuk-Iraq	Rasheed, A. A.; Tahir, S. S.; Kareem, A. D. A.	2021	small sample size
Persistent symptoms and decreased health-related quality of life after symptomatic pediatric COVID-19: A prospective study in a Latin American tertiary hospital	Fink, T. T.; Marques, H. H. S.; Gualano, B.; Lindoso, L.; Bain, V.; Astley, C.; Martins, F.; Matheus, D.; Matsuo, O. M.; Suguita, P.; Trindade, V.; Paula, C. S. Y.; Farhat, S. C. L.; Palmeira, P.; Leal, G. N.; Suzuki, L.; Odone Filho, V.; Carneiro-Sampaio, M.; Duarte, A. J. S.; Antonangelo, L.; Batisttella, L. R.; Polanczyk, G. V.; Pereira, R. M. R.; Carvalho, C. R. R.; Buchpiguel, C. A.; Xavier, A. C. L.; Seelaender, M.; Silva, C. A.; Pereira, M. F. B.; Hc-Fmusp Pediatric Post, Covid-Study Group; Sallum, A. M. E.; Brentani, A. V. M.; Neto, A. J. S.; Ihara, A.; Santos, A. R.; Canton, A. P. M.; Watanabe, A.; Santos, A. C. D.; Pastorino, A. C.; Franco, Bdgm; Caruzo, B.; Ceneviva, C.; Martins, Ccmf; Prado, D.; Abellan, D. M.; Benatti, F. B.; Smaria, F.; Goncalves, F. T.; Penteado, F. D.; Castro, G. S. F.; Goncalves, G. S.; Roschel, H.; Disi, I. R.; Marques, I. G.; Castro, I. A.; Buscatti, I. M.; Faiad, J. Z.; Fiamoncini, J.; Rodrigues, J. C.; Carneiro, J. D. A.; Paz, J. A.; Ferreira, J. C.; Ferreira, J. C. O.; Silva, K. R.; Bastos, K. L. M.; Kozu, K.; Cristofani, L. M.; Souza, L. V. B.; Campos, L. M. A.; Silva Filho, Lvrf; Sapienza, M. T.; Lima, M. S.;	2021	small sample size

Study Title	Author names	Year	Reason for exclusion
	Garanito, M. P.; Santos, M. F. A.; Dorna, M. B.; Aikawa, N. E.; Litvinov, N.; Sakita, N. K.; Gaiolla, P. V. V.; Pasqualucci, P.; Toma, R. K.; Correa-Silva, S.; Sieczkowska, S. M.; Imamura, M.; Forsait, S.; Santos, V. A.; Zheng, Y.		
Clinical and laboratory characteristics of SARS-CoV2-infected paediatric patients in Jordan: serial RT-PCR testing until discharge	Kilani, M. M.; Odeh, M. M.; Shalabi, M.; Al Qassieh, R.; Al-Tamimi, M.	2021	small sample size
Initial report on Spanish pediatric oncologic, hematologic, and post stem cell transplantation patients during SARS-CoV-2 pandemic	Faura, A.; Rives, S.; Lassaletta, A.; Sebastian, E.; Madero, L.; Huerta, J.; Garcia-Morin, M.; Perez Martinez, A.; Sisinni, L.; Astigarraga, I.; Velasco, P.; Gros, L.; Moreno, L.; Carbone, A.; Rodriguez-Vigil, C.; Riesco, S.; Mendoza, M. del C. del; Garcia Macias, E.; Trabazo, M.; Torrent, M.; Badell, I.; Fuster, J. L.; Dominguez-Pinilla, N.; Ribelles, A. J.; Perez-Alonso, V.; Fernandez Sanmartin, M.; Baragano, M.; Gorostegui, M.; Perez-Jaume, S.; Fernandez-Teijeiro, A.; Morales la Madrid, A.; Dapena, J. L.	2020	small sample size
COVID-19 screening in a Portuguese pediatric population	Costa, A.; Almeida, H.; Moniz, M.; Alves, C.	2022	small sample size
Comparison of the medical burden of COVID-19 with seasonal influenza and measles outbreaks	Nesselroth, D.; Yakub Hana, H.; Gleyzer, A.; Simoes, E. A. F.; Abu Atta, M.; Ben Yehuda, Y.; Bibi, H.; Somekh, I.; Somekh, E.	2021	small sample size
Persistent symptoms in Swedish children after hospitalisation due to COVID-19	Sterky, E.; Olsson-Akefeldt, S.; Hertting, O.; Herlenius, E.; Alfven, T.; Ryd Rinder, M.; Rhedin, S.; Hildenwall, H.	2021	small sample size
Pediatric liver transplantation activity in a high-volume program during the COVID-19 pandemic in Brazil	Fonseca, E. A.; Feier, F.; Pugliese, R.; Freitas, A. F.; Porta, G.; Miura, I.; Baggio, V.; Kondo, M.; Benavides, M.; Vincenzi, R.; Roda, K.; Oliveira, C. V.; Chapchap, P.; Seda-Neto, J.	2021	small sample size
Patients in hospital with laboratory-confirmed COVID-19 in a network of Canadian acute care hospitals, Mar. 1 to Aug. 31, 2020: a descriptive analysis	Mitchell, Robyn; Choi, Kelly Baekyung; Pelude, Linda; Rudnick, Wallis; Thampi, Nisha; Taylor, Geoffrey	2021	small sample size
Pre-operative assessment of pediatric congenital heart disease patients in the COVID-19 era: lessons learned	Younis, N. K.; Zareef, R. O.; Diab, M. A.; El Sedawi, O.; El-Rassi, I. M.; Bitar, F.; Arabi, M.	2021	small sample size

**Study Title**	**Author names**	**Year**	**Reason for exclusion**
Comparison of acute pneumonia caused by SARS-COV-2 and other respiratory viruses in children: a retrospective multi-center cohort study during COVID-19 outbreak	Ren, Guang-Li; Wang, Xian-Feng; Xu, Jun; Li, Jun; Meng, Qiong; Xie, Guo-Qiang; Huang, Bo; Zhu, Wei-Chun; Lin, Jing; Tang, Cheng-He; Ye, Sheng; Li, Zhuo; Zhu, Jie; Tang, Zhen; Ma, Ming-Xin; Xie, Cong; Wu, Ying-Wen; Liu, Chen-Xi; Yang, Fang; Zhou, Yu-Zong; Zheng, Ying; Lan, Shu-Ling; Chen, Jian-Feng; Ye, Feng; He, Yu; Wu, Ben-Qing; Chen, Long; Fu, Si-Mao; Zheng, Cheng-Zhong; Shi, Yuan	2021	small sample size
Clusters of COVID-19 associated with Purim celebration in the Jewish community in Marseille, France, March 2020	Aherfi, Sarah; Gautret, Philippe; Chaudet, HervÃ©; Raoult, Didier; La Scola, Bernard	2020	small sample size
Clinical features and laboratory characteristics of patients hospitalized with COVID-19: single centre report from Egypt	El Kassas, M.; Asem, N.; Abdelazeem, A.; Madkour, A.; Sayed, H.; Tawheed, A.; Al Shafie, A.; Gamal, M.; Elsayed, H.; Badr, M.; Hassany, M.; Omran, D.; El Fouly, A.	2020	small sample size
Clinical and CT features of the COVID-19 infection: comparison among four different age groups	Li, W.; Fang, Y.; Liao, J.; Yu, W.; Yao, L.; Cui, H.; Zeng, X.; Li, S.; Huang, C.	2020	small sample size
COVID-19 in the Pediatric Population Admitted to a Tertiary Referral Hospital in Northern Italy: Preliminary Clinical Data	Brambilla, I.; Castagnoli, R.; Caimmi, S.; Ciprandi, G.; Luigi Marseglia, G.	2020	small sample size
Cohort profile: SARS-CoV-2/COVID-19 hospitalised patients in Switzerland	Amaury, T.; Anne, I.; Carlo, B.; Laurence, S.; Nicolas, T.; Andreas, W.; Domenica, F.; Schreiber, P. W.; Miriam, V.; Lauro, D.; Michael, B.; Danielle, V. G.; Christoph, K.; Alexia, C.; Thomas, R.; Yvonne, N. O.; Roman, G.; Ulrich, H.; Christoph, B.; Franziska, Z.; Sara, B. S.; Natascia, C.; Petrau, Z.; Anitau, U.; Anita, N. L.; Celine, G.; Maroussia, R.; Olivia, K.	2021	small sample size
Childhood COVID-19: a multicentre retrospective study	Chen, Z.; Tong, L.; Zhou, Y.; Hua, C.; Wang, W.; Fu, J.; Shu, Q.; Hong, L.; Xu, H.; Xu, Z.; Chen, Y.; Mao, Y.; Ye, S.; Wu, X.; Wang, L.; Luo, Y.; Zou, X.; Tao, X.; Zhang, Y.	2020	small sample size
Characteristics and outcomes of coronavirus disease 2019 (COVID-19) in critically ill pediatric patients admitted to	Abdulla, Alfraij; Bin Alamir, A. A.; Al-Otaibi, A. M.; Danah, Alsharrah; Abdulrahman, Aldaithan; Kamel, A. M.; Muna, Almutairi;	2021	small sample size

**Study Title**	**Author names**	**Year**	**Reason for exclusion**
the intensive care unit: a multicenter retrospective cohort study	Salman, Alshammari; Mohammed, Almazyad; Macarambon, J. M.; Mohammad, Alghounaim		
Household Transmission of Severe Acute Respiratory Syndrome Coronavirus-2 in the United States	Lewis, N. M.; Chu, V. T.; Ye, D.; Conners, E. E.; Gharpure, R.; Laws, R. L.; Reses, H. E.; Freeman, B. D.; Fajans, M.; Rabold, E. M.; Dawson, P.; Buono, S.; Yin, S.; Owusu, D.; Wadhwa, A.; Pomeroy, M.; Yousaf, A.; Pevzner, E.; Njuguna, H.; Battey, K. A.; Tran, C. H.; Fields, V. L.; Salvatore, P.; O'Hegarty, M.; Vuong, J.; Chancey, R.; Gregory, C.; Banks, M.; Rispens, J. R.; Dietrich, E.; Marcenac, P.; Matanock, A. M.; Duca, L.; Binder, A.; Fox, G.; Lester, S.; Mills, L.; Gerber, S. I.; Watson, J.; Schumacher, A.; Pawloski, L.; Thornburg, N. J.; Hall, A. J.; Kiphibane, T.; Willardson, S.; Christensen, K.; Page, L.; Bhattacharyya, S.; Dasu, T.; Christiansen, A.; Pray, I. W.; Westergaard, R. P.; Dunn, A. C.; Tate, J. E.; Nabity, S. A.; Kirking, H. L.	2021	small sample size
The effects of COVID-19 outbreak on Pediatric Emergency Department admissions for acute wheezing	Di Sarno, L.; Curatola, A.; Conti, G.; Covino, M.; Bertolaso, C.; Chiaretti, A.; Gatto, A.	2022	small sample size
Asymptomatic severe acute respiratory syndrome coronavirus 2 infection in patients with inflammatory bowel disease under biologic treatment	Norsa, L.; Cosimo, P.; Indriolo, A.; Sansotta, N.; D'Antiga, L.; Callegaro, A.	2020	small sample size
A single centre study of viral community-acquired pneumonia in children: no evidence of SARS-CoV-2 from October 2019 to March 2020. (Special Section: Coronavirus.)	Mancino, E.; Cristiani, L.; Pierangeli, A.; Scagnolari, C.; Nenna, R.; Petrarca, L.; Mattia, G. di; Regina, D. la; Frassanito, A.; Oliveto, G.; Viscido, A.; Midulla, F.	2020	small sample size
COVID-19 in a cohort of pregnant women and their descendants, the MOACC-19 study	Llorca, J.; Lechosa-Muniz, C.; Gortazar, P.; Fernandez-Ortiz, M.; Jubete, Y.; Cabero, M. J.	2021	small sample size
SARS-CoV-2 acute bronchiolitis in hospitalized children: neither frequent nor more severe	Andina-Martinez, D.; Alonso-Cadenas, J. A.; Cobos-Carrascosa, E.; Bodegas, I.; Oltra-Benavent, M.; Plazaola, A.; Epalza, C.; Jimenez-Garc..a, R.; Moraleda, C.; Tagarro, A.	2021	small sample size

Study Title	Author names	Year	Reason for exclusion
Distinctive clinical and laboratory features of COVID-19 and H1N1 influenza infections among hospitalized pediatric patients	Asseri, A. A.; Shati, A. A.; Al-Qahtani, S. M.; Alzaydani, I. A.; Al-Jarie, A. A.; Alaliani, M. J.; Ali, A. S.	2021	small sample size
Characteristics and outcomes of neonatal SARS-CoV-2 infection in the UK: a prospective national cohort study using active surveillance	Gale, C.; Quigley, M. A.; Placzek, A.; Knight, M.; Ladhani, S.; Draper, E. S.; Sharkey, D.; Doherty, C.; Mactier, H.; Kurinczuk, J. J.	2021	small sample size
Mortality in children with positive SARS-CoV-2 polymerase chain reaction test: Lessons learned from a tertiary referral hospital in Indonesia	Dewi, R.; Kaswandani, N.; Karyanti, M. R.; Setyanto, D. B.; Pudjiadi, A. H.; Hendarto, A.; Djer, M. M.; Prayitno, A.; Yuniar, I.; Indawati, W.; Prawira, Y.; Handryastuti, S.; Sjakti, H. A.; Hidayati, E. L.; Muktiarti, D.; Soebadi, A.; Puspaningtyas, N. W.; Muhaimin, R.; Rahmadhany, A.; Octavius, G. S.; Puspitasari, H. A.; Jasin, M. R.; Tartila, T.; Putri, N. D.	2021	small sample size
Persistent cough and asthma-like symptoms post COVID-19 hospitalization in children	esmaeilzadeh, hossein Dashti Anahita Sanaei Mortazavi Negar Fatemian Fatemian Vali Mohebat	2021	small sample size
Paediatric Contacts of Adult COVID-19 Patients: Clinical Parameters, Risk Factors, and Outcome	Farooq, A.; Sheikh, T. K.; Syed, F.; Mustafa, T.	2021	small sample size
COVID-19 in children with haematological malignancies	Millen, Gerard Cathal; Arnold, Roland; Cazier, Jean-Baptiste; Curley, Helen; Feltbower, Richard; Gamble, Ashley; Glaser, Adam; Grundy, Richard G.; Kirton, Laura; Lee, Lennard Y. W.; McCabe, Martin G.; Palles, Claire; Phillips, Bob; Stiller, Charles A.; Varnai, Csilla; Kearns, Pamela	2021	small sample size
Early experience of COVID-19 in a US Children's Hospital	Kainth, M. K.; Goenka, P. K.; Williamson, K. A.; Fishbein, J. S.; Subramony, A.; Barone, S.; Belfer, J. A.; Feld, L. M.; Krief, W. I.; Palumbo, N.; Rajan, S.; Rocker, J.; Scotto, T.; Sharma, S.; Sokoloff, W. C.; Schleien, C.; Rubin, L. G.	2020	small sample size
COVID-19, Australia: Epidemiology Report 16 (Reporting week to 23:59 AEST 17 May 2020)	Covid- National Incident Room Surveillance, Team	2020	small sample size

Study Title	Author names	Year	Reason for exclusion
91例儿童新型冠状病毒肺炎确诊病例临床及流行病学特征.	Liu	2020	small sample size
婴幼儿新型冠状病毒肺炎临床影像学分析.	Wang	2021	small sample size
新型冠状病毒感染儿童及青少年肝生物化学指标临床研究.	Wang	2021	small sample size
2021年福建莆田新冠delta株感染患儿中医证候及诊治规律初探.	Yuan	2021	small sample size
Paediatric multisystem inflammatory syndrome temporally associated with SARS-CoV-2 (PIMS-TS): Prospective, national surveillance, United Kingdom and Ireland, 2020	Flood, J.; Shingleton, J.; Bennett, E.; Walker, B.; Amin-Chowdhury, Z.; Oligbu, G.; Avis, J.; Lynn, R. M.; Davis, P.; Bharucha, T.; Pain, C. E.; Jyothish, D.; Whittaker, E.; Dwarakanathan, B.; Wood, R.; Williams, C.; Swann, O.; Semple, M. G.; Ramsay, M. E.; Jones, C. E.; Ramanan, A. V.; Gent, N.; Ladhani, S. N.	2021	systematic review
Epidemiological and Clinical Characteristics of COVID-19 in Children: A Systematic Review and Meta-Analysis	Li, B.; Zhang, S.; Zhang, R.; Chen, X.; Wang, Y.; Zhu, C.	2020	systematic review
Children's role in the COVID-19 pandemic: a systematic review of early surveillance data on susceptibility, severity, and transmissibility	Gaythorpe, K. A. M.; Bhatia, S.; Mangal, T.; Unwin, H. J. T.; Imai, N.; Cuomo-Dannenburg, G.; Walters, C. E.; Jauneikaite, E.; Bayley, H.; Kont, M. D.; Mousa, A.; Whittles, L. K.; Riley, S.; Ferguson, N. M.	2021	systematic review
60日龄以下发烧的新冠肺炎患儿的病程特点和临床特征.	Shi	2021	Unclear definition
四川省新型冠状病毒肺炎的流行特征分析.	Chen	2020	wrong age group
襄阳市1175例新型冠状病毒肺炎确诊病例流行病学特征分析.	Chen	2020	wrong age group

Study Title	Author names	Year	Reason for exclusion
四川省新型冠状病毒肺炎本地感染病例流行病学特征分析.	Cheng	2020	wrong age group
北京120转运首都机场入境4476例新型冠状病毒肺炎相关入境人员分析.	Gao	2021	wrong age group
安徽省新型冠状病毒肺炎流行病学特征分析.	Hou	2020	wrong age group
四川省新型冠状病毒肺炎无症状感染者流行病学特征分析.	Huang	2020	wrong age group
基于镇域尺度的河南省新型冠状病毒肺炎病例的空间格局演化与分布特征.	Jian	2020	wrong age group
新型冠状病毒肺炎恢复期患者86例临床特征分析.	Li	2020	wrong age group
55例中青年普通型新型冠状病毒肺炎患者的临床特征及预后分析.	Liang	2020	wrong age group
山东省481例新型冠状病毒肺炎流行病学特点分析.	Liu	2020	wrong age group
无症状或亚临床感染的covid-19儿童临床特征分析.	Liu	2020	wrong age group
新型冠状病毒肺炎患者347例实验室检测结果分析.	Liu	2021	wrong age group
吉林省新型冠状病毒肺炎流行病学特征分析.	Ma	2020	wrong age group
重庆地区2020年新型冠状病毒肺炎患者209例中医证型调查分析.	Ran	2020	wrong age group

Study Title	Author names	Year	Reason for exclusion
Cap患儿早期甄别covid-19的病例对照研究.	Ren	2020	wrong age group
2019-2020年冬春季武汉儿童医院收治流感和新型冠状病毒肺炎患儿的流行病学特点.	Tang	2020	wrong age group
78例新型冠状病毒肺炎患者舌象的初步研究.	Wang	2020	wrong age group
疫情早期107例儿童新型冠状病毒肺炎流行病学特征分析.	Zhang	2020	wrong age group
石家庄地区 133 例儿童 SARS-CoV-2感染流行病学特征	Lu	2021	wrong age group
Risk factors for severe COVID-19 in children	Graff, K.; Smith, C.; Silveira, L.; Jung, S.; Curran-Hays, S.; Jarjour, J.; Carpenter, L.; Pickard, K.; Mattiucci, M.; Fresia, J.; McFarland, E. J.; Dominguez, S. R.; Abuogi, L.	2021	wrong age group
Assessment of Clinical Outcomes Among Children and Adolescents Hospitalized With COVID-19 in 6 Sub-Saharan African Countries	Nachega, J. B.; Sam-Agudu, N. A.; Machekano, R. N.; Rabie, H.; van der Zalm, M. M.; Redfern, A.; Dramowski, A.; O'Connell, N.; Pipo, M. T.; Tshilanda, M. B.; Byamungu, L. N.; Masekela, R.; Jeena, P. M.; Pillay, A.; Gachuno, O. W.; Kinuthia, J.; Ishoso, D. K.; Amoako, E.; Agyare, E.; Agbeno, E. K.; Martyn-Dickens, C.; Sylverken, J.; Enimil, A.; Jibril, A. M.; Abdullahi, A. M.; Amadi, O.; Umar, U. M.; Sigwadhi, L. N.; Hermans, M. P.; Otokoye, J. O.; Mbala-Kingebeni, P.; Muyembe-Tamfum, J. J.; Zumla, A.; Sewankambo, N. K.; Aanyu, H. T.; Musoke, P.; Suleman, F.; Adejumo, P.; Noormahomed, E. V.;	2022	wrong age group

Study Title	Author names	Year	Reason for exclusion
	Deckelbaum, R. J.; Fowler, M. G.; Tshilolo, L.; Smith, G.; Mills, E. J.; Umar, L. W.; Siedner, M. J.; Kruger, M.; Rosenthal, P. J.; Mellors, J. W.; Mofenson, L. M.; African Forum for, Research; Education in Health, Covid-Research Collaboration on Children; Adolescents,		
Epidemiological and clinical features of Croatian children and adolescents with a PCR-confirmed coronavirus disease 2019: differences between the first and second epidemic wave	Krajcar, N.; Maric, L. S.; Surina, A.; Filipovic, S. K.; Trkulja, V.; Roglic, S.; TekiviC, G.	2020	wrong age group
Prevalence of COVID-19 in children, adolescents and adults in remote education situations in the city of Fortaleza, Brazil	Cavalcante Pinto JÃºnior, Valdester; Moura, Luiz Francisco Wemmenson GonÃ§alves; Cavalcante, Rodrigo Cardoso; Lima, JosÃ© Rubens Costa; Bezerra, Arnaldo Solheiro; de Sousa Dantas, Daylana RÃ©gia; Amaral, CÃcero Matheus Lima; Lima, Daniel Freire; JÃºnior, Antonio Brazil Viana; Florindo Guedes, Maria Izabel	2021	wrong age group
Clinical review of COVID-19 in children and adolescents with cancer: Experience from a tertiary care center in East India	Mohapatra, S.; Das, P. K.; Mishra, B.; Panigrahi, A.	2022	wrong age group
Prospective characterisation of SARS-CoV-2 infections among children presenting to tertiary paediatric hospitals across Australia in 2020: a national cohort study	Wurzel, D.; McMinn, A.; Hoq, M.; Blyth, C. C.; Burgner, D.; Tosif, S.; Buttery, J.; Carr, J.; Clark, J. E.; Cheng, A. C.; Dinsmore, N.; Francis, J. R.; Kynaston, A.; Lucas, R.; Marshall, H.; McMullan, B.; Singh-Grewal, D.; Wood, N.; Macartney, K.; Britton, P. N.; Crawford, N. W.	2021	wrong age group
Respiratory viruses in pediatric emergency department patients and their family members	Matienzo, Nelsa; Youssef, Mariam M.; Comito, Devon; Lane, Benjamin; Ligon, Chanel; Morita, Haruka; Winchester, Arianna; Decker, Mary E.; Dayan, Peter; Shopsin, Bo; Shaman, Jeffrey	2021	wrong age group
Acute Liver Injury Among Pediatric Liver Transplantation Recipients With Coronavirus Disease 2019: An International Collaborative Study	Sin, Priscila; DÃaz, Luis Antonio; MartÃnez, Mercedes; Vizcaya, Cecilia; D'Agostino, Daniel; Gana, Juan CristÃ³bal	2021	wrong age group
Socio-economic inequalities and COVID-19 incidence and mortality in Brazilian children: a nationwide register-based study	Martins-Filho, P. R.; Quintans-Junior, L. J.; de Souza Araujo, A. A.; Sposato, K. B.; Souza Tavares, C. S.; Gurgel, R. Q.; Fontes Leite, D. C.; de Paiva, S. M.; Santos, H. P., Jr.; Santos, V. S.	2021	wrong age group

Study Title	Author names	Year	Reason for exclusion
Age differences in clinical features and outcomes in patients with COVID-19, Jiangsu, China: a retrospective, multicentre cohort study	Luo, H.; Liu, S.; Wang, Y.; Phillips-Howard, P. A.; Ju, S.; Yang, Y.; Wang, D.	2020	wrong age group
Epidemiology of COVID-19 in the Kingdom of Saudi Arabia: An Ecological Study	Alyami, Mohammad H.; Naser, Abdallah Y.; Orabi, Mohamed A. A.; Alwafi, Hassan; Alyami, Hamad S.	2020	wrong age group
Clinical-epidemiological profile of children and adolescents with COVID-19 in Ceara. (Special issue.)	Cavalcante, A. N. M.; Tavares, L. V. de S.; Bastos, M. L. A.; Almeida, R. L. F. de	2021	wrong age group
Demographic and health indicators in correlation to interstate variability of incidence, confirmation, hospitalization, and lethality in Mexico: Preliminary analysis from imported and community acquired cases during COVID-19 outbreak	Mendez-Dominguez, N.; Alvarez-Baeza, A.; Carrillo, G.	2020	wrong age group
The effects of hospital organization on treatment during COVID-19 pandemic	Demirhan, R.; Cimenoglu, B.; Yilmaz, E.	2020	wrong age group
The impact of a mobile COVID-19 polymerase chain reaction laboratory at a large tertiary hospital during the first wave of the pandemic: A retrospective analysis	Omar, S.; Brown, J. M.; Mathivha, R. L.; Bahemia, I.; Nabeemeeah, F.; Martinson, N.	2021	wrong age group
SARS-CoV-2 in children and adolescents in Norway: confirmed infection, hospitalisations and underlying conditions	Stordal, K.; Bakken, I. J.; Greve-Isdahl, M.; Klingenberg, C.; Helland, E.; Nystad, W.; Hjellvik, V.; Gulseth, H. L.	2020	wrong age group
COVID-19 risk in elective surgery during a second wave: a prospective cohort study	Myles, P. S.; Wallace, S.; Story, D. A.; Brown, W.; Cheng, A. C.; Forbes, A.; Sidiropoulos, S.; Davidson, A.; Tan, N.; Jeffreys, A.; Hodgson, R.; Scott, D. A.; Radnor, J.	2021	wrong age group
COVID-19 and stem cell transplantation; results from an EBMT and GETH multicenter prospective survey	Ljungman, P.; Camara, R. de la; Mikulska, M.; Tridello, G.; Aguado, B.; Zahrani, M. A.; Apperley, J.; Berceanu, A.; Bofarull, R. M.; Calbacho, M.; Ciceri, F.; Lopez-Corral, L.; Crippa, C.; Fox, M. L.;	2021	wrong age group

**Study Title**	**Author names**	**Year**	**Reason for exclusion**
	Grassi, A.; Jimenez, M. J.; Demir, S. K.; Kwon, M.; Llamas, C. V.; Lorenzo, J. L. L.; Mielke, S.; Orchard, K.; Porras, R. P.; Vallisa, D.; Xhaard, A.; Knelange, N. S.; Cedillo, A.; Kr..ger, N.; Pi..ana, J. L.; Styczynski, J.		
Spectrum of COVID-19 clinical characteristics among patients presenting to the primary healthcare in Qatar during the early stages of the pandemic: a retrospective multicentre cross-sectional study	Ismail, M.; Joudeh, A.; Al-Dahshan, A.; Alsaadi, M. M.; Al Abdulla, S.; Selim, N. A. A.	2021	wrong age group
The first wave of COVID-19 in Malta; a national cross-sectional study	Micallef, S.; Piscopo, T. V.; Casha, R.; Borg, D.; Vella, C.; Zammit, M. A.; Borg, J.; Mallia, D.; Farrugia, J.; Vella, S. M.; Xerri, T.; Portelli, A.; Fenech, M.; Fsadni, C.; Azzopardi, C. M.	2020	wrong age group
Epidemiological Characteristics and Clinical Outcomes of Coronavirus Disease Patients in Northwest China: High-Volume Research From Low Population Density Regions	Zhu, J.; Zhang, Q.; Jia, C.; Chen, J.; Xia, Y.; Wang, W.; Wang, X.; Wen, M.; Wang, H.; Zhang, Z.; Xu, S.; Zhao, J.; Jiang, T.	2020	wrong age group
Rate of thrombosis in children and adolescents hospitalized with COVID-19 or MIS-C	Whitworth, H.; Sartain, S. E.; Kumar, R.; Armstrong, K.; Ballester, L.; Betensky, M.; Cohen, C. T.; Diaz, R.; Diorio, C.; Goldenberg, N. A.; Jaffray, J.; Keegan, J.; Malone, K.; Randolph, A. G.; Rifkin-Zenenberg, S.; Leung, W. S.; Sochet, A.; Srivaths, L.; Zia, A.; Raffini, L.	2021	wrong age group
Factors Associated With Hospitalization in Children and Adolescents With SARS-CoV-2 Infection	Chao, J. Y.; Sugarman, A.; Kimura, A.; Flamer, S.; Jing, T. T.; Fernandes, D. M.; Khine, H.; Shinnar, S.; Lo, Y.; Cabana, M. D.	2022	wrong age group
Male gender and kidney illness are associated with an increased risk of severe laboratory-confirmed coronavirus disease	Murillo-Zamora, E.; Trujillo, X.; Huerta, M.; Rios-Silva, M.; Mendoza-Cano, O.	2020	wrong age group
Demographic, clinical, and laboratory features of COVID-19 in children: The role of mean platelet volume in predicting hospitalization and severity	Guner Ozenen, G.; Sahbudak Bal, Z.; Umit, Z.; Bilen, N. M.; Yildirim Arslan, S.; Yurtseven, A.; Saz, E. U.; Burcu, B.; Sertoz, R.; Kurugol, Z.; Ozkinay, F.	2021	wrong age group

Study Title	Author names	Year	Reason for exclusion
SARS-CoV-2 RNAemia and clinical outcomes in children with COVID-19	Mertz, C.; Glowinski, R.; Cohen, S. H.; Mertz, S.; Ye, F.; Hall, M. W.; Peeples, M. E.; King, T.; Wang, H.; Leber, A. L.; Sanchez, P. J.; Ramilo, O.; Mejias, A.	2021	wrong age group
SARS-CoV-2 infection and mortality during the first epidemic wave in Madurai, south India: a prospective, active surveillance study	Laxminarayan, R.; B, C. M.; G, V. T.; Arjun Kumar, K. V.; Wahl, B.; Lewnard, J. A.	2021	wrong age group
Characteristics and outcomes of patients with COVID-19 admitted to hospital and intensive care in the first phase of the pandemic in Canada: a national cohort study	Murthy, Srinivas; Archambault, Patrick M.; Atique, Anika; Carrier, FranÃ§ois Martin; Cheng, Matthew P.; Codan, Cassidy; Daneman, Nick; Dechert, William; Douglas, Sarah; Fiest, Kirsten M.; Fowler, Robert; Goco, Geraldine; Gu, Yusing; Guerguerian, Anne-Marie; Hall, Richard; Hsu, Jimmy M.; Joffe, Ari; Jouvet, Philippe; Kelly, Laurel; Kho, Michelle E.; Kruisselbrink, Rebecca J.; Kumar, Deepali; Kutsogiannis, Demetrios James; Lamontagne, FranÃ§ois; Lee, Todd C.; Menon, Kusum; O'Grady, Heather; O'Hearn, Katie; Ovakim, Daniel H.; Pharand, Scott G.; Pitre, Tyler; Reel, Riley; Reeve, Brenda; Rewa, Oleksa; Richardson, David; Rishu, Asgar; Sandhu, Gyan; Sarfo-Mensah, Shirley; Shadowitz, Ellen; Sligl, Wendy; Solomon, Joshua; Stelfox, Henry T.; Swanson, Ashleigh; Tessier-Grenier, Hubert; Tsang, Jennifer L. Y.; Wood, Gordon	2021	wrong age group
Impact of the coronavirus disease 2019 (COVID-19) pandemic on the Italian congenital cardiac surgery system: a national survey	Giamberti, A.; Varrica, A.; Agati, S.; Gargiulo, G.; Luciani, G. B.; Mariamarianeschi, S.; Pacenapoleone, C.; Oppido, G.; Brunelli, F.; Palma, G.; Pak, V.; Arcieri, L.; Scalzo, G.; Padalino, M.; Galletti, L.	2020	wrong age group
Racial and Ethnic Disparities in Multisystem Inflammatory Syndrome in Children in the United States, March 2020 to February 2021	Stierman, B.; Abrams, J. Y.; Godfred-Cato, S. E.; Oster, M. E.; Meng, L.; Yip, L.; Patel, P.; Balachandran, N.; Prezzato, E.; Pierce, T.; Hsu, K. K.; Burns, M.; Peterson Pompa, X.; Lauro, P.; Hartley, A.; Jones, C.; Gretsch, S.; Reid, H.; Lim, S.; Campbell, A. P.; Belay, E. D.	2021	wrong age group
Race/Ethnicity Among Children With COVID-19-Associated Multisystem Inflammatory Syndrome	Lee, E. H.; Kepler, K. L.; Geevarughese, A.; Paneth-Pollak, R.; Dorsinville, M. S.; Ngai, S.; Reilly, K. H.	2020	wrong age group
Evaluation of the Clinical and Laboratory Findings of Asthmatic Children with SARS-CoV-2 Infection	Metbulut, A. P.; Mustafaoglu, O.; Sen, G.; Kanik Yuksek, S.; Kulhas Celik, I.; Akca, H.; Dibek Misirlioglu, E.	2021	wrong age group

**Study Title**	**Author names**	**Year**	**Reason for exclusion**
First and second waves of coronavirus disease-19: A comparative study in hospitalized patients in Reus, Spain	Iftimie, S.; Lopez-Azcona, A. F.; Vallverdu, I.; Hernandez-Flix, S.; de Febrer, G.; Parra, S.; Hernandez-Aguilera, A.; Riu, F.; Joven, J.; Andreychuk, N.; Baiges-Gaya, G.; Ballester, F.; Benavent, M.; Burdeos, J.; Catala, A.; Castane, E.; Castane, H.; Colom, J.; Feliu, M.; Gabaldo, X.; Garrido, D.; Garrido, P.; Gil, J.; Guelbenzu, P.; Lozano, C.; Marimon, F.; Pardo, P.; Pujol, I.; Rabassa, A.; Revuelta, L.; Rios, M.; Rius-Gordillo, N.; Rodriguez-Tomas, E.; Rojewski, W.; Roquer-Fanlo, E.; Sabate, N.; Teixido, A.; Vasco, C.; Camps, J.; Castro, A.	2021	wrong age group
COVID-19 infection in pediatric solid organ transplant patients	Bansal, N.; Ovchinsky, N.; Foca, M.; Lamour, J. M.; Kogan-Liberman, D.; Hsu, D. T.; Beddows, K.; Abraham, L.; Coburn, M.; Cunningham, R.; Nguyen, T.; Hayde, N.	2022	wrong age group
Comparison of the First and Second Waves of the Coronavirus Disease 2019 (COVID-19) Pandemic in Children and Adolescents in a Middle-Income Country: Clinical Impact Associated with Severe Acute Respiratory Syndrome Coronavirus 2 (SARS-CoV-2) Gamma Lineage	Oliveira, E. A.; Simoes e Silva, A. C.; Oliveira, M. C. L.; Colosimo, E. A.; Mak, R. H.; Vasconcelos, M. A.; Miranda, D. M.; Martelli, D. B.; Silva, L. R.; Pinhati, C. C.; Martelli-Junior, H.	2022	wrong age group
COVID-19 situation in Honduras: lessons learned	Fuentes-Barahona, I. C.; Henriquez-MÃ¡rquez, K. I.; MuÃ±oz-Lara, F.; Palou, E.; Alvarado, T.; Lorenzana, I.; Valladares-Rosa, V. M.; Corrales-Alvarez, A. G.; Zambrano, L. I.; Sierra-Santos, M. A.	2021	wrong age group
Surveillance of Acute SARS-CoV-2 Infections in School Children and Point-Prevalence During a Time of High Community Transmission in Switzerland	Kriemler, S.; Ulyte, A.; Ammann, P.; Peralta, G. P.; Berger, C.; Puhan, M. A.; Radtke, T.	2021	wrong age group
Patterns of Presentation of SARS-CoV-2 Infection in Children. Experience at the Italian Epicentre of the Pandemic	Mazza, A.; Di Giorgio, A.; Martelli, L.; Pelliccia, C.; Pinotti, M. A.; Quadri, V.; Verdoni, L.; Decio, A.; Ruggeri, M.; D'Antiga, L.	2021	wrong age group
Clinical course of COVID-19 in children with rheumatic disease under biologic therapy. (Special Issue: Letter to editor rheumatology: commentaries and controversies.)	Demir, F.; Ulu, K.; Cagayan, S.; Coskuner, T.; Sozeri, B.	2021	wrong age group

**Study Title**	**Author names**	**Year**	**Reason for exclusion**
The impact of SARS-CoV-2 infection in children with rheumatic/autoinflammatory diseases on immunosuppressive treatment: a single centre experience	Maritsi, D. N.; Krepis, P.; Vartzelis, G.; Syggelou, A.; Tsolia, M.	2022	wrong age group
Pooled RT-qPCR testing for SARS-CoV-2 surveillance in schools - a cluster randomised trial	Joachim, Alexander; Dewald, Felix; SuÃ¡rez, Isabelle; Zemlin, Michael; Lang, Isabelle; Stutz, Regine; Marthaler, Anna; Bosse, Hans Martin; LÃ¼bke, Nadine; MÃ¼nch, Juliane; Bernard, Marie-Annett; Jeltsch, Kathrin; TÃ¶nshoff, Burkhard; Weidner, Niklas; KrÃ¤usslich, Hans-Georg; Birzele, Lena; HÃ¼bner, Johannes; Schmied, Patricia; Meyer-BÃ¼hn, Melanie; Horemheb-Rubio, Gibran; Cornely, Oliver A.; Haverkamp, Heinz; WiesmÃ¼ller, Gerhard; FÃ¤tkenheuer, Gerd; Hero, Barbara; Kaiser, Rolf; DÃ¶tsch, JÃ¶rg; Rybniker, Jan	2021	wrong age group
Systematic Severe Acute Respiratory Syndrome Coronavirus 2 Screening at Hospital Admission in Children: A French Prospective Multicenter Study	Poline, J.; Gaschignard, J.; Leblanc, C.; Madhi, F.; Foucaud, E.; Nattes, E.; Faye, A.; Bonacorsi, S.; Mariani, P.; Varon, E.; Smati-Lafarge, M.; Caseris, M.; Basmaci, R.; Lachaume, N.; Ouldali, N.	2021	wrong age group
Factors associated with the time to return negative RT-PCR from COVID-19 in paediatric patients: A retrospective cohort study	Shao, J.; Liu, Z.; Ying, X.; Xu, H.; Wang, X.; Huang, Y.; Wang, G.; He, Y.; Chen, J.; Ma, S.; Zou, S.; Cui, Y.; Chen, R.; Lu, J.; Li, X.; Li, Z.; Huang, G.; Wang, W.	2021	wrong age group
COVID-19 in Pediatric Hematopoietic Cell Transplant Recipients: A CIBMTR Study	Bhatt, N. S.; Sharma, A.; St. Martin, A.; Martens, M.; Riches, M. L.; Dandoy, C. E.; Auletta, J. J.	2021	wrong age group
Distinguishing active pediatric COVID-19 pneumonia from MIS-C	Reiff, D. D.; Mannion, M. L.; Samuy, N.; Scalici, P.; Cron, R. Q.	2021	wrong age group
Characteristics and mortality of hospitalized patients with COVID-19 in Iran: a national retrospective cohort study free	Jalili, M.; Payandemehr, P.; Saghaei, A.; Sari, H. N.; Safikhani, H.; Kolivand, P.	2021	wrong age group
SARS-CoV-2-Associated Deaths Among Persons Aged <21 Years - United States, February 12-July 31, 2020	Bixler, D.; Miller, A. D.; Mattison, C. P.; Taylor, B.; Komatsu, K.; Peterson Pompa, X.; Moon, S.; Karmarkar, E.; Liu, C. Y.; Openshaw, J. J.; Plotzker, R. E.; Rosen, H. E.; Alden, N.; Kawasaki, B.; Siniscalchi, A.; Leapley, A.; Drenzek, C.; Tobin-D'Angelo, M.;	2020	wrong age group

Study Title	Author names	Year	Reason for exclusion
	Kauerauf, J.; Reid, H.; Hawkins, E.; White, K.; Ahmed, F.; Hand, J.; Richardson, G.; Sokol, T.; Eckel, S.; Collins, J.; Holzbauer, S.; Kollmann, L.; Larson, L.; Schiffman, E.; Kittle, T. S.; Hertin, K.; Kraushaar, V.; Raman, D.; LeGarde, V.; Kinsinger, L.; Peek-Bullock, M.; Lifshitz, J.; Ojo, M.; Arciuolo, R. J.; Davidson, A.; Huynh, M.; Lash, M. K.; Latash, J.; Lee, E. H.; Li, L.; McGibbon, E.; McIntosh-Beckles, N.; Pouchet, R.; Ramachandran, J. S.; Reilly, K. H.; Dufort, E.; Pulver, W.; Zamcheck, A.; Wilson, E.; de Fijter, S.; Naqvi, O.; Nalluswami, K.; Waller, K.; Bell, L. J.; Burch, A. K.; Radcliffe, R.; Fiscus, M. D.; Lewis, A.; Kolsin, J.; Pont, S.; Salinas, A.; Sanders, K.; Barbeau, B.; Althomsons, S.; Atti, S.; Brown, J. S.; Chang, A.; Clarke, K. R.; Datta, S. D.; Iskander, J.; Leitgeb, B.; Pindyck, T.; Priyamvada, L.; Reagan-Steiner, S.; Scott, N. A.; Viens, L. J.; Zhong, J.; Koumans, E. H.; Pediatric Mortality Investigation, Team		
Detection of SARS-CoV-2 infection in a pediatric population from south Italy without symptoms of Coronavirus Disease 2019	Botti, C.; Maglione, A.; Russo, A.; Micillo, A.; Scognamiglio, G.; Cantile, M.	2021	wrong age group
COVID-19 in pediatric patients undergoing chronic dialysis and kidney transplantation	Canpolat, N.; Yildirim, Z. Y.; Yildiz, N.; Tasdemir, M.; Goknar, N.; Evrengul, H.; Gulmez, R.; Aksu, B.; Dursun, H.; Ozcelik, G.; Yavascan, O.; Cicek, R. Y.; Tulpar, S.; Hacihamdioglu, D. O.; Nayir, A.; Alpay, H.	2022	wrong age group
A Report of 85 Cases of COVID-19 and Abdominal Transplantation From a Single Center: What Are the Associated Factors With Death Among Organ Transplantation Patients	Ali Malekhosseini, S.; Nikoupour, H.; Gholami, S.; Shamsaeefar, A.; Arasteh, P.; Kazemi, K.; Dehghani, M.; Eghlimi, H.; Raeisi Shahraki, H.; Roozbeh, J.; Rezaianzadeh, A.; Nikeghbalian, S.	2021	wrong age group
Clinical Characteristics and Outcome of COVID-19 in Turkish Hematological Malignancy Patients	Civriz Bozdag, S.; Cengiz Seval, G.; Yonal-Hindilerden, I.; Hindilerden, F.; Andic, N.; Baydar, M.; Aydin Kaynar, L.; Toprak, S.; Goksoy, H.; Balik, B.; Demirci, U.; Can, F.; Ozkocaman, V.; Gunduz, E.; Guven, T. Z.; Ozkurt, Z. N.; Demircioglu, S.; Beksac, M.; Ince, I.; Yilmaz, U.; Eroglu Kucukdiler, H.; Abishov, E.; Yavuz, B.; Atas, U.; Mutlu, Y. G.; Bas, V.; Uskudar Teke, H.; Gursoy, V.; Celik, S.;	2021	wrong age group

Study Title	Author names	Year	Reason for exclusion
	Ciftciler, R.; Yagci, M.; Topcuoglu, P.; Ceneli, O.; Abbasov, H.; Selim, C.; Ar, M. C.; Yuce, K. O.; Sadri, S.; Albayrak, C.; Guler, N.; Keklik, M.; Terzi, H.; Dogan, A.; Yegin, A. Z.; Kurt Yuksel, M.; Sogol, S.; Yavasoglu, I.; Bekoz, H.; Aksu, T.; Maral, S.; Erol, V.; Kaynar, L.; Ilhan, O.; Bolaman, A. Z.; Sevindik, G. O.; Akyay, A.; Ozcan, M.; Gurman, G.; Unal, S.; Yavuz, Y.; Diz Kucukkaya, R.; Ozsan, G. H.		
Kinetics of Viral Clearance and Antibody Production Across Age Groups in Children with Severe Acute Respiratory Syndrome Coronavirus 2 Infection	Bahar, B.; Jacquot, C.; Mo, Y. D.; DeBiasi, R. L.; Campos, J.; Delaney, M.	2020	wrong age group
Pediatric multisystem SARS COV2 with versus without cardiac involvement: a multicenter study from Latin America	Pignatelli, R.; Antona, C. V.; Rivera, I. R.; Zenteno, P. A.; Acosta, Y. T.; Huertas-Quinones, M.; Murillo, C. A.; Torres, F. M.; Cabalin, C. F.; Camacho, A. G.; Perez, A. A.; Lombardi, A. B.; Soares, A. M.; Garcia, C. T.; Borges, C. T.; Villalba, C. N.; Lechado, C. R.; Dias, D. T.; Morales, D. A.; Copete, E. M.; Goldenberg, G. L.; Salazar, J. S.; Moreira, J. A.; Asakura, J.; Sabando, K. S.; Branco, K. C.; Rosas, L. T.; Duarte, M. P.; Carbajal, M. J.; Hernandez, M. R.; Martinez, M. M.; Echeverria, N. G.; Caneva, O. M.; Sepulveda, P. R.; Diaz, P. A.; Pluas, R. R.; Alvarado, T. C.; Faundes, L. T.; Diaz, Y. B.; Zachariah, J. P.	2021	wrong age group
Risk factors and outcome of COVID-19 in patients with hematological malignancies	Pinana, J. L.; Martino, R.; Garcia-Garcia, I.; Parody, R.; Morales, M. D.; Benzo, G.; Gomez-Catalan, I.; Coll, R.; De La Fuente, I.; Luna, A.; Merchan, B.; Chinea, A.; de Miguel, D.; Serrano, A.; Perez, C.; Diaz, C.; Lopez, J. L.; Saez, A. J.; Bailen, R.; Zudaire, T.; Martinez, D.; Jurado, M.; Calbacho, M.; Vazquez, L.; Garcia-Cadenas, I.; Fox, L.; Pimentel, A. I.; Bautista, G.; Nieto, A.; Fernandez, P.; Vallejo, J. C.; Solano, C.; Valero, M.; Espigado, I.; Saldana, R.; Sisinni, L.; Ribera, J. M.; Jimenez, M. J.; Trabazo, M.; Gonzalez-Vicent, M.; Fernandez, N.; Talarn, C.; Montoya, M. C.; Cedillo, A.; Sureda, A.; Infectious Complications Subcommittee of the Spanish Hematopoietic Stem Cell, Transplantation; Cell Therapy, Group	2020	wrong age group

Study Title	Author names	Year	Reason for exclusion
Impact of outpatient SARS-CoV-2 infections in minority children	Denny, V.; Shah, N.; Petro, K.; Choksey, K.; DeSantis, E.; Hintz, M.; Rethi, S.; Sanchez, S.; Sylla, B.; Chiu, S.; Gagliardo, C.; Kairam, N.; Nwaobasi-Iwuh, E.; Di Pentima, M. C.	2021	wrong age group
COVID-19 in pediatric kidney transplantation: The Improving Renal Outcomes Collaborative	Varnell, Charles; Harshman, Lyndsay A.; Smith, Laurie; Liu, Chunyan; Chen, Shiran; Al-Akash, Samhar; Barletta, Gina-Marie; Belsha, Craig; Brakeman, Paul; Chaudhuri, Abanti; Fadakar, Paul; Garro, Rouba; Gluck, Caroline; Goebel, Jens; Kershaw, David; Matossian, Debora; Nailescu, Corina; Patel, Hiren P.; Pruette, Cozumel; Ranabothu, Saritha; Rodig, Nancy; Smith, Jodi; Sebestyen VanSickle, Judith; Weng, Patricia; Danziger-Isakov, Lara; Hooper, David K.; Seifert, Michael	2021	wrong age group
Severe Acute Respiratory Syndrome Coronavirus 2 Infections Among Children in the Biospecimens from Respiratory Virus-Exposed Kids (BRAVE Kids) Study	Hurst, J. H.; Heston, S. M.; Chambers, H. N.; Cunningham, H. M.; Price, M. J.; Suarez, L.; Crew, C. G.; Bose, S.; Aquino, J. N.; Carr, S. T.; Griffin, S. M.; Smith, S. H.; Jenkins, K.; Pfeiffer, T. S.; Rodriguez, J.; DeMarco, C. T.; De Naeyer, N. A.; Gurley, T. C.; Louzao, R.; Zhao, C.; Cunningham, C. K.; Steinbach, W. J.; Denny, T. N.; Lugo, D. J.; Moody, M. A.; Permar, S. R.; Rotta, A. T.; Turner, N. A.; Walter, E. B.; Woods, C. W.; Kelly, M. S.	2021	wrong age group
Epidemiology, Clinical Features, and Disease Severity in Patients with Coronavirus Disease 2019 (COVID-19) in a Children's Hospital in New York City, New York	Zachariah, P.; Johnson, C. L.; Halabi, K. C.; Ahn, D.; Sen, A. I.; Fischer, A.; Banker, S. L.; Giordano, M.; Manice, C. S.; Diamond, R.; Sewell, T. B.; Schweickert, A. J.; Babineau, J. R.; Carter, R. C.; Fenster, D. B.; Orange, J. S.; McCann, T. A.; Kernie, S. G.; Saiman, L.	2020	wrong age group
Management of childhood-onset autoinflammatory diseases during the COVID-19 pandemic	Haslak, F.; Yildiz, M.; Adrovic, A.; Sahin, S.; Koker, O.; Aliyeva, A.; Barut, K.; Kasapcopur, O.	2020	wrong age group
Impact of COVID-19 pandemic in natural course of Moyamoya Angiopathy: an experience from tertiary-care-center in India	Das, S.; Ray, B. K.; Ghosh, R.; Sengupta, S.; Pandit, A.; Dubey, S.	2021	wrong age group
COVID-19 in children: analysis of the first pandemic peak in England	Ladhani, S. N.; Amin-Chowdhury, Z.; Davies, H. G.; Aiano, F.; Hayden, I.; Lacy, J.; Sinnathamby, M.; de Lusignan, S.; Demirjian, A.; Whittaker, H.; Andrews, N.; Zambon, M.; Hopkins, S.; Ramsay, M. E.	2020	wrong age group

**Study Title**	**Author names**	**Year**	**Reason for exclusion**
Asthma as a risk factor for hospitalization in children with COVID-19: a nested case-control study	Gaietto, K.; Freeman, M. C.; DicCcco, L. A.; Rauenswinter, S.; Squire, J. R.; Aldewereld, Z.; Iagnemma, J.; Campfield, B. T.; Wolfson, D.; Kazmerski, T. M.; Forno, E.	2022	wrong age group
Thyroid Dysfunction in COVID-19	Dabas, A.; Singh, H.; Goswami, B.; Kumar, K.; Dubey, A.; Jhamb, U.; Yadav, S.; Garg, S.	2021	wrong age group
Severity of COVID-19 in hospitalized patients with and without atopic disease	Timberlake, D. T.; Narayanan, D.; Ogbogu, P. U.; Raveendran, R.; Porter, K.; Scherzer, R.; Prince, B.; Grayson, M. H.	2021	wrong age group
Severe Coronavirus Disease Pneumonia in Pediatric Patients in a Referral Hospital	Ozcan, S.; Emeksiz, S.; Perk, O.; Uyar, E.; Kanik Yuksek, S.	2021	wrong age group
Spectrum of SARS-CoV-2-Related Clinical Syndromes in Children: A Year in the Life	Khan, M.; Dang, L.; Singh, H.; Dalrymple, A.; Miller, A.; Tanios, A.	2022	wrong age group
COVID-19 in children and young adults with kidney disease: risk factors, clinical features and serological response	Weinbrand-Goichberg, J.; Ben Shalom, E.; Rinat, C.; Choshen, S.; Tzvi-Behr, S.; Frishberg, Y.; Becker-Cohen, R.	2022	wrong age group
Epidemiology and prevention strategies of SARS-CoV-2 infection in pediatric hematology and oncology centers in Poland	Styczynski, J.; Balwierz, W.; Wachowiak, J.; Kalwak, K.; Kazanowska, B.; Mlynarski, W.; Dembowska-Baginska, B.; Matysiak, M.; Krawczuk-Rybak, M.; Adamkiewicz-Drozynska, E.; Kowalczyk, J.; Raciborska, A.; Urasinski, T.; Peregud-Pogorzelski, J.; Chaber, R.; Badowska, W.; Karolczyk, G.; Mizia-Malarz, A.; Machnik, K.; Gozdzik, J.; Wysocki, M.; Szczepanski, T.	2020	wrong age group
Severe COVID-19 in uganda across two epidemic phases: A prospective cohort study	Bakamutumaho, B.; Cummings, M. J.; Owor, N.; Kayiwa, J.; Namulondo, J.; Byaruhanga, T.; Muwanga, M.; Nsereko, C.; Rwamutwe, E.; Mutonyi, R.; Achan, J.; Wanyenze, L.; Ndazarwe, A.; Nakanjako, R.; Natuhwera, R.; Nsangi, A.; Bosa, H. K.; Ocom, F.; O'Donnell, M. R.; Kikaire, B.; Lutwama, J. J.	2021	wrong age group
Severe acute respiratory syndrome coronavirus 2 point prevalence among asymptomatic hospitalized children and subsequent healthcare worker evaluation	Patel, A. B.; Clifford, A.; Creaden, J.; Kato, K.; Malakooti, M. R.; Muller, W. J.; Anna, O'Donnell; Reynolds, S.; Richey, K.; Rippe, J.; Wheeler, D. S.; Kociolek, L. K.	2020	wrong age group
Preliminary Clinical and Epidemiological Analysis of the First 1,000 Pediatric COVID-19 Cases in Moscow Region	Meskina, Elena R.	2020	wrong age group

Study Title	Author names	Year	Reason for exclusion
Gender and age factors in COVID-19 patients in Punjab, Pakistan;A cohort study	Sharif, N.; Bukhari, N.; Yousfani, Z. A.; Saleem, A.; Arif, A.; Abbas, H.; Khan, M. A.	2020	wrong age group
Comparison of clinical characteristics of coronavirus disease (COVID-19) and severe acute respiratory syndrome (SARS) as experienced in Taiwan	Su, Yu-Jang; Lai, Yen-Chun	2020	wrong age group
PREVALENCE OF MORTALITY AND ITS DISTRIBUTION BY SEX AND AGE GROUPS IN INDOOR COVID-19 PATIENTS IN D.I.KHAN DIVISION, PAKISTAN	Aamir, M.; Ahmad, W.; Ahmad, B.; Khan, A.; Fawad, M.; Abdullah, M.	2021	wrong age group
Patients assisted at the Department of Medicine of a pediatric hospital at the beginning of the COVID-19 pandemic in Buenos Aires, Argentina	Cairoli, HÃ©ctor; Raiden, Silvina; Chiolo, MarÃa J.; Di Lalla, Sandra; Ferrero, Fernando	2020	wrong age group
A case-control study of the causes of acute respiratory infection among hospitalized patients in Northeastern Laos	Phommasone, K.; Xaiyaphet, X.; Garcia-Rivera, J. A.; Hontz, R. D.; Pathavongsa, V.; Keomoukda, P.; Vongsouvath, M.; Mayxay, M.; Vongsouvath, M.; Newton, P. N.; Ashley, E. A.; Dubot-Peres, A.	2022	wrong age group
Children hospitalized for COVID-19 during the first winter of the pandemic in Buenos Aires, Argentina	Raiden, S.; Cairoli, H.; Potasnik, J.; Di Lalla, S.; Chiolo, M. J.; Torres, F.; Dominguez, P.; Ferrero, F.	2021	wrong age group
COVID-19 outcomes in a large pediatric hematology-oncology center in Houston, Texas	Kamdar, K. Y.; Kim, T. O.; Doherty, E. E.; Pfeiffer, T. M.; Qasim, S. L.; Suell, M. N.; Yates, A. M.; Blaney, S. M.	2021	wrong age group
Characteristics of emergency department patients with COVID-19 at a single site in northern California: clinical observations and public health implications	Duanmu, Y.; Brown, I. P.; Gibb, W. R.; Singh, J.; Matheson, L. W.; Blomkalns, A. L.; Govindarajan, P.	2020	wrong age group
COVID-19 in children with underlying chronic respiratory diseases: survey results from 174 centres	Moeller, A.; Thanikkel, L.; Duijts, L.; Gaillard, E. A.; Garcia-Marcos, L.; Kantar, A.; Tabin, N.; Turner, S.; Zacharasiewicz, A.; Pijnenburg, M. W. H.	2020	wrong age group

Study Title	Author names	Year	Reason for exclusion
COVID-19 in Patients with Hematologic Disorders Undergoing Therapy: Perspective of a Large Referral Hematology Center in Rome	Girmenia, C.; Gentile, G.; Micozzi, A.; Petrucci, L.; Malaspina, F.; D. I. Prima A; Baldacci, E.; Bianchi, S.; Pugliese, P.; Turriziani, O.; Antonelli, G.; Tombolini, V.; Foa, R.; Martelli, M.	2020	wrong age group
Biphasic variation over time in presenting features of patients with COVID-19	Jamal, N.; Whittier, S.; Carter, R. C.; Zachariah, P.	2020	wrong age group
Characterizing coinfection in children with COVID-19: A dual center retrospective analysis	Zhang, D. D.; Acree, M. E.; Ridgway, J. P.; Shah, N.; Hazra, A.; Ravichandran, U.; Kumar, M.	2021	wrong age group
Frequency of Children vs Adults Carrying Severe Acute Respiratory Syndrome Coronavirus 2 Asymptomatically	Milani, G. P.; Bottino, I.; Rocchi, A.; Marchisio, P.; Elli, S.; Agostoni, C.; Costantino, G.	2021	wrong age group
COVID-19 in US Youth Soccer Athletes During Summer 2020	Watson, A. M.; Haraldsdottir, K.; Biese, K. M.; Goodavish, L.; Stevens, B.; McGuine, T. A.	2021	wrong age group
COVID-19 and Inherited Metabolic Disorders: One-Year Experience of a Referral Center	Tummolo, A.; Paterno, G.; Dicintio, A.; Stefanizzi, P.; Melpignano, L.; Arico, M.	2021	wrong age group
Gestation in times of COVID-19 pandemic. Hospital nacional docente Madre Nino San Bartolome, Lima, Peru	Vera Loyola, E. M.; Cruz, I. M.; Cabrejos, V. C.; Pacheco, H. M.; Benitez, M. A.; Chomba, M. P.	2020	wrong age group
Virological Characteristics of Hospitalized Children With SARS-CoV-2 Infection	Pinninti, S. G.; Pati, S.; Poole, C.; Latting, M.; Seleme, M. C.; Yarbrough, A.; Arora, N.; Britt, W. J.; Boppana, S.	2021	wrong age group
The impact of the COVID-19 pandemic on pediatric operations: a retrospective study of Chinese children	Wei, Y.; Yu, C.; Zhao, T. X.; Lin, T.; Dawei, H. E.; Wu, S. D.; Wei, G. H.	2020	wrong age group
Association Between Age and Ethnicity with Pediatric Clinical Outcomes in COVID-2019	Snowden, J.; Patwardhan, A.	2021	wrong age group
A real-world experience of SARS-CoV-2 infection in a tertiary referral centre of MontrÃ©al: Unexpected low prevalence and low mortality	Ruiz, Isaac; Huard, GeneviÃ¨ve; Fournier, Claire; Bissonnette, Julien; Castel, HÃ©lÃ¨ne; Giard, Jeanne-Marie; Villeneuve, Jean-Pierre; Fenyves, Daphna; Marleau, Denis; Willems, Bernard; Corsilli, Daniel;	2021	wrong age group

Study Title	Author names	Year	Reason for exclusion
	Correal, Florence; Ferreira, Victor; Martel, Dominic; Mathieu, Alexandre; Vincent, Catherine; Bilodeau, Marc		
Acute Morbidity and Mortality Analysis of COVID-19 in Children Receiving Cancer Treatment	Shaheen, N.; Wali, R. M.; Saeed, H.; Sandhu,, II; Qaisar, M.; Qazi, R.	2021	wrong age group
Heart Disease, Advanced Age, Minority Race, and Hispanic Ethnicity Are Associated With Mortality in COVID-19 Patients	Conway, B. J.; Kim, J. W.; Brousseau, D. C.; Conroy, M.	2021	wrong age group
Comparison of Leukocyte, Neutrophil, and Lymphocyte Levels in Child Patients that Experienced Breathlessness with COVID-19 and Non-COVID-19 (2020â€“2021) in Medan, Indonesia	Wijaya, H.; Pitaloka, A.; Amelia, R.; Lubis, I.	2022	wrong age group
Characteristics of Hospitalized Children Positive for SARS-CoV-2: Experience of a Large Center	Webb, N. E.; Osburn, T. S.	2021	wrong age group
Characteristics of Children Diagnosed With SARS-CoV-2 in the Ambulatory Setting	Guo, N.; Crim, K.; Foote, S.; Batra, B.; Parrish, C.; Crocetti, M.	2022	wrong age group
Clinical and epidemiological characteristics of pediatric and adolescent patients of COVID-19, an experience of a Divisional Corona Center, Bahawalpur from South Punjab, Pak	Hammad, Ali Naeem Muhammad Anwar Hafiz Muhammad Ahmad Ameer	2021	wrong age group
Severe acute respiratory syndrome coronavirus 2 RNAemia and clinical outcomes in children with coronavirus disease 2019	Mertz, C.; Glowinski, R.; Cohen, S. H.; Mertz, S.; Ye, Fang; Hall, M. W.; Peeples, M. E.; King, T.; Wang, HuanYu; Leber, A. L.; Sanchez, P. J.; Ramilo, O.; Mejias, A.	2021	wrong age group
Severe Acute Respiratory Syndrome Coronavirus 2 Clinical Syndromes and Predictors of Disease Severity in Hospitalized Children and Youth	Fernandes, D. M.; Oliveira, C. R.; Guerguis, S.; Eisenberg, R.; Choi, J.; Kim, M.; Abdelhemid, A.; Agha, R.; Agarwal, S.; Aschner, J. L.; Avner, J. R.; Ballance, C.; Bock, J.; Bhavsar, S. M.; Campbell, M.; Clouser, K. N.; Gesner, M.; Goldman, D. L.; Hammerschlag, M. R.;	2021	wrong age group

**Study Title**	**Author names**	**Year**	**Reason for exclusion**
	Hymes, S.; Howard, A.; Jung, H. J.; Kohlhoff, S.; Kojaoghlanian, T.; Lewis, R.; Nachman, S.; Naganathan, S.; Paintsil, E.; Pall, H.; Sy, S.; Wadowski, S.; Zirinsky, E.; Cabana, M. D.; Herold, B. C.; Tri-State Pediatric, Covid-Research Consortium		
Comparison of clinical features and laboratory findings of coronavirus disease 2019 and influenza A and B infections in children: A single-center study	Siddiqui, M.; Gltekingil, A.; Bakirci, O.; Uslu, N.; Baskin, E.	2021	wrong age group
Influenza vs. COVID-19: Comparison of Clinical Characteristics and Outcomes in Pediatric Patients in Mexico City	Laris-Gonzalez, A.; Aviles-Robles, M.; Dominguez-Barrera, C.; Parra-Ortega, I.; Sanchez-Huerta, J. L.; Ojeda-Diezbarroso, K.; Bonilla-Pellegrini, S.; Olivar-Lopez, V.; Chavez-Lopez, A.; Jimenez-Juarez, R.	2021	wrong age group
The utility of paired upper and lower COVID-19 sampling in patients with artificial airways	Kitt, E.; Davis, D. H.; Kerman, C.; Sammons, J. S.; Handy, L. K.; Gallagher, E.; O'Callaghan, K.; Harris, R. M.; Coffin, S. E.; Bell, L. M.; Chiotos, K.	2021	wrong age group
30-Day morbidity and mortality of bariatric metabolic surgery in adolescence during the COVID-19 pandemic - The GENEVA study	Singhal, R.; Super, J.; Alqahtani, A.; Nadler, E. P.; Ludwig, C.; Tahrani, A.; Mahawar, K.; Pedziwiatr, M.; Major, P.; Zarzycki, P.; Pantelis, A.; Lapatsanis, D. P.; Stravodimos, G.; Matthys, C.; Focquet, M.; Vleeschouwers, W.; Spaventa, A. G.; Zerrweck, C.; Vitiello, A.; Berardi, G.; Musella, M.; Sanchez-Meza, A.; Cantu, F. J.; Mora, F.; Cantu, M. A.; Katakwar, A.; Reddy, D. N.; Elmaleh, H.; Hassan, M.; Elghandour, A.; Elbanna, M.; Khan, A.; Layani, L.; Kiran, N.; Velikorechin, A.; Solovyeva, M.; Melali, H.; Shahabi, S.; Agrawal, A.; Shrivastava, A.; Sharma, A.; Narwaria, B.; Narwaria, M.; Raziel, A.; Sakran, N.; Susmallian, S.; Karagoz, L.; Akbaba, M.; Piskin, S. Z.; Ziya, A.; Senol, Z.; Manno, E.; Iovino, M. G.; Osman, A.; Qassem, M.; Arana-Garza, S.; Povoas, H. P.; Vilas-Boas, M. L.; Naumann, D.; Li, A.; Ammori, B. J.; Balamoun, H.; Salman, M.; Nasta, A. M.; Goel, R.; Sanchez-Aguilar, H.; Herrera, M. F.; Abou-Mrad, A.; Cloix, L.; Mazzini, G. S.; Kristem, L.; Lazaro, A.; Campos, J.; Bernardo, J.; Gonzalez, J.; Trindade, C.; Viveiros, O.; Ribeiro, R.; Goitein, D.; Hazzan, D.; Segev, L.; Beck, T.; Reyes, H.; Monterrubio, J.; Garcia, P.; Benois, M.; Kassir, R.; Contine, A.; Elshafei, M.; Aktas, S.;	2021	wrong age group

Study Title	Author names	Year	Reason for exclusion
	Weiner, S.; Heidsieck, T.; Level, L.; Pinango, S.; Ortega, P. M.; Moncada, R.; Valenti, V.; Vlahovic, I.; Boras, Z.; Liagre, A.; Martini, F.; Juglard, G.; Motwani, M.; Saggu, S. S.; Al Momani, H.; Lopez, L. A. A.; Cortez, M. A. C.; Zavala, R. A.; D'Haese, C.; Kempeneers, I.; Himpens, J.; Lazzati, A.; Paolino, L.; Bathaei, S.; Bedirli, A.; Yavuz, A.; Buyukkasap, C.; Ozaydin, S.; Kwiatkowski, A.; Bartosiak, K.; Waledziak, M.; Santonicola, A.; Angrisani, L.; Iovino, P.; Palma, R.; Iossa, A.; Boru, C. E.; De Angelis, F.; Silecchia, G.; Hussain, A.; Balchandra, S.; Coltell, I. B.; Perez, J. L.; Bohra, A.; Awan, A. K.; Madhok, B.; Leeder, P. C.; Awad, S.; Al-Khyatt, W.; Shoma, A.; Elghadban, H.; Ghareeb, S.; Mathews, B.; Kurian, M.; Larentzakis, A.; Vrakopoulou, G. Z.; Albanopoulos, K.; Bozdag, A.; Lale, A.; Kirkil, C.; Dincer, M.; Bashir, A.; Haddad, A.; Hijleh, L. A.; Zilberstein, B.; de Marchi, D. D.; Souza, W. P.; Broden, C. M.; Gislason, H.; Shah, K.; Ambrosi, A.; Pavone, G.; Tartaglia, N.; Kona, S. L. K.; Kalyan, K.; Perez, C. E. G.; Botero, M. A. F.; Covic, A.; Timofte, D.; Maxim, M.; Faraj, D.; Tseng, L.; Liem, R.; Oren, G.; Dilektasli, E.; Yalcin, I.; AlMukhtar, H.; Al Hadad, M.; Mohan, R.; Arora, N.; Bedi, D.; Rives-Lange, C.; Chevallier, J. M.; Poghosyan, T.; Sebbag, H.; Zinai, L.; Khaldi, S.; Mauchien, C.; Mazza, D.; Dinescu, G.; Rea, B.; Perez-Galaz, F.; Zavala, L.; Besa, A.; Curell, A.; Balibrea, J. M.; Vaz, C.; Galindo, L.; Silva, N.; Caballero, J. L. E.; Sebastian, S. O.; Marchesini, J. C. D.; da Fonseca Pereira, R. A.; Sobottka, W. H.; Fiolo, F. E.; Turchi, M.; Coelho, A. C. J.; Zacaron, A. L.; Barbosa, A.; Quinino, R.; Menaldi, G.; Paleari, N.; Martinez-Duartez, P.; Aragon Ramirez de Esparza, D. G. M.; Esteban, V. S.; Garcia-Galocha, J. L.; Josa, M. I.; Pacheco-Garcia, J. M.; Mayo-Ossorio, M. A.; Chowbey, P.; Soni, V.; de Vasconcelos Cunha, H. A.; Castilho, M. V.; Ferreira, R. M. A.; Barreiro, T. A.; Charalabopoulos, A.; Sdralis, E.; Davakis, S.; Bomans, B.; Dapri, G.; Van Belle, K.; MazenTakieddine,; Vaneukem, P.; Karaca, E. S. A.; Karaca, F. C.; Sumer, A.; Peksen, C.; Savas, O. A.; Chousleb, E.; Elmokayed, F.; Fakhereldin, I.; Aboshanab, H. M.; Swelium, T.; Gudal, A.; Gamloo,		

Study Title	Author names	Year	Reason for exclusion
	L.; Ugale, A.; Ugale, S.; Boeker, C.; Reetz, C.; Hakami, I. A.; Mall, J.; Alexandrou, A.; Baili, E.; Bodnar, Z.; Maleckas, A.; Gudaityte, R.; Guldogan, C. E.; Gundogdu, E.; Ozmen, M. M.; Thakkar, D.; Dukkipati, N.; Shah, P. S.; Shah, S. S.; Jambulingam, P.; Mamidanna, R.; Whitelaw, D.; Adil, M. T.; Jain, V.; Veetil, D. K.; Wadhawan, R.; Torres, A.; Torres, M.; Tinoco, T.; Leclercq, W.; Romeijn, M.; van de Pas, K.; Alkhazraji, A. K.; Taha, S. A.; Ustun, M.; Yigit, T.; Inam, A.; Burhanulhaq, M.; Pazouki, A.; Eghbali, F.; Kermansaravi, M.; Jazi, A. H. D.; Mahmoudieh, M.; Mogharehabed, N.; Tsiotos, G.; Stamou, K.; Barrera Rodriguez, F. J.; Rojas Navarro, M. A.; Torres, O. M. O.; Martinez, S. L.; Tamez, E. R. M.; Millan Cornejo, G. A.; Flores, J. E. G.; Mohammed, D. A.; Elfawal, M. H.; Shabbir, A.; Guowei, K.; So, J. B. Y.; Kaplan, E. T.; Kaplan, M.; Kaplan, T.; Pham, D. T.; Rana, G.; Kappus, M.; Gadani, R.; Kahitan, M.; Pokharel, K.; Osborne, A.; Pournaras, D.; Hewes, J.; Napolitano, E.; Chiappetta, S.; Bottino, V.; Dorado, E.; Schoettler, A.; Gaertner, D.; Fedtke, K.; Aguilar-Espinosa, F.; Aceves-Lozano, S.; Balani, A.; Nagliati, C.; Pennisi, D.; Rizzi, A.; Frattini, F.; Foschi, D.; Benuzzi, L.; Parikh, C. H. I. R. A. G.; Shah, H. A. R. S. H. I. L.; Pinotti, E.; Montuori, M.; Borrelli, V.; Dargent, J.; Copaescu, C. A.; Hutopila, I.; Smeu, B.; Witteman, B.; Hazebroek, E.; Deden, L.; Heusschen, L.; Okkema, S.; Aufenacker, T.; den Hengst, W.; Vening, W.; van der Burgh, Y.; Ghazal, A.; Ibrahim, H.; Niazi, M.; Alkhaffaf, B.; Altarawni, M.; Cesana, G. C.; Anselmino, M.; Uccelli, M.; Olmi, S.; Stier, C.; Akmanlar, T.; Sonnenberg, T.; Schieferbein, U.; Marcolini, A.; Awruch, D.; Vicentin, M.; de Souza Bastos, E. L.; Gregorio, S. A.; Ahuja, A.; Mittal, T.; Bolckmans, R.; Wiggins, T.; Baratte, C.; Wisnewsky, J. A.; Genser, L.; Ward, S.; Chong, L.; Taylor, L.; Hi, M. W.; Plamper, A.; Rheinwalt, K.; Heneghan, H.; Geoghegan, J.; Ng, K. C.; Fearon, N.; Kaseja, K.; Kotowski, M.; Samarkandy, T. A.; Leyva-Alvizo, A.; Corzo-Culebro, L.; Wang, C.; Yang, W.; Dong, Z.; Riera, M.; Jain, R.; Hamed, H.; Said, M.; Zarzar, K.; Garcia, M.; Turkcapar, A. G.; Sen, O.; Baldini, E.; Conti, L.; Wietzycoski, C.; Lopes, E.; Pintar, T.; Salobir, J.; Aydin,		

**Study Title**	**Author names**	**Year**	**Reason for exclusion**
	C.; Atici, S. D.; Ergin, A.; Ciyiltepe, H.; Bozkurt, M. A.; Kizilkaya, M. C.; Onalan, N. B. D.; Zuber, M. N. B. A.; Wong, W. J.; Garcia, A.; Vidal, L.; Beisani, M.; Pasquier, J.; Vilallonga, R.; Sharma, S.; Parmar, C.; Lee, L.; Sufi, P.; Sinan, H.; Saydam, M.		
High prevalence of sars-cov-2 genetic variation and d614g mutation in pediatric patients with covid-19	Pandey, U.; Yee, R.; Shen, L.; Judkins, A. R.; Bootwalla, M.; Ryutov, A.; Maglinte, D. T.; Ostrow, D.; Precit, M.; Biegel, J. A.; Bender, J. M.; Gai, X.; Dien Bard, J.	2021	wrong age group
International Analysis of Electronic Health Records of Children and Youth Hospitalized With COVID-19 Infection in 6 Countries	Bourgeois, Florence T.; GutiÃ©rrez-SacristÃ¡n, Alba; Keller, Mark S.; Liu, Molei; Hong, Chuan; Bonzel, Clara-Lea; Tan, Amelia L. M.; Aronow, Bruce J.; Boeker, Martin; Booth, John; Cruz Rojo, Jaime; Devkota, Batsal; GarcÃa Barrio, Noelia; Gehlenborg, Nils; Geva, Alon; Hanauer, David A.; Hutch, Meghan R.; Issitt, Richard W.; Klann, Jeffrey G.; Luo, Yuan; Mandl, Kenneth D.; Mao, Chengsheng; Moal, Bertrand; Moshal, Karyn L.; Murphy, Shawn N.; Neuraz, Antoine; Ngiam, Kee Yuan; Omenn, Gilbert S.; Patel, Lav P.; JimÃ©nez, Miguel Pedrera; Sebire, Neil J.; Balazote, Pablo Serrano; Serret-Larmande, Arnaud; South, Andrew M.; Spiridou, Anastasia; Taylor, Deanne M.; Tippmann, Patric; Visweswaran, Shyam; Weber, Griffin M.; Kohane, Isaac S.; Cai, Tianxi; Avillach, Paul	2021	wrong age group
COVID-19 in children treated with immunosuppressive medication for kidney diseases	Marlais, M.; Wlodkowski, T.; Al-Akash, S.; Ananin, P.; Bandi, V. K.; Baudouin, V.; Boyer, O.; Vasquez, L.; Govindan, S.; Hooman, N.; Ijaz, I.; Loza, R.; Melgosa, M.; Pande, N.; Pape, L.; Saha, A.; Samsonov, D.; Schreuder, M. F.; Sharma, J.; Siddiqui, S.; Sinha, R.; Stewart, H.; Tasic, V.; Tonshoff, B.; Twombley, K.; Upadhyay, K.; Vivarelli, M.; Weaver, D. J.; Woroniecki, R.; Schaefer, F.; Tullus, K.	2020	wrong age group
How COVID-19 Pandemic Changed Children and Adolescents Use of the Emergency Department: the Experience of a Secondary Care Pediatric Unit in Central Italy	Vierucci, F.; Bacci, C.; Mucaria, C.; Dini, F.; Federico, G.; Maielli, M.; Vaccaro, A.	2020	wrong age group
Clinical and Immune Features of Hospitalized Pediatric Patients With	Wu, H.; Zhu, H.; Yuan, C.; Yao, C.; Luo, W.; Shen, X.; Wang, J.; Shao, J.; Xiang, Y.	2020	wrong age group

**Study Title**	**Author names**	**Year**	**Reason for exclusion**
Coronavirus Disease 2019 (COVID-19) in Wuhan, China			
Organizational aspects of pediatric anesthesia and surgery between two waves of Covid-19	Camporesi, A.; Melloni, G. E. M.; Diotto, V.; Bertani, P.; La Pergola, E.; Pelizzo, G.	2021	wrong age group
Multisystem Inflammatory Syndrome in Children-United States, February 2020-July 2021	Miller, A. D.; Zambrano, L. D.; Yousaf, A. R.; Abrams, J. Y.; Meng, L.; Wu, M. J.; Melgar, M.; Oster, M. E.; Godfred Cato, S. E.; Belay, E. D.; Campbell, A. P.	2021	wrong age group
Comparison of Clinical Features of COVID-19 vs Seasonal Influenza A and B in US Children	Song, X.; Delaney, M.; Shah, R. K.; Campos, J. M.; Wessel, D. L.; DeBiasi, R. L.	2020	wrong age group
Is cardiorespiratory disease associated with increased susceptibility of SARS-CoV-2 in children?	Berry, C. du; Saunders, T.; McMinn, A.; Tosif, S.; Shanthikumar, S.; Vandeleur, M.; Harrison, J.; Burgner, D.; Ranganathan, S.; Crawford, N.; Wurzel, D.	2021	wrong age group
Comparative study of hospitalized children with acute respiratory distress syndrome caused by SARS-CoV-2 and influenza virus	Liu, X.; Li, W.; Zhang, B.; Guo, Y.; Hu, Z.; Peng, C.; Lei, X.; Luo, Q.; Zhang, Q.; Deng, W.; Wang, J.; Tang, J.; Li, Y.; Chen, J.	2021	wrong age group
National Trends of Cases of COVID-19 in Children Based on US State Health Department Data	Sisk, B.; Cull, W.; Harris, J. M.; Rothenburger, A.; Olson, L.	2020	wrong age group
Effect of influenza vaccine on COVID-19 mortality: a retrospective study	Candelli, M.; Pignataro, G.; Torelli, E.; Gulli, A.; Nista, E. C.; Petrucci, M.; Saviano, A.; Marchesini, D.; Covino, M.; Ojetti, V.; Antonelli, M.; Gasbarrini, A.; Franceschi, F.	2021	wrong age group
Characterization of COVID-19 disease in pediatric oncology patients: The New York-New Jersey regional experience	Madhusoodhan, P. P.; Pierro, J.; Musante, J.; Kothari, P.; Gampel, B.; Appel, B.; Levy, A.; Tal, A.; Hogan, L.; Sharma, A.; Feinberg, S.; Kahn, A.; Pinchinat, A.; Bhatla, T.; Glasser, C. L.; Satwani, P.; Raetz, E. A.; Onel, K.; Carroll, W. L.	2021	wrong age group
Adolescents as partners in the fight against COVID-19	Kest, H.; Kaushik, A.; Jagunla, A.; Shaheen, S.; Zaveri, S.; Fernandez, N.; Gupta, S.; Goldberg, D.	2021	wrong age group
Pediatric Severe Acute Respiratory Syndrome Coronavirus 2 (SARS-CoV-2):	Yonker, L. M.; Neilan, A. M.; Bartsch, Y.; Patel, A. B.; Regan, J.; Arya, P.; Gootkind, E.; Park, G.; Hardcastle, M.; St John, A.; Appleman, L.; Chiu, M. L.; Fialkowski, A.; De la Flor, D.; Lima, R.;	2020	wrong age group

**Study Title**	**Author names**	**Year**	**Reason for exclusion**
Clinical Presentation, Infectivity, and Immune Responses	Bordt, E. A.; Yockey, L. J.; D'Avino, P.; Fischinger, S.; Shui, J. E.; Lerou, P. H.; Bonventre, J. V.; Yu, X. G.; Ryan, E. T.; Bassett, I. V.; Irimia, D.; Edlow, A. G.; Alter, G.; Li, J. Z.; Fasano, A.		
Comparative analysis of two molecular tests for the detection of covid-19 in Cameroon	Ngaba, G. P.; Kalla, G. C. M.; Assob, J. C. N.; Njouendou, A. J.; Jembe, C. N.; Mboudou, E. T.; Mbopi-Keou, F. X.	2021	wrong age group
Pre-surgical screening and incidence of COVID-19 infection at the shriners hospitals for children - Honolulu	Fujimoto, D.; Blair, B.; Miyamoto, Robin H.	2021	wrong age group
Pooled SARS-CoV-2 antigen tests in asymptomatic children and their caregivers: Screening for SARS-CoV-2 in a pediatric emergency department	Reichert, F.; Enninger, A.; Plecko, T.; Zoller, W. G.; Paul, G.	2021	wrong age group
Prevalence of COVID-19 in adolescents and youth compared with older adults in states experiencing surges	Rumain, B.; Schneiderman, M.; Geliebter, A.	2021	wrong age group
Paediatric burns epidemiology during COVID-19 pandemic and 'stay home' era	D'Asta, F.; Choong, J.; Thomas, C.; Adamson, J.; Wilson, Y.; Wilson, D.; Moiemen, N.; Farroha, A.	2020	wrong age group
Differences among Severe Cases of Sars-CoV-2, Influenza, and Other Respiratory Viral Infections in Pediatric Patients: Symptoms, Outcomes and Preexisting Comorbidities	Sousa, B. L. A.; Sampaio-Carneiro, M.; de Carvalho, W. B.; Silva, C. A.; Ferraro, A. A.	2020	wrong age group
Hospitalized Children with Critical SARS-CoV-2 Infection Cared for in a Limited Resource Setting: Multicenter Cohort Study	de Fariasa, Emmerson C. F.; Piva, Jefferson Junior Manoel J. C. PavÃ£o Sales Susan C. D.; Nascimento, Luciana M. P.; PavÃ£o, Dalila C. A.; Santos, Valeria T. S. dos Pinheiroa Andressa H. O.; Alves, MarÃlia C. B.; Mello, Mary L. F. M. F.; Carvalho, Patricia Justino Maria C. A.; Clemente, Gleice Terreri Maria Teresa	2021	wrong age group
Clinical Picture and Risk Factors of Severe Respiratory Symptoms in COVID-19 in Children	Mania, A.; Faltin, K.; Mazur-Melewska, K.; Malecki, P.; Jonczyk-Potoczna, K.; Lubarski, K.; Lewandowska, Z.; Cwalinska, A.; Rosada-Kurasinska, J.; Bartkowska-Sniatkowska, A.; Figlerowicz, M.	2021	wrong age group

**Study Title**	**Author names**	**Year**	**Reason for exclusion**
Clinical Characteristics and Outcomes of COVID-19 in Turkish Patients with Hematological Malignancies	Civriz Bozdag, S.; Cengiz Seval, G.; Yonal Hindilerden, I.; Hindilerden, F.; Andic, N.; Baydar, M.; Aydin Kaynar, L.; Toprak, S. K.; Goksoy, H. S.; Balik Aydin, B.; Demirci, U.; Can, F.; Ozkocaman, V.; Gunduz, E.; Guven, Z. T.; Ozkurt, Z. N.; Demircioglu, S.; Beksac, M.; Ince, I.; Yilmaz, U.; Eroglu Kucukdiler, H.; Abishov, E.; Yavuz, B.; Atas, U.; Mutlu, Y. G.; Bas, V.; Ozkalemkas, F.; Uskudar Teke, H.; Gursoy, V.; Celik, S.; Ciftciler, R.; Yagci, M.; Topcuoglu, P.; Ceneli, O.; Abbasov, H.; Selim, C.; Ar, M. C.; Yucel, O. K.; Sadri, S.; Albayrak, C.; Demir, A. M.; Guler, N.; Keklik, M.; Terzi, H.; Dogan, A.; Yegin, Z. A.; Kurt Yuksel, M.; Sadri, S.; Yavasoglu, I.; Bekoz, H. S.; Aksu, T.; Maral, S.; Erol, V.; Kaynar, L.; Ilhan, O.; Bolaman, A. Z.; Sevindik, O. G.; Akyay, A.; Ozcan, M.; Gurman, G.; Unal, S.; Yavuz, Y.; Diz Kucukkaya, R.; Ozsan, G. H.	2022	wrong age group
Reported COVID-19 Incidence in Wisconsin High School Athletes in Fall 2020	Sasser, P.; McGuine, T.; Haraldsdottir, K.; Biese, K.; Goodavish, L.; Stevens, B.; Watson, A. M.	2021	wrong age group
Symptomatology and racial disparities among children undergoing universal preoperative COVID-19 screening at three US children's hospitals: Early pandemic through resurgence	Adler, A. C.; Shah, A. S.; Blumberg, T. J.; Fazal, F. Z.; Chandrakantan, A.; Ellingsen, K.; Nathanson, B. H.; Lin, E. E.	2021	wrong age group
Prevalence and mortality in beta-thalassaemias due to outbreak of novel coronavirus disease (COVID-19): the nationwide Iranian experience	Karimi, M.; Haghpanah, S.; Azarkeivan, A.; Zahedi, Z.; Zarei, T.; Tavakoli, M. A.; Bazrafshan, A.; Shirkavand, A.; Sanctis, V. de	2020	wrong age group
Clinical utility of targeted SARS-CoV-2 serology testing to aid the diagnosis and management of suspected missed, late or post-COVID-19 infection syndromes: results from a pilot service implemented during the first pandemic wave	Sweeney, N.; Merrick, B.; Galao, R. P.; Pickering, S.; Botgros, A.; Wilson, H. D.; Signell, A. W.; Betancor, G.; Tan, KiaIk; Ramble, J.; Kouphou, N.; Acors, S.; Graham, C.; Seow, J.; MacMahon, E.; Neil, S. J. D.; Malim, M. H.; Doores, K.; Douthwaite, S.; Batra, R.; Nebbia, G.; Edgeworth, J. D.	2021	wrong age group

Study Title	Author names	Year	Reason for exclusion
Changes in Cancer Management due to COVID-19 Illness in Patients with Cancer in Northern California	Wu, J. T.; Kwon, D. H.; Glover, M. J.; Henry, S.; Wood, D.; Rubin, D. L.; Koshkin, V. S.; Schapira, L.; Shah, S. A.	2021	wrong age group
Characteristics and risk factors associated with critical illness in pediatric COVID-19	Fisler, Grace; Izard, Stephanie M.; Shah, Sareen; Lewis, Deirdre; Kainth, Mundeep K.; Hagmann, Stefan H. F.; Belfer, Joshua A.; Feld, Lance M.; Mastroianni, Fiore; Kvasnovsky, Charlotte L.; Capone, Christine A.; Schneider, James; Sweberg, Todd; Schleien, Charles; Taylor, Matthew D.	2020	wrong age group
Prevalence of COVID-19 in children affected by allergic rhinoconjunctivitis and asthma: results from the second "SIAIP rhinosinusitis and conjunctivitis committee" survey	Brindisi, G.; Zicari, A. M.; Parisi, G. F.; Diaferio, L.; Indolfi, C.; Marchese, G.; Ghiglioni, D. G.; Umano, G. R.; Klain, A.; Marseglia, G. L.; Del Giudice, M. M.	2022	wrong age group
The epidemiological and trending pattern of ncovid-19 in the state of Rajasthan, India	Sudhir, Bhandari; Ajeet, Singh; Subrata, Banerjee; Raman, Sharma; Govind, Rankawat; Vishal, Gupta; Prakash, Keswani; Ashwin, Mathur; Abhishek, Agarwal; Shrikant, Sharma; Pd, Meena	2020	wrong age group
Basrah Preliminary Experience With COVID-19: A Report on 6404 Patients	Hammadi, S.; AlKanan, A. K.; Fares, M.; Mohammed, N. K.; Hashim, A. R.; Habeeb, A.; Mansour, A. A.	2021	wrong age group
Anesthetic outcomes in pediatric patients with COVID-19: A matched cohort study	Cronin, J. A.; Nelson, J. H.; Farquhar, I.; Braffett, B.; Bebu, I.; Pestieau, S. R.; Geng-Ramos, G.; Heitmiller, E.; Deutsch, N.	2021	wrong age group
Racial and/or ethnic and socioeconomic disparities of SARS-CoV-2 infection among children	Goyal, M. K.; Simpson, J. N.; Boyle, M. D.; Badolato, G. M.; Delaney, M.; McCarter, R.; Cora-Bramble, D.	2020	wrong age group
Contribution of Serological Rapid Diagnostic Tests to the Strategy of Contact Tracing in Households Following SARS-CoV-2 Infection Diagnosis in Children	Charbonnier, L.; Roupret-Serzec, J.; Caseris, M.; Danse, M.; Cointe, A.; Cohen, L.; Faye, A.; Ouldali, N.; Gaschignard, J.	2021	wrong age group
Impact of COVID-19 in Immunosuppressed Children With Neuroimmunologic Disorders	Olive-Cirera, G.; Fonseca, E.; Cantarin-Extremera, V.; Vazquez-Lopez, M.; Jimenez-Legido, M.; Gonzalez-Alvarez, V.; Ribeiro-Constante, J.; Camacho-Salas, A.; Marti, I.; Cancho-Candela, R.; Martinez-Gonzalez, M. J.; Saiz, A.; Armangue, T.	2022	wrong age group

Study Title	Author names	Year	Reason for exclusion
Prevalence of asthma in hospitalized and non-hospitalized children with COVID-19	Floyd, G. C.; Dudley, J. W.; Xiao, R.; Feudtner, C.; Taquechel, K.; Miller, K.; Henrickson, S. E.; Hill, D. A.; Kenyon, C. C.	2021	wrong age group
Outcome of Children Admitted With SARS-CoV-2 Infection: Experiences From a Pediatric Public Hospital	Rao, S.; Gavali, V.; Prabhu, S. S.; Mathur, R.; Dabre, L. R.; Prabhu, S. B.; Bodhanwala, M.	2021	wrong age group
Clinical profile and outcome of COVID-19 among immunocompromised children	Rao, S. K.; Ashutosh, Kumar; Rajniti, Prasad; Vineeta, Gupta; Mishra, O. P.	2021	wrong age group
Impact of Social Activity Restriction and Routine Patient Screening as Preventive Measurement for Tertiary Referral Hospital Working Staff in Countries with High Incidence Covid-19 Cases	Adrizain, Riyadi Jubaedah Siti Fitriany Eva Nursanty Wicaksana Rudi Hartantri Yovita Prihatini Delita Turbawati Dewi Kartika Andriyoko Basti Ramdan Ahmad Rachman Iwan Abdul Sudiro Melati Lasminingrum Lina	2021	wrong age group
Human respiratory viruses, including SARS-CoV-2, circulating in the winter season 2019-2020 in Parma, Northern Italy	Calderaro, A.; De Conto, F.; Buttrini, M.; Piccolo, G.; Montecchini, S.; Maccari, C.; Martinelli, M.; Di Maio, A.; Ferraglia, F.; Pinardi, F.; Montagna, P.; Arcangeletti, M. C.; Chezzi, C.	2021	wrong age group
Clinical course of COVID-19 infection in paediatric familial Mediterranean fever patients	Kaya Akca, Ummusen; Sener, Seher; Balik, Zeynep; Gurlevik, Sibel; Oygar, Pembe Derin; Atalay, Erdal; Cuceoglu, Muserref Kasap; Basaran, Ozge; Batu, Ezgi Deniz; Teksam, Ozlem; Bilginer, Yelda; Ozsurekci, Yasemin; Ozen, Seza	2021	wrong age group
COVID-19 in Children with Cancer in New York City	Boulad, F.; Kamboj, M.; Bouvier, N.; Mauguen, A.; Kung, A. L.	2020	wrong age group
Low prevalence of asthma in Mexican children and adults with a positive rtRT-PCR test for SARS-CoV-2: a cross-sectional study during the 2020 pandemic	Bedolla-Barajas, M.; Morales-Romero, J.; Bedolla-Pulido, T. R.; Meza-Lopez, C.; Robles-Figueroa, M.; Pulido-Guillen, N. A.; Orozco-Alatorre, L. G.; Andrade-Castellanos, C. A.	2021	wrong age group
COVID-19 in a subset of hospitalized children in Israel	Ben-Shimol, S.; Livni, G.; Megged, O.; Greenberg, D.; Danino, D.; Youngster, I.; Shachor-Meyouhas, Y.; Dabaja-Younis, H.; Scheuerman, O.; Mor, M.; Somekh, E.; Hanna, H. Y.; Givon-Lavi, N.; Guri, A.; Leibovitz, E.; Alkan, Y.; Grupel, D.; Rubinstein, U.; Zeev, Z. S. B.; Bamberger, E.; Kuperman, A. A.; Grisaru-Soen, G.; Tasher, D.; Gottesman, G.; Glikman, D.; Stein, M.	2021	wrong diagnostic test
Criteria for referral of pediatric SARS-CoV-2 infection: a real-life experience in the pandemic era	Montagnani, C.; Venturini, E.; L'Erario, M.; Tersigni, C.; Bortone, B.; Bianchi, L.; Menegazzo, F.; Indolfi, G.; Chiappini, E.; Galli, L.	2020	wrong diagnostic test

Study Title	Author names	Year	Reason for exclusion
Worse impact of second wave COVID-19 pandemic in adults but not in children with inflammatory bowel disease: an Italian single tertiary center experience	Carparelli, S.; Pastore, M. R.; Valvano, M. R.; Marseglia, A.; Latiano, A.; Palmieri, O.; Guerra, M.; Martino, G.; Perri, F.; Bossa, F.	2021	wrong diagnostic test
Prevalence and Risk Factors of Neurologic Manifestations in Hospitalized Children Diagnosed with Acute SARS-CoV-2 or MIS-C	Fink, E. L.; Robertson, C. L.; Wainwright, M. S.; Roa, J. D.; Lovett, M. E.; Stulce, C.; Yacoub, M.; Potera, R. M.; Zivick, E.; Holloway, A.; Nagpal, A.; Wellnitz, K.; Czech, T.; Even, K. M.; Brunow de Carvalho, W.; Rodriguez, I. S.; Schwartz, S. P.; Walker, T. C.; Campos-Mino, S.; Dervan, L. A.; Geneslaw, A. S.; Sewell, T. B.; Pryce, P.; Silver, W. G.; Lin, J. E.; Vargas, W. S.; Topjian, A.; Alcamo, A. M.; McGuire, J. L.; Dominguez Rojas, J. A.; Munoz, J. T.; Hong, S. J.; Muller, W. J.; Doerfler, M.; Williams, C. N.; Drury, K.; Bhagat, D.; Nelson, A.; Price, D.; Dapul, H.; Santos, L.; Kahoud, R.; Francoeur, C.; Appavu, B.; Guilliams, K. P.; Agner, S. C.; Walson, K. H.; Rasmussen, L.; Janas, A.; Ferrazzano, P.; Farias-Moeller, R.; Snooks, K. C.; Chang, C. H.; Yun, J.; Schober, M. E.; Global Consortium Study of Neurologic Dysfunction in, Covid-Investigators	2022	wrong diagnostic test
The Comparison of Children Who Were Diagnosed with COVID-19 in the First and the Second Waves of the SARS-CoV-2 Pandemic	Turan, C.; Basa, E. G.; Elitez, D.; Yilmaz, O.; Gumus, E.; Anil, M.	2021	wrong diagnostic test
Distinguishing Features of Patients Evaluated for Multisystem Inflammatory Syndrome in Children	Kelly, M. S.; Fernandes, N. D.; Carr, A. V.; Lahoud-Rahme, M.; Cummings, B. M.; Chiu, J. S.	2021	wrong diagnostic test
Post-acute COVID-19 outcomes in children with mild and asymptomatic disease	Say, D.; Crawford, N.; McNab, S.; Wurzel, D.; Steer, A.; Tosif, S.	2021	wrong diagnostic test
Trends and Risk Factors of In-Hospital Mortality of Patients with COVID-19 in Germany: Results of a Large Nationwide Inpatient Sample	Hobohm, L.; Sagoschen, I.; Barco, S.; Schmidtmann, I.; Espinola-Klein, C.; Konstantinides, S.; Munzel, T.; Keller, K.	2022	wrong diagnostic test

**Study Title**	**Author names**	**Year**	**Reason for exclusion**
Underlying Medical Conditions Associated With Severe COVID-19 Illness Among Children	Kompaniyets, L.; Agathis, N. T.; Nelson, J. M.; Preston, L. E.; Ko, J. Y.; Belay, B.; Pennington, A. F.; Danielson, M. L.; DeSisto, C. L.; Chevinsky, J. R.; Schieber, L. Z.; Yusuf, H.; Baggs, J.; Mac Kenzie, W. R.; Wong, K. K.; Boehmer, T. K.; Gundlapalli, A. V.; Goodman, A. B.	2021	wrong diagnostic test
Pediatric intensive care unit admissions for COVID-19: Insights using state-level data	Loomba, R. S.; Villarreal, E. G.; Farias, J. S.; Bronicki, R. A.; Flores, S.	2020	wrong diagnostic test
Influence of sex on disease severity in children with multisystem inflammatory syndrome and covid-19 in latin america	Brizuela, M.; Lenzi, J.; Ulloa-GutiÃ©rrez, R.; AntÃºnez-Montes, O. Y.; Aida, J. A. R.; del Aguila, O.; Arteaga-Menchaca, E.; Campos, F.; Uribe, F.; Buitrago, A. P.; LondoÃ±o, L. M. B.; GÃ³mez-Vargas, J.; Yock-Corrales, A.; Buonsenso, D.	2021	wrong diagnostic test
Clinical spectrum of COVID-19 and risk factors associated with severity in Spanish children	Tagarro, A.; Cobos-Carrascosa, E.; Villaverde, S.; Sanz-Santaeufemia, F. J.; Grasa, C.; Soriano-Arandes, A.; Hernanz, A.; Navarro, M. L.; Pino, R.; Epalza, C.; Batista, R.; Rizo, J.; Iglesias-Bouzas, M. I.; Rodriguez-Molino, P.; Villanueva-Medina, S.; Carrasco-Colom, J.; Alonso-Cadenas, J. A.; Mellado, M. J.; Herrero, B.; Melendo, S.; De La Torre, M.; Calleja, L.; Calvo, C.; Urretavizcaya-Martinez, M.; Astigarraga, I.; Menasalvas, A.; Penin, M.; Neth, O.; Berzosa, A.; De Ceano-Vivas, M.; Vidal, P.; Romero, I.; Gonzalez, R.; Garcia, M. L.; Mesa, J. M.; Ballesteros, A.; Bernardino, M.; Moraleda, C.; Epico-Aep Working, Group	2021	wrong diagnostic test
Characteristics of children admitted to hospital with acute SARS-CoV-2 infection in Canada in 2020	Drouin, O.; Hepburn, C. M.; Farrar, D. S.; Baerg, K.; Chan, K.; Cyr, C.; Donner, E. J.; Embree, J. E.; Farrell, C.; Forgie, S.; Giroux, R.; Kang, K. T.; King, M.; Laffin, M.; Luu, T. M.; Orkin, J.; Papenburg, J.; Pound, C. M.; Price, V. E.; Purewal, R.; Sadarangani, M.; Salvadori, M. I.; Top, K. A.; Viel-Theriault, I.; Kakkar, F.; Morris, S. K.; Canadian Paediatric Surveillance Program, Covid-Study Team	2021	wrong diagnostic test
Epidemiology, Clinical Features and Prognostic Factors of Pediatric SARS-CoV-2 Infection: Results From an Italian Multicenter Study	Garazzino, S.; Lo Vecchio, A.; Pierantoni, L.; Calo Carducci, F. I.; Marchetti, F.; Meini, A.; Castagnola, E.; Vergine, G.; Dona, D.; Bosis, S.; Dodi, I.; Venturini, E.; Felici, E.; Giacchero, R.; Denina, M.; Pierri, L.; Nicolini, G.; Montagnani, C.; Krzysztofiak, A.; Bianchini, S.; Marabotto, C.; Tovo, P. A.; Pruccoli, G.; Lanari, M.; Villani, A.;	2021	wrong diagnostic test

**Study Title**	**Author names**	**Year**	**Reason for exclusion**
	Castelli Gattinara, G.; Italian, Sitip- S. I. P. Pediatric Infection Study Group		
Features of COVID-19 Among Children and Adolescents Without Risk Factors Before and After the Delta Variant Outbreak in South Korea	Ryu, Byung-Han; Hong, Sun In; Lim, Su Jin; Cho, Younghwa; Hong, Kyung-Wook; Bae, In-Gyu; Cho, Oh-Hyun	2022	wrong diagnostic test
Benign Evolution of SARS-Cov2 Infections in Children With Inflammatory Bowel Disease: Results From Two International Databases	Brenner, Erica J.; Pigneur, BÃ©nÃ©dicte; Focht, Gili; Zhang, Xian; Ungaro, Ryan C.; Colombel, Jean-Frederic; Turner, Dan; Kappelman, Michael D.; Ruemmele, Frank M.	2021	wrong diagnostic test
SARS-CoV-2 infection in children requiring hospitalization: the experience of Navarra, Spain	Moreno-Galarraga, L.; Urretavizcaya-Martinez, M.; Alegria Echauri, J.; Garcia Howard, M.; Ruperez Garcia, E.; Aguilera-Albesa, S.; Alzina de Aguilar, V.; Herranz Aguirre, M.	2020	wrong diagnostic test
Factors Associated With Severe Gastrointestinal Diagnoses in Children With SARS-CoV-2 Infection or Multisystem Inflammatory Syndrome	Lo Vecchio, A.; Garazzino, S.; Smarrazzo, A.; Venturini, E.; Poeta, M.; Berlese, P.; Denina, M.; Meini, A.; Bosis, S.; Galli, L.; Cazzato, S.; Nicolini, G.; Vergine, G.; Giacchero, R.; Ballardini, G.; Dodi, I.; Salvini, F. M.; Manzoni, P.; Ferrante, G.; Quadri, V.; Campana, A.; Badolato, R.; Villani, A.; Guarino, A.; Gattinara, G. C.; Italian, Sitip- S. I. P. Paediatric Sars-CoV-Infection Study Group	2021	wrong diagnostic test
Incidence of Multisystem Inflammatory Syndrome in Children Among US Persons Infected With SARS-CoV-2	Payne, A. B.; Gilani, Z.; Godfred-Cato, S.; Belay, E. D.; Feldstein, L. R.; Patel, M. M.; Randolph, A. G.; Newhams, M.; Thomas, D.; Magleby, R.; Hsu, K.; Burns, M.; Dufort, E.; Maxted, A.; Pietrowski, M.; Longenberger, A.; Bidol, S.; Henderson, J.; Sosa, L.; Edmundson, A.; Tobin-D'Angelo, M.; Edison, L.; Heidemann, S.; Singh, A. R.; Giuliano, J. S., Jr.; Kleinman, L. C.; Tarquinio, K. M.; Walsh, R. F.; Fitzgerald, J. C.; Clouser, K. N.; Gertz, S. J.; Carroll, R. W.; Carroll, C. L.; Hoots, B. E.; Reed, C.; Dahlgren, F. S.; Oster, M. E.; Pierce, T. J.; Curns, A. T.; Langley, G. E.; Campbell, A. P.; Mis, C. Incidence Authorship Group; Balachandran, N.; Murray, T. S.; Burkholder, C.; Brancard, T.; Lifshitz, J.; Leach, D.; Charpie, I.; Tice, C.; Coffin, S. E.; Perella, D.; Jones, K.; Marohn, K. L.; Yager, P. H.; Fernandes, N. D.; Flori, H. R.; Koncicki, M. L.; Walker, K. S.; Di Pentima, M. C.;	2021	wrong diagnostic test

Study Title	Author names	Year	Reason for exclusion
	Li, S.; Horwitz, S. M.; Gaur, S.; Coffey, D. C.; Harwayne-Gidansky, I.; Hymes, S. R.; Thomas, N. J.; Ackerman, K. G.; Cholette, J. M.		
Characteristics, Outcomes, and Severity Risk Factors Associated With SARS-CoV-2 Infection Among Children in the US National COVID Cohort Collaborative	Martin, B.; DeWitt, P. E.; Russell, S.; Anand, A.; Bradwell, K. R.; Bremer, C.; Gabriel, D.; Girvin, A. T.; Hajagos, J. G.; McMurry, J. A.; Neumann, A. J.; Pfaff, E. R.; Walden, A.; Wooldridge, J. T.; Yoo, Y. J.; Saltz, J.; Gersing, K. R.; Chute, C. G.; Haendel, M. A.; Moffitt, R.; Bennett, T. D.	2022	wrong diagnostic test
The impact of COVID-19 lockdown on children with medical complexity in pediatric emergency department	Brisca, G.; Vagelli, G.; Tagliarini, G.; Rotulo, A.; Pirlo, D.; Romanengo, M.; Piccotti, E.	2021	wrong diagnostic test
The low incidence of clinically significant heart disease in school-age children following COVID-19	Powell, A. W.; Statile, C. J.; Madsen, N. L.; Divanovic, A. A.; Lang, S. M.	2022	wrong diagnostic test
Impact of COVID-19 on the Children with Cancer in 6 Pediatric Oncology Units (POU's) of Pakistan-a Multi-Center Study	Raza, M. R.; Maqsood, S.; Rana, Z. A.; Hamid, H.; Yasmeen, N.; Rehman, M. F. U.; Iqbal, R.; Ashraf, M. S.	2021	wrong diagnostic test
Evaluation of covid-19 disease and the effect of trends in intervention measures: The pediatric perspective from a tertiary care hospital in turkey	Sik, N.; Duman, M.; Yilmaz, D.; Asrak, H. K.; Erbas, I. C.; Guzin, A. C.; Alatas, S. O.; Appak, O.; Sayiner, A. A.; Belet, N.	2021	wrong diagnostic test
Neurologic Involvement in Children and Adolescents Hospitalized in the United States for COVID-19 or Multisystem Inflammatory Syndrome	LaRovere, K. L.; Riggs, B. J.; Poussaint, T. Y.; Young, C. C.; Newhams, M. M.; Maamari, M.; Walker, T. C.; Singh, A. R.; Dapul, H.; Hobbs, C. V.; McLaughlin, G. E.; Son, M. B. F.; Maddux, A. B.; Clouser, K. N.; Rowan, C. M.; McGuire, J. K.; Fitzgerald, J. C.; Gertz, S. J.; Shein, S. L.; Munoz, A. C.; Thomas, N. J.; Irby, K.; Levy, E. R.; Staat, M. A.; Tenforde, M. W.; Feldstein, L. R.; Halasa, N. B.; Giuliano, J. S., Jr.; Hall, M. W.; Kong, M.; Carroll, C. L.; Schuster, J. E.; Doymaz, S.; Loftis, L. L.; Tarquinio, K. M.; Babbitt, C. J.; Nofziger, R. A.; Kleinman, L. C.; Keenaghan, M. A.; Cvijanovich, N. Z.; Spinella, P. C.; Hume, J. R.; Wellnitz, K.; Mack, E. H.; Michelson, K. N.; Flori, H. R.; Patel, M. M.; Randolph, A. G.; Overcoming, Covid-Investigators	2021	wrong diagnostic test

Study Title	Author names	Year	Reason for exclusion
Association of Age With Likelihood of Developing Symptoms and Critical Disease Among Close Contacts Exposed to Patients With Confirmed SARS-CoV-2 Infection in Italy	Poletti, P.; Tirani, M.; Cereda, D.; Trentini, F.; Guzzetta, G.; Sabatino, G.; Marziano, V.; Castrofino, A.; Grosso, F.; Del Castillo, G.; Piccarreta, R.; Andreassi, A.; Melegaro, A.; Gramegna, M.; Ajelli, M.; Merler, S.; A. T. S. Lombardy COVID-19 Task Force	2021	wrong diagnostic test
Antibiotic Prescribing in Children Hospitalized With COVID-19 and Multisystem Inflammatory Syndrome in Spain: Prevalence, Trends, and Associated Factors	Aguilera-Alonso, D.; Epalza, C.; Sanz-Santaeufemia, F. J.; Grasa, C.; Villanueva-Medina, S.; Melendo Perez, S.; Cervantes Hernandez, E.; Urretavizcaya-Martinez, M.; Pino, R.; Gomez, M. N.; Orive, J. P.; Gonzalez Zarate, A.; Vidal Lana, P.; Gonzalez Montero, R.; Ruiz Gonzalez, S.; Calvo, C.; Iglesias-Bouzas, M. I.; Caro-Teller, J. M.; Dominguez-Rodriguez, S.; Ballesteros, A.; Mesa, J.; Cobos-Carrascosa, E.; Tagarro, A.; Moraleda, C.	2022	wrong diagnostic test
Characterisation of 22445 patients attending UK emergency departments with suspected COVID-19 infection: Observational cohort study	Goodacre, S.; Thomas, B.; Lee, E.; Sutton, L.; Loban, A.; Waterhouse, S.; Simmonds, R.; Biggs, K.; Marincowitz, C.; Schutter, J.; Connelly, S.; Sheldon, E.; Hall, J.; Young, E.; Bentley, A.; Challen, K.; Fitzsimmons, C.; Harris, T.; Lecky, F.; Lee, A.; Maconochie, I.; Walter, D.	2020	wrong diagnostic test
Differences in children and adolescents with SARS-CoV-2 infection: a cohort study in a Brazilian tertiary referral hospital	Marques, H. H. S.; Pereira, M. F. B.; Santos, A. C. D.; Fink, T. T.; Paula, C. S. Y.; Litvinov, N.; Schvartsman, C.; Delgado, A. F.; Gibelli, Mabc; Carvalho, W. B.; Odone Filho, V.; Tannuri, U.; Carneiro-Sampaio, M.; Grisi, S.; Duarte, Ajds; Antonangelo, L.; Francisco, R. P. V.; Okay, T. S.; Batisttella, L. R.; Carvalho, C. R. R.; Brentani, A. V. M.; Silva, C. A.; Hc-Fmusp Pediatric, Covid Study Group; Eisencraft, A. P.; Rossi Junior, A.; Fante, A. L.; Cora, A. P.; Reis, Agac; Ferrer, A. P. S.; Andrade, A. P. M.; Watanabe, A.; Goncalves, A. M. F.; Waetge, A. R. P.; Silva, C. A.; Ceneviva, C.; Lazari, C. D. S.; Abellan, D. M.; Santos, E. H. D.; Sabino, E. C.; Bianchini, F. R. M.; Alcantara, F. F. P.; Ramos, G. F.; Leal, G. N.; Rodriguez, I. S.; Pinho, J. R. R.; Carneiro, J. D. A.; Paz, J. A.; Ferreira, J. C.; Ferranti, J. F.; Ferreira, J. O. A.; Framil, J. V. S.; Silva, K. R. D.; Kanunfre, K. A.; Bastos, K. L. M.; Galleti, K. V.; Cristofani, L. M.; Suzuki, L.; Campos, L. M. A.; Perondi, M. B. M.; Diniz, M. F. R.; Fonseca, M. F. M.; Cordon, M. N. A.; Pissolato, M.; Peres, M. S.;	2021	wrong diagnostic test

Study Title	Author names	Year	Reason for exclusion
	Garanito, M. P.; Imamura, M.; Dorna, M. B.; Luglio, M.; Rocha, M. C.; Aikawa, N. E.; Degaspare, N. V.; Sakita, N. K.; Udsen, N. L.; Scudeller, P. G.; Gaiolla, P. V. V.; Severini, Rdsg; Rodrigues, R. M.; Toma, R. K.; Paula, R. I. C.; Palmeira, P.; Forsait, S.; Farhat, S. C. L.; Sakano, T. M. S.; Koch, V. H. K.; Cobello Junior, V.		
Clinical characteristics of children and young people admitted to hospital with covid-19 in United Kingdom: prospective multicentre observational cohort study	Swann, O. V.; Holden, K. A.; Turtle, L.; Pollock, L.; Fairfield, C. J.; Drake, T. M.; Seth, S.; Egan, C.; Hardwick, H. E.; Halpin, S.; Girvan, M.; Donohue, C.; Pritchard, M.; Patel, L. B.; Ladhani, S.; Sigfrid, L.; Sinha, I. P.; Olliaro, P. L.; Nguyen-Van-Tam, J. S.; Horby, P. W.; Merson, L.; Carson, G.; Dunning, J.; Openshaw, P. J. M.; Baillie, J. K.; Harrison, E. M.; Docherty, A. B.; Semple, M. G.; Isaric, C. Investigators	2020	wrong diagnostic test
Multisystem Inflammatory Syndrome in U.S. Children and Adolescents	Feldstein, L. R.; Rose, E. B.; Horwitz, S. M.; Collins, J. P.; Newhams, M. M.; Son, M. B. F.; Newburger, J. W.; Kleinman, L. C.; Heidemann, S. M.; Martin, A. A.; Singh, A. R.; Li, S.; Tarquinio, K. M.; Jaggi, P.; Oster, M. E.; Zackai, S. P.; Gillen, J.; Ratner, A. J.; Walsh, R. F.; Fitzgerald, J. C.; Keenaghan, M. A.; Alharash, H.; Doymaz, S.; Clouser, K. N.; Giuliano, J. S., Jr.; Gupta, A.; Parker, R. M.; Maddux, A. B.; Havalad, V.; Ramsingh, S.; Bukulmez, H.; Bradford, T. T.; Smith, L. S.; Tenforde, M. W.; Carroll, C. L.; Riggs, B. J.; Gertz, S. J.; Daube, A.; Lansell, A.; Coronado Munoz, A.; Hobbs, C. V.; Marohn, K. L.; Halasa, N. B.; Patel, M. M.; Randolph, A. G.; Overcoming, Covid-Investigators; Cdc Covid- Response, Team	2020	wrong diagnostic test
Risk for Newly Diagnosed Diabetes >30 Days After SARS-CoV-2 Infection Among Persons Aged <18 Years - United States, March 1, 2020-June 28, 2021	Barrett, C. E.; Koyama, A. K.; Alvarez, P.; Chow, W.; Lundeen, E. A.; Perrine, C. G.; Pavkov, M. E.; Rolka, D. B.; Wiltz, J. L.; Bull-Otterson, L.; Gray, S.; Boehmer, T. K.; Gundlapalli, A. V.; Siegel, D. A.; Kompaniyets, L.; Goodman, A. B.; Mahon, B. E.; Tauxe, R. V.; Remley, K.; Saydah, S.	2022	wrong diagnostic test
Management of Children Admitted to Hospitals across Bangladesh with Suspected or Confirmed COVID-19 and the Implications for the Future: A Nationwide Cross-Sectional Study	Chowdhury, K.; Haque, M.; Nusrat, N.; Adnan, N.; Islam, S.; Lutfor, A. B.; Begum, D.; Rabbany, A.; Karim, E.; Malek, A.; Jahan, N.; Akter, J.; Ashraf, S.; Hasan, M. N.; Hassan, M.; Akhter, N.; Mazumder, M.; Sihan, N.; Naher, N.; Akter, S.; Zaman, S. U.; Chowdhury, T.; Nesa, J.; Biswas, S.; Islam, M. D.; Hossain, A. M.;	2022	wrong diagnostic test

Study Title	Author names	Year	Reason for exclusion
	Rahman, H.; Biswas, P. K.; Shaheen, M.; Chowdhury, F.; Kumar, S.; Kurdi, A.; Mustafa, Z. U.; Schellack, N.; Gowere, M.; Meyer, J. C.; Opanga, S.; Godman, B.		
Multisystem Inflammatory Syndrome in Infants <12 months of Age, United States, May 2020-January 2021	Godfred-Cato, S.; Tsang, C. A.; Giovanni, J.; Abrams, J.; Oster, M. E.; Lee, E. H.; Lash, M. K.; Le Marchand, C.; Liu, C. Y.; Newhouse, C. N.; Richardson, G.; Murray, M. T.; Lim, S.; Haupt, T. E.; Hartley, A.; Sosa, L. E.; Ngamsnga, K.; Garcia, A.; Datta, D.; Belay, E. D.	2021	wrong diagnostic test
Early report from the Pediatric Heart Transplant Society on COVID-19 infections in pediatric heart transplant candidates and recipients	Conway, J.; Auerbach, S. R.; Richmond, M. E.; Sharp, B.; Pahl, E.; Feingold, B.; Azeka, E.; Dryer, W. J.; Cantor, R. S.; Kirklin, J. K.	2022	wrong diagnostic test
Demographic and Clinical Factors Associated With Death Among Persons <21 Years Old With Multisystem Inflammatory Syndrome in Children-United States, February 2020-March 2021	Bowen, A.; Miller, A. D.; Zambrano, L. D.; Wu, M. J.; Oster, M. E.; Godfred-Cato, S.; Belay, E. D.; Campbell, A. P.	2021	wrong diagnostic test
COVID-19-Associated Multisystem Inflammatory Syndrome in Children - United States, March-July 2020	Godfred-Cato, S.; Bryant, B.; Leung, J.; Oster, M. E.; Conklin, L.; Abrams, J.; Roguski, K.; Wallace, B.; Prezzato, E.; Koumans, E. H.; Lee, E. H.; Geevarughese, A.; Lash, M. K.; Reilly, K. H.; Pulver, W. P.; Thomas, D.; Feder, K. A.; Hsu, K. K.; Plipat, N.; Richardson, G.; Reid, H.; Lim, S.; Schmitz, A.; Pierce, T.; Hrapcak, S.; Datta, D.; Morris, S. B.; Clarke, K.; Belay, E.; California, M. I. S. C. Response Team	2020	wrong diagnostic test
Myocardial involvement in children with post-COVID multisystem inflammatory syndrome: a cardiovascular magnetic resonance based multicenter international study-the CARDOVID registry	Aeschlimann, F. A.; Misra, N.; Hussein, T.; Panaioli, E.; Soslow, J. H.; Crum, K.; Steele, J. M.; Huber, S.; Marcora, S.; Brambilla, P.; Jain, S.; Navallas, M.; Giuli, V.; Rucker, B.; Angst, F.; Patel, M. D.; Azarine, A.; Caro-Dominguez, P.; Cavaliere, A.; Di Salvo, G.; Ferroni, F.; Agnoletti, G.; Bonnemains, L.; Martins, D.; Boddaert, N.; Wong, J.; Pushparajah, K.; Raimondi, F.	2021	wrong diagnostic test
Multisystem Inflammatory Syndrome in Children: An International Survey	Bautista-Rodriguez, C.; Sanchez-de-Toledo, J.; Clark, B. C.; Herberg, J.; Bajolle, F.; Randanne, P. C.; Salas-Mera, D.; Foldvari, S.; Chowdhury, D.; Munoz, R.; Bianco, F.; Singh, Y.; Levin, M.; Bonnet, D.; Fraisse, A.	2021	wrong diagnostic test

**Study Title**	**Author names**	**Year**	**Reason for exclusion**
Differentiating multisystem inflammatory syndrome in children: a single-centre retrospective cohort study	Roberts, J. E.; Campbell, J. I.; Gauvreau, K.; Lamb, G. S.; Newburger, J.; Son, M. B.; Dionne, A.	2022	wrong diagnostic test
Mental Health in Children in the Context of COVID-19: Focus on Discharged Children	Zhang, A.; Shi, L.; Yan, W.; Xiao, H.; Bao, Y.; Wang, Z.; Deng, J.; Ravindran, A.; Yuan, K.; Mei, H.; Shi, J.; Liu, Z.; Liu, J.; Lu, L.	2021	wrong diagnostic test
Screening for SARS-CoV-2 infection in pediatric oncology patients during the epidemic peak in Italy	Cesaro, S.; Compagno, F.; Zama, D.; Meneghello, L.; Giurici, N.; Soncini, E.; Onofrillo, D.; Mercolini, F.; Mura, R.; Perruccio, K.; De Santis, R.; Colombini, A.; Barone, A.; Sainati, L.; Baretta, V.; Petris, M. G.	2020	wrong diagnostic test
Flash survey on severe acute respiratory syndrome coronavirus-2 infections in paediatric patients on anticancer treatment	Hrusak, O.; Kalina, T.; Wolf, J.; Balduzzi, A.; Provenzi, M.; Rizzari, C.; Rives, S.; Del Pozo Carlavilla, M.; Alonso, M. E. V.; Dominguez-Pinilla, N.; Bourquin, J. P.; Schmiegelow, K.; Attarbaschi, A.; Grillner, P.; Mellgren, K.; van der Werff Ten Bosch, J.; Pieters, R.; Brozou, T.; Borkhardt, A.; Escherich, G.; Lauten, M.; Stanulla, M.; Smith, O.; Yeoh, A. E. J.; Elitzur, S.; Vora, A.; Li, C. K.; Ariffin, H.; Kolenova, A.; Dallapozza, L.; Farah, R.; Lazic, J.; Manabe, A.; Styczynski, J.; Kovacs, G.; Ottoffy, G.; Felice, M. S.; Buldini, B.; Conter, V.; Stary, J.; Schrappe, M.	2020	wrong diagnostic test
Distinct characteristics of multisystem inflammatory syndrome in children in Poland	Ludwikowska, Kamila Maria; Okarska-Napierala, Magdalena; Dudek, Natalia; Tracewski, Pawel; Kusa, Jacek; Piwonski, Krzysztof Piotr; Afelt, Aneta; Cysewski, Dominik; Biela, Mateusz; Werner, Bozena; Jackowska, Teresa; Suski-Grabowski, Catherine; Kursa, Miron Bartosz; Kuchar, Ernest; Szenborn, Leszek	2021	wrong diagnostic test
Multisystem inflammatory syndrome in children in Canada	Laverty, M.; Salvadori, M.; Squires, S. G.; Ahmed, M.; Eisenbeis, L.; Lee, S.; Cormiers, A. des; Li, Y. A.	2021	wrong diagnostic test
COVID-19-associated multisystem inflammatory syndrome in children: a multicentric retrospective cohort study	Bharat, Mehra; Mukul, Pandey; Dhiren, Gupta; Tania, Oberoi; Nameet, Jerath; Rachna, Sharma; Naresh, Lal; Chandrasekhar, Singha; Bhavana, Malhotra; Vinamra, Manocha; Simalti, A. K.; Yogesh, Arya; Dugaya, S. K.; Swati, Kalra; Chitkara, A. J.; Anil, Sachdev; Neeraj, Gupta	2021	wrong diagnostic test

**Study Title**	**Author names**	**Year**	**Reason for exclusion**
Factors linked to severe outcomes in multisystem inflammatory syndrome in children (MIS-C) in the USA: a retrospective surveillance study	Abrams, J. Y.; Oster, M. E.; Godfred-Cato, S. E.; Bryant, B.; Datta, S. D.; Campbell, A. P.; Leung, J. W.; Tsang, C. A.; Pierce, T. J.; Kennedy, J. L.; Hammett, T. A.; Belay, E. D.	2021	wrong diagnostic test
Neurological manifestations of SARS-CoV-2 infection in hospitalised children and adolescents in the UK: a prospective national cohort study	Ray, S. T. J.; Abdel-Mannan, O.; Sa, M.; Fuller, C.; Wood, G. K.; Pysden, K.; Yoong, M.; McCullagh, H.; Scott, D.; McMahon, M.; Thomas, N.; Taylor, M.; Illingworth, M.; McCrea, N.; Davies, V.; Whitehouse, W.; Zuberi, S.; Guthrie, K.; Wassmer, E.; Shah, N.; Baker, M. R.; Tiwary, S.; Tan, H. J.; Varma, U.; Ram, D.; Avula, S.; Enright, N.; Hassell, J.; Ross Russell, A. L.; Kumar, R.; Mulholland, R. E.; Pett, S.; Galea, I.; Thomas, R. H.; Lim, M.; Hacohen, Y.; Solomon, T.; Griffiths, M. J.; Michael, B. D.; Kneen, R.; CoroNerve study, group	2021	wrong diagnostic test
COVID-19 in Turkey: a tertiary center experience	Onal, P.; Kilinc, A. A.; Aygun, F.; Durak, C.; Cokugras, H.	2020	wrong diagnostic test
Pseudo-likelihood based logistic regression for estimating COVID-19 infection and case fatality rates by gender, race, and age in California. (Special Issue: Epidemics now.)	Xiong, Di; Zhang, Lu; Watson, G. L.; Sundin, P.; Bufford, T.; Zoller, J. A.; Shamshoian, J.; Suchard, M. A.; Ramirez, C. M.	2020	wrong diagnostic test
Trends in Geographic and Temporal Distribution of US Children With Multisystem Inflammatory Syndrome During the COVID-19 Pandemic	Belay, E. D.; Abrams, J.; Oster, M. E.; Giovanni, J.; Pierce, T.; Meng, L.; Prezzato, E.; Balachandran, N.; Openshaw, J. J.; Rosen, H. E.; Kim, M.; Richardson, G.; Hand, J.; Tobin-D'Angelo, M.; Wilson, S.; Hartley, A.; Jones, C.; Kolsin, J.; Mohamed, H.; Colles, Z.; Hammett, T.; Patel, P.; Stierman, B.; Campbell, A. P.; Godfred-Cato, S.	2021	wrong diagnostic test
Population-based study of multisystem inflammatory syndrome associated with COVID-19 found that 36% of children had persistent symptoms	Kahn, R.; Berg, S.; Berntson, L.; Berthold, E.; Brodin, P.; B..ckstr..m, F.; Compagno, M.; Fasth, A.; Framme, J. L.; Horne, A.; H..tting, J.; Kr..l, P.; Kukka, A. J.; Mossberg, M.; M..nsson, B.; Nordenh..ll, C.; Nordstr..m, S. I.; Nystr..m, F. K.; Palmblad, K.; Rasti, R.; Rudolph, A.; Rydenman, K.; Sundberg, E.; S..ve-S..derbergh, E.; Altman, M.	2021	wrong diagnostic test
Trends in Pediatric Hospitalizations for Coronavirus Disease 2019	Levin, Z.; Choyke, K.; Georgiou, A.; Sen, S.; Karaca-Mandic, P.	2021	wrong diagnostic test

**Study Title**	**Author names**	**Year**	**Reason for exclusion**
SARS-CoV-2 in Childhood Cancer in 2020: A Disease of Disparities	Johnston, E. E.; Martinez, I.; Davis, E. S.; Caudill, C.; Richman, J.; Brackett, J.; Dickens, D. S.; Kahn, A.; Schwalm, C.; Sharma, A.; Patel, P. A.; Bhatia, S.; Levine, J. M.; Wolfson, J. A.	2021	wrong diagnostic test
Retropharyngeal Edema and Neck Pain in Multisystem Inflammatory Syndrome in Children (MIS-c)	Jenkins, Elan; Sherry, Whitney; Smith, Alison G. C.; Rostad, Bradley S.; Rostad, Christina A.; Jones, Kaitlin; Jaggi, Preeti	2021	wrong diagnostic test
Treatment of Multisystem Inflammatory Syndrome in Children	McArdle, Andrew J.; Vito, Ortensia; Patel, Harsita; Seaby, Eleanor G.; Shah, Priyen; Wilson, Clare; Broderick, Claire; Nijman, Ruud; Tremoulet, Adriana H.; Munblit, Daniel; Ulloa-Gutierrez, Rolando; Carter, Michael J.; De, Tisham; Hoggart, Clive; Whittaker, Elizabeth; Herberg, Jethro A.; Kaforou, Myrsini; Cunnington, Aubrey J.; Levin, Michael	2021	wrong diagnostic test
Pediatric Ischemic Stroke: An Infrequent Complication of SARS-CoV-2	Beslow, L. A.; Linds, A. B.; Fox, C. K.; Kossorotoff, M.; Zuniga Zambrano, Y. C.; Hernandez-Chavez, M.; Hassanein, S. M. A.; Byrne, S.; Lim, M.; Maduaka, N.; Zafeiriou, D.; Dowling, M. M.; Felling, R. J.; Rafay, M. F.; Lehman, L. L.; Noetzel, M. J.; Bernard, T. J.; Dlamini, N.	2021	wrong diagnostic test
Multisystem inflammatory syndrome in children in New York state	Dufort, E. M.; Koumans, E. H.; Chow, E. J.; Rosenthal, E. M.; Muse, A.; Rowlands, J.; Barranco, M. A.; Maxted, A. M.; Rosenberg, E. S.; Easton, D.; Udo, T.; Kumar, J.; Pulver, W.; Smith, L.; Hutton, B.; Blog, D.; Zucker, H.	2020	wrong diagnostic test
Neither inflammatory bowel disease nor immunosuppressants are associated with an increased risk of severe COVID-19: an observational Dutch cohort study	Gilissen, Lennard P. L.; Heinen, Stefan G. H.; Rijpma-Jacobs, Lotte; Schoon, Erik; Schreuder, Ramon-Michel; Wensing, Anne-Marie; van der Ende-van Loon, Mirjam C. M.; Bloemen, Johanne G.; Stapelbroek, Janneke M.; Stronkhorst, Arnold	2021	wrong diagnostic test
Long-term effects of malnutrition on severity of COVID-19	Kurtz, A.; Grant, K.; Marano, R.; Arrieta, A.; Grant, K., Jr.; Feaster, W.; Steele, C.; Ehwerhemuepha, L.	2021	wrong diagnostic test
The impact of covid-19 on cognitive development and executive functioning in adolescents: A first exploratory investigation	Frolli, A.; Ricci, M. C.; Carmine, F. D.; Lombardi, A.; Bosco, A.; Saviano, E.; Franzese, L.	2021	wrong diagnostic test

**Study Title**	**Author names**	**Year**	**Reason for exclusion**
Experience of COVID positive pediatric surgical patients from a tertiary care center in a Himalayan state	Bashir, I.; Hamid, R.; Sudhanshu, A.; Wani, T. A.; Bhat, N. A.; Baba, A. A.; Mufti, G. N.; Jan, W.	2021	wrong diagnostic test
Prevalence of SARS-CoV-2 Infection in Children and Their Parents in Southwest Germany	Tonshoff, B.; Muller, B.; Elling, R.; Renk, H.; Meissner, P.; Hengel, H.; Garbade, S. F.; Kieser, M.; Jeltsch, K.; Grulich-Henn, J.; Euler, J.; Stich, M.; Chobanyan-Jurgens, K.; Zernickel, M.; Janda, A.; Wolfle, L.; Stamminger, T.; Iftner, T.; Ganzenmueller, T.; Schmitt, C.; Gorne, T.; Laketa, V.; Olberg, S.; Plaszczyca, A.; Cortese, M.; Bartenschlager, R.; Pape, C.; Remme, R.; Huzly, D.; Panning, M.; Weigang, S.; Giese, S.; Ciminski, K.; Ankerhold, J.; Kochs, G.; Schwemmle, M.; Handgretinger, R.; Niemeyer, C. M.; Engel, C.; Kern, W. V.; Hoffmann, G. F.; Franz, A. R.; Henneke, P.; Debatin, K. M.; Krausslich, H. G.	2021	wrong diagnostic test
COVID-19 in pediatric palliative care: what can we learn from the pandemic and possible future directions	Avagnina, Irene; Zanin, Anna; Lazzarin, Pierina; Grigolon, Enrica; Shahi, Aashni; Papa, Simonetta; Giacomelli, Luca; Benini, Franca	2021	wrong diagnostic test
Demographic, clinical, and laboratory characteristics of children with confirmed Coronavirus disease 2019 (COVID-19) in Bali	Purniti, N. P. S.; Sidiartha, I. G. L.; Subanada, I. B.; Mayangsari, A. S. M.; Aryani, G. A. D.; Adi, I. P. D.	2021	wrong diagnostic test
Management of Juvenile idiopathic arthritis-associated uveitis duringthe COVID-19 pandemic in a pediatric referral center in Lombardy	Miserocchi, E.; Giuffre, C.; Modorati, G. M.; Cimaz, R.	2020	wrong diagnostic test
Severe Acute Respiratory Syndrome due to COVID-19 among children and adolescents in Brazil: profile of deaths and hospital lethality as at Epidemiological Week 38, 2020	Hillesheim, D.; Tomasi, Y. T.; Figueiro, T. H.; de Paiva, K. M.	2020	wrong diagnostic test
Factors Associated With COVID-19 Disease Severity in US Children and Adolescents	Antoon, J. W.; Grijalva, C. G.; Thurm, C.; Richardson, T.; Spaulding, A. B.; Teufel, R. J., 2nd; Reyes, M. A.; Shah, S. S.; Burns, J. E.; Kenyon, C. C.; Hersh, A. L.; Williams, D. J.	2021	wrong diagnostic test

Study Title	Author names	Year	Reason for exclusion
COVID-19 in children: clinical and epidemiological spectrum in the community	Garcia-Vera, C.; Castejon-Ramirez, S.; Lain Miranda, E.; Hernandez Abadia, R.; Garcia Ventura, M.; Borque Navarro, E.; Rubio Sanchez, P.; Baeta Ruiz, A.; Mengual Gil, J. M.	2021	wrong diagnostic test
Abdominal US in Pediatric Inflammatory Multisystem Syndrome Associated with COVID-19	Meshaka, R.; Whittam, F. C.; Guessoum, M.; Eleti, S.; Shelmerdine, S. C.; Arthurs, O. J.; McHugh, K.; Hiorns, M. P.; Humphries, P. D.; Calder, A. D.; Easty, M. J.; Gaynor, E. P.; Watson, T.	2021	wrong diagnostic test
Populationâ€• based study of multisystem inflammatory syndrome associated with COVIDâ€• 19 found that 36% of children had persistent symptoms	Kahn, Robin; Berg, Stefan; Berntson, Lillemor; Berthold, Elisabet; Brodin, Petter; BÃ¤ckstrÃ¶m, Fredrik; Compagno, Michele; Fasth, Anders; Lingman Framme, Jenny; Horne, AnnaCarin; HÃ¤tting, Josefin; KrÃ³l, Petra; Kukka, Antti J.; Mossberg, Maria; MÃ¥nsson, Bengt; NordenhÃ¤ll, Charlotta; Idring NordstrÃ¶m, Selma; Khammari NystrÃ¶m, Fatine; Palmblad, Karin; Rasti, Reza	2022	wrong diagnostic test
Spectrum of COVID-19 in children	Ranabothu, S.; Onteddu, S.; Nalleballe, K.; Dandu, V.; Veerapaneni, K.; Veerapandiyan, A.	2020	wrong diagnostic test
Acute pancreatitis in children hospitalized with COVID-19	Suchman, K.; Raphael, K. L.; Liu, Y.; Wee, D.; Trindade, A. J.	2021	wrong diagnostic test
Patterns of myocardial involvement during COVID-19 pandemic; from newborn to adolescents	Tun..er, T.; Varol, F.; Coskun, S.; G..zel, B.; G..ven, S.; am, H.	2021	wrong diagnostic test
Preventing COVID-19 Transmission in Education Settings	Kaiser, S. V.; Watson, A.; Dogan, B.; Karmur, A.; Warren, K.; Wang, P.; Camano Sosa, M.; Olarte, A.; Dorsey, S.; Su, M.; Brown, L.; Sachdev, D.; Bardach, N. S.	2021	wrong diagnostic test
SARS-CoV-2 infection and return to play in junior competitive athletes: is systematic cardiac screening needed?	Cavigli, L.; Cillis, M.; Mochi, V.; Frascaro, F.; Mochi, N.; Hajdarevic, A.; Roselli, A.; Capitani, M.; Alvino, F.; Giovani, S.; Lisi, C.; Cappellini, M. T.; Colloca, R. A.; Mandoli, G. E.; Valente, S.; Focardi, M.; Cameli, M.; Bonifazi, M.; D'Ascenzi, F.	2022	wrong diagnostic test
To study demographics as risk factor for mortality associated with COVID-19: a retrospective cohort study	Bakshi, A. S.; Neetu, Sharma; Jasbir, Singh; Sandeep, Batish; Vijay, Sehgal	2021	wrong diagnostic test
Clinical spectrum and atypical presentations of COVID-19 in hospitalized children in a tertiary care hospital: prospective observational study	Shweta, Pathak; Lazarus, M.; Ghanghoriya, P.	2020	wrong diagnostic test

Study Title	Author names	Year	Reason for exclusion
Clinical, epidemiological, and laboratory characteristics of mild-to-moderate COVID-19 patients in Saudi Arabia: an observational cohort study	Al Mutair, A.; Alhumaid, S.; Alhuqbani, W. N.; Zaidi, A. R. Z.; Alkoraisi, S.; Al-Subaie, M. F.; AlHindi, A. M.; Abogosh, A. K.; Alrasheed, A. K.; Alsharafi, A. A.; Alhuqbani, M. N.; Alhowar, N. A.; Salih, S.; Alhedaithy, M. A.; Al-Tawfiq, J. A.; Al-Shammari, H.; Abdulqawi, R.; Ismail, A. F.; Hamdan, N.; Saad, F.; Olhaye, F. A.; Eltahir, T. A.; Rabaan, A. A.; Al-Omari, A.	2020	wrong diagnostic test
Characterizing the differences between multisystem inflammatory syndrome in children and Kawasaki disease	Bar-Meir, M.; Guri, A.; Godfrey, M. E.; Shack, A. R.; Hashkes, P. J.; Goldzweig, O.; Megged, O.	2021	wrong diagnostic test
The clinical course of SARS-CoV-2 infection among children with rheumatic disease under biologic therapy: a retrospective and multicenter study	Sozeri, B.; Ulu, K.; Kaya-Akca, U.; Haslak, F.; Pac-Kisaarslan, A.; Otar-Yener, G.; Baba, O.; Altug-Gucenmez, O.; Sahin, N.; Baglan, E.; Sonmez, H. E.; Cakmak, F.; Ozturk, K.; Gezgin-Yildirim, D.; Sener, S.; Barut, K.; Batu, E. D.; Yildiz, M.; Basaran, O.; Adrovic, A.; Sahin, S.; Ozdel, S.; Bilginer, Y.; Poyrazoglu, M. H.; Demir, F.; Yuksel, S.; Kalyoncu, M.; Kasapcopur, O.; Ozen, S.; Aktay-Ayaz, N.	2022	wrong diagnostic test
Acute Kidney Injury in Pediatric Acute SARS-CoV-2 Infection and Multisystem Inflammatory Syndrome in Children (MIS-C): Is There a Difference?	Grewal, M. K.; Gregory, M. J.; Jain, A.; Mohammad, D.; Cashen, K.; Ang, J. Y.; Thomas, R. L.; Valentini, R. P.	2021	wrong diagnostic test
Multisystem Inflammatory Syndrome in Children by COVID-19 Vaccination Status of Adolescents in France	Levy, M.; Recher, M.; Hubert, H.; Javouhey, E.; Flechelles, O.; Leteurtre, S.; Angoulvant, F.	2022	wrong diagnostic test
COVID-19 Gastrointestinal Manifestations Are Independent Predictors of PICU Admission in Hospitalized Pediatric Patients	Gonzalez Jimenez, D.; Velasco Rodriguez-Belvis, M.; Ferrer Gonzalez, P.; Dominguez Ortega, G.; Segarra, O.; Medina Benitez, E.; Garcia Tirado, D.; Garcia Romero, R.; Vecino Lopez, R.; Crehua-Gaudiza, E.; Queralt, M.; Palomino Perez, L. M.; Diaz Martin, J. J.	2020	wrong diagnostic test
Impact of COVID-19 pandemic on the management of paediatric inflammatory bowel disease: An Italian multicentre study on behalf of the SIGENP IBD Group	Arrigo, S.; Alvisi, P.; Banzato, C.; Bramuzzo, M.; Celano, R.; Civitelli, F.; D'Arcangelo, G.; Dilillo, A.; Dipasquale, V.; Felici, E.; Fuoti, M.; Gatti, S.; Knafelz, D.; Lionetti, P.; Mario, F.; Marseglia, A.; Martelossi, S.; Moretti, C.; Norsa, L.; Panceri, R.; Renzo, S.; Romano, C.; Romeo, E.; Strisciuglio, C.; Martinelli, M.	2021	wrong diagnostic test
Acute Cardiovascular Manifestations in 286 Children With Multisystem	Valverde, I.; Singh, Y.; Sanchez-de-Toledo, J.; Theocharis, P.; Chikermane, A.; Di Filippo, S.; Kucinska, B.; Mannarino, S.;	2021	wrong diagnostic test

**Study Title**	**Author names**	**Year**	**Reason for exclusion**
Inflammatory Syndrome Associated With COVID-19 Infection in Europe	Tamariz-Martel, A.; Gutierrez-Larraya, F.; Soda, G.; Vandekerckhove, K.; Gonzalez-Barlatay, F.; McMahon, C. J.; Marcora, S.; Napoleone, C. P.; Duong, P.; Tuo, G.; Deri, A.; Nepali, G.; Ilina, M.; Ciliberti, P.; Miller, O.; Aepc Covid- Rapid Response, Team		
Increased frequency of severe diabetic ketoacidosis at type 1 diabetes onset among children during COVID-19 pandemic lockdown: an observational cohort study	Dzygalo, K.; Nowaczyk, J.; Szwilling, A.; Kowalska, A.	2020	wrong diagnostic test
Worse Hospital Outcomes for Children and Adults with COVID-19 and Congenital Heart Disease	Strah, D. D.; Kowalek, K. A.; Weinberger, K.; Mendelson, J.; Hoyer, A. W.; Klewer, S. E.; Seckeler, M. D.	2021	wrong diagnostic test
Association of Ethnicity With Multisystem Inflammatory Syndrome in Children Related to SARS-CoV-2 Infection: An International Case-Referent Study	Middelburg, J. G.; Crijnen, T. E. M.; D'Antiga, L.; Verdoni, L.; Chikermane, A.; Garg, P.; Acharyya, B. C.; Pruccoli, G.; Schnapp, A.; Rauf, A.; Middelburg, R. A.	2021	wrong diagnostic test
Laboratory-confirmed COVID-19 in children and youth in Canada, January 15-April 27, 2020	Paquette, D.; Bell, C.; Roy, M.; Whitmore, L.; Currie, A.; Archibad, C.; MacDonald, D.; Pennock, J.	2020	wrong diagnostic test
Pilot Study on the Current Management of Children with COVID-19 In Hospitals in Bangladesh; Findings and Implications	Nusrat, N.; Haque, M.; Chowdhury, K.; Adnan, N.; Lutfor, A. B.; Karim, E.; Hassan, M.; Rabbany, A.; Begum, D.; Hasan, M. N.; Sihan, N.; Zaman, S. U.; Islam, S.; Schellack, N.; Gowere, M.; Kurdi, A.; Godman, B.	2021	wrong diagnostic test
Clinical Features of Critical Coronavirus Disease 2019 in Children	Bhumbra, S.; Malin, S.; Kirkpatrick, L.; Khaitan, A.; John, C. C.; Rowan, C. M.; Enane, L. A.	2020	wrong diagnostic test
Acute kidney injury in critically Ill children and young adults with suspected SARS-CoV2 infection	Basu, R. K.; Bjornstad, E. C.; Gist, K. M.; Starr, M.; Khandhar, P.; Chanchlani, R.; Krallman, K. A.; Zappitelli, M.; Askenazi, D.; Goldstein, S. L.	2021	wrong diagnostic test
The impact of obesity on disease severity and outcomes among hospitalized children with COVID-19	Tripathi, S.; Christison, A. L.; Levy, E.; McGravery, J.; Tekin, A.; Bolliger, D.; Kumar, V. K.; Bansal, V.; Chiotos, K.; Gist, K. M.; Dapul, H. R.; Bhalala, U. S.; Gharpure, V. P.; Heneghan, J. A.; Gupta, N.; Bjornstad, E. C.; Montgomery, V. L.; Walkey, A.; Kashyap, R.	2021	wrong diagnostic test

Study Title	Author names	Year	Reason for exclusion
Trends in COVID-19 cases, emergency department visits, and hospital admissions among children and adolescents aged 0-17 years - United States, August 2020-August 2021	Siegel, D. A.; Reses, H. E.; Cool, A. J.; Shapiro, C. N.; Hsu, J.; Boehmer, T. K.; Cornwell, C. R.; Gray, E. B.; Henley, S. J.; Lochner, K.; Suthar, A. B.; Lyons, B. C.; Mattocks, L.; Hartnett, K.; Adjemian, J.; Santen, K. L. van; Sheppard, M.; Soetebier, K. A.; Logan, P.; Martin, M.; Idubor, O.; Natarajan, P.; Sircar, K.; Oyegun, E.; Dalton, J.; Perrine, C. G.; Peacock, G.; Schweitzer, B.; Morris, S. B.; Raizes, E.	2021	wrong diagnostic test
Severe acute respiratory syndrome in indigenous people in the context of the COVID-19's pandemic in Brazil: an analysis from the perspective of epidemiological surveillance	da Silva, W. N. T.; Rosa, M. F. P.; Mendonca, K. S.; Queiroz, G. D.; de Oliveira, S. V.	2021	wrong diagnostic test
Intriguing new faces of Covid-19: persisting clinical symptoms and cardiac effects in children	Erol, N.; Alpinar, A.; Erol, C.; Sari, E.; Alkan, K.	2021	wrong diagnostic test
Age and Hospitalization Risk in People With Type 1 Diabetes and COVID-19: Data From the T1D Exchange Surveillance Study	Demeterco-Berggren, C.; Ebekozien, O.; Rompicherla, S.; Jacobsen, L.; Accacha, S.; Gallagher, M. P.; Todd Alonso, G.; Seyoum, B.; Vendrame, F.; Haw, J. S.; Basina, M.; Levy, C. J.; Maahs, D. M.	2022	wrong diagnostic test
Clinical profile and risk factors for severe disease in 402 children hospitalized with SARS-CoV-2 from India: collaborative Indian pediatric COVID study group	Jat, K. R.; Jhuma, Sankar; Das, R. R.; Ratageri, V. H.; Bharat, Choudhary; Bhat, J. I.; Baijayantimala, Mishra; Sushma, Bhatnagar; Bijayini, Behera; Charoo, B. A.; Goyal, J. P.; Gupta, A. K.; Gulla, K. M.; Rani, Gera; Shivanand, Illalu; Kabra, S. K.; Khera, D.; Balbir, Kumar; Rakesh, Lodha; Anant, Mohan; Mohanty, P. K.; Satapathy, A. K.; Kuldeep, Singh; Amitabh, Singh; Sharma, S. V.; Pawan, Tiwari; Anjan, Trikha; Wari, P. K.	2021	wrong diagnostic test
Hospitalization of Adolescents Aged 12-17 Years with Laboratory-Confirmed COVID-19 - COVID-NET, 14 States, March 1, 2020-April 24, 2021	Havers, F. P.; Whitaker, M.; Self, J. L.; Chai, S. J.; Kirley, P. D.; Alden, N. B.; Kawasaki, B.; Meek, J.; Yousey-Hindes, K.; Anderson, E. J.; Openo, K. P.; Weigel, A.; Teno, K.; Monroe, M. L.; Ryan, P. A.; Reeg, L.; Kohrman, A.; Lynfield, R.; Como-Sabetti, K.; Poblete, M.; McMullen, C.; Muse, A.; Spina, N.; Bennett, N. M.; Gaitan, M.; Billing, L. M.; Shiltz, J.; Sutton, M.; Abdullah, N.; Schaffner, W.; Talbot, H. K.; Crossland, M.; George, A.; Patel, K.; Pham, H.;	2021	wrong diagnostic test

**Study Title**	**Author names**	**Year**	**Reason for exclusion**
	Milucky, J.; Anglin, O.; Ujamaa, D.; Hall, A. J.; Garg, S.; Taylor, C. A.; Covid-Net Surveillance, Team		
Global characteristics and outcomes of SARS-CoV-2 infection in children and adolescents with cancer (GRCCC): a cohort study	Mukkada, S.; Bhakta, N.; Chantada, G. L.; Chen, Y.; Vedaraju, Y.; Faughnan, L.; Homsi, M. R.; Muniz-Talavera, H.; Ranadive, R.; Metzger, M.; Friedrich, P.; Agulnik, A.; Jeha, S.; Lam, C.; Dalvi, R.; Hessissen, L.; Moreira, D. C.; Santana, V. M.; Sullivan, M.; Bouffet, E.; Caniza, M. A.; Devidas, M.; Pritchard-Jones, K.; Rodriguez-Galindo, C.; Global Registry of, Covid-in Childhood Cancer	2021	wrong diagnostic test
Pediatric Patients with SARS-CoV-2 Infection: Clinical Characteristics in the United States from a Large Global Health Research Network	Desai, A.; Mills, A.; Delozier, S.; Cabrera Aviles, C.; Edwards, A.; Dirajlal-Fargo, S.; McComsey, G.	2020	wrong diagnostic test
Overcrowding and exposure to secondhand smoke increase risk for COVID-19 infection among Latinx families in the greater San Francisco Bay Area	Mendez, A. D.; Escobar, M.; Romero, M.; Wojcicki, J. M.	2021	wrong diagnostic test
Clinical characteristics of SARS-CoV-2 infection in children with cystic fibrosis: An international observational study	Bain, Robert; Cosgriff, Rebecca; Zampoli, Marco; Elbert, Alexander; Burgel, Pierre-RÃ©gis; Carr, SiobhÃ¡n B.; CastaÃ±os, Claudio; Colombo, Carla; Corvol, Harriet; Faro, Albert; Goss, Christopher H.; Gutierrez, Hector; Jung, Andreas; Kashirskaya, Nataliya; Marshall, Bruce C.; Melo, Joel; Mondejar-Lopez, Pedro; de Monestrol, Isabelle; Naehrlich, Lutz; Padoan, Rita; Pastor-Vivero, Maria Dolores; Rizvi, Samar; Salvatore, Marco; Filho, Luiz Vicente Ribeiro Ferreira da Silva; Brownlee, Keith G.; Haq, Iram J.; Brodlie, Malcolm	2021	wrong diagnostic test
A Population-Based Study of COVID-19 Infection Among Childhood Cancer Survivors	Agha, M.; Leung, F.; Moineddin, R.; Bradley, N. M.; Gibson, P. J.; Hodgson, D. C.	2021	wrong diagnostic test
Comparative analysis of pediatric COVID-19 infection in Southeast Asia, South Asia, Japan, and China	Ming Wong, J. J.; Abbas, Q.; Chuah, S. L.; Malisie, R. F.; Pon, K. M.; Katsuta, T.; Dang, H.; Lee, P. C.; Jayashree, M.; Sultana, R.; Maha, Q.; Gan, C. S.; Shimizu, N.; Xu, F.; Tang, S. F.; Shi, L.; Lee, J. H.; Thoon, K. C.; Yung, C. F.	2021	wrong diagnostic test

**Study Title**	**Author names**	**Year**	**Reason for exclusion**
Prevalence and Clinical Characteristics of SARS-CoV-2 Confirmed and Negative Kawasaki Disease Patients During the Pandemic in Spain	Fernandez-Cooke, E.; Grasa, C. D.; Dominguez-Rodriguez, S.; Barrios Tascon, A.; Sanchez-Manubens, J.; Anton, J.; Mercader, B.; Villalobos, E.; Camacho, M.; Navarro Gomez, M. L.; Oltra Benavent, M.; Giralt, G.; Bustillo, M.; Bello Naranjo, A. M.; Rocandio, B.; Rodriguez-Gonzalez, M.; Nunez Cuadros, E.; Aracil Santos, J.; Moreno, D.; Calvo, C.; Kawa-Race Study, Group	2020	wrong diagnostic test
Impact of COVID-19 epidemics in paediatric morbidity and utilisation of hospital paediatric services in Italy	Manzoni, P.; Militello, M. A.; Fiorica, L.; Cappiello, A. R.; Manzionna, M.	2020	wrong diagnostic test
Covid-19 Incidence and Mortality by Age Strata and Comorbidities in Mexico City: A Focus in the Pediatric Population	Gonzalez-Garcia, N.; Castilla-Peon, M. F.; Solorzano Santos, F.; Jimenez-Juarez, R. N.; Martinez Bustamante, M. E.; Minero Hibert, M. A.; Garduno-Espinosa, J.	2021	wrong diagnostic test
Sars-Cov-2 in children - insights and conclusions from the mandatory reporting data in Frankfurt am Main, Germany, March-July 2020	Heudorf, U.; Steul, K.; Gottschalk, R.	2020	wrong diagnostic test
Racial and ethnic disparities in COVID-19 incidence by age, sex, and period among persons aged <25 years - 16 U.S. jurisdictions, January 1-December 31, 2020	Dyke, M. E. van; Mendoza, M. C. B.; Li, Wen; Parker, E. M.; Belay, B.; Davis, E. M.; Quint, J. J.; Penman-Aguilar, A.; Clarke, K. E. N.	2021	wrong diagnostic test
Association between pediatric asthma and positive tests for SARS-CoV-2 in the District of Columbia	Margolis, R. H. F.; Patel, S. J.; Sheehan, W. J.; Simpson, J. N.; Kachroo, N.; Bahar, B.; Teach, S. J.	2021	wrong diagnostic test
COVID-19 trends and severity among symptomatic children aged 0-17 years in 10 European Union countries, 3 August 2020 to 3 October 2021	Bundle, N.; Dave, N.; Pharris, A.; Spiteri, G.; Deogan, C.; Suk, J. E.	2021	wrong diagnostic test
Risks of covid-19 hospital admission and death for people with learning disability: population based cohort study using the OpenSAFELY platform	Williamson, E. J.; McDonald, H. I.; Bhaskaran, K.; Walker, A. J.; Bacon, S.; Davy, S.; Schultze, A.; Tomlinson, L.; Bates, C.; Ramsay, M.; Curtis, H. J.; Forbes, H.; Wing, K.; Minassian, C.; Tazare, J.; Morton, C. E.; Nightingale, E.; Mehrkar, A.; Evans, D.; Inglesby, P.; MacKenna, B.; Cockburn, J.; Rentsch, C. T.; Mathur, R.; Wong, A. Y.	2021	wrong diagnostic test

**Study Title**	**Author names**	**Year**	**Reason for exclusion**
	S.; Eggo, R. M.; Hulme, W.; Croker, R.; Parry, J.; Hester, F.; Harper, S.; Douglas, I. J.; Evans, S. J. W.; Smeeth, L.; Goldacre, B.; Kuper, H.		
Characterization and Outcomes of Hospitalized Children With Coronavirus Disease 2019: A Report From a Multicenter, Viral Infection and Respiratory Illness Universal Study (Coronavirus Disease 2019) Registry	Bhalala, U. S.; Gist, K. M.; Tripathi, S.; Boman, K.; Kumar, V. K.; Retford, L.; Chiotos, K.; Blatz, A. M.; Dapul, H.; Verma, S.; Sayed, I. A.; Gharpure, V. P.; Bjornstad, E.; Tofil, N.; Irby, K.; Sanders, R. C., Jr.; Heneghan, J. A.; Thomas, M.; Gupta, M. K.; Oulds, F. E.; Arteaga, G. M.; Levy, E. R.; Gupta, N.; Kaufman, M.; Abdelaty, A.; Shlomovich, M.; Medar, S. S.; Iqbal O'Meara, A. M.; Kuehne, J.; Menon, S.; Khandhar, P. B.; Miller, A. S.; Barry, S. M.; Danesh, V. C.; Khanna, A. K.; Zammit, K.; Stulce, C.; McGonagill, P. W.; Bercow, A.; Amzuta, I. G.; Gupta, S.; Almazyad, M. A.; Pierre, L.; Sendi, P.; Ishaque, S.; Anderson, H. L., 3rd; Nawathe, P.; Akhter, M.; Lyons, P. G.; Chen, C.; Walkey, A. J.; Bihorac, A.; Wada Bello, I.; Ben Ari, J.; Kovacevic, T.; Bansal, V.; Brinton, J. T.; Zimmerman, J. J.; Kashyap, R.; Society of Critical Care Medicine Discovery Viral, Infection; Respiratory Illness Universal Study, Covid-Registry Investigator Group	2022	wrong diagnostic test
Association Between Race and COVID-19 Outcomes Among 2.6 Million Children in England	Saatci, D.; Ranger, T. A.; Garriga, C.; Clift, A. K.; Zaccardi, F.; Tan, P. S.; Patone, M.; Coupland, C.; Harnden, A.; Griffin, S. J.; Khunti, K.; Dambha-Miller, H.; Hippisley-Cox, J.	2021	wrong diagnostic test
Thirty-day outcomes of children and adolescents with COVID-19: An international experience	Duarte-Salles, T.; Vizcaya, D.; Pistillo, A.; Casajust, P.; Sena, A. G.; Hui Lai, L. Y.; Prats-Uribe, A.; Ahmed, W. U. R.; Alshammari, T. M.; Alghoul, H.; Alser, O.; Burn, E.; You, S. C.; Areia, C.; Blacketer, C.; DuVall, S.; Falconer, T.; Fernandez-Bertolin, S.; Fortin, S.; Golozar, A.; Gong, M.; Tan, E. H.; Huser, V.; Iveli, P.; Morales, D. R.; Nyberg, F.; Posada, J. D.; Recalde, M.; Roel, E.; Schilling, L. M.; Shah, N. H.; Shah, K.; Suchard, M. A.; Zhang, L.; Zhang, Y.; Williams, A. E.; Reich, C. G.; Hripcsak, G.; Rijnbeek, P.; Ryan, P.; Kostka, K.; Prieto-Alhambra, D.	2021	wrong diagnostic test
Provisional mortality data - United States, 2020	Ahmad, F. B.; Cisewski, J. A.; Minino, A.; Anderson, R. N.	2021	wrong diagnostic test

Study Title	Author names	Year	Reason for exclusion
Demographic predictors of hospitalization and mortality in US children with COVID-19	Chorath, K.; Rajasekaran, K.; Burmeister, F.; Ahmed, M.; Moreira, A.	2021	wrong diagnostic test
Factors associated with severe SARS-CoV-2 infection	Ouldali, N.; Yang, DaWei; Madhi, F.; Levy, M.; Gaschignard, J.; Craiu, I.; Guiddir, T.; Schweitzer, C.; Wiedemann, A.; Lorrot, M.; Romain, A. S.; Garraffo, A.; Haas, H.; Rouget, S.; Pontual, L. de; Aupiais, C.; Martinot, A.; Toubiana, J.; Dupic, L.; Minodier, P.; Passard, M.; Belot, A.; Levy, C.; Bechet, S.; Jung, C.; Sarakbi, M.; Ducrocq, S.; Danekova, N.; Jhaouat, I.; Vignaud, O.; Garrec, N.; Caron, E.; Cohen, R.; Gajdos, V.; Angoulvant, F.	2021	wrong diagnostic test
The younger the milder clinical course of COVID-19: Even in newborns?	Leung, C.	2021	wrong diagnostic test
Characteristics of Hospitalized Pediatric Coronavirus Disease 2019 Cases in Chicago, Illinois, March-April 2020	Mannheim, J.; Gretsch, S.; Layden, J. E.; Fricchione, M. J.	2020	wrong diagnostic test
COVID-19-Associated Pulmonary Embolism in Pediatric Patients	Chima, M.; Williams, D.; Thomas, N. J.; Krawiec, C.	2021	wrong diagnostic test
Coronavirus disease 2019 in children - United States, February 12-April 2, 2020	Cdc Covid- Response Team	2020	wrong diagnostic test
COVID-19 and Paediatric Inflammatory Bowel Diseases: Global Experience and Provisional Guidance (March 2020) from the Paediatric IBD Porto group of ESPGHAN	Turner, Dan; Ying, Huang; MartÃn-de-Carpi, Javier; Aloi, Marina; Focht, Gili; Ben, Kang; Ying, Zhou; Sanchez, Cesar; Kappelman, Michael D.; Uhlig, Holm H.; Pujol-Muncunill, Gemma; Ledder, Oren; Lionetti, Paolo; Dias, Jorge Amil; Ruemmele, Frank M.; Russell, Richard K.; Huang, Ying; Kang, Ben; Zhou, Ying	2020	wrong diagnostic test
The Impact of Coronavirus Disease 2019 Pandemic on U.S. and Canadian PICUs	Sachdeva, R.; Rice, T. B.; Reisner, B.; Brundage, N.; Hulbert, C.; Kaminski, A.; Wetzel, R. C.	2020	wrong diagnostic test
Open schools, Covid-19, and child and teacher morbidity in Sweden	Ludvigsson, J. F.; Engerstrom, L.; Nordenhall, C.; Larsson, E.	2021	wrong diagnostic test
National Trends in Disease Activity for COVID-19 Among Children in the US	Hutch, M. R.; Liu, M.; Avillach, P.; Luo, Y.; Bourgeois, F. T.	2021	wrong diagnostic test
Comparison of First and Second Wave Cohorts of Multisystem Inflammatory Disease Syndrome IN Children	Harahsheh, Ashraf S.; Sharron, Matthew P.; Bost, James E.; Ansusinha, Emily; Wessel, David; DeBiasi, Roberta L.	2022	wrong diagnostic test

Study Title	Author names	Year	Reason for exclusion
Severe Coronavirus Disease-2019 in Children and Young Adults in the Washington, DC, Metropolitan Region	DeBiasi, R. L.; Song, X.; Delaney, M.; Bell, M.; Smith, K.; Pershad, J.; Ansusinha, E.; Hahn, A.; Hamdy, R.; Harik, N.; Hanisch, B.; Jantausch, B.; Koay, A.; Steinhorn, R.; Newman, K.; Wessel, D.	2020	wrong diagnostic test
Comorbidities are risk factors for hospitalization and serious COVID-19 illness in children and adults with sickle cell disease	Mucalo, L.; Brandow, A. M.; Dasgupta, M.; Mason, S. F.; Simpson, P. M.; Singh, A.; Taylor, B. W.; Woods, K. J.; Yusuf, F. I.; Panepinto, J. A.	2021	wrong diagnostic test
Epidemiological characteristics and transmission dynamics of paediatric cases with coronavirus disease 2019 in Hubei province, China	Wang, M.; Nie, X.; Huang, S.; Pi, W.; Wang, D.; Zhou, M.; Ma, J.; Li, M.; Chen, W.	2021	wrong diagnostic test
Acute Kidney Injury in Pediatric Inflammatory Multisystem Syndrome Temporally Associated With Severe Acute Respiratory Syndrome Coronavirus-2 Pandemic: Experience From PICUs Across United Kingdom	Deep, A.; Upadhyay, G.; du Pre, P.; Lillie, J.; Pan, D.; Mudalige, N.; Kanthimathinathan, H. K.; Johnson, M.; Riphagen, S.; Dwarakanathan, B.; Raffaj, D.; Sundararajan, S.; Davies, P.; Mohammad, Z.; Shetty, N.; Playfor, S.; Jardine, M.; Ross, O.; Levin, R.; Waters, G.; Sinha, R.; Scholefield, B. R.; Boot, E.; Koul, A.; Freire-Gomez, X.; Ramnarayan, P.	2020	wrong diagnostic test
Coronavirus disease 2019-associated PICU admissions: a report from the society of critical care medicine discovery network viral infection and respiratory illness universal study registry	Sandeep, Tripathi; Gist, K. M.; Bjornstad, E. C.; Kashyap, R.; Boman, K.; Chiotos, K.; Gharpure, V. P.; Dapul, H.; Sayed, I. A.; Kuehne, J.; Heneghan, J. A.; Manoj, Gupta; Khandhar, P. B.; Menon, S.; Neha, Gupta; Kumar, V. K.; Retford, L. C.; Zimmerman, J.; Bhalala, U. S.	2021	wrong diagnostic test
Impact of environmental and individual factors on COVID-19 mortality in children and adolescents in Mexico: An observational study	Sanchez-Piedra, C.; Gamino-Arroyo, A. E.; Cruz-Cruz, C.; Prado-Galbarro, F. J.	2022	wrong diagnostic test
Assessment of 135794 Pediatric Patients Tested for Severe Acute Respiratory Syndrome Coronavirus 2 Across the United States	Bailey, L. C.; Razzaghi, H.; Burrows, E. K.; Bunnell, H. T.; Camacho, P. E. F.; Christakis, D. A.; Eckrich, D.; Kitzmiller, M.; Lin, S. M.; Magnusen, B. C.; Newland, J.; Pajor, N. M.; Ranade, D.; Rao, S.; Sofela, O.; Zahner, J.; Bruno, C.; Forrest, C. B.	2021	wrong diagnostic test
Asthma and allergic diseases are not risk factors for hospitalization in children with coronavirus disease 2019	Beken, B.; Ozturk, G. K.; Aygun, F. D.; Aydogmus, C.; Akar, H. H.	2021	wrong diagnostic test

Study Title	Author names	Year	Reason for exclusion
Pregnancy and neonatal outcomes of COVID-19: coreporting of common outcomes from PAN-COVID andÂ AAP-SONPM registries	Mullins, E.; Hudak, M. L.; Banerjee, J.; Getzlaff, T.; Townson, J.; Barnette, K.; Playle, R.; Perry, A.; Bourne, T.; Lees, C. C.	2021	wrong diagnostic test
Prevalence of thrombotic complications in children with SARS-CoV-2	Aguilera-Alonso, D.; Murias, S.; Garde, A. M. de A.; Soriano-Arandes, A.; Pareja, M.; Otheo, E.; Moraleda, C.; Tagarro, A.; Calvo, C	2021	wrong diagnostic test
COVID-19 hospitalization rate in children across a private hospital network in the United States: COVID-19 hospitalization rate in children	Kim, T. Y.; Kim, E. C.; Agudelo, A. Z.; Friedman, L.	2021	wrong diagnostic test
Characteristics and outcomes of pediatric covid-19 patients in osaka, japan	Katayama, Y.; Zha, L.; Kitamura, T.; Hirayama, A.; Takeuchi, T.; Tanaka, K.; Komukai, S.; Shimazu, T.; Sobue, T.	2021	wrong diagnostic test
Risk of COVID-19 hospital admission among children aged 5-17 years with asthma in Scotland: a national incident cohort study	Shi, T.; Pan, J.; Katikireddi, S. V.; McCowan, C.; Kerr, S.; Agrawal, U.; Shah, S. A.; Simpson, C. R.; Ritchie, L. D.; Robertson, C.; Sheikh, A.	2022	wrong diagnostic test
Epidemiology of COVID-19 in Indonesia: common source and propagated source as a cause for outbreaks	Hikmawati, I.; Setiyabudi, R	2021	wrong diagnostic test
Characteristics in Pediatric Patients with Coronavirus Disease 2019 in Korea	Seon, J. Y.; Jeon, W. H.; Bae, S. C.; Eun, B. L.; Choung, J. T.; Oh, I. H.	2021	wrong diagnostic test
Do malnutrition, pre-existing morbidities, and poor household environmental conditions aggravate susceptibility to Coronavirus disease (COVID-19)? A study on under-five children in India	Saha, J.; Chouhan, P.	2021	wrong diagnostic test
Severe Acute Respiratory Syndrome Coronavirus 2 Infections in Primary School Age Children After Partial Reopening of Schools in England	Powell, A. A.; Amin-Chowdhury, Z.; Mensah, A.; Ramsay, M. E.; Saliba, V.; Ladhani, S. N.	2021	wrong diagnostic test
COVID-19, Australia: Epidemiology Report 25 (Fortnightly reporting period ending 13 September 2020)	Covid- National Incident Room Surveillance, Team	2020	wrong outcome-incidence per 100,000 given but no 95% CI

Study Title	Author names	Year	Reason for exclusion
COVID-19, Australia: Epidemiology Report 18 (Fortnightly reporting period ending 7 June 2020)	Covid- National Incident Room Surveillance, Team	2020	wrong outcome- incidence per 100,000 given but no 95% CI
Mass Events Trigger Malta's Second Peak After Initial Successful Pandemic Suppression	Cuschieri, Sarah; Balzan, Martin; Gauci, Charmaine; Aguis, Steve; Grech, Victor	2021	wrong outcomes
SARS-CoV-2 transmission in schools in Korea: nationwide cohort study	Choe, Young June; Park, Young-Joon; Kim, Eun-Young; Jo, Myoungyoun; Cho, Eun Young; Lee, Hyunju; Kim, Yun-Kyung; Kim, Yae-Jean; Choi, Eun Hwa	2021	wrong outcomes
Incidence of COVID-19 in children and young people who play federated football	Bestilleiro, R. S.; Hern..ndez, J. S.; Bautista, D. B.; Rodr..guez, M. J. P.; Mart..n, C. G.; Rodriguez, M. T. G.; D..az, S. P.	2022	wrong outcomes
[Differential clinic in children infected by SARS-CoV-2, traceability of contacts and cost-effectiveness of diagnostic tests: Cross-sectional observational study]	Fiel-Ozores, A.; Gonzalez-Duran, M. L.; Novoa-Carballal, R.; Portugues-de la Red, M. D. M.; Fernandez-Pinilla, I.; Cabrera-Alvargonzalez, J. J.; Martinez-Reglero, C.; Rey-Cao, S.; Concheiro-Guisan, A.	2021	wrong outcomes
Characterization of coronavirus disease 2019 (COVID-19) in children and adolescents in Latin American and the Caribbean countries: A descriptive study. [Spanish]	Atamari-Anahui, N.; Cruz-Nina, N. D.; Condori-Huaraka, M.; Nunez-Paucar, H.; Rondon-Abuhadba, E. A.; Ordonez-Linares, M. E.; Pereira-Victoriod, C. J.	2020	wrong outcomes
CRIANÃ‡AS COM SÃ• NDROME RESPIRATÃ“RIA AGUDA GRAVE (SRAG) CONFIRMADA EM HOSPITAL DE REFERÃŠNCIA PEDIÃ• TRICA EM BELO HORIZONTE, MINAS GERAIS	Oliveira e Silva, M. A.; Bentes, A. A.; Cunha, A. L. G.; Ramos, L. D. A.; do Amaral, D. B.; Nascimento, P. F. S. D.; Assis, P. A.; Pinto, C. M. T.; da Silva, D. R. L.; Paiva, S. V.; de Souza, D. B.; Moreira, L. M.	2022	wrong outcomes
[Case Series of 103 Children with SARS-CoV-2 Infection in Portugal]	Picao de Carvalho, C.; Castro, C.; Sampaio Graca, I.; Lorenzo, C.; Barbosa Rodrigues, A.; Inacio, R.; Prata, F.; Mouzinho, A.; Pinto, S.; Marques, J. G.	2020	wrong outcomes
Respiratory viruses detected through molecular biology in childrenhospitalized	De Leon, I. R. P.; Cruz, S.; Rojas, J. P.	2021	wrong outcomes

Study Title	Author names	Year	Reason for exclusion
for acute respiratory infection in times of SARS-CoV-2/COVID-19. [Spanish]			
Nuevos diagnÃ³sticos de diabetes mellitus tipo 1 en niÃ±os durante la pandemia COVID-19. Estudio multicÃ©ntrico regional en EspaÃ±a	Herrero, MarÃa HernÃ¡ndez Mercader Pilar Terradas Martinez Ester Latorre Rovira Albert Feliu Zaragoza Neus RodrÃguez Ricart Ester Parada	2022	wrong outcomes
Effectiveness of Maternal Vaccination with mRNA COVID-19 Vaccine During Pregnancy Against COVID-19-Associated Hospitalization in Infants Aged <6 Months - 17 States, July 2021-January 2022	Halasa, N. B.; Olson, S. M.; Staat, M. A.; Newhams, M. M.; Price, A. M.; Boom, J. A.; Sahni, L. C.; Cameron, M. A.; Pannaraj, P. S.; Bline, K. E.; Bhumbra, S. S.; Bradford, T. T.; Chiotos, K.; Coates, B. M.; Cullimore, M. L.; Cvijanovich, N. Z.; Flori, H. R.; Gertz, S. J.; Heidemann, S. M.; Hobbs, C. V.; Hume, J. R.; Irby, K.; Kamidani, S.; Kong, M.; Levy, E. R.; Mack, E. H.; Maddux, A. B.; Michelson, K. N.; Nofziger, R. A.; Schuster, J. E.; Schwartz, S. P.; Smallcomb, L.; Tarquinio, K. M.; Walker, T. C.; Zinter, M. S.; Gilboa, S. M.; Polen, K. N.; Campbell, A. P.; Randolph, A. G.; Patel, M. M.; Overcoming, Covid-Investigators; Overcoming, Covid-Network	2022	wrong outcomes
Clinical spectrum of SARS-CoV-2 infection and protection from symptomatic re-infection	Maier, H. E.; Kuan, G.; Saborio, S.; Bustos Carrillo, F. A.; Plazaola, M.; Barilla, C.; Sanchez, N.; Lopez, R.; Smith, M.; Kubale, J.; Ojeda, S.; Zuniga-Moya, J. C.; Carlson, B.; Lopez, B.; Gajewski, A. M.; Chowdhury, M.; Harris, E.; Balmaseda, A.; Gordon, A.	2021	wrong outcomes
Hospitalizations of Children and Adolescents with Laboratory-Confirmed COVID-19 - COVID-NET, 14 States, July 2021-January 2022	Marks, K. J.; Whitaker, M.; Anglin, O.; Milucky, J.; Patel, K.; Pham, H.; Chai, S. J.; Kirley, P. D.; Armistead, I.; McLafferty, S.; Meek, J.; Yousey-Hindes, K.; Anderson, E. J.; Openo, K. P.; Weigel, A.; Henderson, J.; Nunez, V. T.; Como-Sabetti, K.; Lynfield, R.; Ropp, S. L.; Smelser, C.; Barney, G. R.; Muse, A.; Bennett, N. M.; Bushey, S.; Billing, L. M.; Shiltz, E.; Abdullah, N.; Sutton, M.; Schaffner, W.; Talbot, H. K.; Chatelain, R.; George, A.; Taylor, C. A.; McMorrow, M. L.; Perrine, C. G.; Havers, F. P.; Covid-Net Surveillance, Team	2022	wrong outcomes
Patterns and descriptors of COVID-19 testing and lab-confirmed COVID-19 incidence in Manitoba, Canada, March 2020-May 2021: A population-based study	Righolt, Christiaan H.; Zhang, Geng; Sever, Emrah; Wilkinson, Krista; Mahmud, Salaheddin M.	2021	wrong outcomes

Study Title	Author names	Year	Reason for exclusion
Temporal rise in the proportion of younger adults and older adolescents among coronavirus disease (COVID-19) cases following the introduction of physical distancing measures, Germany, March to April 2020	Goldstein, E.; Lipsitch, M.	2020	wrong outcomes
Serum zinc levels in pediatric patients with COVID-19	Ekemen Keles, Y.; Yilmaz Ciftdogan, D.; Colak, A.; Kara Aksay, A.; Ustundag, G.; Sahin, A.; Yilmaz, N.	2022	wrong outcomes
Comparison of clinical and laboratory features in coronavirus disease 2019 and pediatric multisystem inflammatory syndrome patients	Yakut, N.; Yuksel, E.; Algul, M.; Armut, M.; Sahin, B.; Karagoz, G.; Yakut, K.; Kilinc, A.; Tanidir, I. C.	2022	wrong outcomes
Clinical Characteristics and Histopathology of Coronavirus Disease 2019-Related Deaths in African Children	Mabena, F. C.; Baillie, V. L.; Hale, M. J.; Thwala, B. N.; Mthembu, N.; Els, T.; Serafin, N.; du Plessis, J.; Swart, P.; Velaphi, S. C.; Petersen, K. L.; Wadula, J.; Govender, N. P.; Verwey, C.; Moore, D. P.; Moosa, F. Y.; Nakwa, F. L.; Maroane, B. V.; Okudo, G.; Mabaso, T. M.; Dangor, Z.; Nunes, M. C.; Madhi, S. A.	2021	wrong outcomes
Characteristics and Outcomes of US Children and Adolescents With Multisystem Inflammatory Syndrome in Children (MIS-C) Compared With Severe Acute COVID-19	Feldstein, L. R.; Tenforde, M. W.; Friedman, K. G.; Newhams, M.; Rose, E. B.; Dapul, H.; Soma, V. L.; Maddux, A. B.; Mourani, P. M.; Bowens, C.; Maamari, M.; Hall, M. W.; Riggs, B. J.; Giuliano, J. S., Jr.; Singh, A. R.; Li, S.; Kong, M.; Schuster, J. E.; McLaughlin, G. E.; Schwartz, S. P.; Walker, T. C.; Loftis, L. L.; Hobbs, C. V.; Halasa, N. B.; Doymaz, S.; Babbitt, C. J.; Hume, J. R.; Gertz, S. J.; Irby, K.; Clouser, K. N.; Cvijanovich, N. Z.; Bradford, T. T.; Smith, L. S.; Heidemann, S. M.; Zackai, S. P.; Wellnitz, K.; Nofziger, R. A.; Horwitz, S. M.; Carroll, R. W.; Rowan, C. M.; Tarquinio, K. M.; Mack, E. H.; Fitzgerald, J. C.; Coates, B. M.; Jackson, A. M.; Young, C. C.; Son, M. B. F.; Patel, M. M.; Newburger, J. W.; Randolph, A. G.; Overcoming, Covid-Investigators	2021	wrong outcomes
COVID-19 Pandemic: A comparison of adult and pediatric populations	Demirkol, Muhammed Emin Kaya Musa Balci Mehmet Ozsari Emine	2021	wrong outcomes
Impact of Ethnicity on COVID-19 Related Hospitalizations in Children	Baronio, R.; Savare, L.; Ruggiero, J.; Crotti, B.; Mazza, A.; Marseglia, G. L.; Dodi, I.; Cavalli, C.; Schumacher, R. F.	2021	wrong outcomes

**Study Title**	**Author names**	**Year**	**Reason for exclusion**
During the First Pandemic Wave in Northern Italy			
Decreased risk of COVID-19 pneumonia in children and adolescents during the Delta variant emergence	Murillo-Zamora, E.; Trujillo, X.; Huerta, M.; Rios-Silva, M.; Baltazar-Rodriguez, L. M.; Guzman-Esquivel, J.; Benites-Godinez, V.; Ortega-Ramirez, A. D.; Mendoza-Cano, O.	2021	wrong outcomes
Pediatric Hospitalizations after School Reopening during the SARS-CoV-2 Alpha (B.1.1.7) Variant Spread: A Multicenter Cross-sectional Study in Israel	Friedman, N.; Levy, N.; Kaplan, O.; Padeh, G.; Krupik, D.; Jacob, R.; Gamsu, S.; Weiser, G.; Cohen, N. K.; Schnapp, Z.; Cohen, N.; Feldman, O.; Porat, D.; Gal, M.; Gleyzer, A.; Capua, T.; Klein, A.; Sharkansky, L.; Shilo, S.; Grotto, I.; Kozer, E.; Shavit, I.	2022	wrong outcomes
Household Transmission of SARS-CoV-2: A Prospective Longitudinal Study Showing Higher Viral Load and Increased Transmissibility of the Alpha Variant Compared to Previous Strains	Julin, Cathinka Halle; Robertson, Anna Hayman; Hungnes, Olav; Tunheim, Gro; Bekkevold, Terese; Laake, Ida; Aune, Idunn Forland; Killengreen, Marit Fodnes; Strand, Torunn Ramsem; Rykkvin, Rikard; Dorenberg, Dagny Haug; Stene-Johansen, Kathrine; Berg, Einar Sverre; Bodin, Johanna Eva; Oftung, Fredrik; Steens, Anneke; NÃ¦ss, Lisbeth Meyer	2021	wrong outcomes
Demographic and epidemiological characterization of COVID-19 confirmed cases in Diwaniya Governorate, 2020	Suhail, H. J.; Waleed, S. M.; Hussien, Y. A.; Kadhim, S. F.	2021	wrong outcomes
Iranian Pediatric COVID-19 Epidemiology and Clinical Characteristics	Armin, S.; Mirkarimi, M.; Pourmoghaddas, Z.; Tariverdi, M.; Jafrasteh, A.; Marhamati, N.; Shirvani, A.; Karimi, A.; Rafiei Tabatabaei, S.; Mansour Ghanaei, R.; Fahimzad, S.; Shirvani, F.; Hoseini-Alfatemi, S. M.	2021	wrong outcomes
Quarantine at home may not enough!-from the epidemiological data in Shaanxi Province of China	Shi, L.; Li, Q.; Li, K.; Zheng, J.; He, Y.; Zhang, X.; Gong, X.; Wang, W.; Zhang, Q.; Dai, C.; Zhao, W.; Meng, X.; Du, F.; Fan, P.; Li, C.; Gao, C.; Yang, Y.; Liu, X.; Chen, Y.; Liu, J.; Li, J.; Yang, N.; Niu, Y.; Chen, H.; Zhang, G.; Yan, T.; Zhu, L.; Han, Q.; Fan, W.; Ye, F.; Liu, Z.; Lin, S.; Zhao, Y.; Chen, T.	2020	wrong outcomes
Quantitative detection of SARS-CoV-2 RNA in nasopharyngeal samples from infected patients with mild disease	Bustos, P.; Tambley, C.; Acevedo, A.; Andrade, W.; Leal, G.; Vidal, D.; Roldan, F.; Fasce, R.; Ramirez, E.	2021	wrong outcomes

Study Title	Author names	Year	Reason for exclusion
On the increasing incidence of SARS-CoV- 2 in older adolescents and younger adults during the epidemic in Mexico	Stern, D.; Lajous, M.; De la Rosa, B.; Goldstein, E.	2021	wrong outcomes
Effectiveness of Pfizer-BioNTech mRNA Vaccination Against COVID-19 Hospitalization Among Persons Aged 12-18 Years - United States, June-September 2021	Olson, S. M.; Newhams, M. M.; Halasa, N. B.; Price, A. M.; Boom, J. A.; Sahni, L. C.; Irby, K.; Walker, T. C.; Schwartz, S. P.; Pannaraj, P. S.; Maddux, A. B.; Bradford, T. T.; Nofziger, R. A.; Boutselis, B. J.; Cullimore, M. L.; Mack, E. H.; Schuster, J. E.; Gertz, S. J.; Cvijanovich, N. Z.; Kong, M.; Cameron, M. A.; Staat, M. A.; Levy, E. R.; Chatani, B. M.; Chiotos, K.; Zambrano, L. D.; Campbell, A. P.; Patel, M. M.; Randolph, A. G.; Overcoming, Covid-Investigators	2021	wrong outcomes
COVID-19, Australia: Epidemiology Report 19 (Fortnightly reporting period ending 21 June 2020)	Covid- National Incident Room Surveillance, Team	2020	wrong outcomes
Incidence of Kawasaki disease before and during the COVID-19 pandemic: a retrospective cohort study in Japan	Iio, K.; Matsubara, K.; Miyakoshi, C.; Ota, K.; Yamaoka, R.; Eguchi, J.; Matsumura, O.; Okutani, T.; Ueda, I.; Nishiyama, M.	2021	wrong outcomes
Saliva as a promising biofluid for SARS-CoV-2 detection during the early stages of infection	Lopez-Martinez, B.; Guzman-Ortiz, A. L.; Nevarez-Ramirez, A. J.; Parra-Ortega, I.; Olivar-Lopez, V. B.; Angeles-Floriano, T.; Vilchis-Ordonez, A.; Quezada, H.	2020	wrong outcomes
COVID-19 in Children: Clinical Characteristics and Follow-Up Study	Ruan, P. S.; Xu, H. Q.; Wu, J. H.; Song, Q. F.; Qiu, H. Y.	2020	wrong outcomes
COVID-19 cases and transmission in 17 K-12 schools - Wood County, Wisconsin, August 31-November 29, 2020	Falk, A.; Benda, A.; Falk, P.; Steffen, S.; Wallace, Z.; Hoeg, T. B.	2021	wrong outcomes
Transmission Dynamics of COVID-19 Outbreaks Associated with Child Care Facilities - Salt Lake City, Utah, April-July 2020	Lopez, A. S.; Hill, M.; Antezano, J.; Vilven, D.; Rutner, T.; Bogdanow, L.; Claflin, C.; Kracalik, I. T.; Fields, V. L.; Dunn, A.; Tate, J. E.; Kirking, H. L.; Kiphibane, T.; Risk, I.; Tran, C. H.	2020	wrong outcomes
SARS-CoV-2 transmission in educational settings during an early summer epidemic wave in Luxembourg, 2020	Mossong, J.; Mombaerts, L.; Veiber, L.; Pastore, J.; Coroller, G. L.; Schnell, M.; Masi, S.; Huiart, L.; Wilmes, P.	2021	wrong outcomes

Study Title	Author names	Year	Reason for exclusion
Pediatric transplantation in Europe during the COVID-19 pandemic: Early impact on activity and healthcare	Dona, D.; Torres Canizales, J.; Benetti, E.; Cananzi, M.; De Corti, F.; Calore, E.; Hierro, L.; Ramos Boluda, E.; Melgosa Hijosa, M.; Garcia Guereta, L.; Perez Martinez, A.; Barrios, M.; Costa Reis, P.; Teixeira, A.; Lopes, M. F.; Kalicinski, P.; Branchereau, S.; Boyer, O.; Debray, D.; Sciveres, M.; Wennberg, L.; Fischler, B.; Barany, P.; Baker, A.; Baumann, U.; Schwerk, N.; Nicastro, E.; Candusso, M.; Toporski, J.; Sokal, E.; Stephenne, X.; Lindemans, C.; Miglinas, M.; Rascon, J.; Jara, P.; E. R. N. TransplantChild	2020	wrong outcomes
Ocular Manifestations and Clinical Characteristics of Children With Laboratory-Confirmed COVID-19 in Wuhan, China	Ma, N.; Li, P.; Wang, X.; Yu, Y.; Tan, X.; Chen, P.; Li, S.; Jiang, F.	2020	wrong outcomes
Comparison of RT-PCR, lung CT Scan, and Anti-COVID-19 Antibody Results in Hospitalized Children Suspected for COVID- 19	Nafei, Z.; Akbarian, E.; Naserzadeh, N.; Ferdosian, F.	2021	wrong outcomes
Seizures as the main presenting manifestation of acute SARS-CoV-2 infection in children	Kurd, Mohammad; Hashavya, Saar; Benenson, Shmuel; Gilboa, Tal	2021	wrong outcomes
Infectivity of severe acute respiratory syndrome coronavirus 2 in children compared with adults	Bullard, J.; Funk, D.; Dust, K.; Garnett, L.; Tran, K.; Bello, A.; Strong, J. E.; Lee, S. J.; Waruk, J.; Hedley, A.; Alexander, D.; Van Caeseele, P.; Loeppky, C.; Poliquin, G.	2021	wrong outcomes
Mask Use and Ventilation Improvements to Reduce COVID-19 Incidence in Elementary Schools - Georgia, November 16-December 11, 2020	Gettings, J.; Czarnik, M.; Morris, E.; Haller, E.; Thompson-Paul, A. M.; Rasberry, C.; Lanzieri, T. M.; Smith-Grant, J.; Aholou, T. M.; Thomas, E.; Drenzek, C.; MacKellar, D.	2021	wrong outcomes
Reopening Schools and the Dynamics of SARS-CoV-2 Infections in Israel: A Nationwide Study	Somekh, Ido; Shohat, Tamy; Boker, Lital Keinan; SimÃµes, Eric A. F.; Somekh, Eli	2021	wrong outcomes
Changes in children's surgical services during the COVID-19 pandemic at a tertiary-level government hospital in a lower middle-income country	Farooq, Md Abdullah Al; Kabir, S. M. Humayun; Chowdhury, Tanvir Kabir; Sadia, Ayesha; Alam, Md Afruzul; Farhad, Tanzil	2021	wrong outcomes

Study Title	Author names	Year	Reason for exclusion
Reopening schools in the context of increasing COVID-19 community transmission: The French experience	Gras-Le Guen, C.; Cohen, R.; Rozenberg, J.; Launay, E.; Levy-Bruhl, D.; Delacourt, C.	2021	wrong outcomes
Surveillance of COVID-19 school outbreaks, Germany, March to August 2020	Im Kampe, E. O.; Lehfeld, A. S.; Buda, S.; Buchholz, U.; Haas, W.	2020	wrong outcomes
Comparison of hospitalized patients with pneumonia caused by COVID-19 and influenza A in children under 5 years	Li, Y.; Wang, H.; Wang, F.; Du, H.; Liu, X.; Chen, P.; Wang, Y.; Lu, X.	2020	wrong outcomes
Surge in SARS-CoV-2 transmission in school-aged children and household contacts, England, August to October 2021	Chudasama, D. Y.; Tessier, E.; Flannagan, J.; Leeman, D.; Webster, H.; Demirjian, A.; Falconer, C.; Thelwall, S.; Kall, M.; Saliba, V.; Ramsay, M.; Dabrera, G.; Lamagni, T.	2021	wrong outcomes
Weekly SARS-CoV-2 sentinel surveillance in primary schools, kindergartens, and nurseries, Germany, June-November 2020	Hoch, M.; Vogel, S.; Kolberg, L.; Dick, E.; Fingerle, V.; Eberle, U.; Ackermann, N.; Sing, A.; Huebner, J.; Rack-Hoch, A.; Schober, T.; von Both, U.	2021	wrong outcomes
Risk factors associated with household transmission of SARS-CoV-2 in Negeri Sembilan, Malaysia	Ng, David Chun-Ern; Tan, Kah Kee; Chin, Ling; Cheng, Xiang Lin; Vijayakulasingam, Thalitha; Liew, Damian Wen Xian; Zainol Abidin, Nor Zanariah; Lee, Ming Lee; Ganasegeran, Kurubaran; Khoo, Erwin Jiayuan	2021	wrong outcomes
Pediatric COVID-19 Cases Prelockdown and Postlockdown in Italy	Bellino, S.; Rota, M. C.; Riccardo, F.; Andrianou, X.; Mateo Urdiales, A.; Del Manso, M.; Punzo, O.; Bella, A.; Villani, A.; Pezzotti, P.; Covid- Working, Group	2021	wrong outcomes
COVID-19 in Primary and Secondary School Settings During the First Semester of School Reopening - Florida, August-December 2020	Doyle, T.; Kendrick, K.; Troelstrup, T.; Gumke, M.; Edwards, J.; Chapman, S.; Propper, R.; Rivkees, S. A.; Blackmore, C.	2021	wrong outcomes
SARS-CoV-2 infection and transmission in educational settings: a prospective, cross-sectional analysis of infection clusters and outbreaks in England	Ismail, S. A.; Saliba, V.; Lopez Bernal, J.; Ramsay, M. E.; Ladhani, S. N.	2021	wrong outcomes

Study Title	Author names	Year	Reason for exclusion
Continued proportional age shift of confirmed positive COVID-19 incidence over time to children and young adults: Washington State March-August 2020	Malmgren, J.; Guo, B.; Kaplan, H. G.	2021	wrong outcomes
Reopening schools in a context of low COVID-19 contagion: consequences for teachers, students and their parents	GodÃ¸y, Anna; GrÃ¸tting, Maja Weemes; Hart, Rannveig Kaldager	2022	wrong outcomes
Combined RT-qPCR and pyrosequencing of a Spike glycoprotein polybasic cleavage motif can uncover pediatric SARS-CoV-2 infections associated with heterogeneous presentation	Weil, P. P.; Hentschel, J.; Schult, F.; Pembaur, A.; Ghebremedhin, B.; Mboma, O.; Heusch, A.; Reuter, A. C.; Muller, D.; Wirth, S.; Aydin, M.; Jenke, A. C. W.; Postberg, J.	2021	wrong outcomes
Re-opening schools in Croatia did not have a negative impact on children under 14, but it could not be ruled out in older children	Belavic, A.; Dimnjakovic, J.; Istvanovic, A.; Svajda, M.; Poljicanin, T.; Pavic Simetin, I.	2021	wrong outcomes
Age-dependency of the Propagation Rate of Coronavirus Disease 2019 Inside School Bubble Groups in Catalonia, Spain	Alonso, S.; Alvarez-Lacalle, E.; Catala, M.; Lopez, D.; Jordan, I.; Garcia-Garcia, J. J.; Soriano-Arandes, A.; Lazcano, U.; Salles, P.; Masats, M.; Urrutia, J.; Gatell, A.; Capdevila, R.; Soler-Palacin, P.; Bassat, Q.; Prats, C.	2021	wrong outcomes
Pediatric Kawasaki Disease Trends Prior to and During COVID-19 Outbreak in Singapore	Chong, C. Y.; Tan, N. W. H.; Yung, C. F.; Nadua, K.; Kam, K. Q.; Li, J.; Thoon, K. C.	2021	wrong outcomes
Coronavirus disease spread during summer vacation, Israel, 2020	Somekh, I.; Sim..es, E. A. F.; Somekh, E.	2021	wrong outcomes
A Description of COVID-19-Directed Therapy in Children Admitted to US Intensive Care Units 2020	Schuster, J. E.; Halasa, N. B.; Nakamura, M.; Levy, E. R.; Fitzgerald, J. C.; Young, C. C.; Newhams, M. M.; Bourgeois, F.; Staat, M. A.; Hobbs, C. V.; Dapul, H.; Feldstein, L. R.; Jackson, A. M.; Mack, E. H.; Walker, T. C.; Maddux, A. B.; Spinella, P. C.; Loftis, L. L.; Kong, M.; Rowan, C. M.; Bembea, M. M.; McLaughlin, G. E.; Hall, M. W.; Babbitt, C. J.; Maamari, M.; Zinter, M. S.; Cvijanovich, N. Z.; Michelson, K. N.; Gertz, S. J.; Carroll, C. L.; Thomas, N. J.; Giuliano, J. S.; Singh, A. R.; Hymes, S. R.; Schwarz, A. J.; McGuire, J. K.;	2022	wrong outcomes

Study Title	Author names	Year	Reason for exclusion
	Nofziger, R. A.; Flori, H. R.; Clouser, K. N.; Wellnitz, K.; Cullimore, M. L.; Hume, J. R.; Patel, M.; Randolph, A. G.; Overcoming, Covid-Investigators		
Epidemiological characteristics of children with coronavirus at a joint commission-accredited hospital in the United Arab Emirates	Al Mansoori, Latifa; Al Kaabi, Salwa; Nair, Satish Chandrasekhar; Al Katheeri, Manal; Ghatasheh, Ghassan; Al Dhanhani, Huda; Al Kaabi, Aysha	2021	wrong outcomes
Obesity and immunodeficiencies are the main preâ€• existing conditions associated with mild to moderate COVIDâ€• 19 in children	Leonâ€• Abarca, Juan Alonso	2020	wrong outcomes
Obstetric, maternal, and neonatal outcomes in COVID-19 compared to healthy pregnant women in Iran: a retrospective, case-control study	Taghavi, Seyed-Abdolvahab; Heidari, Solmaz; Jahanfar, Shayesteh; Amirjani, Shakiba; Aji-Ramkani, Amireh; Azizi-Kutenaee, Maryam; Bazarganipour, Fatemeh	2021	wrong outcomes
Eff ectiveness of bnt162b2 vaccine in adolescents during outbreak of sars-cov-2 delta variant infection, israel, 2021	Glatman-Freedman, A.; Hershkovitz, Y.; Kaufman, Z.; Dichtiar, R.; Keinan-Boker, L.; Bromberg, M.	2021	wrong outcomes
Limited Secondary Transmission of SARS-CoV-2 in Child Care Programs - Rhode Island, June 1-July 31, 2020	Link-Gelles, Ruth; DellaGrotta, Amanda L.; Molina, Caitlin; Clyne, Ailis; Campagna, Kristine; Lanzieri, Tatiana M.; Hast, Marisa A.; Palipudi, Krishna; Dirlikov, Emilio; Bandy, Utpala	2020	wrong outcomes
Etiology and clinical characteristics of SARS-CoV-2 and other human coronaviruses among children in Zhejiang Province, China 2017-2019	Zhang, Y.; Su, L.; Chen, Y.; Yu, S.; Zhang, D.; Mao, H.; Fang, L.	2021	wrong time period

Table S12: PRISMA checklist

Section and Topic	Item #	Checklist item	Location where item is reported
**TITLE**			
Title	1	Identify the report as a systematic review.	Page 1
**ABSTRACT**			
Abstract	2	See the PRISMA 2020 for Abstracts checklist.	Supplementary materials S34
**INTRODUCTION**			
Rationale	3	Describe the rationale for the review in the context of existing knowledge.	Page 3 (line 9 to 23)
Objectives	4	Provide an explicit statement of the objective(s) or question(s) the review addresses.	Page 3 (line 26 to 29)
**METHODS**			
Eligibility criteria	5	Specify the inclusion and exclusion criteria for the review and how studies were grouped for the syntheses.	Page 4 (line 27) to page 5 (line 8)
Information sources	6	Specify all databases, registers, websites, organisations, reference lists and other sources searched or consulted to identify studies. Specify the date when each source was last searched or consulted.	Page 4(line 14 to 19)
Search strategy	7	Present the full search strategies for all databases, registers and websites, including any filters and limits used.	Supplementary materials S2
Selection process	8	Specify the methods used to decide whether a study met the inclusion criteria of the review, including how many reviewers screened each record and each report retrieved, whether they worked independently, and if applicable, details of automation tools used in the process.	Page 4(line 23 to 25) and page 5 (line 8 to 13)
Data collection process	9	Specify the methods used to collect data from reports, including how many reviewers collected data from each report, whether they worked independently, any processes for obtaining or confirming data from study investigators, and if applicable, details of automation tools used in the process.	Page 5 (line 15 to 23)
Data items	10a	List and define all outcomes for which data were sought. Specify whether all results that were compatible with each outcome domain in each study were sought (e.g. for all measures, time points, analyses), and if not, the methods used to decide which results to collect.	Supplementary materials S3
	10b	List and define all other variables for which data were sought (e.g. participant and intervention characteristics, funding sources). Describe any assumptions made about any missing or unclear information.	Supplementary materials S3
Study risk of bias assessment	11	Specify the methods used to assess risk of bias in the included studies, including details of the tool(s) used, how many reviewers assessed each study and whether they worked independently, and if applicable, details of automation tools used in the process.	Page 5 (line 25) to page 6 (line 12)
Effect measures	12	Specify for each outcome the effect measure(s) (e.g. risk ratio, mean difference) used in the synthesis or presentation of results.	Page 6 (line 28 to 29), page 8 (line 20 to 21), page 9 (line 10 to 24), page 10 (line 11)
Synthesis methods	13a	Describe the processes used to decide which studies were eligible for each synthesis (e.g. tabulating the study intervention characteristics and comparing against the planned groups for each synthesis (item #5)).	Supplementary materials S35

Section and Topic	Item #	Checklist item	Location where item is reported
	13b	Describe any methods required to prepare the data for presentation or synthesis, such as handling of missing summary statistics, or data conversions.	Page 6 (line 16 to line 26)
	13c	Describe any methods used to tabulate or visually display results of individual studies and syntheses.	Page 6 (line 25 to line 26)
	13d	Describe any methods used to synthesize results and provide a rationale for the choice(s). If meta-analysis was performed, describe the model(s), method(s) to identify the presence and extent of statistical heterogeneity, and software package(s) used.	Page 6 (line 14 to line 26)
	13e	Describe any methods used to explore possible causes of heterogeneity among study results (e.g. subgroup analysis, meta-regression).	Page 6(line 23 to line 26)
	13f	Describe any sensitivity analyses conducted to assess robustness of the synthesized results.	Page 7 (line 4 to line 6)
Reporting bias assessment	14	Describe any methods used to assess risk of bias due to missing results in a synthesis (arising from reporting biases).	--
Certainty assessment	15	Describe any methods used to assess certainty (or confidence) in the body of evidence for an outcome.	Page 6 (line 21 to line 23)
**RESULTS**			
Study selection	16a	Describe the results of the search and selection process, from the number of records identified in the search to the number of studies included in the review, ideally using a flow diagram.	Figure 1
	16b	Cite studies that might appear to meet the inclusion criteria, but which were excluded, and explain why they were excluded.	Supplementary materials S32
Study characteristics	17	Cite each included study and present its characteristics.	Supplementary materials S30
Risk of bias in studies	18	Present assessments of risk of bias for each included study.	Supplementary materials S31
Results of individual studies	19	For all outcomes, present, for each study: (a) summary statistics for each group (where appropriate) and (b) an effect estimate and its precision (e.g. confidence/credible interval), ideally using structured tables or plots.	Page 10 (line 22) to page 15 (line 2); Supplementary materials S5 to S29
Results of syntheses	20a	For each synthesis, briefly summarise the characteristics and risk of bias among contributing studies.	Page 10 (line 28 to line 30)
	20b	Present results of all statistical syntheses conducted. If meta-analysis was done, present for each the summary estimate and its precision (e.g. confidence/credible interval) and measures of statistical heterogeneity. If comparing groups, describe the direction of the effect.	Page 11 (line
	20c	Present results of all investigations of possible causes of heterogeneity among study results.	Page 11 (line 15) to page 13 (line 28)
	20d	Present results of all sensitivity analyses conducted to assess the robustness of the synthesized results.	For primary analysis (Page 11- line 10 to line 13)
Reporting biases	21	Present assessments of risk of bias due to missing results (arising from reporting biases) for each synthesis assessed.	--
Certainty of evidence	22	Present assessments of certainty (or confidence) in the body of evidence for each outcome assessed.	Table 1 and table 2, supplementary materials S5 to S29

Section and Topic	Item #	Checklist item	Location where item is reported
**DISCUSSION**			
Discussion	23a	Provide a general interpretation of the results in the context of other evidence.	Page 15 (line 13) to Page 18 (line 27)
	23b	Discuss any limitations of the evidence included in the review.	Page 19 (line 2 to line 6), page 19 (line 8 to line 9)
	23c	Discuss any limitations of the review processes used.	Page 19 (line 6 to line 8), page 19 (line 10 to line 14)
	23d	Discuss implications of the results for practice, policy, and future research.	Page 20 (line 2 to line 9)
**OTHER INFORMATION**			
Registration and protocol	24a	Provide registration information for the review, including register name and registration number, or state that the review was not registered.	Page 4 (line 7 to line 9)
	24b	Indicate where the review protocol can be accessed, or state that a protocol was not prepared.	Page 4 (line 7 to line 9)
	24c	Describe and explain any amendments to information provided at registration or in the protocol.	Page 4 (line 25 to line 29)
Support	25	Describe sources of financial or non-financial support for the review, and the role of the funders or sponsors in the review.	Page 20 (line 15 to line 26)
Competing interests	26	Declare any competing interests of review authors.	Page 20 (line 27) to page 21 (line 2)
Availability of data, code and other materials	27	Report which of the following are publicly available and where they can be found: template data collection forms; data extracted from included studies; data used for all analyses; analytic code; any other materials used in the review.	Page 20 (line 13 to line 14)

From: Page MJ, McKenzie JE, Bossuyt PM, Boutron I, Hoffmann TC, Mulrow CD, et al. The PRISMA 2020 statement: an updated guideline for reporting systematic reviews. BMJ 2021;372:n71. doi: 10.1136/bmj.n71

For more information, visit: http://www.prisma-statement.org/

Table S13: PRISMA abstract checklist

Section and Topic	Item #	Checklist item	Reported (Yes/No)
**TITLE**			
Title	1	Identify the report as a systematic review.	Page 2 (line 4)
**BACKGROUND**			
Objectives	2	Provide an explicit statement of the main objective(s) or question(s) the review addresses.	Page 2 (line 5 to line 7)
**METHODS**			
Eligibility criteria	3	Specify the inclusion and exclusion criteria for the review.	Page 2 (line 10 to line 11)
Information sources	4	Specify the information sources (e.g. databases, registers) used to identify studies and the date when each was last searched.	Page 2 (line 8 to line 10)
Risk of bias	5	Specify the methods used to assess risk of bias in the included studies.	Page 2 (line 11 to line 12)
Synthesis of results	6	Specify the methods used to present and synthesise results.	Page 2 (line 12 to line 14)
**RESULTS**			
Included studies	7	Give the total number of included studies and participants and summarise relevant characteristics of studies.	Page 2 (line 15)
Synthesis of results	8	Present results for main outcomes, preferably indicating the number of included studies and participants for each. If meta-analysis was done, report the summary estimate and confidence/credible interval. If comparing groups, indicate the direction of the effect (i.e. which group is favoured).	Page 2 (line 16 to line 24)
**DISCUSSION**			
Limitations of evidence	9	Provide a brief summary of the limitations of the evidence included in the review (e.g. study risk of bias, inconsistency and imprecision).	Page 2 (line 17 to line 24) (confidence interval for certainty)
Interpretation	10	Provide a general interpretation of the results and important implications.	Page 2 (line 25 to line 28)
**OTHER**			
Funding	11	Specify the primary source of funding for the review.	Page 2 (line 31)
Registration	12	Provide the register name and registration number.	Page 2 (line 29 to line 30)

From: Page MJ, McKenzie JE, Bossuyt PM, Boutron I, Hoffmann TC, Mulrow CD, et al. The PRISMA 2020 statement: an updated guideline for reporting systematic reviews. BMJ 2021;372:n71. doi: 10.1136/bmj.n71

For more information, visit: http://www.prisma-statement.org/

Table S14: Outcomes reported by each study

Covidence number	Study	Proportion positive	Risk factor age	Risk factor sex	Risk factor ethnicity	Risk factor immunnosuppressed condition	Risk factor diabetes	Risk factor asthma	Risk factor cardiovascular disease	Risk factor epilepsy	Risk factor congenital cardiac disease	Risk factor neurological disease	Risk factor pregnancy	Severity-asymptomatic	Severity-mild	Severity-moderate	Severity-severe or critical	Severity-hospitalisation	Severity-ICU admission	Severity-O2 support	Severity-mechanical ventilation	Death	Total outcomes reported by study
#20080	Ã‡elik 2021													y					y			y	3
#13198	Abo 2021	y																					1
#13791	Ahmed 2021	y	y	y																			3
#12524	Aizawa 2021	y																					1
#13565	Akkoc 2021													y	y			y					3
#12118	Alattas 2021													y		y	y	y	y	y	y	y	8
#14215	AlGhamdi 2022													y	y		y		y		y	y	6
#18667	Alharbi 2021													y				y	y			y	4
#12167	Almuzaini 2021													y				y	y				3
#13654	Alonso 2021																	y		y	y	y	4
#14329	Alp 2021	y	y	y				y		y													5

**#18474**	**Alqayoudhi 2021**													y									1
**#12046**	**Alsharrah 2021**													y	y	y	y		y				5
**#14219**	**Alshengeti 2021**													y	y	y	y		y	y	y	y	8
**#17026**	**Alshukairi 2021**	y																					1
**#18847**	**AnaLaura 2021**	y																					1
**#12731**	**AntÃºnez-Montes 2021**													y				y	y			y	4
**#12143**	**Anugulruengkitt 2021**													y	y	y	y			y	y		6
**#17010**	**Aprea 2021**	y												y									2
**#20057**	**Arellano-Llamas 2020**					y	y	y	y				y						y			y	7
**#12209**	**Armocida 2022**	y	y	y																			3
**#12043**	**Arslan 2021**	y	y	y										y	y	y	y		y	y	y	y	11
**#11993**	**Asseri 2021**	y																					1
**#20144**	**Ayed 2020**	y																					1
**#14146**	**Bai 2021**													y	y		y				y	y	5

**#10 899**	**Bandi 2020**	y	y		y																		3
**#18 317**	**Barrera 2021**													y				y				y	3
**#15 768**	**Baumgarte 2022**	y																					1
**#13 036**	**Bayesheva 2021**														y	y	y		y		y	y	6
**#12 845**	**Bellino 2020**													y	y		y	y	y			y	6
**#11 967**	**Berksoy 2021**	y																					1
**#12 938**	**Besli 2021**													y	y	y	y	y	y	y	y	y	9
**#15 039**	**Biko 2021**	y												y				y	y	y	y		6
**#11 943**	**Bolanos-Almeida 2021**													y	y			y	y			y	5
**#17 624**	**Brotons 2021**	y																					1
**#17 729**	**Buonsenso 2021**													y				y	y				3
**#12 204**	**Buta 2022**														y	y	y			y		y	5
**#18 120**	**Calvani 2021**	y	y	y																			3
**#17 455**	**Capozza 2021**	y																					1
**#14 391**	**Capra 2021**	y															y	y				y	4

**#18681**	**Carrasco 2021**	y																					1
**#12127**	**Cheng 2022**	y	y															y					3
**#15615**	**Chiang 2021**													y	y	y	y	y		y	y	y	8
**#10777**	**Chopra 2021**																					y	1
**#17381**	**Chowdhoury 2021**	y																					1
	**Chua 2020**													y								y	2
**#12010**	**Chua 2021**													y	y				y	y			4
**#14451**	**CiofiDegliAtti 2020**	y																					1
**#17094**	**Cloete 2022**																		y	y	y	y	4
**#12132**	**Cofre 2020**													y					y			y	3
**#11196**	**Cohen 2021**	y	y	y	y																		4
**#11841**	**Colson 2020**	y																					1
**#19451**	**Colson 2020**	y																					1
**#18590**	**Cooper 2021**	y																					1
**#18622**	**Corso 2021**													y	y		y	y	y	y	y	y	8
**#12906**	**Dash 2021**	y																				y	2

#15 547	Dawood 2022	y																					1
#14 905	Delahoy 2021																		y	y	y	y	4
#18 417	deLusignan 2020	y																					1
#12 361	Devrim 2022																		y				1
#16 765	Dilber 2021	y																y				y	3
#11 258	Dominguez -Rodriguez 2021														y				y			y	3
#13 432	Dominguez Rojas 2021																		y				1
#14 191	Dong 2020	y												y	y	y	y						5
	Du 2021													y								y	2
#12 015	Du 2021													y	y		y			y	y	y	6
#18 117	Eleftheriou 2021	y																					1
#12 898	Elghoudi 2020													y				y		y	y	y	5
#19 860	Elif 2021		y	y																			2
#19 823	Engels 2022	y																					1
#12 021	Ennab 2021													y	y	y	y		y			y	6

**#15528**	**Ergenc 2021**																	y	y			y	3
**#16095**	**Erturk 2021**	y																					1
**#14144**	**Ferraro 2021**													y	y	y	y	y	y			y	7
**#14530**	**Forster 2022**	y																					1
**#19329**	**Foster 2021**	y																					1
**#17042**	**Funk 2022**	y												y				y	y			y	5
**#14176**	**Gaborieau 2020**	y																	y	y	y	y	5
**#19930**	**Galli 2021**	y	y																				2
**#12844**	**Gampel 2020**	y																					1
**#16369**	**Garazzino 2020**													y				y	y	y	y		5
**#11758**	**Gavriliu 2021**	y												y	y	y		y	y				6
**#12220**	**Ghosh 2020**	y																					1
**#14321**	**Goktug 2021**	y												y	y	y	y	y	y		y	y	9
**#18315**	**Gomes 2021**																		y		y	y	3
**#12888**	**Gotzinger 2020**													y					y	y	y	y	5

**#19 181**	**Gudbjartsson 2020**	y																					1
**#11 470**	**Gujar 2021**													y	y	y	y					y	5
**#12 032**	**Gumus 2021**													y					y			y	3
**#10 914**	**Guzman 2021**																y	y		y	y	y	5
	**Haeusler 2021**													y	y	y	y	y	y		y	y	8
**#12 147**	**Hedberg 2022**																		y			y	2
**#18 426**	**Hendler 2021**	y																	y	y	y	y	5
**#12 292**	**Hernandez-Garduno 2020**	y	y	y		y	y	y	y				y					y	y		y	y	12
**#17 005**	**Hijazi 2021**													y				y	y		y	y	5
**#14 625**	**Hobbs 2022**																y		y	y	y	y	5
**#14 562**	**Howard 2021**																	y	y		y	y	4
**#17 773**	**Huete-Perez 2021**	y		y										y									3
**#18 738**	**Ibrahim 2020**	y		y			y																3
**#11 754**	**Ibrahim 2021**					y		y						y				y	y				5
**#18 509**	**Imamura 2021**	y																					1

**#11 703**	**Indriyani 2021**													y	y	y	y	y	y			y	7
**#12 383**	**Isoldi 2021**	y																					1
**#14 149**	**Jang 2022**													y			y	y				y	4
**#11 737**	**Ji 2021**													y	y	y	y						4
	**Jiang 2022**													y	y	y	y					y	5
**#17 583**	**Jimenez-Garcia 2021**	y																					1
**#14 392**	**Kaba 2021**	y																					1
**#11 962**	**Kaliyan 2021**													y						y	y	y	4
**#13 359**	**Kanthimathinathan 2020**	y																					1
**#15 108**	**Kapoor 2021**	y														y	y				y	y	5
**#14 331**	**Kara 2021**														y	y	y		y		y	y	6
**#12 245**	**Karaaslan 2021**	y																					1
**#19 023**	**Karaci 2021**	y	y	y										y	y	y	y	y	y			y	10
**#14 170**	**Karbuz 2021**													y	y	y	y		y		y	y	7
**#18 565**	**Kavanagh 2021**	y																					1

**#12 840**	**Kepenekli 2022**																	y	y	y	y	y	5
**#14 904**	**Kim 2020**																		y	y	y	y	4
**#16 921**	**Krithika 2021**													y	y	y	y					y	5
**#11 883**	**Kuchar 2021**	y																					1
**#17 757**	**Kuczborska 2021**	y				y																	2
**#14 210**	**Kufa 2022**	y																	y	y	y	y	5
**#17 040**	**Kumar 2021**	y		y										y						y	y	y	6
**#14 601**	**Kushner 2021**													y		y	y		y	y		y	6
**#18 693**	**Ladhani 2021**	y																					1
**#14 522**	**Lanari 2021**	y																					1
**#11 715**	**Lazzerini 2021**	y	y	y		y	y	y	y			y		y	y	y	y	y	y	y	y	y	17
**#10 983**	**Lee 2022**																y						1
**#13 257**	**Leidman 2021**																	y	y			y	3
**#11 659**	**Levy 2020**	y																					1
**#14 462**	**Lindsay 2021**	y																					1

**#13 612**	**Liu 2020**	y																					1
**#18 392**	**Liu 2020**		y																				1
**#18 661**	**Loconsole 2020**													y	y	y	y	y	y			y	7
**#11 887**	**Lopez-Aguilar 2020**	y																					1
**#13 694**	**Lorenzo 2021**	y	y	y	y		y			y	y	y											8
**#18 653**	**Lu 2020**	y												y					y		y	y	5
**#19 361**	**Lu 2020**													y						y			2
**#16 059**	**Lynam 2021**	y																					1
**#18 165**	**Madani 2021**													y					y			y	3
**#11 847**	**Maltezou 2020**													y	y	y	y	y	y			y	7
**#19 960**	**Mania 2021**	y																y				y	3
**#17 584**	**Mania 2022**																	y	y	y	y	y	5
**#12 041**	**Matteudi 2021**	y												y				y				y	4
**#16 052**	**Mele 2021**	y	y	y	y				y			y											6
**#18 300**	**Messiah 2021**																	y	y				2

#13 238	Meyer 2021	y	y	y																			3
#16 311	Meyer 2022	y		y																			2
#18 704	Michos 2021	y	y																				2
#11 954	Morban 2021													y				y	y			y	4
#18 395	Moreno-Noguez 2021																		y				1
#17 716	Murillo-Zamora 2020	y	y	y		y										y	y						6
#17 759	Musa 2021													y								y	2
#13 825	MveangNzoghe 2021	y																					1
#13 292	Navarro-Olivos 2021	y																				y	2
#11 947	Ng 2021													y					y	y		y	4
#14 784	Nunziata 2020	y																y		y	y		4
#19 294	Odeleye 2021	y																					1
	Okonkwo 2020	y																					1
#12 113	Okur 2021																		y	y	y	y	4

**#12169**	**Olivar-Lopez 2020**	y	y	y							y	y											5
**#12008**	**Oliveira 2021**																					y	1
**#17910**	**Ollier 2022**	y																					1
**#13982**	**Olson 2022**																y		y		y	y	4
**#14571**	**Omrani 2020**																	y				y	2
**#11835**	**Ortiz-Pinto 2022**	y												y	y			y	y				5
**#18407**	**Osmanov 2022**																y						1
	**Otto 2021**	y			y																		2
**#14212**	**Otto 2020**	y												y									2
**#11364**	**Ozlu 2022**													y	y	y	y					y	5
**#11716**	**Paduano 2021**	y	y	y																			3
**#12162**	**Pande 2021**													y	y	y	y		y	y	y	y	8
**#15539**	**Pandey 2020**	y																					1
**#12915**	**Parambil 2021**																			y	y	y	3
**#18316**	**Parcha 2021**																	y	y				2
**#12864**	**Parri 2020**													y	y	y	y	y		y	y	y	8

#18904	Peaper 2021	y																					1
#17051	Peng 2021													y									1
#18798	Perramon 2021	y																					1
#11945	Pokorska-Spiewak 2021													y				y	y	y	y	y	6
#12338	Pokorska-Spiewak 2021	y																					1
#17273	Pudjiadi 2021																					y	1
#18170	Qian 2021														y	y	y					y	4
#11176	Rabha 2021													y	y	y	y	y	y	y		y	8
#12124	Rabha 2021																y	y	y	y	y	y	6
#13983	Reis 2021													y			y	y				y	4
#18898	Rha 2020	y																					1
#14463	Rivas-Ruiz 2020	y																	y		y	y	4
#18779	Rizzo 2021	y																					1
#19524	RodriguezVelasquez 2021																					y	1

**#18 260**	**Rose 2021**	y																					1
**#12 348**	**Sahni 2021**	y																					1
**#17 768**	**Salako 2021**	y																					1
**#18 966**	**Saleh 2021**																y		y	y	y	y	5
**#17 604**	**Sananez 2021**													y	y	y	y		y	y	y	y	8
**#15 025**	**Schneider 2021**	y	y	y	y	y	y	y		y	y	y											10
**#16 363**	**Sedighi 2021**																y		y	y		y	4
**#11 948**	**See 2021**													y	y	y	y		y	y		y	7
**#11 996**	**Sena 2021**													y				y				y	3
**#12 084**	**Shahid 2021**													y	y	y	y	y		y	y	y	8
**#12 894**	**ShapiroBen David 2021**													y				y	y	y	y	y	6
**#14 140**	**Sharma 2020**													y	y	y	y	y		y	y	y	8
**#17 720**	**Sharma 2021**														y	y	y	y		y	y	y	7
	**Shayganmehr 2021**	y																					1
	**Shi 2022**							y										y					2
**#12 033**	**Shoji 2021**													y					y	y	y	y	5

#12 357	**Shoji 2022**													y					y	y	y	y	5
#12 163	**Singh 2022**	y													y	y	y	y		y	y	y	8
#17 820	**Sola 2021**	y																					1
#11 753	**Somekh 2021**																	y					1
#12 277	**Song 2021**	y																	y			y	3
#14 919	**Soriano-Arandes 2021**	y												y				y				y	4
#16 835	**Sousa 2021**																					y	1
#12 523	**Stokes 2020**																	y	y			y	3
#14 125	**Talarico 2021**	y												y				y	y				4
#12 080	**Tang 2021**														y	y	y		y			y	5
#11 710	**Tosif 2021**	y												y				y	y			y	5
#14 465	**Uka 2021**													y					y	y	y	y	5
#12 974	**Ustundag 2021**													y	y	y	y	y					5
#12 076	**vanderZalm 2021**	y																y	y			y	4
#13 788	**Verd 2021**	y																					1

**#14 883**	**Vergine 2020**	y																					1
**#18 725**	**Vogel 2022**	y																					1
**#18 807**	**Wang 2020**	y																					1
**#11 261**	**Wang 2021**																		y				1
**#11 691**	**Wanga 2021**																		y	y	y	y	4
**#18 408**	**Ward 2022**																		y			y	2
**#12 998**	**Weclawek-Tompol 2021**																			y	y	y	3
**#17 266**	**Wong 2022**	y							y			y		y	y	y	y		y	y	y	y	11
**#11 997**	**Wong-Chew 2021**																		y			y	2
**#12 319**	**Xiong 2020**													y						y	y	y	4
	**Yayla 2020**	y																					1
**#14 344**	**Yilmaz 2020**													y	y	y	y		y	y		y	7
**#13 790**	**Yilmaz 2021**																		y	y	y	y	4
**#14 312**	**YilmazCelebi 2022**																	y	y				2
**#17 403**	**Yonker 2020**	y																					1

**#11820**	**Yoon 2021**	y																					1
		117	22	21	6	7	6	7	5	3	3	6	2	87	50	44	59	67	93	57	63	119	

Text S1: Selection criteria for inclusion of studies in the review

Inclusion criteria

Population:

- Humans aged ≤18 years old (can include any subgroups of population within this age-group. For e.g., <5 years old, 2-8 years old, infants etc.) OR
- Studies reporting data on children and adult population if age-stratified data for the ≤18 years old are available

Exposure:

- SARS-CoV-2 infection confirmed by PCR or rapid antigen tests

Outcomes: Studies reporting at least one of the following outcomes

- Proportion positive
- Incidence
- Severity of COVID-19 disease
- COVID-19 disease leading to death
- Risk factors of COVID-19 (Univariate or multivariate analysis with ORs or IRs for testing positive for SARS-CoV-2 with any risk factor/s)
- Differential impacts of different variants of SARS-CoV-2

Settings:

- Community OR
- Outpatient OR
- Inpatient or hospital OR
- Schools and day care centres OR
- Children seeking therapy at health centres for other conditions. For e.g., children under treatment for cancer at a centre providing cancer care

Type of study:

- Observational studies (cross-sectional studies, case-control studies, prospective and retrospective cohort studies) AND
- Studies must enrol an eligible sample of at least 100 children

Exclusion criteria

Population:

- Data for the ≤18 years age-group unavailable

Exposure:

- Clinical suspicion of COVID-19 disease OR
- SARS-CoV-2 infection diagnosed by serological tests

Outcomes:

- Health problems (for example, anxiety, depression, obesity) occurring not as a direct consequence of the COVID-19 disease in children but generally as a result of non-pharmaceutical interventions and effect of the pandemic on the health delivery for other health problems (for example, cancer care or asthma care)

Settings:

There will not be any restrictions on study settings

Type of study:

- Recommendations OR
- Consensus papers OR
- Study protocols OR
- Narrative reviews, systematic reviews, and meta-analyses OR
- Commentaries OR
- Modelling studies without primary data OR
- Studies enrolling an eligible sample of less than 100 children OR
- Pre-prints OR
- Conference or meeting abstracts

Text S2: Search strategies

Searches Run: 27/02/2022

Cinahl (1,021)

S20	limiters - Published Date: 20191201-
S19	S3 AND S6 AND S18
S18	S7 OR S8 OR S9 OR S10 OR S11 OR S12 OR S13 OR S14 OR S15 OR S16 OR S17
S17	"case fatality"
S16	(MH "Mortality") OR (MH "Child Mortality") OR (MH "Hospital Mortality") OR (MH "Infant Mortality")
S15	MH "Systemic Inflammatory Response Syndrome”
S14	(MIS or PIMS or "MIS-C" or "multisystem* inflammatory syndrome")
S13	TI (asthma or "chronic lung disease" or "congenital heart disease" or cardiac or cancer) OR AB (asthma or "chronic lung disease" or "congenital heart disease" or cardiac or cancer)) AND (TI ((risk* or impact or rate* or involvement or role)) OR AB ((risk* or impact or rate* or involvement or role)))) OR (TI ("congenital heart disease" or cardiac or cancer) OR AB (asthma or "chronic lung disease" or "congenital heart disease" or cardiac or cancer)) AND (TI ((risk* or impact or rate* or involvement or role)) OR AB ((risk* or impact or rate* or involvement or role)))) OR (TI ((immunosuppress* or immunocompromis*) or ("underlying medical condition*" or "underlying health status")) OR AB ((immunosuppress* or immunocompromis*) or ("underlying medical condition*" or "underlying health status"))
S12	(MH risk factors AND (MW epidemiology or MW mortality)) OR (MH age factors AND MW epidemiology) OR MH comorbidity
S11	TI "oxygen support" or AB "oxygen support"
S10	(MH "Positive Pressure Ventilation+") OR (MH Ventilation+") OR (MH Respiration, Artificial")
S9	TI (hospitaliz* or hospitalis*) OR AB (hospitaliz* or hospitalis*)) AND (TI (mild or moderate or severe or symptoms or complication*) OR AB (mild or moderate or severe or symptoms or complication*))) OR (TI "hospitali#ation rate*" OR AB "hospitali#ation rate*"
S8	(MH "Hospitalization") OR (MH "Length of Stay") OR (MH "Patient Admission") OR (MH "Readmission")
S7	(MH "Incidence)
S6	S4 OR S5
S5	(MH "Pediatrics")
S4	TI ((p#ediatric* or children or adolescent*)) OR AB ((p#ediatric* or children or adolescent*)
S3	S1 OR S2
S2	TI (("COVID-19*" OR Coronavirus disease OR 2019*" or "Coronavirus 2019" or "COVID-2019*" or "SARS-CoV-2" or "SARS CoV 2" or

	"SARSCoV-2019")) OR AB (("COVID-19*" OR Coronavirus disease OR 2019*" or "Coronavirus 2019" or "COVID-2019*" or "SARS-CoV-2" or "SARS CoV 2" or "SARSCoV-2019"))
S1	(MH "COVID-19") OR (MH "SARS-CoV-2")

Embase (6048)

Embase <1974 to 2022 February 27>

#	**Query**
1	Coronavirus Disease 2019/ or exp Severe acute respiratory syndrome coronavirus 2/
2	(COVID-19* or Coronavirus disease 2019* or Coronavirus 2019 or COVID-2019* or SARS-CoV-2 or SARS CoV 2 or SARS-CoV-2019).ti,ab.
3	1 or 2
4	(p?ediatric* or children or adolescent*).ti,ab.
5	pediatrics/
6	4 or 5
7	incidence/
8	hospitalization/ or hospital admission/ or "length of stay"/
9	(((hospitaliz* or hospitalis*) and (mild or moderate or severe or symptoms or complication*)) or hospitali?ation rate*).ti,ab.
10	artificial ventilation/ or invasive ventilation/ or noninvasive ventilation/ or positive pressure ventilation/
11	oxygen support.ti,ab.
12	(risk factor/ and (epidemiology or mortality).fs.) or (age distribution/ and epidemiology.fs.) or comorbidity/
13	(((asthma or chronic lung disease or congenital heart disease or cardiac or cancer) and (risk* or impact or rate* or involvement or role)) or (immunosuppress* or immunocompromis*) or underlying medical condition* or underlying health status).ti,ab.
14	(MIS or PIMS or MIS-C or multisystem* inflammatory syndrome).mp.
15	systemic inflammatory response syndrome/ or hyperinflammatory syndrome/ or pediatric multisystem inflammatory syndrome/
16	mortality/ or childhood mortality/ or exp hospital mortality/ or infant mortality/ or infection fatality ratio/ or exp mortality rate/
17	case fatality.mp.

18	7 or 8 or 9 or 10 or 11 or 12 or 13 or 14 or 15 or 16 or 17
19	3 and 6 and 18
20	("201948" or "201949" or "20195*" or 2020* or 2021* or 2022*).em.
21	19 and 20

Global Health (1460)
Global Health <1910 to 2022 Week 08>

#	**Query**
1	Betacoronavirus/ or Human Coronaviruses/ or exp severe acute respiratory syndrome-related coronavirus/
2	(COVID-19* or Coronavirus disease 2019* or Coronavirus 2019 or COVID-2019* or SARS-CoV-2 or SARS CoV 2 or SARS-CoV-2019).ti,ab.
3	1 or 2
4	(p?ediatric* or children or adolescent*).ti,ab.
5	paediatrics/
6	4 or 5
7	exp incidence/
8	exp hospital admission/ or hospital stay/
9	(((hospitaliz* or hospitalis*) and (mild or moderate or severe or symptoms or complication*)) or hospitali?ation rate*).ti,ab.
10	artificial respiration/
11	oxygen support.ti,ab.
12	(risk factor/ and (epidemiology or mortality).hw.) or (age distribution/ and epidemiology.hw.) or comorbidity/
13	(((asthma or chronic lung disease or congenital heart disease or cardiac or cancer) and (risk* or impact or rate* or involvement or role)) or (immunosuppress* or immunocompromis*) or underlying medical condition* or underlying health status).ti,ab.
14	systemic inflammatory response syndrome/
15	(MIS or PIMS or MIS-C or multisystem* inflammatory syndrome).mp.

16	mortality/ or infant mortality/
17	(child mortality or case fatality).mp.
18	7 or 8 or 9 or 10 or 11 or 12 or 13 or 14 or 15 or 16 or 17
19	3 and 6 and 18
20	("2019" or "2020" or "2021" or "2022").dp.
21	19 and 20

Medline (3487)
Ovid MEDLINE(R) and Epub Ahead of Print, In-Process, In-Data-Review & Other Non-Indexed Citations, Daily and Versions(R) <1946 to February 27, 2022>

#	**Query**
1	COVID-19/ or SARS-CoV-2/
2	(COVID-19* or Coronavirus disease 2019* or Coronavirus 2019 or COVID-2019* or SARS-CoV-2 or SARS CoV 2 or SARS-CoV-2019).ti,ab.
3	1 or 2
4	(p?ediatri* or children or adolescent*).ti,ab.
5	Pediatrics/
6	4 or 5
7	Incidence/
8	hospitalization/ or "length of stay"/ or patient admission/
9	(((hospitaliz* or hospitalis*) and (mild or moderate or severe or symptoms or complication*)) or hospitali?ation rate*).ti,ab.
10	respiration, artificial/ or noninvasive ventilation/ or exp positive-pressure respiration/
11	oxygen support.ti,ab.
12	(risk factors/ and (epidemiology or mortality).fs.) or (age factors/ and epidemiology.fs.) or exp comorbidity/
13	(((asthma or chronic lung disease or congenital heart disease or cardiac or cancer) and (risk* or impact or rate* or involvement or role)) or (immunosuppress* or immunocompromis*) or (underlying medical condition* or underlying health status)).ti,ab.

14	(MIS or PIMS or MIS-C or multisystem* inflammatory syndrome).mp.
15	systemic inflammatory response syndrome/ or cytokine release syndrome/
16	mortality/ or child mortality/ or hospital mortality/ or infant mortality/
17	case fatality.mp.
18	7 or 8 or 9 or 10 or 11 or 12 or 13 or 14 or 15 or 16 or 17
19	3 and 6 and 18
20	limit 19 to dt="20191201-20220227"

WHO Covid (7034)

(((ti:(pediatri* OR paediatri* OR children OR adolescent*)) OR (ab:(pediatri* OR paediatri* OR children OR adolescent*)))) AND (((ti:(incidence)) OR (ab:(incidence))) OR (((ti:(hospitaliz* OR hospitalis*)) OR (ab:(hospitaliz* OR hospitalis*))) AND ((ti:((mild OR moderate OR severe OR symptoms OR complication*))) OR (ab:((mild OR moderate OR severe OR symptoms OR complication*))))) OR (((ti:("hospitalization rate" OR "hospitalization rates" OR "hospitalisation rate" OR "hospitalisation rates")) OR (ab:("hospitalization rate" OR "hospitalization rates" OR "hospitalisation rate" OR "hospitalisation rates")))) OR (((ti:("artificial respiration" OR "invasive ventilation" OR "noninvasive ventilation" OR "non-invasive ventilation" OR "positive pressure respiration" OR "oxygen support" OR incidence)) OR (ab:("artificial respiration" OR "invasive ventilation" OR "noninvasive ventilation" OR "non-invasive ventilation" OR "positive pressure respiration" OR "oxygen support")))) OR (((ti:("risk factor" OR "risk factors" OR asthma OR "chronic lung disease" OR "congenital heart disease" OR cardiac OR cancer)) OR (ab:("risk factor" OR "risk factors" OR asthma OR "chronic lung disease" OR "congenital heart disease" OR cardiac OR cancer))) AND ((ti:((risk OR risks OR impact OR rate OR rates OR involvement OR role))) OR (ab:((risk OR risks OR impact OR rate OR rates OR involvement OR role))))) OR (((ti:((immunosuppress* OR immunocompromis* OR "underlying medical condition" OR "underlying medical conditions" OR "underlying health status" OR comorbid*))) OR (ab:((immunosuppress* OR immunocompromis* OR "underlying medical condition" OR "underlying medical conditions" OR "underlying health status" OR comorbid*))))) OR (((ti:(mis OR pims OR "MIS-C" OR "multisystem* inflammatory syndrome" OR "multisystemic inflammatory syndrome" OR "systemic inflammatory response syndrome")) OR (ab:(mis OR pims OR "MIS-C" OR "multisystem* inflammatory syndrome" OR "multisystemic inflammatory syndrome" OR "systemic inflammatory response syndrome")))) OR (((ti:(mortality OR "case fatality")) OR (ab:(mortality OR "case fatality"))))

CNKI
(SU=儿童+小儿+幼儿+婴儿+学龄+少年+青年+青少年+学龄前+幼儿园+小学+初中+高中) AND (SU=新型冠状病毒+新冠+新型冠状病毒肺炎+COVID-19+SARS-CoV-2)
学术期刊与论文
~ to 2022-03-21
1365 records

Wanfang
(主题:(新型冠状病毒) or 主题:(新冠) or 主题:(新型冠状病毒肺炎) or 主题:(COVID-19) or 主题:(SARS-CoV-2))， 中英文扩展、主题词扩展 AND

(主题:(儿童) or 主题:(小儿) or 主题:(幼儿) or 主题:(婴儿) or 主题:(学龄) or 主题:(少年) or 主题:(青年) or 主题:(青少年) or 主题:(学龄前) or 主题:(幼儿园) or 主题:(小学) or 主题:(初中) or 主题:(高中))， 中英文扩展、主题词扩展
学科分类：医药、卫生

~ to 2022-03-21
2539 records

CQvip
(题名或关键词=儿童 OR 题名或关键词=幼儿 OR 题名或关键词=小儿OR 题名或关键词=未成年人) AND (题名或关键词=新型冠状病毒 OR 题名或关键词=新冠 OR (题名或关键词=2019-nCoV)
学科分类：医药、卫生
~ to 2020-03-21
888 records

Text S3: List of variables for which data were extracted

Study characteristics

1. Author name: last name of first author
2. Published year: year of publication of the study
3. Covidence number: Unique study code assigned by Covidence
4. Study ID: Unique study code assigned by DK
5. Data collection start month: month in which data collection was started
6. Data collection start year: year in which data collection was started
7. Data collection end month: month in which data collection ceased
8. Data collection end year: year in which data collection ceased
9. Country: name of country/ countries where the study was conducted

Test positivity

1. Author name: last name of first author
2. Published year: year of publication of the study
3. Covidence number: Unique study code assigned by Covidence
4. Study ID: Unique study code assigned by DK
5. Samples: Total number of specimens tested
6. Cases: total number of positive tests
7. Age group 1: age group 1 as defined by the study authors
8. Samples in age group 1: total number of specimens tested in age group 1
9. Cases in age group 1: total number of positive tests in age group 1
10. Age group 2: age group 1 as defined by the study authors
11. Samples in age group 2: total number of specimens tested in age group 2
12. Cases in age group 2: total number of positive tests in age group 2
13. Age group 3: age group 1 as defined by the study authors
14. Samples in age group 3: total number of specimens tested in age group 3
15. Cases in age group 3: total number of positive tests in age group 3
16. Age group 4: age group 1 as defined by the study authors
17. Samples in age group 4: total number of specimens tested in age group 4
18. Cases in age group 4: total number of positive tests in age group 4
19. Age group 5: age group 1 as defined by the study authors
20. Samples in age group 5: total number of specimens tested in age group 5
21. Cases in age group 5: total number of positive tests in age group 5
22. WHO region: WHO region according to country
23. Country income level: Country income level classification according to the World Bank
24. Dominant variant: Dominant variant in the country at the mid-study time
25. Study setting: if study was conducted in community, health facility, educational institution or other setting.
26. Testing method: PCR, antigen test or both

Risk factors

1. Author name: last name of first author
2. Published year: year of publication of the study
3. Covidence number: Unique study code assigned by Covidence
4. Study ID: Unique study code assigned by DK
5. Risk factor: age, sex, race/ethnicity/ comorbidity/ pregnancy
6. Risk factor group: name of the group (for example: 0 to 5 years for age, male for sex, Caucasian for race/ethnicity, neurological disease for comorbidity)
7. Total sample size for unadjusted analysis: sample size for unadjusted analysis
8. Exposed and positive: number of individuals with risk factor present and positive SARS-CoV-2 test
9. Exposed and negative: number of individuals with risk factor present and negative SARS-CoV-2 test
10. Unexposed and positive: number of individuals with risk factor absent and positive SARS-CoV-2 test

11. Unexposed and negative: number of individuals with risk factor absent and negative SARS-CoV-2 test
12. Unadjusted RR: unadjusted risk ratio
13. Unadjusted RR lower 95% CI: lower 95% CI for the unadjusted risk ratio estimate
14. Unadjusted RR upper 95% CI: upper 95% CI for the unadjusted risk ratio estimate
15. Adjusted RR: adjusted risk ratio
16. Adjusted RR lower 95% CI: lower 95% CI for the adjusted risk ratio estimate
17. Adjusted RR upper 95% CI: upper 95% CI for the adjusted risk ratio estimate
18. Factors adjusted for: factors adjusted for in the adjusted analysis
19. Total sample size for comparison between continuous variables: sample size for continuous variable analysis
20. Summary estimate: mean (S.D.) or median (IQR) or median (range)
21. Values in SARS-CoV-2 positive group: mean (S.D.) or median (IQR) or median (range) in SARS-CoV-2 positive group
22. Values in SARS-CoV-2 negative group: mean (S.D.) or median (IQR) or median (range) in SARS-CoV-2 negative group
23. p-value: p-value for the difference in SARS-CoV-2 positive and negative groups

Severity:

1. Author name: last name of first author
2. Published year: year of publication of the study
3. Covidence number: Unique study code assigned by Covidence
4. Study ID: Unique study code assigned by DK
5. Cases: number of cases followed-up
6. Asymptomatic: number of cases with asymptomatic disease
7. Mild: number of cases with mild disease
8. Moderate: number of cases with moderate disease
9. Severe/critical: number of cases with severe or critical disease
10. Hospitalisation: number of cases requiring hospitalisation
11. ICU admission: number of cases requiring intensive care unit or high dependency unit admission
12. Oxygen support: number of cases requiring oxygen supplementation
13. Assisted ventilation: number of cases requiring assisted ventilation

Mortality:

1. Author name: last name of first author
2. Published year: year of publication of the study
3. Covidence number: Unique study code assigned by Covidence
4. Study ID: Unique study code assigned by DK
5. Cases: number of cases followed-up
6. Death: Cases: number of cases with fatal outcomes

Text S4: Checklists used for quality appraisal of included studies (according to study design)

JBI critical appraisal checklist for analytical cross-sectional studies

1. Were the criteria for inclusion in the sample clearly defined?
2. Were the study subjects and the setting described in detail?
3. Was the exposure measured in a valid and reliable way?
4. Were objective, standard criteria used for measurement of the condition?
5. Were confounding factors identified?
6. Were strategies to deal with confounding factors stated?
7. Were the outcomes measured in a valid and reliable way?
8. Was appropriate statistical analysis used?

JBI critical appraisal checklist for case-control studies

1. Were the groups comparable other than the presence of disease in cases or the absence of disease in controls?
2. Were cases and controls matched appropriately?
3. Were the same criteria used for identification of cases and controls?
4. Was exposure measured in a standard, valid and reliable way?
5. Was exposure measured in the same way for cases and controls?
6. Were confounding factors identified?
7. Were strategies to deal with confounding factors stated?
8. Were outcomes assessed in a standard, valid and reliable way for cases and controls?
9. Was the exposure period of interest long enough to be meaningful?
10. Was appropriate statistical analysis used?

JBI critical appraisal checklist for cohort studies

1. Were the criteria for testing for SARS-CoV-2 clear and consistent across the study groups?
2. Were the exposures measured similarly to assign people to both exposed and unexposed groups?
3. Was the exposure measured in a valid and reliable way?
4. Were confounding factors identified?
5. Were strategies to deal with confounding factors stated?
6. Were the groups/participants free of the outcome at the start of the study (or at the moment of exposure)?
7. Were the outcomes measured in a valid and reliable way?
8. Was the follow up time reported and sufficient to be long enough for outcomes to occur?

9. Was follow up complete, and if not, were the reasons to loss to follow up described and explored?

10. Were strategies to address incomplete follow up utilized?

11. Was appropriate statistical analysis used?

JBI critical appraisal checklist for quasi-experimental studies

1. Is it clear in the study what is the ‘cause’ and what is the ‘effect’ (i.e. there is no confusion about which variable comes first)?

2. Were the participants included in any comparisons similar?

3. Were the participants included in any comparisons receiving similar treatment/care, other than the exposure or intervention of interest?

4. Was there a control group?

5. Were there multiple measurements of the outcome both pre and post the intervention/exposure?

6. Was follow up complete and if not, were differences between groups in terms of their follow up adequately described and analyzed?

7. Were the outcomes of participants included in any comparisons measured in the same way?

8. Were outcomes measured in a reliable way?

9. Was appropriate statistical analysis used?

JBI critical appraisal checklist for diagnostic test accuracy studies

1. Was a consecutive or random sample of patients enrolled?

2. Was a case control design avoided?

3. Did the study avoid inappropriate exclusions?

4. Were the index test results interpreted without knowledge of the results of the reference standard?

5. If a threshold was used, was it pre-specified?

6. Is the reference standard likely to correctly classify the target condition?

7. Were the reference standard results interpreted without knowledge of the results of the index test?

8. Was there an appropriate interval between index test and reference standard?

9. Did all patients receive the same reference standard?

10. Were all patients included in the analysis?

Scoring of studies: Studies were deemed to be of good quality if they had a score between 7 and 8 (cross-sectional studies), 8 and 10 (case control studies and quasi experimental studies), 9 and 11 (cohort studies), or 8 and 10 (diagnostic test accuracy studies). Studies were considered to be of fair quality if they had a score between 4 and 6 (cross-sectional studies), 4 and 7 (case control studies and quasi experimental studies), 6 and 8 (cohort studies), or 4 and 7 (diagnostic test accuracy studies). Poor quality studies had a score between 0 and 3 (cross-sectional, case control, quasi experimental studies, and diagnostic test accuracy studies) or 0 and 5 (cohort studies).

Figure S1: Forest plot: Pooled proportion of positive SARS-CoV-2 tests in people aged ≤18 years

Study	Events	Total	Proportion	95%-CI	Weight (common)	Weight (random)
Carrasco 2021	0	101	0.00	[0.00; 0.04]	0.0%	0.8%
Erturk 2021	4	101	0.04	[0.01; 0.10]	0.0%	0.8%
Rose 2021	1	101	0.01	[0.00; 0.05]	0.0%	0.8%
Jimenez-Garcia 2021	19	107	0.18	[0.11; 0.26]	0.0%	0.8%
Mele 2021	7	110	0.06	[0.03; 0.13]	0.0%	0.8%
Alshukairi 2021	9	119	0.08	[0.04; 0.14]	0.0%	0.8%
Odeleye 2021	0	124	0.00	[0.00; 0.03]	0.0%	0.8%
Yonker 2020	34	132	0.26	[0.19; 0.34]	0.0%	0.8%
Alp 2021	41	135	0.30	[0.23; 0.39]	0.0%	0.8%
Gampel 2020	16	135	0.12	[0.07; 0.19]	0.0%	0.8%
Kavanagh 2021	1	141	0.01	[0.00; 0.04]	0.0%	0.8%
Nunziata 2020	104	144	0.72	[0.64; 0.79]	0.0%	0.8%
Chowdhoury 2021	12	146	0.08	[0.04; 0.14]	0.0%	0.8%
Ahmed 2021	36	148	0.24	[0.18; 0.32]	0.0%	0.8%
Yoon 2021	0	154	0.00	[0.00; 0.02]	0.0%	0.8%
Ana Laura 2021	17	156	0.11	[0.06; 0.17]	0.0%	0.8%
Ayed 2020	2	167	0.01	[0.00; 0.04]	0.0%	0.8%
Engels 2022	8	168	0.05	[0.02; 0.09]	0.0%	0.8%
Capozza 2021	8	181	0.04	[0.02; 0.09]	0.0%	0.8%
Gaborieau 2020	157	192	0.82	[0.76; 0.87]	0.0%	0.8%
Karaaslan 2021	93	209	0.44	[0.38; 0.52]	0.0%	0.8%
Lorenzo 2021	62	210	0.30	[0.23; 0.36]	0.0%	0.8%
Aizawa 2021	9	211	0.04	[0.02; 0.08]	0.0%	0.8%
Lynam 2021	7	220	0.03	[0.01; 0.06]	0.0%	0.8%
Lopez-Aguilar 2020	73	226	0.32	[0.26; 0.39]	0.0%	0.8%
Galli 2021	1	230	0.00	[0.00; 0.02]	0.0%	0.8%
Shayganmehr 2021	15	230	0.07	[0.04; 0.11]	0.0%	0.8%
Ghosh 2020	71	236	0.30	[0.24; 0.36]	0.0%	0.8%
Cheng 2022	209	239	0.87	[0.83; 0.91]	0.0%	0.8%
Wang 2020	1	258	0.00	[0.00; 0.02]	0.0%	0.8%
Schneider 2021	18	263	0.07	[0.04; 0.11]	0.0%	0.8%
Rizzo 2021	0	293	0.00	[0.00; 0.01]	0.0%	0.9%
Cooper 2021	17	300	0.06	[0.03; 0.09]	0.0%	0.9%
Salako 2021	50	307	0.16	[0.12; 0.21]	0.0%	0.9%
Isoldi 2021	17	311	0.05	[0.03; 0.09]	0.0%	0.9%
Kuczborska 2021	88	313	0.28	[0.23; 0.33]	0.0%	0.9%
Pokorska-Spiewak 2021	15	319	0.05	[0.03; 0.08]	0.0%	0.9%
MveangNzoghe 2021	44	328	0.13	[0.10; 0.18]	0.0%	0.9%
Paduano 2021	78	349	0.22	[0.18; 0.27]	0.0%	0.9%
Liu 2020	6	366	0.02	[0.01; 0.04]	0.0%	0.9%
Baumgarte 2022	33	368	0.09	[0.06; 0.12]	0.0%	0.9%
Okonkwo 2020	5	373	0.01	[0.00; 0.03]	0.0%	0.9%
Kanthimathinathan 2020	45	389	0.12	[0.09; 0.15]	0.0%	0.9%
Arslan 2021	176	404	0.44	[0.39; 0.49]	0.0%	0.9%
Huete-Perez 2021	181	418	0.43	[0.38; 0.48]	0.0%	0.9%
Berksoy 2021	78	422	0.18	[0.15; 0.23]	0.0%	0.9%
Ibrahim 2020	4	433	0.01	[0.00; 0.02]	0.0%	0.9%
Forster 2022	0	442	0.00	[0.00; 0.01]	0.0%	0.9%
Bandi 2020	25	474	0.05	[0.03; 0.08]	0.0%	0.9%
deLusignan 2020	23	499	0.05	[0.03; 0.07]	0.0%	0.9%
Olivar-Lopez 2020	79	510	0.15	[0.12; 0.19]	0.0%	0.9%
Sahni 2021	114	567	0.20	[0.17; 0.24]	0.0%	0.9%
Vergine 2020	90	567	0.16	[0.13; 0.19]	0.0%	0.9%
Karaci 2021	222	581	0.38	[0.34; 0.42]	0.0%	0.9%
Asseri 2021	79	644	0.12	[0.10; 0.15]	0.0%	0.9%
Dawood 2022	51	652	0.08	[0.06; 0.10]	0.0%	0.9%
Verd 2021	16	691	0.02	[0.01; 0.04]	0.0%	0.9%
Brotons 2021	84	708	0.12	[0.10; 0.14]	0.0%	0.9%
Eleftheriou 2021	51	744	0.07	[0.05; 0.09]	0.0%	0.9%
Meyer 2022	24	748	0.03	[0.02; 0.05]	0.0%	0.9%
Capra 2021	152	753	0.20	[0.17; 0.23]	0.0%	0.9%
Colson 2020	20	845	0.02	[0.01; 0.04]	0.0%	0.9%
Kuchar 2021	15	904	0.02	[0.01; 0.03]	0.0%	0.9%
Wong 2022	326	932	0.35	[0.32; 0.38]	0.0%	0.9%
Aprea 2021	215	952	0.23	[0.20; 0.25]	0.0%	0.9%
Ollier 2022	46	1009	0.05	[0.03; 0.06]	0.0%	0.9%
Kaba 2021	85	1076	0.08	[0.06; 0.10]	0.0%	0.9%
van der Zalm 2021	159	1126	0.14	[0.12; 0.16]	0.0%	0.9%
Lanari 2021	98	1178	0.08	[0.07; 0.10]	0.0%	0.9%
Lu 2020	171	1391	0.12	[0.11; 0.14]	0.0%	0.9%
Gudbjartsson 2020	38	1412	0.03	[0.02; 0.04]	0.0%	0.9%
Kumar 2021	182	1434	0.13	[0.11; 0.15]	0.0%	0.9%
Armocida 2022	127	1484	0.09	[0.07; 0.10]	0.0%	0.9%
Murillo- Zamora 2020	294	1849	0.16	[0.14; 0.18]	0.0%	0.9%
Ladhani 2021	15	1896	0.01	[0.00; 0.01]	0.0%	0.9%
Ciofi Degli Atti 2020	33	1970	0.02	[0.01; 0.02]	0.0%	0.9%
Ortiz-Pinto 2022	170	1985	0.09	[0.07; 0.10]	0.0%	0.9%
Dong 2020	728	2135	0.34	[0.32; 0.36]	0.0%	0.9%
Lazzerini 2021	190	2494	0.08	[0.07; 0.09]	0.0%	0.9%
Dilber 2021	382	2530	0.15	[0.14; 0.17]	0.0%	0.9%
Yayla 2020	211	2530	0.08	[0.07; 0.09]	0.0%	0.9%
Calvani 2021	96	2837	0.03	[0.03; 0.04]	0.0%	0.9%
Singh 2022	255	2919	0.09	[0.08; 0.10]	0.0%	0.9%
Goktug 2021	621	3118	0.20	[0.19; 0.21]	0.0%	0.9%
Kapoor 2021	295	3180	0.09	[0.08; 0.10]	0.0%	0.9%
Rha 2020	4	3187	0.00	[0.00; 0.00]	0.0%	0.9%
Hernandez-Garduno 2020	961	3696	0.26	[0.25; 0.27]	0.0%	0.9%
Vogel 2022	5	3752	0.00	[0.00; 0.00]	0.0%	0.9%
Matteudi 2021	201	3810	0.05	[0.05; 0.06]	0.0%	0.9%
Michos 2021	90	4901	0.02	[0.01; 0.02]	0.0%	0.9%
Meyer 2021	22	5730	0.00	[0.00; 0.01]	0.0%	0.9%
Colson 2020	346	5767	0.06	[0.05; 0.07]	0.0%	0.9%
Biko 2020	313	5969	0.05	[0.05; 0.06]	0.0%	0.9%
Mania 2021	106	6115	0.02	[0.01; 0.02]	0.0%	0.9%
Levy 2020	382	6490	0.06	[0.05; 0.06]	0.1%	0.9%
Otto 2020	377	6835	0.06	[0.05; 0.06]	0.1%	0.9%
Gavriliu 2021	172	7902	0.02	[0.02; 0.03]	0.1%	0.9%
Song 2021	249	8162	0.03	[0.03; 0.03]	0.1%	0.9%
Otto 2021	1284	10138	0.13	[0.12; 0.13]	0.1%	0.9%
Funk 2022	3222	10382	0.31	[0.30; 0.32]	0.1%	0.9%
Pandey 2020	22	10777	0.00	[0.00; 0.00]	0.1%	0.9%
Rivas-Ruiz 2020	1443	11405	0.13	[0.12; 0.13]	0.1%	0.9%
Talarico 2021	1276	11728	0.11	[0.10; 0.11]	0.1%	0.9%
Tosif 2021	180	14419	0.01	[0.01; 0.01]	0.1%	0.9%
Foster 2020	1136	15815	0.07	[0.07; 0.08]	0.1%	0.9%
Peaper 2021	734	23137	0.03	[0.03; 0.03]	0.2%	0.9%
Abo 2021	186	28893	0.01	[0.01; 0.01]	0.2%	0.9%
Sola 2021	250	33041	0.01	[0.01; 0.01]	0.3%	0.9%
Lindsay 2021	2120	39691	0.05	[0.05; 0.06]	0.3%	0.9%
Hendler 2021	228	41324	0.01	[0.00; 0.01]	0.3%	0.9%
Dash 2021	5988	51621	0.12	[0.11; 0.12]	0.4%	0.9%
Cohen 2021	6207	64409	0.10	[0.09; 0.10]	0.5%	0.9%
Navarro-Olivos 2021	48505	167856	0.29	[0.29; 0.29]	1.3%	0.9%
Kufa 2022	45609	315570	0.14	[0.14; 0.15]	2.4%	0.9%
Soriano-Arandes 2021	26665	417578	0.06	[0.06; 0.06]	3.2%	0.9%
Perramon 2021	91857	1654099	0.06	[0.06; 0.06]	12.8%	0.9%
Imamura 2021	2450	9860000	0.00	[0.00; 0.00]	76.3%	0.9%
**Common effect model**		**12916952**	**0.00**	**[0.00; 0.00]**	**100.0%**	**--**
**Random effects model**			**0.09**	**[0.07; 0.12]**	**--**	**100.0%**
**Prediction interval**				**[0.00; 0.45]**		

Heterogeneity: $I^2 = 100\%$, $\tau^2 = 0.0460$, $p = 0$

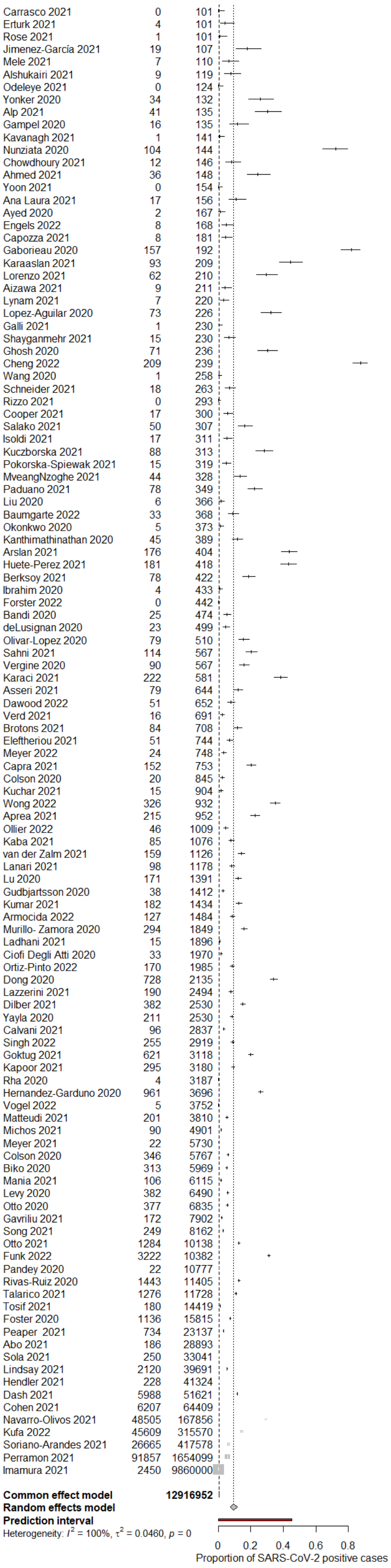

Figure S2: Influential plots for test positivity analysis

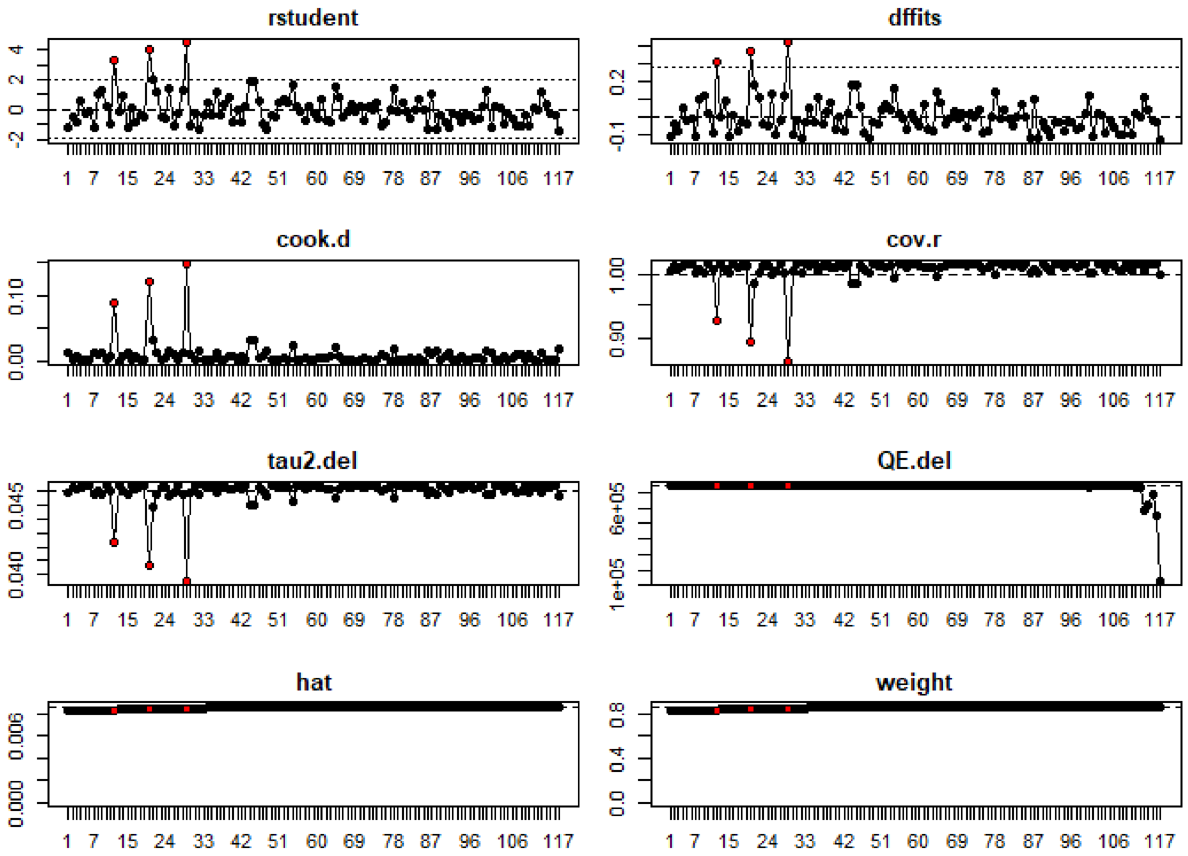

Figure S3: Subgroup analysis: Plot of subgroup analysis according to WHO region

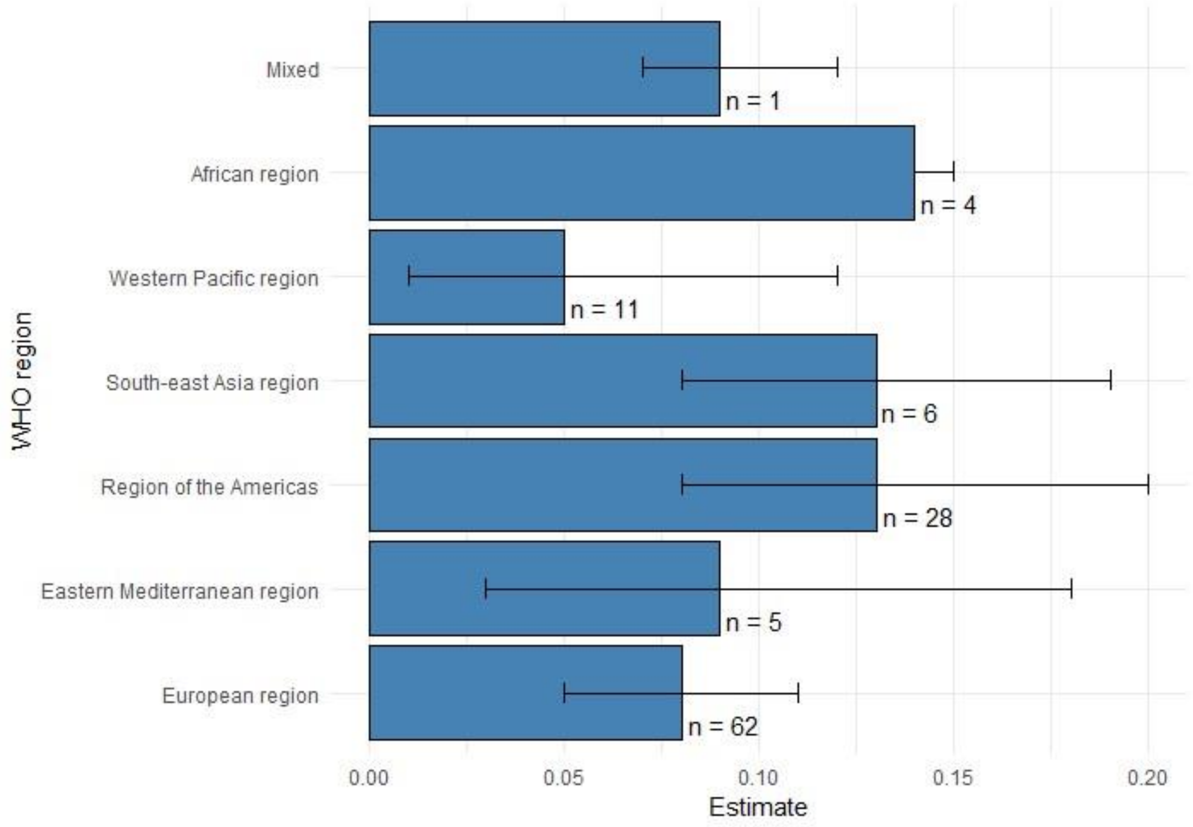

n = Number of studies

Figure S4: Subgroup analysis: Plot of subgroup analysis according to country income level

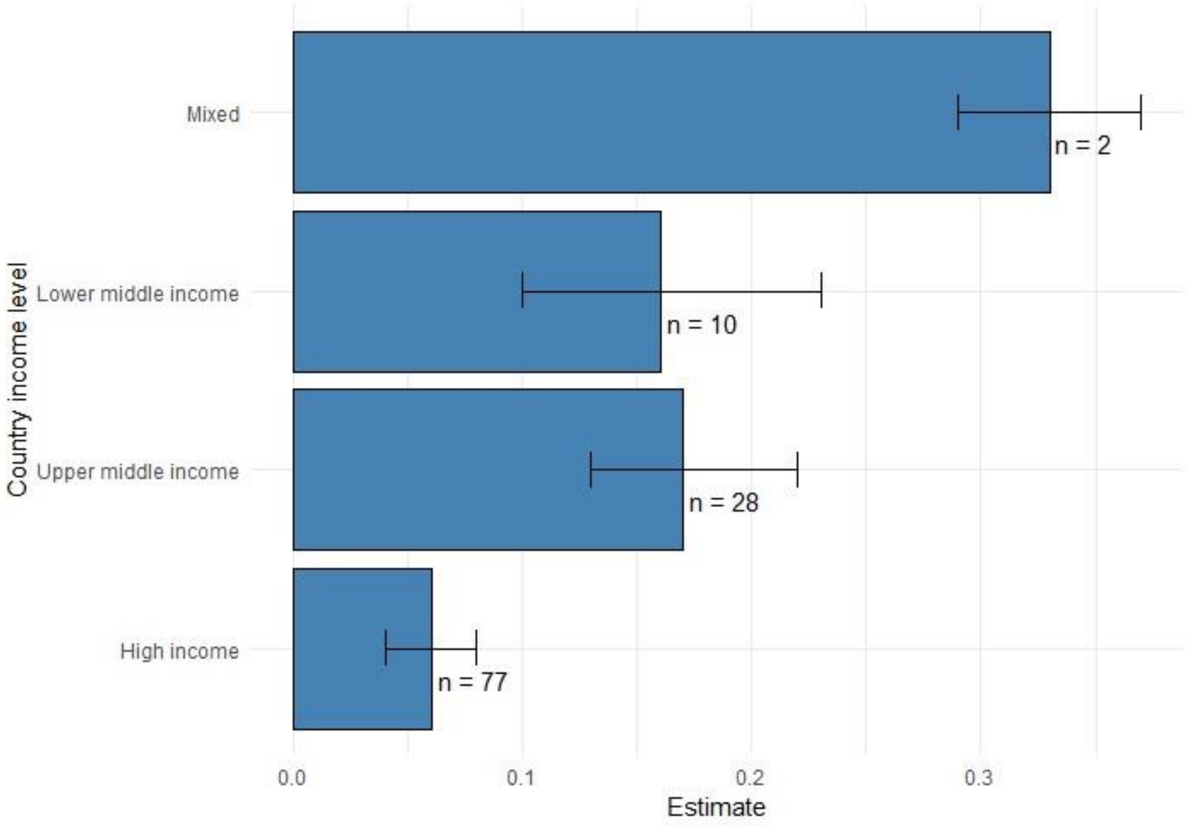

n = Number of studies

Figure S5: Subgroup analysis: Plot of subgroup analysis according to study setting

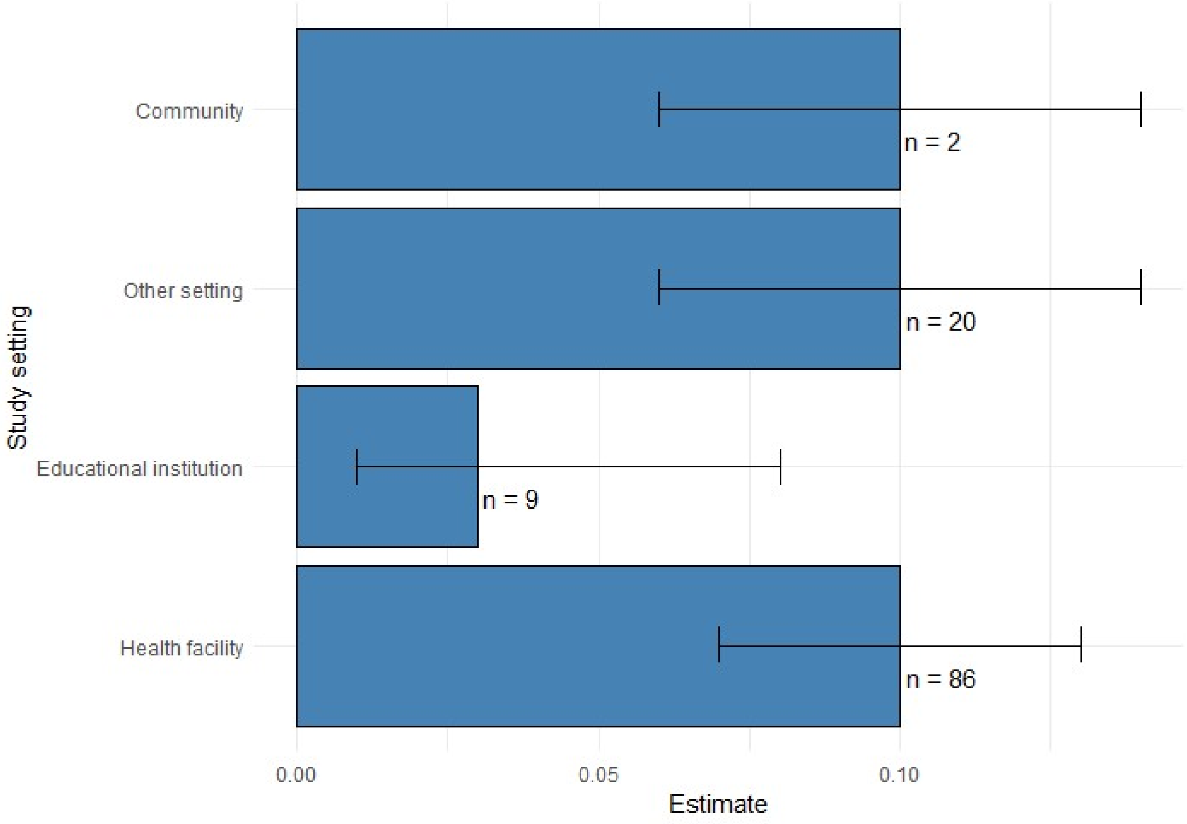

n = Number of studies

Figure S6: Subgroup analysis: Plot of subgroup analysis according to testing method

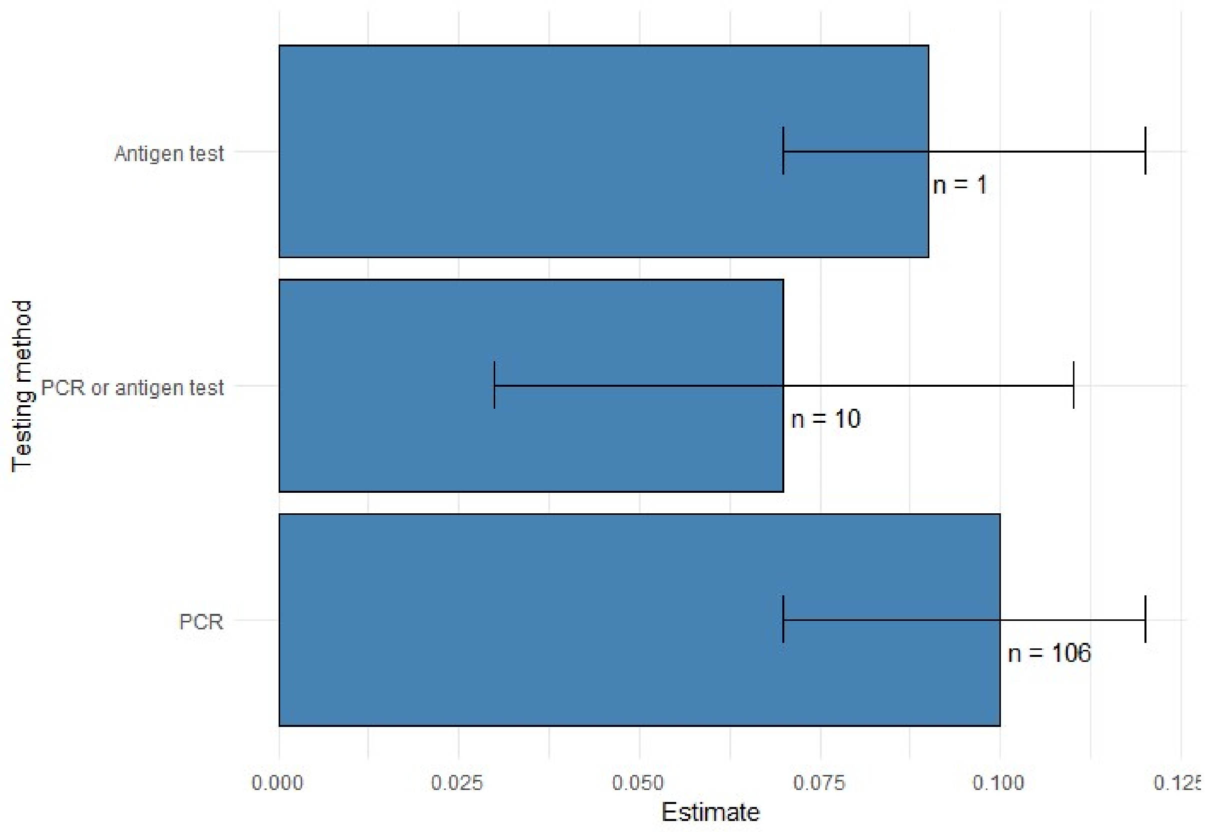

n = Number of studies

Figure S7: Subgroup analysis: Plot of subgroup analysis according to SARS-CoV-2 dominant variant in the country at the mid study time

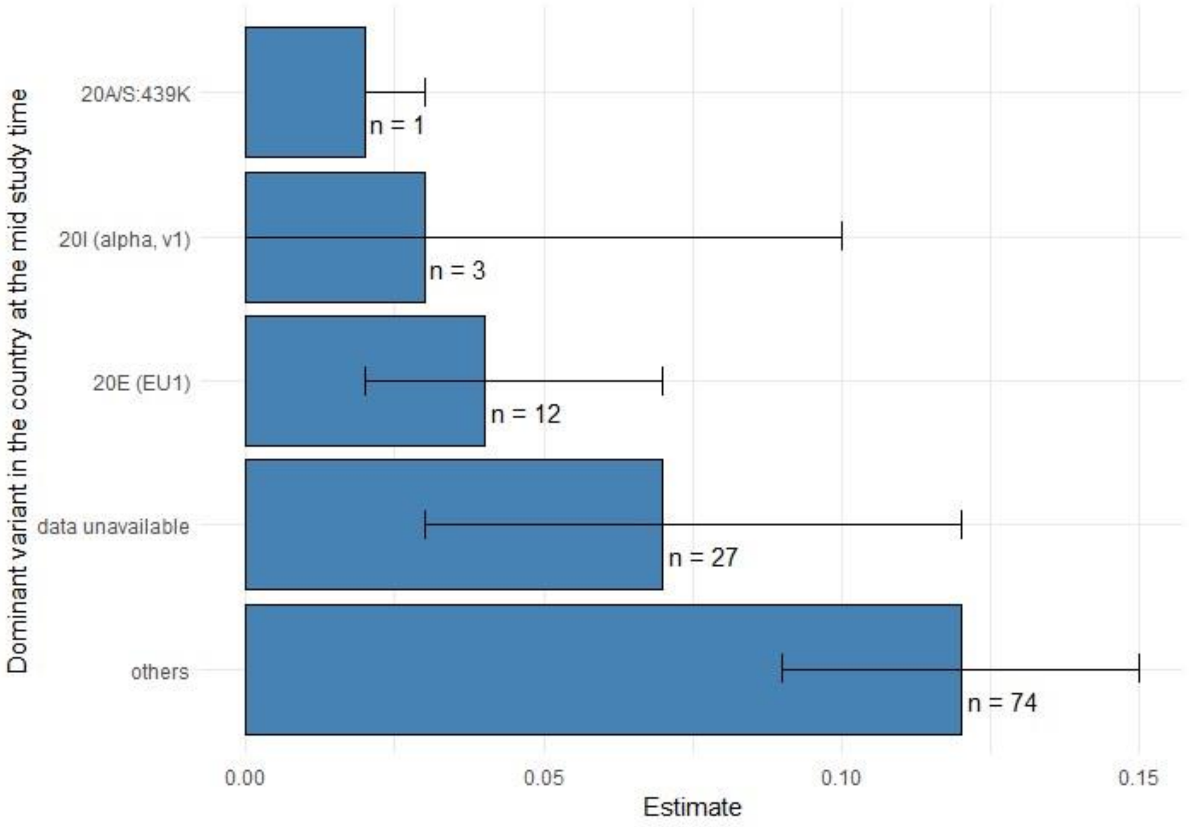

n = Number of studies

Figure S8: Forest plot: Pooled relative risk of male sex as risk factor of SARS-CoV-2 infection in people aged ≤18 years

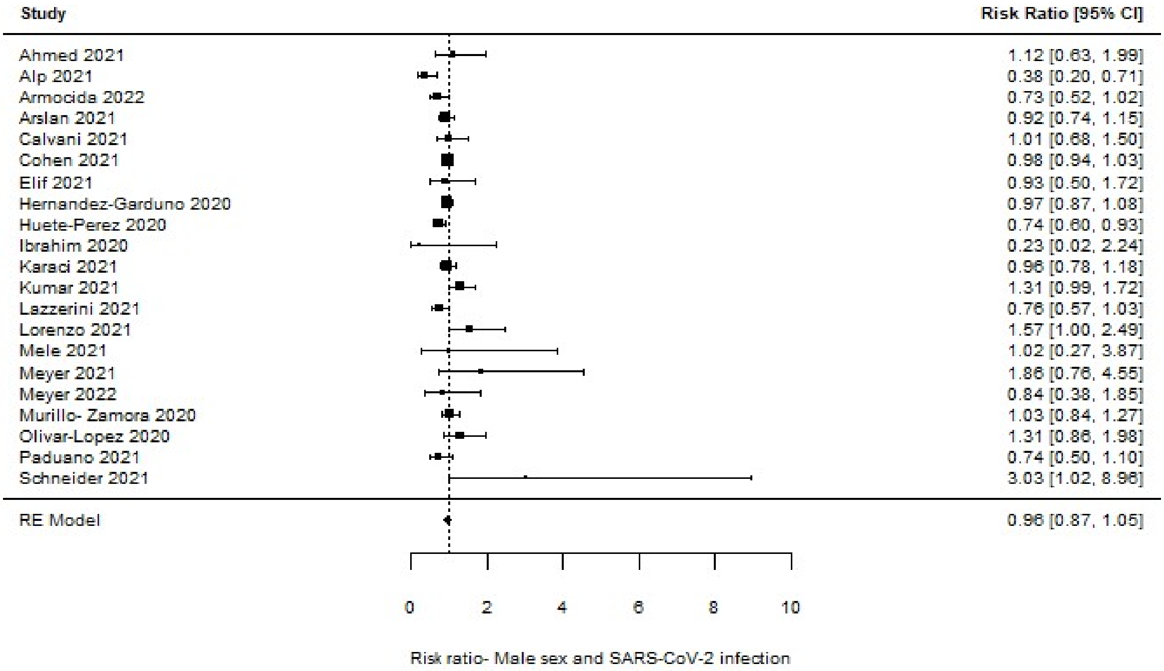

Figure S9: Forest plot: Pooled proportion of people aged ≤18 years presenting with asymptomatic SARS-CoV-2 infection

Study	Events	Total	Proportion	95%-CI	Weight (common)	Weight (random)
Ozlu 2022	72	100	0.72	[0.62; 0.81]	0.1%	1.1%
Pande 2021	9	100	0.09	[0.04; 0.16]	0.1%	1.1%
Besli 2021	7	104	0.07	[0.03; 0.13]	0.1%	1.1%
Yilmaz 2020	59	105	0.56	[0.46; 0.66]	0.1%	1.1%
Alshengeti 2022	7	106	0.07	[0.03; 0.13]	0.1%	1.1%
Lu 2020	29	110	0.26	[0.18; 0.36]	0.1%	1.1%
Ennab 2021	43	111	0.39	[0.30; 0.48]	0.1%	1.1%
Du 2020	41	114	0.36	[0.27; 0.45]	0.1%	1.1%
Rabha 2021	3	115	0.03	[0.01; 0.07]	0.1%	1.1%
Kushner 2021	46	117	0.39	[0.30; 0.49]	0.1%	1.1%
Sharma 2020	87	121	0.72	[0.63; 0.80]	0.1%	1.1%
Cofre 2020	39	127	0.31	[0.23; 0.40]	0.1%	1.1%
Buonsenso 2021	33	129	0.26	[0.18; 0.34]	0.1%	1.1%
Jiang 2022	2	129	0.02	[0.00; 0.05]	0.1%	1.1%
Gumus 2021	71	130	0.55	[0.46; 0.63]	0.1%	1.1%
Haeusler 2021	42	131	0.32	[0.24; 0.41]	0.1%	1.1%
Alsharrah 2020	91	134	0.68	[0.59; 0.76]	0.2%	1.1%
Matteudi 2021	38	137	0.28	[0.20; 0.36]	0.2%	1.1%
Shahid 2021	88	141	0.62	[0.54; 0.70]	0.2%	1.1%
Indriyani 2021	76	146	0.52	[0.44; 0.60]	0.2%	1.1%
Lazzerini 2021	8	159	0.05	[0.02; 0.10]	0.2%	1.1%
Loconsole 2020	104	166	0.63	[0.55; 0.70]	0.2%	1.1%
Akkoc 2021	34	167	0.20	[0.15; 0.27]	0.2%	1.1%
Garazzino 2020	4	168	0.02	[0.01; 0.06]	0.2%	1.1%
Chiang 2021	58	169	0.34	[0.27; 0.42]	0.2%	1.1%
Kumar 2021	120	170	0.71	[0.63; 0.77]	0.2%	1.1%
Ortiz-Pinto 2022	86	170	0.51	[0.43; 0.58]	0.2%	1.1%
Parri 2020	29	170	0.17	[0.12; 0.24]	0.2%	1.1%
Lu 2020	27	171	0.16	[0.11; 0.22]	0.2%	1.1%
Gavriliu 2021	30	172	0.17	[0.12; 0.24]	0.2%	1.1%
Ustundag 2021	60	173	0.35	[0.28; 0.42]	0.2%	1.1%
Arslan 2021	59	176	0.34	[0.27; 0.41]	0.2%	1.1%
Corso 2021	25	179	0.14	[0.09; 0.20]	0.2%	1.1%
Alattas 2021	60	180	0.33	[0.26; 0.41]	0.2%	1.1%
Tosif 2021	60	180	0.33	[0.26; 0.41]	0.2%	1.1%
Huete- Perez 2021	46	181	0.25	[0.19; 0.32]	0.2%	1.1%
Du 2021	24	182	0.13	[0.09; 0.19]	0.2%	1.1%
Kaliyan 2021	136	191	0.71	[0.64; 0.78]	0.2%	1.1%
Celik 2021	150	197	0.76	[0.70; 0.82]	0.2%	1.1%
Peng 2021	65	201	0.32	[0.26; 0.39]	0.2%	1.1%
Maltezou 2020	111	203	0.55	[0.48; 0.62]	0.2%	1.1%
Bai 2021	34	209	0.16	[0.12; 0.22]	0.2%	1.1%
Aprea 2021	75	215	0.35	[0.29; 0.42]	0.2%	1.1%
Karaci 2021	69	222	0.31	[0.25; 0.38]	0.2%	1.1%
Ji 2021	0	241	0.00	[0.00; 0.02]	0.3%	1.1%
Xiong 2020	51	244	0.21	[0.16; 0.27]	0.3%	1.2%
Ng 2021	151	261	0.58	[0.52; 0.64]	0.3%	1.2%
Gujar 2021	180	274	0.66	[0.60; 0.71]	0.3%	1.2%
Ferraro D 2021	20	280	0.07	[0.04; 0.11]	0.3%	1.2%
Elghoudi 2020	76	288	0.26	[0.21; 0.32]	0.3%	1.2%
Biko 2020	92	313	0.29	[0.24; 0.35]	0.4%	1.2%
Wong 2022	161	326	0.49	[0.44; 0.55]	0.4%	1.2%
Otto 2020	52	377	0.14	[0.10; 0.18]	0.4%	1.2%
Ibrahim 2021	98	393	0.25	[0.21; 0.30]	0.4%	1.2%
Chua 2021	154	397	0.39	[0.34; 0.44]	0.4%	1.2%
Krithika 2021	215	400	0.54	[0.49; 0.59]	0.4%	1.2%
Antúnez-Montes 2021	53	409	0.13	[0.10; 0.17]	0.5%	1.2%
Angulruengkitt 2021	82	416	0.20	[0.16; 0.24]	0.5%	1.2%
Chua 2020	111	423	0.26	[0.22; 0.31]	0.5%	1.2%
Sena 2021	6	446	0.01	[0.00; 0.03]	0.5%	1.2%
Sananez 2021	84	550	0.15	[0.12; 0.19]	0.6%	1.2%
AlGhamdi 2022	221	567	0.39	[0.35; 0.43]	0.6%	1.2%
Gotzinger 2020	92	582	0.16	[0.13; 0.19]	0.7%	1.2%
Goktug 2021	24	621	0.04	[0.02; 0.06]	0.7%	1.2%
Almuzaini 2021	129	654	0.20	[0.17; 0.23]	0.7%	1.2%
Hijazi 2021	288	660	0.44	[0.40; 0.48]	0.7%	1.2%
Uka 2021	39	678	0.06	[0.04; 0.08]	0.8%	1.2%
Dong 2020	94	726	0.13	[0.11; 0.16]	0.8%	1.2%
Alharbi 2021	405	742	0.55	[0.51; 0.58]	0.8%	1.2%
Alqayoudhi 2021	293	1026	0.29	[0.26; 0.31]	1.1%	1.2%
Shapiro Ben David 2021	432	1032	0.42	[0.39; 0.45]	1.2%	1.2%
Shoji 2021	308	1038	0.30	[0.27; 0.33]	1.2%	1.2%
Soriano-Arandes 2021	491	1040	0.47	[0.44; 0.50]	1.2%	1.2%
Karbuz 2021	263	1156	0.23	[0.20; 0.25]	1.3%	1.2%
Talarico 2021	804	1276	0.63	[0.60; 0.66]	1.4%	1.2%
Pokorska-Spiewak 2021	271	1283	0.21	[0.19; 0.23]	1.4%	1.2%
Shoji 2022	281	1299	0.22	[0.19; 0.24]	1.5%	1.2%
See 2021	853	1489	0.57	[0.55; 0.60]	1.7%	1.2%
Reis 2021	397	2089	0.19	[0.17; 0.21]	2.3%	1.2%
Morban 2021	1062	2992	0.35	[0.34; 0.37]	3.3%	1.2%
Funk 2022	172	3222	0.05	[0.05; 0.06]	3.6%	1.2%
Bellino 2020	785	3836	0.20	[0.19; 0.22]	4.3%	1.2%
Bolanos-Almeida 2021	854	5062	0.17	[0.16; 0.18]	5.7%	1.2%
Madani 2021	945	6610	0.14	[0.13; 0.15]	7.4%	1.2%
Barrera 2021	5930	10437	0.57	[0.56; 0.58]	11.7%	1.2%
Musa 2021	7258	11445	0.63	[0.63; 0.64]	12.8%	1.2%
Jang 2022	6555	14967	0.44	[0.43; 0.45]	16.7%	1.2%
**Common effect model**		**89345**	**0.36**	**[0.35; 0.36]**	**100.0%**	**--**
**Random effects model**			**0.30**	**[0.25; 0.35]**	**--**	**100.0%**

Heterogeneity: $I^2 = 100\%$, $\tau^2 = 0.0564$, $p = 0$

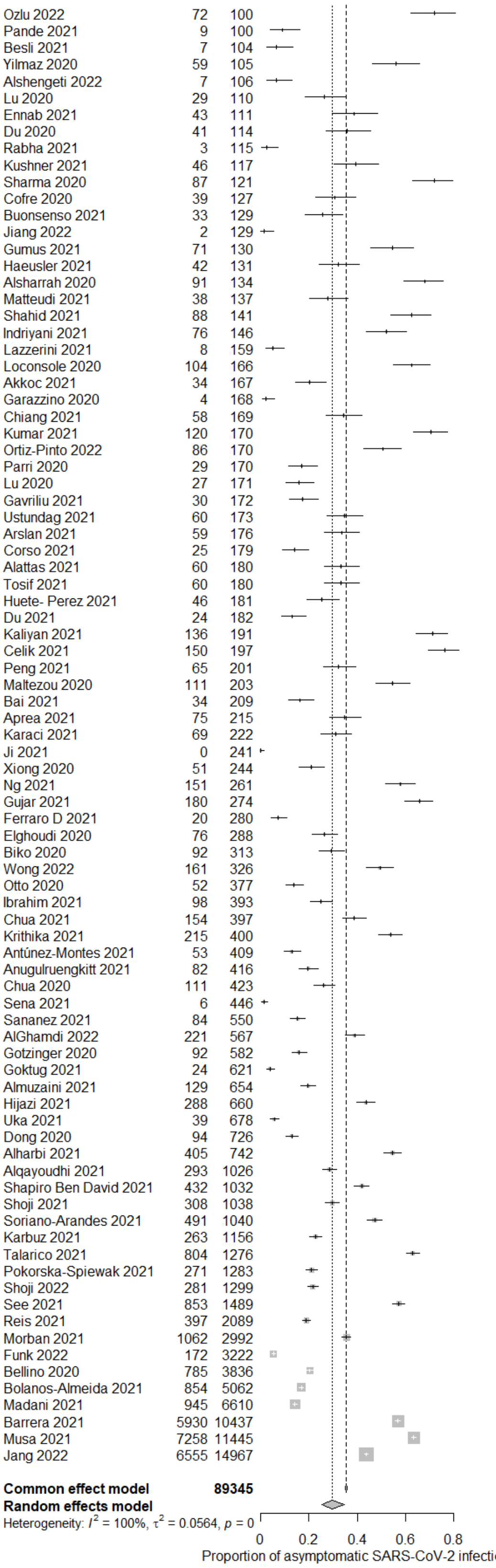

Figure S10: Forest plot: Pooled proportion of people aged ≤18 years presenting with mild COVID-19 disease

Study	Events	Total	Proportion	95%-CI	Weight (common)	Weight (random)
Ozlu 2022	14	100	0.14	[0.08; 0.22]	0.4%	2.0%
Pande 2021	33	100	0.33	[0.24; 0.43]	0.4%	2.0%
Besli 2021	84	104	0.81	[0.72; 0.88]	0.4%	2.0%
Yilmaz 2020	32	105	0.30	[0.22; 0.40]	0.4%	2.0%
Alshengeti 2022	89	106	0.84	[0.76; 0.90]	0.5%	2.0%
Ennab 2021	32	111	0.29	[0.21; 0.38]	0.5%	2.0%
Sharma 2020	27	121	0.22	[0.15; 0.31]	0.5%	2.0%
Qian 2021	57	127	0.45	[0.36; 0.54]	0.5%	2.0%
Tang 2021	46	127	0.36	[0.28; 0.45]	0.5%	2.0%
Jiang 2022	126	129	0.98	[0.93; 1.00]	0.6%	2.0%
Haeusler 2021	61	131	0.47	[0.38; 0.55]	0.6%	2.0%
Alsharrah 2020	43	134	0.32	[0.24; 0.41]	0.6%	2.0%
Shahid 2021	88	141	0.62	[0.54; 0.70]	0.6%	2.0%
Indriyani 2021	17	146	0.12	[0.07; 0.18]	0.6%	2.0%
Lazzerini 2021	124	159	0.78	[0.71; 0.84]	0.7%	2.0%
Loconsole 2020	37	166	0.22	[0.16; 0.29]	0.7%	2.0%
Akkoc 2021	113	167	0.68	[0.60; 0.75]	0.7%	2.0%
Chiang 2021	93	169	0.55	[0.47; 0.63]	0.7%	2.0%
Ortiz-Pinto 2022	84	170	0.49	[0.42; 0.57]	0.7%	2.0%
Parri 2020	107	170	0.63	[0.55; 0.70]	0.7%	2.0%
Gavriliu 2021	30	172	0.17	[0.12; 0.24]	0.7%	2.0%
Ustundag 2021	98	173	0.57	[0.49; 0.64]	0.7%	2.0%
Arslan 2021	94	176	0.53	[0.46; 0.61]	0.8%	2.0%
Corso 2021	41	179	0.23	[0.17; 0.30]	0.8%	2.0%
Du 2021	154	182	0.85	[0.79; 0.90]	0.8%	2.0%
Maltezou 2020	68	203	0.33	[0.27; 0.40]	0.9%	2.0%
Bai 2021	172	209	0.82	[0.76; 0.87]	0.9%	2.0%
Dominguez-Rodriguez 2021	37	214	0.17	[0.12; 0.23]	0.9%	2.0%
Karaci 2021	118	222	0.53	[0.46; 0.60]	0.9%	2.0%
Ji 2021	10	241	0.04	[0.02; 0.07]	1.0%	2.0%
Sharma 2021	148	255	0.58	[0.52; 0.64]	1.1%	2.0%
Singh 2022	144	255	0.56	[0.50; 0.63]	1.1%	2.0%
Gujar 2021	94	274	0.34	[0.29; 0.40]	1.2%	2.0%
Ferraro D 2021	184	280	0.66	[0.60; 0.71]	1.2%	2.0%
Kara 2021	198	292	0.68	[0.62; 0.73]	1.2%	2.0%
Wong 2022	321	326	0.98	[0.96; 1.00]	1.4%	2.0%
Chua 2021	394	397	0.99	[0.98; 1.00]	1.7%	2.0%
Krithika 2021	127	400	0.32	[0.27; 0.37]	1.7%	2.0%
Anugulruengkitt 2021	232	416	0.56	[0.51; 0.61]	1.8%	2.0%
Sananez 2021	358	550	0.65	[0.61; 0.69]	2.3%	2.0%
AlGhamdi 2022	319	567	0.56	[0.52; 0.60]	2.4%	2.0%
Rabha 2021	519	588	0.88	[0.85; 0.91]	2.5%	2.0%
Goktug 2021	546	621	0.88	[0.85; 0.90]	2.6%	2.0%
Bayesheva 2021	558	650	0.86	[0.83; 0.88]	2.8%	2.0%
Buta 2022	52	701	0.07	[0.06; 0.10]	3.0%	2.0%
Dong 2020	314	726	0.43	[0.40; 0.47]	3.1%	2.0%
Karbuz 2021	668	1156	0.58	[0.55; 0.61]	4.9%	2.0%
See 2021	622	1489	0.42	[0.39; 0.44]	6.3%	2.0%
Bellino 2020	652	3836	0.17	[0.16; 0.18]	16.3%	2.0%
Bolanos-Almeida 2021	4022	5062	0.79	[0.78; 0.81]	21.5%	2.0%
**Common effect model**		**23495**	**0.54**	**[0.54; 0.55]**	**100.0%**	--
**Random effects model**			**0.52**	**[0.44; 0.61]**	--	**100.0%**

Heterogeneity: $I^2 = 99\%$, $\tau^2 = 0.0909$, $p = 0$

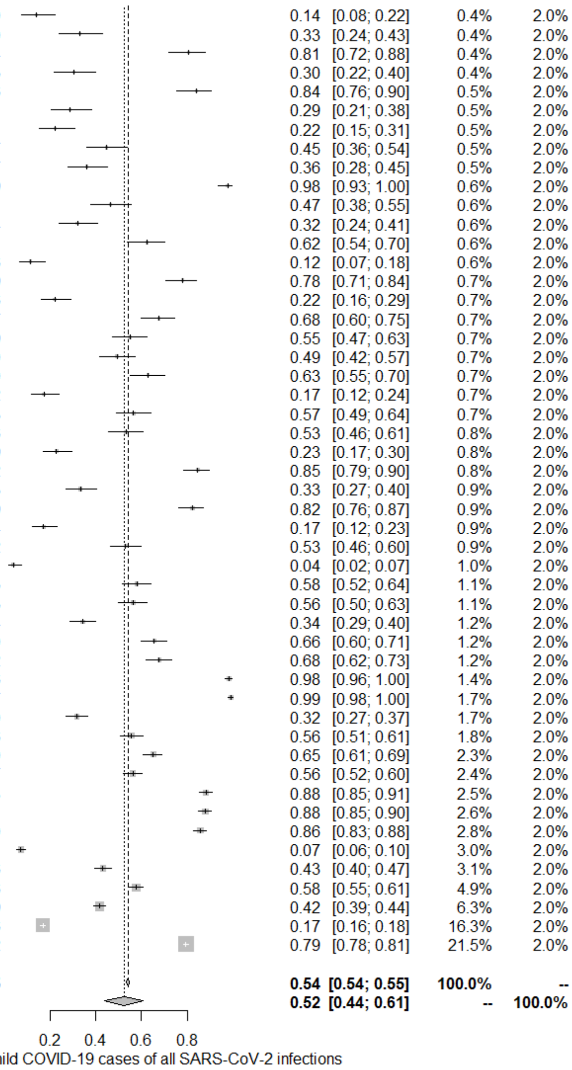

Figure S11: Forest plot: Pooled proportion of people aged ≤18 years presenting with moderate severity of COVID-19 disease

Study	Events	Total	Proportion	95%-CI	Weight (common)	Weight (random)
Ozlu 2022	14	100	0.14	[0.08; 0.22]	0.8%	2.3%
Pande 2021	21	100	0.21	[0.13; 0.30]	0.8%	2.3%
Sharma 2021	26	100	0.26	[0.18; 0.36]	0.8%	2.3%
Besli 2021	9	104	0.09	[0.04; 0.16]	0.8%	2.3%
Yilmaz 2020	11	105	0.10	[0.05; 0.18]	0.8%	2.3%
Alshengeti 2022	4	106	0.04	[0.01; 0.09]	0.8%	2.3%
Ennab 2021	36	111	0.32	[0.24; 0.42]	0.9%	2.3%
Kushner 2021	33	117	0.28	[0.20; 0.37]	0.9%	2.3%
Kapoor 2021	84	120	0.70	[0.61; 0.78]	0.9%	2.3%
Sharma 2020	6	121	0.05	[0.02; 0.10]	0.9%	2.3%
Qian 2021	63	127	0.50	[0.41; 0.59]	1.0%	2.3%
Tang 2021	124	127	0.98	[0.93; 1.00]	1.0%	2.3%
Jiang 2022	1	129	0.01	[0.00; 0.04]	1.0%	2.3%
Haeusler 2021	11	131	0.08	[0.04; 0.15]	1.0%	2.3%
Alsharrah 2020	0	134	0.00	[0.00; 0.03]	1.0%	2.3%
Shahid 2021	36	141	0.26	[0.19; 0.34]	1.1%	2.3%
Indriyani 2021	4	146	0.03	[0.01; 0.07]	1.1%	2.3%
Lazzerini 2021	20	159	0.13	[0.08; 0.19]	1.2%	2.3%
Loconsole 2020	22	166	0.13	[0.08; 0.19]	1.3%	2.3%
Chiang 2021	14	169	0.08	[0.05; 0.14]	1.3%	2.3%
Parri 2020	19	170	0.11	[0.07; 0.17]	1.3%	2.3%
Gavriliu 2021	142	172	0.83	[0.76; 0.88]	1.3%	2.3%
Ustundag 2021	11	173	0.06	[0.03; 0.11]	1.3%	2.3%
Arslan 2021	22	176	0.12	[0.08; 0.18]	1.3%	2.3%
Alattas 2021	117	180	0.65	[0.58; 0.72]	1.4%	2.3%
Maltezou 2020	23	203	0.11	[0.07; 0.17]	1.6%	2.3%
Karaci 2021	25	222	0.11	[0.07; 0.16]	1.7%	2.3%
Ji 2021	217	241	0.90	[0.86; 0.94]	1.8%	2.3%
Singh 2022	27	255	0.11	[0.07; 0.15]	2.0%	2.3%
Gujar 2021	0	274	0.00	[0.00; 0.01]	2.1%	2.3%
Ferraro D 2021	3	280	0.01	[0.00; 0.03]	2.1%	2.3%
Kara 2021	82	292	0.28	[0.23; 0.34]	2.2%	2.3%
Murillo- Zamora 2020	216	294	0.73	[0.68; 0.78]	2.2%	2.3%
Wong 2022	1	326	0.00	[0.00; 0.02]	2.5%	2.3%
Krithika 2021	12	400	0.03	[0.02; 0.05]	3.1%	2.3%
Anugulruengkitt 2021	90	416	0.22	[0.18; 0.26]	3.2%	2.3%
Sananez 2021	100	550	0.18	[0.15; 0.22]	4.2%	2.3%
Rabha 2021	8	588	0.01	[0.01; 0.03]	4.5%	2.3%
Goktug 2021	42	621	0.07	[0.05; 0.09]	4.7%	2.3%
Bayesheva 2021	88	650	0.14	[0.11; 0.16]	5.0%	2.3%
Buta 2022	590	701	0.84	[0.81; 0.87]	5.4%	2.3%
Dong 2020	298	726	0.41	[0.37; 0.45]	5.6%	2.3%
Karbuz 2021	209	1156	0.18	[0.16; 0.20]	8.8%	2.3%
See 2021	7	1489	0.00	[0.00; 0.01]	11.4%	2.3%
**Common effect model**		**13068**	**0.17**	**[0.17; 0.18]**	**100.0%**	**--**
**Random effects model**			**0.20**	**[0.12; 0.28]**	**--**	**100.0%**

Heterogeneity: $I^2 = 99\%$, $\tau^2 = 0.1168$, $p = 0$

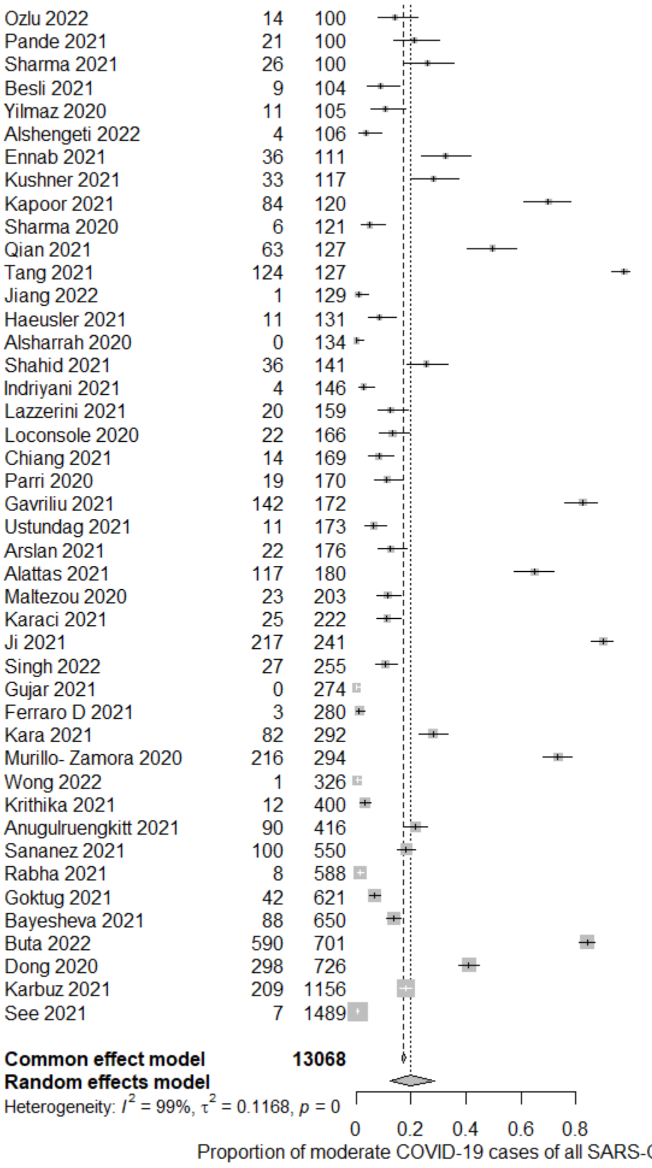

Figure S12: Forest plot: Pooled proportion of people aged ≤18 years presenting with severe or critical COVID-19 disease

Study	Events	Total	Proportion	95%-CI	Weight (common)	Weight (random)
Ozlu 2022	0	100	0.00	[0.00; 0.04]	0.1%	1.7%
Pande 2021	37	100	0.37	[0.28; 0.47]	0.1%	1.7%
Sharma 2021	44	100	0.44	[0.34; 0.54]	0.1%	1.7%
Besli 2021	4	104	0.04	[0.01; 0.10]	0.1%	1.7%
Yilmaz 2020	3	105	0.03	[0.01; 0.08]	0.1%	1.7%
Alshengeti 2022	5	106	0.05	[0.02; 0.11]	0.1%	1.7%
Ennab 2021	32	111	0.29	[0.21; 0.38]	0.1%	1.7%
Rabha 2021	0	115	0.00	[0.00; 0.03]	0.2%	1.7%
Kushner 2021	24	117	0.21	[0.14; 0.29]	0.2%	1.7%
Kapoor 2021	36	120	0.30	[0.22; 0.39]	0.2%	1.7%
Sharma 2020	1	121	0.01	[0.00; 0.05]	0.2%	1.7%
Qian 2021	7	127	0.06	[0.02; 0.11]	0.2%	1.7%
Tang 2021	3	127	0.02	[0.00; 0.07]	0.2%	1.7%
Jiang 2022	0	129	0.00	[0.00; 0.03]	0.2%	1.7%
Haeusler 2021	17	131	0.13	[0.08; 0.20]	0.2%	1.7%
Alsharrah 2020	0	134	0.00	[0.00; 0.03]	0.2%	1.7%
Shahid 2021	17	141	0.12	[0.07; 0.19]	0.2%	1.7%
Indriyani 2021	49	146	0.34	[0.26; 0.42]	0.2%	1.7%
Capra 2021	0	152	0.00	[0.00; 0.02]	0.2%	1.7%
Lazzerini 2021	7	159	0.04	[0.02; 0.09]	0.2%	1.7%
Loconsole 2020	3	166	0.02	[0.00; 0.05]	0.2%	1.7%
Sedighi 2021	70	166	0.42	[0.35; 0.50]	0.2%	1.7%
Chiang 2021	4	169	0.02	[0.01; 0.06]	0.2%	1.7%
Parri 2020	2	170	0.01	[0.00; 0.04]	0.2%	1.7%
Ustundag 2021	4	173	0.02	[0.01; 0.06]	0.2%	1.7%
Arslan 2021	1	176	0.01	[0.00; 0.03]	0.2%	1.7%
Corso 2021	41	179	0.23	[0.17; 0.30]	0.2%	1.7%
Alattas 2021	3	180	0.02	[0.00; 0.05]	0.2%	1.7%
Du 2021	4	182	0.02	[0.01; 0.06]	0.2%	1.7%
Maltezou 2020	1	203	0.00	[0.00; 0.03]	0.3%	1.7%
Hobbs 2022	104	206	0.50	[0.43; 0.58]	0.3%	1.7%
Bai 2021	3	209	0.01	[0.00; 0.04]	0.3%	1.7%
Guzman 2021	20	213	0.09	[0.06; 0.14]	0.3%	1.7%
Karaci 2021	10	222	0.05	[0.02; 0.08]	0.3%	1.7%
Ji 2021	14	241	0.06	[0.03; 0.10]	0.3%	1.7%
Singh 2022	73	255	0.29	[0.23; 0.35]	0.3%	1.7%
Gujar 2021	0	274	0.00	[0.00; 0.01]	0.4%	1.7%
Ferraro D 2021	46	280	0.16	[0.12; 0.21]	0.4%	1.7%
Kara 2021	12	292	0.04	[0.02; 0.07]	0.4%	1.7%
Murillo- Zamora 2020	78	294	0.27	[0.22; 0.32]	0.4%	1.7%
Wong 2022	3	326	0.01	[0.00; 0.03]	0.4%	1.7%
Saleh 2021	103	398	0.26	[0.22; 0.30]	0.5%	1.7%
Krithika 2021	3	400	0.01	[0.00; 0.02]	0.5%	1.7%
Anugulruengkitt 2021	12	416	0.03	[0.01; 0.05]	0.5%	1.7%
Oslon 2022	196	445	0.44	[0.39; 0.49]	0.6%	1.7%
Osmanov 2022	14	515	0.03	[0.01; 0.05]	0.7%	1.7%
Sananez 2021	8	550	0.01	[0.01; 0.03]	0.7%	1.7%
AlGhamdi 2022	0	567	0.00	[0.00; 0.01]	0.7%	1.7%
Rabha 2021	61	588	0.10	[0.08; 0.13]	0.8%	1.7%
Goktug 2021	9	621	0.01	[0.01; 0.03]	0.8%	1.7%
Bayesheva 2021	4	650	0.01	[0.00; 0.02]	0.9%	1.7%
Buta 2022	59	701	0.08	[0.06; 0.11]	0.9%	1.7%
Dong 2020	21	726	0.03	[0.02; 0.04]	1.0%	1.7%
Karbuz 2021	16	1156	0.01	[0.01; 0.02]	1.5%	1.7%
See 2021	4	1489	0.00	[0.00; 0.01]	2.0%	1.7%
Reis 2021	0	2089	0.00	[0.00; 0.00]	2.7%	1.7%
Bellino 2020	86	3836	0.02	[0.02; 0.03]	5.0%	1.7%
Jang 2022	1	14967	0.00	[0.00; 0.00]	19.6%	1.7%
Lee 2022	8	39146	0.00	[0.00; 0.00]	51.3%	1.7%
**Common effect model**		**76281**	**0.00**	**[0.00; 0.00]**	**100.0%**	**--**
**Random effects model**			**0.06**	**[0.04; 0.09]**	**--**	**100.0%**

Heterogeneity: $I^2 = 99\%$, $\tau^2 = 0.0459$, $p = 0$

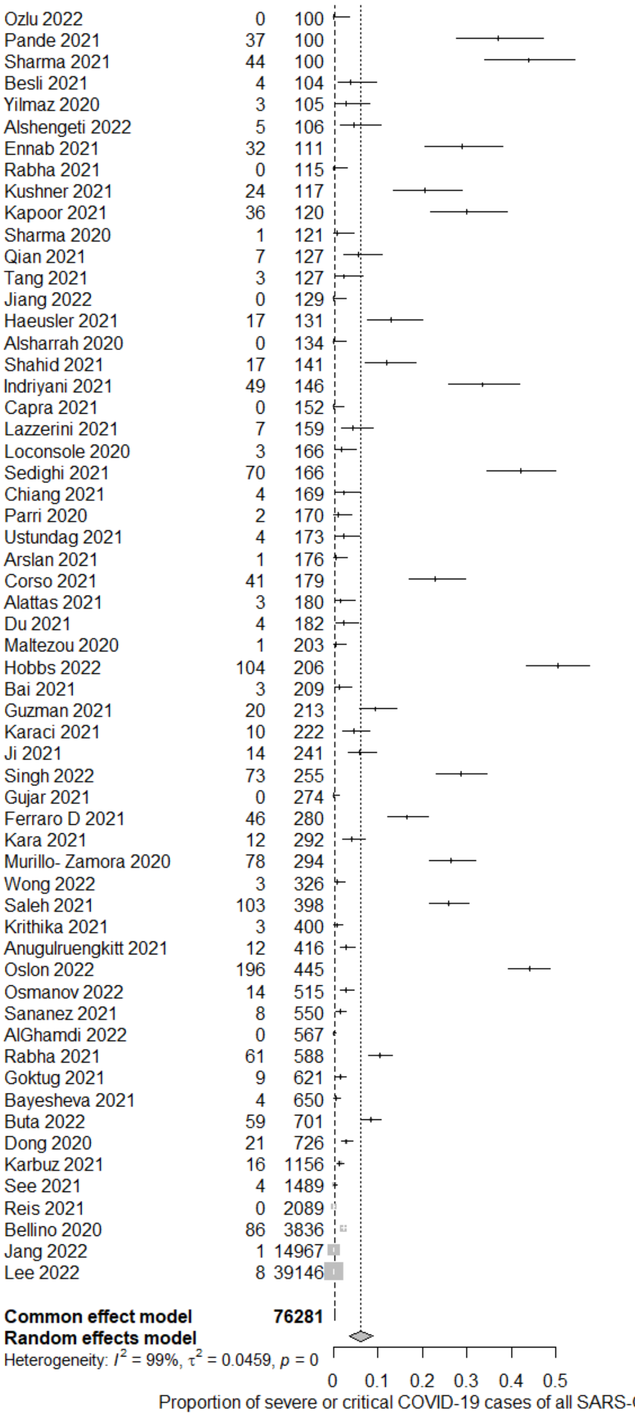

Figure S13: Forest plot: Pooled proportion of SARS-CoV-2 infections in people aged ≤18 years requiring hospital admission

Study	Events	Total	Proportion	95%-CI	Weight (common)	Weight (random)
Besli 2021	8	104	0.08	[0.03; 0.15]	0.0%	1.5%
Nunziata 2020	30	104	0.29	[0.20; 0.39]	0.0%	1.5%
Mania 2021	12	106	0.11	[0.06; 0.19]	0.0%	1.5%
Rabha 2021	22	115	0.19	[0.12; 0.28]	0.0%	1.5%
Sharma 2020	87	121	0.72	[0.63; 0.80]	0.0%	1.5%
Buonsenso 2021	6	129	0.05	[0.02; 0.10]	0.0%	1.5%
Haeusler 2021	49	131	0.37	[0.29; 0.46]	0.0%	1.5%
Omrani 2020	15	131	0.11	[0.07; 0.18]	0.0%	1.5%
Matteudi 2021	19	137	0.14	[0.09; 0.21]	0.0%	1.5%
Kepenekli 2022	39	139	0.28	[0.21; 0.36]	0.0%	1.5%
Shahid 2021	29	141	0.21	[0.14; 0.28]	0.0%	1.5%
Indriyani 2021	55	146	0.38	[0.30; 0.46]	0.0%	1.5%
Capra 2021	22	152	0.14	[0.09; 0.21]	0.0%	1.5%
Lazzerini 2021	45	159	0.28	[0.21; 0.36]	0.0%	1.5%
van der Zalm 2021	62	159	0.39	[0.31; 0.47]	0.0%	1.5%
Loconsole 2020	10	166	0.06	[0.03; 0.11]	0.0%	1.5%
Akkoc 2021	20	167	0.12	[0.07; 0.18]	0.0%	1.5%
Garazzino 2020	110	168	0.65	[0.58; 0.73]	0.0%	1.5%
Chiang 2021	21	169	0.12	[0.08; 0.18]	0.0%	1.5%
Ortiz-Pinto 2022	1	170	0.01	[0.00; 0.03]	0.0%	1.5%
Parri 2020	73	170	0.43	[0.35; 0.51]	0.0%	1.5%
Gavriliu 2021	68	172	0.40	[0.32; 0.47]	0.0%	1.5%
Ustundag 2021	17	173	0.10	[0.06; 0.15]	0.0%	1.5%
Corso 2021	143	179	0.80	[0.73; 0.85]	0.0%	1.5%
Alattas 2021	175	180	0.97	[0.94; 0.99]	0.0%	1.5%
Tosif 2021	18	180	0.10	[0.06; 0.15]	0.0%	1.5%
Maltezou 2020	92	203	0.45	[0.38; 0.52]	0.0%	1.5%
Cheng 2022	2	209	0.01	[0.00; 0.03]	0.0%	1.5%
Guzman 2021	68	214	0.32	[0.26; 0.38]	0.0%	1.5%
Karaci 2021	25	222	0.11	[0.07; 0.16]	0.0%	1.5%
Sharma 2021	100	255	0.39	[0.33; 0.46]	0.0%	1.5%
Singh 2022	100	255	0.39	[0.33; 0.46]	0.0%	1.5%
Alonso 2021	61	266	0.23	[0.18; 0.28]	0.0%	1.5%
Ferraro D 2021	209	280	0.75	[0.69; 0.80]	0.0%	1.5%
Elghoudi 2020	193	288	0.67	[0.61; 0.72]	0.0%	1.5%
Biko 2021	55	313	0.18	[0.14; 0.22]	0.0%	1.5%
Dilber 2021	34	382	0.09	[0.06; 0.12]	0.1%	1.5%
Ibrahim 2021	44	393	0.11	[0.08; 0.15]	0.1%	1.5%
Antúnez-Montes 2021	191	409	0.47	[0.42; 0.52]	0.1%	1.5%
Sena 2021	249	446	0.56	[0.51; 0.60]	0.1%	1.5%
Rabha 2021	85	588	0.14	[0.12; 0.18]	0.1%	1.5%
Goktug 2021	34	621	0.05	[0.04; 0.08]	0.1%	1.5%
Almuzaini 2021	31	654	0.05	[0.03; 0.07]	0.1%	1.5%
Hijazi 2021	29	660	0.04	[0.03; 0.06]	0.1%	1.5%
Yilmaz Celebi 2022	83	680	0.12	[0.10; 0.15]	0.1%	1.5%
Alharbi 2021	71	742	0.10	[0.08; 0.12]	0.1%	1.5%
Hernandez-Garduno 2020	314	961	0.33	[0.30; 0.36]	0.1%	1.5%
Howard 2021	41	1000	0.04	[0.03; 0.06]	0.1%	1.5%
Shapiro Ben David 2021	24	1032	0.02	[0.01; 0.03]	0.1%	1.5%
Soriano-Arandes 2021	27	1040	0.03	[0.02; 0.04]	0.1%	1.5%
Talarico 2021	13	1276	0.01	[0.01; 0.02]	0.2%	1.5%
Mania 2022	1110	1283	0.87	[0.85; 0.88]	0.2%	1.5%
Pokorska-Spiewak 2021	1008	1283	0.79	[0.76; 0.81]	0.2%	1.5%
Ergenc 2021	86	1340	0.06	[0.05; 0.08]	0.2%	1.5%
Reis 2021	9	2089	0.00	[0.00; 0.01]	0.3%	1.5%
Messiah 2021	1145	2116	0.54	[0.52; 0.56]	0.3%	1.5%
Morban 2021	276	2992	0.09	[0.08; 0.10]	0.4%	1.5%
Funk 2022	735	3221	0.23	[0.21; 0.24]	0.4%	1.5%
Bellino 2020	511	3836	0.13	[0.12; 0.14]	0.5%	1.5%
Shi 2022	67	4339	0.02	[0.01; 0.02]	0.6%	1.5%
Bolanos-Almeida 2021	146	5062	0.03	[0.02; 0.03]	0.7%	1.5%
Barrera 2021	132	10437	0.01	[0.01; 0.01]	1.4%	1.5%
Parcha 2021	672	12306	0.05	[0.05; 0.06]	1.7%	1.5%
Jang 2022	4982	14967	0.33	[0.33; 0.34]	2.1%	1.5%
Stokes 2020	848	20458	0.04	[0.04; 0.04]	2.8%	1.5%
Somekh 2021	640	99485	0.01	[0.01; 0.01]	13.6%	1.5%
Leidman 2021	11882	526716	0.02	[0.02; 0.02]	72.2%	1.5%
**Common effect model**		**729187**	**0.03**	**[0.03; 0.03]**	**100.0%**	**--**
**Random effects model**			**0.21**	**[0.15; 0.27]**	**--**	**100.0%**

Heterogeneity: $I^2 = 100\%$, $\tau^2 = 0.0935$, $p = 0$

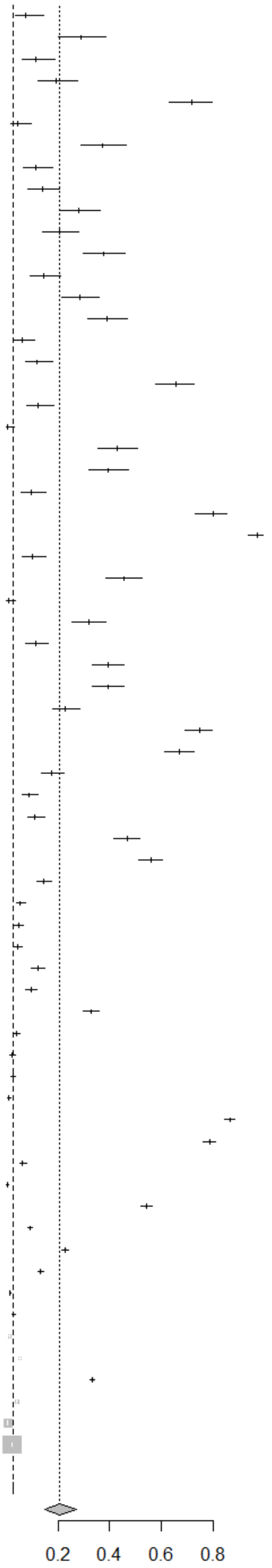

Figure S14: Forest plot: Pooled proportion of SARS-CoV-2 infections in people aged ≤18 years requiring oxygen supplementation.

Study	Events	Total	Proportion	95%-CI	Weight (common)	Weight (random)
Pande 2021	21	100	0.21	[0.13; 0.30]	0.4%	1.7%
Parri 2020	13	100	0.13	[0.07; 0.21]	0.4%	1.7%
Sharma 2021	54	100	0.54	[0.44; 0.64]	0.4%	1.7%
Besli 2021	4	104	0.04	[0.01; 0.10]	0.4%	1.7%
Nunziata 2020	0	104	0.00	[0.00; 0.03]	0.4%	1.7%
Yilmaz 2020	3	105	0.03	[0.01; 0.08]	0.4%	1.7%
Alshengeti 2022	17	106	0.16	[0.10; 0.24]	0.4%	1.7%
Lu 2020	0	110	0.00	[0.00; 0.03]	0.4%	1.7%
Rabha 2021	7	115	0.06	[0.02; 0.12]	0.5%	1.7%
Kushner 2021	26	117	0.22	[0.15; 0.31]	0.5%	1.7%
Sharma 2020	2	121	0.02	[0.00; 0.06]	0.5%	1.7%
Parambil 2021	7	122	0.06	[0.02; 0.11]	0.5%	1.7%
Cloete 2022	27	133	0.20	[0.14; 0.28]	0.5%	1.7%
Kepenekli 2022	1	139	0.01	[0.00; 0.04]	0.6%	1.7%
Shahid 2021	16	141	0.11	[0.07; 0.18]	0.6%	1.7%
Węcławek□Tompol 2021	4	155	0.03	[0.01; 0.06]	0.6%	1.7%
Gaborieau 2020	14	157	0.09	[0.05; 0.15]	0.6%	1.7%
Yilmaz 2021	3	164	0.02	[0.00; 0.05]	0.7%	1.7%
Sedighi 2021	61	166	0.37	[0.29; 0.45]	0.7%	1.7%
Garazzino 2020	18	168	0.11	[0.06; 0.16]	0.7%	1.7%
Chiang 2021	21	169	0.12	[0.08; 0.18]	0.7%	1.7%
Arslan 2021	1	176	0.01	[0.00; 0.03]	0.7%	1.7%
Corso 2021	41	178	0.23	[0.17; 0.30]	0.7%	1.7%
Alattas 2021	3	180	0.02	[0.00; 0.05]	0.7%	1.7%
Du 2021	5	182	0.03	[0.01; 0.06]	0.7%	1.7%
Kumar 2021	46	182	0.25	[0.19; 0.32]	0.7%	1.7%
Lazzerini 2021	4	190	0.02	[0.01; 0.05]	0.8%	1.7%
Kaliyan 2021	11	191	0.06	[0.03; 0.10]	0.8%	1.7%
Hobbs	40	206	0.19	[0.14; 0.25]	0.8%	1.8%
Kim 2020	25	207	0.12	[0.08; 0.17]	0.8%	1.8%
Guzman 2021	11	214	0.05	[0.03; 0.09]	0.9%	1.8%
Xiong 2020	9	244	0.04	[0.02; 0.07]	1.0%	1.8%
Singh 2022	100	255	0.39	[0.33; 0.46]	1.0%	1.8%
Ng 2021	0	261	0.00	[0.00; 0.01]	1.1%	1.8%
Okur 2021	9	262	0.03	[0.02; 0.06]	1.1%	1.8%
Alonso 2021	3	266	0.01	[0.00; 0.03]	1.1%	1.8%
Elghoudi 2020	4	288	0.01	[0.00; 0.04]	1.2%	1.8%
Hendler	160	288	0.56	[0.50; 0.61]	1.2%	1.8%
Biko 2020	14	313	0.04	[0.02; 0.07]	1.3%	1.8%
Wong 2022	4	326	0.01	[0.00; 0.03]	1.3%	1.8%
Chua 2021	0	397	0.00	[0.00; 0.01]	1.6%	1.8%
Saleh 2021	43	398	0.11	[0.08; 0.14]	1.6%	1.8%
Anugulruengkitt 2021	12	416	0.03	[0.01; 0.05]	1.7%	1.8%
Sananez 2021	17	550	0.03	[0.02; 0.05]	2.2%	1.8%
Gotzinger 2020	75	582	0.13	[0.10; 0.16]	2.3%	1.8%
Rabha 2021	25	588	0.04	[0.03; 0.06]	2.4%	1.8%
Uka 2021	34	678	0.05	[0.03; 0.07]	2.7%	1.8%
Buta 2022	21	701	0.03	[0.02; 0.05]	2.8%	1.8%
Wanga 2021	385	713	0.54	[0.50; 0.58]	2.9%	1.8%
Shapiro Ben David 2021	0	1032	0.00	[0.00; 0.00]	4.2%	1.8%
Shoji 2021	15	1038	0.01	[0.01; 0.02]	4.2%	1.8%
Mania 2022	22	1283	0.02	[0.01; 0.03]	5.2%	1.8%
Pokorska-Spiewak 2021	25	1283	0.02	[0.01; 0.03]	5.2%	1.8%
Shoji 2022	31	1299	0.02	[0.02; 0.03]	5.2%	1.8%
See 2021	4	1489	0.00	[0.00; 0.01]	6.0%	1.8%
Kufa 2022	57	2007	0.03	[0.02; 0.04]	8.1%	1.8%
Delahoy 2021	518	3280	0.16	[0.15; 0.17]	13.2%	1.8%
**Common effect model**		**24839**	**0.06**	**[0.05; 0.06]**	**100.0%**	**--**
**Random effects model**			**0.07**	**[0.05; 0.10]**	**--**	**100.0%**

Heterogeneity: $I^2 = 98\%$, $\tau^2 = 0.0401$, $p = 0$

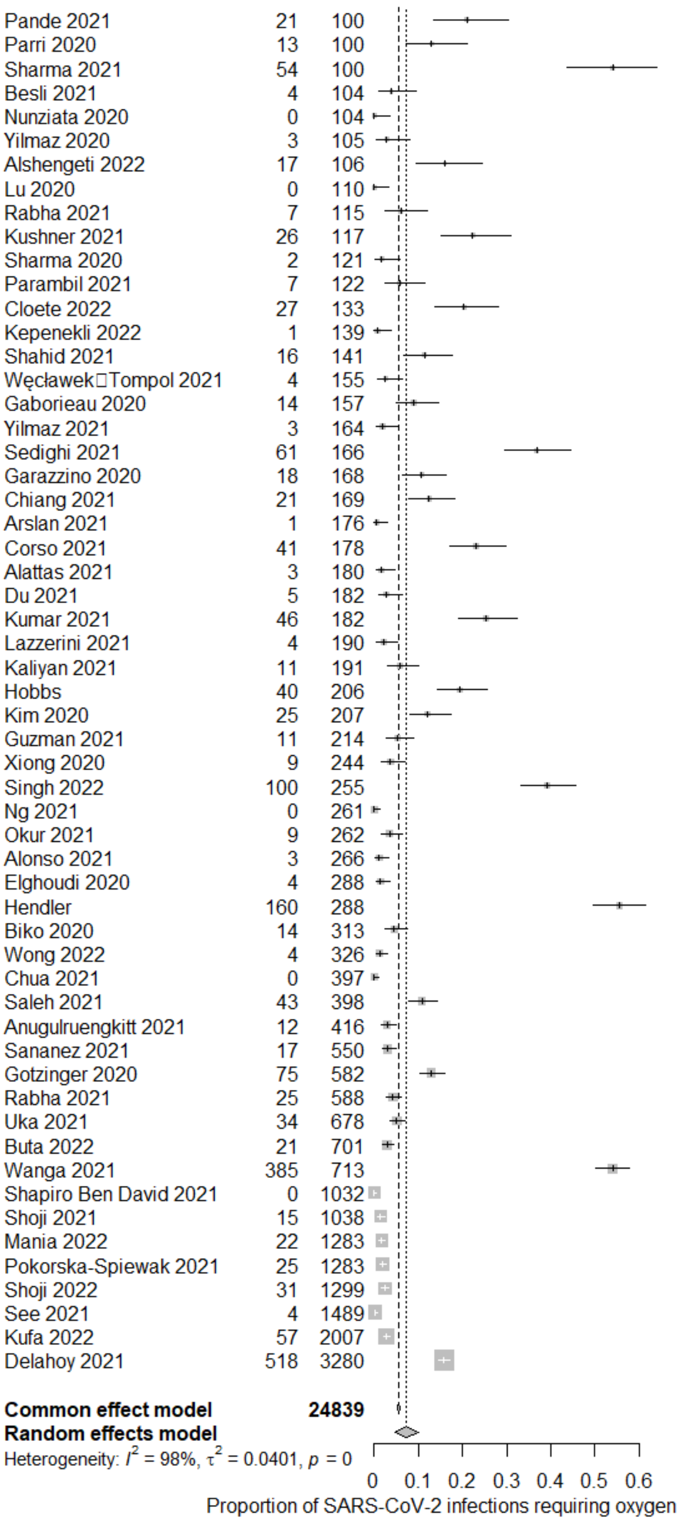

Figure S15: Forest plot: Pooled proportion of SARS-CoV-2 infections in people aged ≤18 years requiring ICU admission

Study	Events	Total	Proportion	95%-CI	Weight (common)	Weight (random)
Dominguez Rojas 2021	13	100	0.13	[0.07; 0.21]	0.0%	1.0%
Pande 2021	37	100	0.37	[0.28; 0.47]	0.0%	1.0%
Hedberg 2022	4	101	0.04	[0.01; 0.10]	0.0%	1.0%
Besli 2021	4	104	0.04	[0.01; 0.10]	0.0%	1.0%
Yilmaz 2020	3	105	0.03	[0.01; 0.08]	0.0%	1.0%
Alshengeti 2022	9	106	0.08	[0.04; 0.16]	0.0%	1.0%
Ennab 2021	0	111	0.00	[0.00; 0.03]	0.0%	1.0%
Rabha 2021	12	115	0.10	[0.06; 0.18]	0.0%	1.0%
Kushner 2021	31	117	0.26	[0.19; 0.35]	0.0%	1.0%
Cofre 2020	33	127	0.26	[0.19; 0.35]	0.1%	1.0%
Tang 2021	1	127	0.01	[0.00; 0.04]	0.1%	1.0%
Buonsenso 2021	3	129	0.02	[0.00; 0.07]	0.1%	1.0%
Gumus 2021	7	130	0.05	[0.02; 0.11]	0.1%	1.0%
Haeusler 2021	15	131	0.11	[0.07; 0.18]	0.1%	1.0%
Alsharrah 2020	0	134	0.00	[0.00; 0.03]	0.1%	1.0%
Cloete 2022	7	138	0.05	[0.02; 0.10]	0.1%	1.0%
Kepenekli 2022	0	139	0.00	[0.00; 0.03]	0.1%	1.0%
Indriyani 2021	2	146	0.01	[0.00; 0.05]	0.1%	1.0%
Gaborieau 2020	16	157	0.10	[0.06; 0.16]	0.1%	1.1%
Lazzerini 2021	2	159	0.01	[0.00; 0.04]	0.1%	1.1%
van der Zalm 2021	11	159	0.07	[0.04; 0.12]	0.1%	1.1%
Yilmaz 2021	3	164	0.02	[0.00; 0.05]	0.1%	1.1%
Loconsole 2020	0	166	0.00	[0.00; 0.02]	0.1%	1.1%
Sedighi 2021	61	166	0.37	[0.29; 0.45]	0.1%	1.1%
Garazzino 2020	2	168	0.01	[0.00; 0.04]	0.1%	1.1%
Ortiz-Pinto 2022	0	170	0.00	[0.00; 0.02]	0.1%	1.1%
Lu 2020	3	171	0.02	[0.00; 0.05]	0.1%	1.1%
Gavriliu	0	172	0.00	[0.00; 0.02]	0.1%	1.1%
Arslan 2021	6	176	0.03	[0.01; 0.07]	0.1%	1.1%
Corso 2021	34	179	0.19	[0.14; 0.26]	0.1%	1.1%
Alattas 2021	3	180	0.02	[0.00; 0.05]	0.1%	1.1%
Tosif 2021	1	180	0.01	[0.00; 0.03]	0.1%	1.1%
Celik 2021	2	197	0.01	[0.00; 0.04]	0.1%	1.1%
Maltezou 2020	1	203	0.00	[0.00; 0.03]	0.1%	1.1%
Hobbs 2022	41	206	0.20	[0.15; 0.26]	0.1%	1.1%
Kim 2020	69	208	0.33	[0.27; 0.40]	0.1%	1.1%
Dominguez-Rodriguez 2021	52	214	0.24	[0.19; 0.31]	0.1%	1.1%
Karaci 2021	6	222	0.03	[0.01; 0.06]	0.1%	1.1%
Wang 2020	3	238	0.01	[0.00; 0.04]	0.1%	1.1%
Song 2021	27	249	0.11	[0.07; 0.15]	0.1%	1.1%
Ng 2021	0	261	0.00	[0.00; 0.01]	0.1%	1.1%
Okur 2021	20	262	0.08	[0.05; 0.12]	0.1%	1.1%
Moreno-Noguez 2021	48	272	0.18	[0.13; 0.23]	0.1%	1.1%
Ferraro D 2021	12	280	0.04	[0.02; 0.07]	0.1%	1.1%
Hendler 2021	89	288	0.31	[0.26; 0.37]	0.1%	1.1%
Kara 2021	9	292	0.03	[0.01; 0.06]	0.1%	1.1%
Biko 2020	19	313	0.06	[0.04; 0.09]	0.1%	1.1%
Wong 2022	4	326	0.01	[0.00; 0.03]	0.1%	1.1%
Devrim 2022	9	392	0.02	[0.01; 0.04]	0.2%	1.1%
Ibrahim 2021	3	393	0.01	[0.00; 0.02]	0.2%	1.1%
Chua 2021	0	397	0.00	[0.00; 0.01]	0.2%	1.1%
Saleh 2021	103	398	0.26	[0.22; 0.30]	0.2%	1.1%
Antúnez-Montes 2021	52	409	0.13	[0.10; 0.16]	0.2%	1.1%
Oslon 2022	180	445	0.40	[0.36; 0.45]	0.2%	1.1%
Sananez 2021	12	550	0.02	[0.01; 0.04]	0.2%	1.1%
AlGhamdi 2022	10	567	0.02	[0.01; 0.03]	0.2%	1.1%
Gotzinger 2020	48	582	0.08	[0.06; 0.11]	0.2%	1.1%
Rabha 2021	28	588	0.05	[0.03; 0.07]	0.2%	1.1%
Goktug 2021	2	621	0.00	[0.00; 0.01]	0.3%	1.1%
Bayesheva 2021	6	650	0.01	[0.00; 0.02]	0.3%	1.1%
Almuzaini 2021	1	654	0.00	[0.00; 0.01]	0.3%	1.1%
Hijazi 2021	1	660	0.00	[0.00; 0.01]	0.3%	1.1%
Uka 2021	11	663	0.02	[0.01; 0.03]	0.3%	1.1%
Yilmaz Celebi 2022	12	680	0.02	[0.01; 0.03]	0.3%	1.1%
Arellano-Llamas 2020	16	682	0.02	[0.01; 0.04]	0.3%	1.1%
Wanga 2021	210	713	0.29	[0.26; 0.33]	0.3%	1.1%
Alharbi 2021	7	742	0.01	[0.00; 0.02]	0.3%	1.1%
Hernandez-Garduno 2020 2020	64	961	0.07	[0.05; 0.08]	0.4%	1.1%
Howard 2021	8	1000	0.01	[0.00; 0.02]	0.4%	1.1%
Shapiro Ben David 2021	0	1032	0.00	[0.00; 0.00]	0.4%	1.1%
Shoji 2021	1	1038	0.00	[0.00; 0.01]	0.4%	1.1%
Karbuz 2021	19	1156	0.02	[0.01; 0.03]	0.5%	1.1%
Talarico 2021	0	1276	0.00	[0.00; 0.00]	0.5%	1.1%
Mania 2022	3	1283	0.00	[0.00; 0.01]	0.5%	1.1%
Pokorska-Spiewak 2021	3	1283	0.00	[0.00; 0.01]	0.5%	1.1%
Shoji 2022	6	1299	0.00	[0.00; 0.01]	0.5%	1.1%
Ergenc 2021	5	1340	0.00	[0.00; 0.01]	0.6%	1.1%
Rivas-Ruiz 2020	48	1443	0.03	[0.02; 0.04]	0.6%	1.1%
See 2021	0	1489	0.00	[0.00; 0.00]	0.6%	1.1%
Kufa 2022	154	2007	0.08	[0.07; 0.09]	0.8%	1.1%
Messiah 2021	27	2116	0.01	[0.01; 0.02]	0.9%	1.1%
Morban 2021	4	2992	0.00	[0.00; 0.00]	1.3%	1.1%
Funk 2022	124	3221	0.04	[0.03; 0.05]	1.4%	1.1%
Delahoy 2021	865	3280	0.26	[0.25; 0.28]	1.4%	1.1%
Bellino 2020	18	3836	0.00	[0.00; 0.01]	1.6%	1.1%
Bolanos-Almeida 2021	26	5062	0.01	[0.00; 0.01]	2.1%	1.1%
Ward 2022	259	5830	0.04	[0.04; 0.05]	2.5%	1.1%
Gomes 2021	1219	6199	0.20	[0.19; 0.21]	2.6%	1.1%
Madani 2021	858	6610	0.13	[0.12; 0.14]	2.8%	1.1%
Parcha 2021	118	12306	0.01	[0.01; 0.01]	5.2%	1.1%
Wong-Chew 2021	449	18465	0.02	[0.02; 0.03]	7.8%	1.1%
Stokes 2020	141	20458	0.01	[0.01; 0.01]	8.7%	1.1%
Leidman 2021	866	110100	0.01	[0.01; 0.01]	46.6%	1.1%
**Common effect model**		**236426**	**0.02**	**[0.02; 0.02]**	**100.0%**	**--**
**Random effects model**			**0.04**	**[0.03; 0.06]**	**--**	**100.0%**

Heterogeneity: $I^2 = 99\%$, $\tau^2 = 0.0301$, $p = 0$

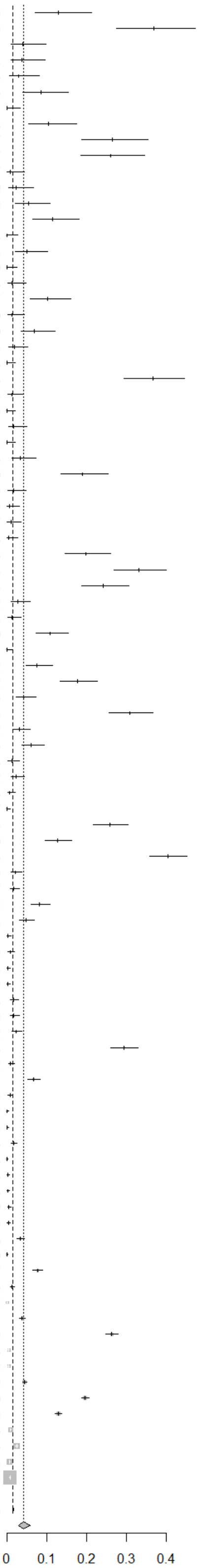

Proportion of SARS-CoV-2 infections in people aged under 18 years requiring ICU admission

Figure S16: Forest plot: Pooled proportion of SARS-CoV-2 infections in people aged ≤18 years requiring assisted ventilation

Study	Events	Total	Proportion	95%-CI	Weight (common)	Weight (random)
Pande 2021	6	100	0.06	[0.02; 0.13]	0.3%	1.5%
Parri 2020	2	100	0.02	[0.00; 0.07]	0.3%	1.5%
Sharma 2021	35	100	0.35	[0.26; 0.45]	0.3%	1.5%
Besli 2021	0	104	0.00	[0.00; 0.03]	0.3%	1.5%
Nunziata 2020	0	104	0.00	[0.00; 0.03]	0.3%	1.5%
Alshengeti 2022	6	106	0.06	[0.02; 0.12]	0.3%	1.5%
Rabha 2021	0	115	0.00	[0.00; 0.03]	0.3%	1.5%
Kapoor 2021	35	120	0.29	[0.21; 0.38]	0.3%	1.5%
Sharma 2020	0	121	0.00	[0.00; 0.03]	0.3%	1.5%
Parambil 2021	3	122	0.02	[0.01; 0.07]	0.3%	1.5%
Haeusler 2021	10	131	0.08	[0.04; 0.14]	0.4%	1.5%
Cloete 2022	7	138	0.05	[0.02; 0.10]	0.4%	1.5%
Kepenekli 2022	1	139	0.01	[0.00; 0.04]	0.4%	1.5%
Shahid 2021	6	141	0.04	[0.02; 0.09]	0.4%	1.5%
Węcławek□Tompol 2021	1	155	0.01	[0.00; 0.04]	0.4%	1.5%
Gaborieau 2020	9	157	0.06	[0.03; 0.11]	0.4%	1.5%
Kumar 2021	25	164	0.15	[0.10; 0.22]	0.5%	1.6%
Yilmaz 2021	2	164	0.01	[0.00; 0.04]	0.5%	1.6%
Garazzino 2020	2	168	0.01	[0.00; 0.04]	0.5%	1.6%
Chiang 2021	3	169	0.02	[0.00; 0.05]	0.5%	1.6%
Lu 2020	3	171	0.02	[0.00; 0.05]	0.5%	1.6%
Arslan 2021	1	176	0.01	[0.00; 0.03]	0.5%	1.6%
Corso 2021	17	178	0.10	[0.06; 0.15]	0.5%	1.6%
Alattas 2021	2	180	0.01	[0.00; 0.04]	0.5%	1.6%
Du 2021	3	182	0.02	[0.00; 0.05]	0.5%	1.6%
Lazzerini 2021	1	190	0.01	[0.00; 0.03]	0.5%	1.6%
Kaliyan 2021	5	191	0.03	[0.01; 0.06]	0.5%	1.6%
Hobbs	10	206	0.05	[0.02; 0.09]	0.6%	1.6%
Kim 2020	20	208	0.10	[0.06; 0.14]	0.6%	1.6%
Bai 2021	1	209	0.00	[0.00; 0.03]	0.6%	1.6%
Guzman 2021	7	214	0.03	[0.01; 0.07]	0.6%	1.6%
Xiong 2020	3	244	0.01	[0.00; 0.04]	0.7%	1.6%
Singh 2022	35	255	0.14	[0.10; 0.19]	0.7%	1.6%
Okur 2021	9	262	0.03	[0.02; 0.06]	0.7%	1.6%
Alonso 2021	1	266	0.00	[0.00; 0.02]	0.7%	1.6%
Elghoudi 2020	0	288	0.00	[0.00; 0.01]	0.8%	1.6%
Hendler 2021	40	288	0.14	[0.10; 0.18]	0.8%	1.6%
Kara 2021	4	292	0.01	[0.00; 0.03]	0.8%	1.6%
Biko 2020	14	313	0.04	[0.02; 0.07]	0.9%	1.6%
Wong 2022	2	326	0.01	[0.00; 0.02]	0.9%	1.6%
Saleh 2021	43	398	0.11	[0.08; 0.14]	1.1%	1.6%
Anugulruengkitt 2021	0	416	0.00	[0.00; 0.01]	1.2%	1.6%
Olson 2022	140	443	0.32	[0.27; 0.36]	1.2%	1.6%
Sananez 2021	4	550	0.01	[0.00; 0.02]	1.5%	1.6%
AlGhamdi 2022	10	567	0.02	[0.01; 0.03]	1.6%	1.6%
Gotzinger 2020	42	582	0.07	[0.05; 0.10]	1.6%	1.6%
Goktug 2021	2	621	0.00	[0.00; 0.01]	1.7%	1.6%
Bayesheva 2021	3	650	0.00	[0.00; 0.01]	1.8%	1.6%
Hijazi 2021	0	660	0.00	[0.00; 0.01]	1.9%	1.6%
Uka 2021	8	678	0.01	[0.01; 0.02]	1.9%	1.6%
Wanga 2021	125	713	0.18	[0.15; 0.21]	2.0%	1.6%
Hernandez-Garduno 2020	37	961	0.04	[0.03; 0.05]	2.7%	1.7%
Howard 2021	2	1000	0.00	[0.00; 0.01]	2.8%	1.7%
Shapiro Ben David 2021	0	1032	0.00	[0.00; 0.00]	2.9%	1.7%
Shoji 2021	0	1038	0.00	[0.00; 0.00]	2.9%	1.7%
Karbuz 2021	5	1156	0.00	[0.00; 0.01]	3.3%	1.7%
Mania 2022	0	1283	0.00	[0.00; 0.00]	3.6%	1.7%
Pokorska-Spiewak 2021	0	1283	0.00	[0.00; 0.00]	3.6%	1.7%
Shoji 2022	0	1299	0.00	[0.00; 0.00]	3.7%	1.7%
Rivas-Ruiz 2020	26	1443	0.02	[0.01; 0.03]	4.1%	1.7%
Kufa 2022	57	2007	0.03	[0.02; 0.04]	5.6%	1.7%
Delahoy 2021	343	3280	0.10	[0.09; 0.12]	9.2%	1.7%
Gomes 2021	822	6199	0.13	[0.12; 0.14]	17.4%	1.7%
**Common effect model**		**35516**	**0.03**	**[0.03; 0.04]**	**100.0%**	**--**
**Random effects model**			**0.03**	**[0.02; 0.04]**	**--**	**100.0%**

Heterogeneity: $I^2 = 98\%$, $\tau^2 = 0.0202$, $p = 0$

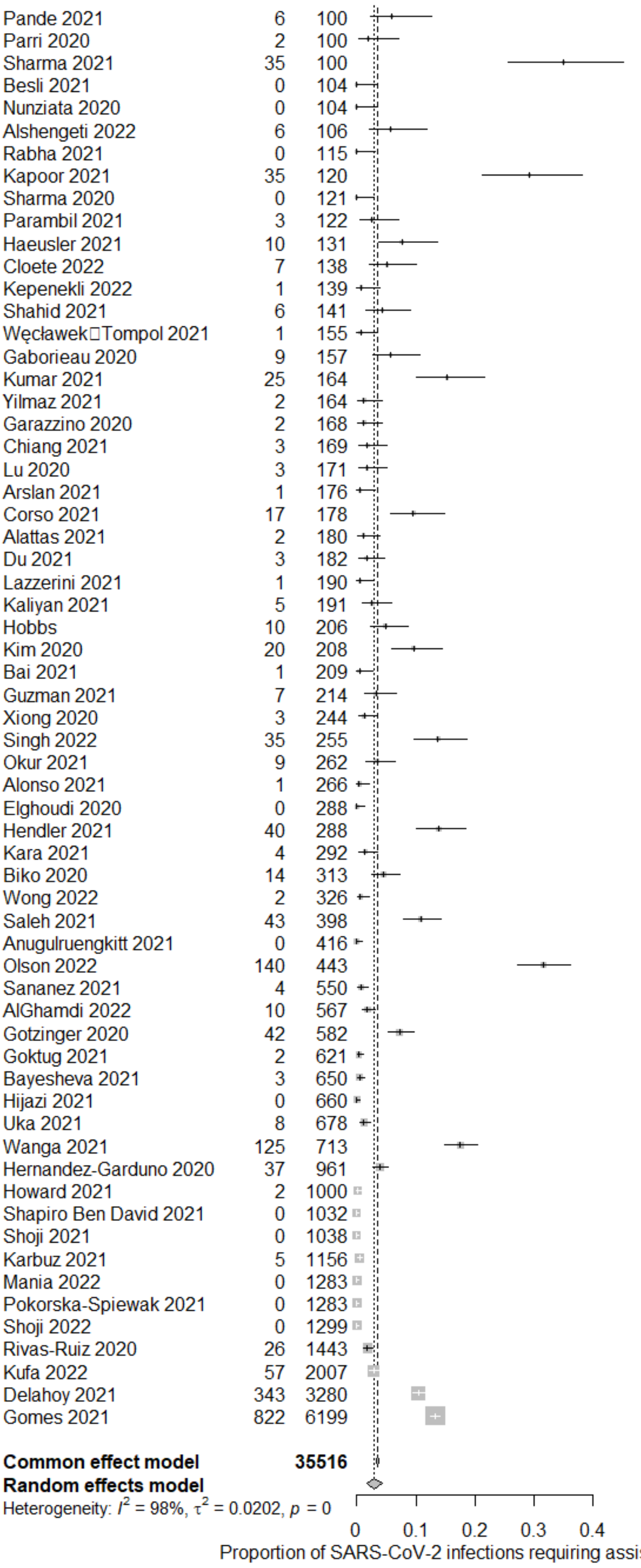

Figure S17: Forest plot: Pooled case fatality rate of SARS-CoV-2 infections in people aged ≤18 years

Study	Events	Total	Proportion	95%-CI	Weight (common)	Weight (random)
Dominguez Rojas 2021	4	100	0.04	[0.01; 0.10]	0.0%	0.7%
Ozlu 2022	0	100	0.00	[0.00; 0.04]	0.0%	0.7%
Pande 2021	3	100	0.03	[0.01; 0.09]	0.0%	0.7%
Hedberg 2022	1	101	0.01	[0.00; 0.05]	0.0%	0.7%
Besli 2021	0	104	0.00	[0.00; 0.03]	0.0%	0.7%
Chopra 2021	27	105	0.26	[0.18; 0.35]	0.0%	0.7%
Yilmaz 2020	0	105	0.00	[0.00; 0.03]	0.0%	0.7%
Alshengeti 2022	2	106	0.02	[0.00; 0.07]	0.0%	0.7%
Mania 2021	0	106	0.00	[0.00; 0.03]	0.0%	0.7%
Ennab 2021	0	111	0.00	[0.00; 0.03]	0.0%	0.7%
Du 2020	0	114	0.00	[0.00; 0.03]	0.0%	0.8%
Rabha 2021	0	115	0.00	[0.00; 0.03]	0.0%	0.8%
Kushner 2021	1	117	0.01	[0.00; 0.05]	0.0%	0.8%
Maltezou 2020	0	118	0.00	[0.00; 0.03]	0.0%	0.8%
Kapoor 2021	29	120	0.24	[0.17; 0.33]	0.0%	0.8%
Sharma 2020	0	121	0.00	[0.00; 0.03]	0.0%	0.8%
Parambil 2021	6	122	0.05	[0.02; 0.10]	0.0%	0.8%
Cofre 2020	1	127	0.01	[0.00; 0.04]	0.0%	0.8%
Qian 2021	2	127	0.02	[0.00; 0.06]	0.0%	0.8%
Tang 2021	1	127	0.01	[0.00; 0.04]	0.0%	0.8%
Jiang 2022	0	129	0.00	[0.00; 0.03]	0.0%	0.8%
Gumus 2021	6	130	0.05	[0.02; 0.10]	0.0%	0.8%
Haeusler 2021	4	131	0.03	[0.01; 0.08]	0.0%	0.8%
Omrani 2020	0	131	0.00	[0.00; 0.03]	0.0%	0.8%
Matteudi 2021	1	137	0.01	[0.00; 0.04]	0.0%	0.8%
Cloete 2022	4	138	0.03	[0.01; 0.07]	0.0%	0.8%
Kepenekli 2022	0	139	0.00	[0.00; 0.03]	0.0%	0.8%
Shahid 2021	10	141	0.07	[0.03; 0.13]	0.0%	0.8%
Indriyani 2021	4	146	0.03	[0.01; 0.07]	0.0%	0.8%
Capra 2021	0	152	0.00	[0.00; 0.02]	0.0%	0.8%
Węcławek□Tompol 2021	4	155	0.03	[0.01; 0.06]	0.0%	0.8%
Gaborieau 2020	3	157	0.02	[0.00; 0.05]	0.0%	0.8%
Lazzerini 2021	0	159	0.00	[0.00; 0.02]	0.0%	0.8%
van der Zalm 2021	2	159	0.01	[0.00; 0.04]	0.0%	0.8%
Yilmaz 2021	2	164	0.01	[0.00; 0.04]	0.0%	0.8%
Loconsole 2020	0	166	0.00	[0.00; 0.02]	0.0%	0.8%
Sedighi 2021	30	166	0.18	[0.13; 0.25]	0.0%	0.8%
Chiang 2021	1	169	0.01	[0.00; 0.03]	0.0%	0.8%
Parri 2020	0	170	0.00	[0.00; 0.02]	0.0%	0.8%
Lu 2020	1	171	0.01	[0.00; 0.03]	0.0%	0.8%
Arslan 2021	1	176	0.01	[0.00; 0.03]	0.0%	0.8%
Corso 2021	22	179	0.12	[0.08; 0.18]	0.0%	0.8%
Alattas 2021	0	180	0.00	[0.00; 0.02]	0.0%	0.8%
Tosif 2021	0	180	0.00	[0.00; 0.02]	0.0%	0.8%
Du 2021	1	182	0.01	[0.00; 0.03]	0.0%	0.8%
Kumar 2021	3	182	0.02	[0.00; 0.05]	0.0%	0.8%
Kaliyan 2021	1	191	0.01	[0.00; 0.03]	0.0%	0.8%
Celik 2021	1	197	0.01	[0.00; 0.03]	0.0%	0.8%
Hobbs 2022	1	206	0.00	[0.00; 0.03]	0.0%	0.8%
Kim 2020	1	208	0.00	[0.00; 0.03]	0.0%	0.8%
Bai 2021	0	209	0.00	[0.00; 0.02]	0.0%	0.8%
Guzman 2021	1	214	0.00	[0.00; 0.03]	0.0%	0.8%
Karaci 2021	0	222	0.00	[0.00; 0.02]	0.0%	0.8%
Xiong 2020	1	244	0.00	[0.00; 0.02]	0.0%	0.8%
Song 2021	2	249	0.01	[0.00; 0.03]	0.0%	0.8%
Sharma 2021	27	255	0.11	[0.07; 0.15]	0.0%	0.8%
Singh 2022	29	255	0.11	[0.08; 0.16]	0.0%	0.8%
Ng 2021	0	261	0.00	[0.00; 0.01]	0.0%	0.8%
Okur 2021	0	262	0.00	[0.00; 0.01]	0.0%	0.8%
Alonso 2021	0	266	0.00	[0.00; 0.01]	0.0%	0.8%
Gujar 2021	0	274	0.00	[0.00; 0.01]	0.0%	0.8%
Ferraro D 2021	2	280	0.01	[0.00; 0.03]	0.0%	0.8%
Elghoudi 2020	0	288	0.00	[0.00; 0.01]	0.0%	0.8%
Hendler 2021	17	288	0.06	[0.03; 0.09]	0.0%	0.8%
Kara 2021	1	292	0.00	[0.00; 0.02]	0.0%	0.8%
Wong 2022	0	326	0.00	[0.00; 0.01]	0.0%	0.9%
Dilber 2021	0	382	0.00	[0.00; 0.01]	0.0%	0.9%
Saleh 2021	27	398	0.07	[0.05; 0.10]	0.0%	0.9%
Krithika 2021	0	400	0.00	[0.00; 0.01]	0.0%	0.9%
Oslon 2022	7	407	0.02	[0.01; 0.04]	0.0%	0.9%
Antúnez-Montes 2021	17	409	0.04	[0.02; 0.07]	0.0%	0.9%
Chua 2020	0	423	0.00	[0.00; 0.01]	0.0%	0.9%
Sena 2021	28	446	0.06	[0.04; 0.09]	0.1%	0.9%
Sananez 2021	2	550	0.00	[0.00; 0.01]	0.1%	0.9%
AlGhamdi 2022	4	567	0.01	[0.00; 0.02]	0.1%	0.9%
Gotzinger 2020	4	582	0.01	[0.00; 0.02]	0.1%	0.9%
Rabha 2021	0	588	0.00	[0.00; 0.01]	0.1%	0.9%
Goktug 2021	2	621	0.00	[0.00; 0.01]	0.1%	0.9%
Bayasheva 2021	0	650	0.00	[0.00; 0.01]	0.1%	0.9%
Hijazi 2021	2	660	0.00	[0.00; 0.01]	0.1%	0.9%
Uka 2021	3	678	0.00	[0.00; 0.01]	0.1%	0.9%
Arellano-Llamas 2020	12	682	0.02	[0.01; 0.03]	0.1%	0.9%
Buta 2022	0	701	0.00	[0.00; 0.01]	0.1%	0.9%
Wanga 2021	11	713	0.02	[0.01; 0.03]	0.1%	0.9%
Alharbi 2021	2	742	0.00	[0.00; 0.01]	0.1%	0.9%
Hernandez-Garduno 2020	40	961	0.04	[0.03; 0.06]	0.1%	0.9%
Howard 2021	1	1000	0.00	[0.00; 0.01]	0.1%	0.9%
Shapiro Ben David 2021	0	1032	0.00	[0.00; 0.00]	0.1%	0.9%
Shoji 2021	0	1038	0.00	[0.00; 0.00]	0.1%	0.9%
Soriano-Arandes 2021	0	1040	0.00	[0.00; 0.00]	0.1%	0.9%
Karbuz 2021	2	1156	0.00	[0.00; 0.01]	0.1%	0.9%
Mania 2022	0	1283	0.00	[0.00; 0.00]	0.1%	0.9%
Pokorska-Spiewak 2021	0	1283	0.00	[0.00; 0.00]	0.1%	0.9%
Shoji 2022	0	1299	0.00	[0.00; 0.00]	0.1%	0.9%
Ergenc 2021	0	1340	0.00	[0.00; 0.00]	0.2%	0.9%
Rivas-Ruiz 2020	27	1443	0.02	[0.01; 0.03]	0.2%	0.9%
See 2021	0	1489	0.00	[0.00; 0.00]	0.2%	0.9%
Kufa 2022	47	2007	0.02	[0.02; 0.03]	0.2%	0.9%
Reis 2021	0	2089	0.00	[0.00; 0.00]	0.2%	0.9%
Morban 2021	6	2992	0.00	[0.00; 0.00]	0.3%	0.9%
Funk 2022	4	3221	0.00	[0.00; 0.00]	0.4%	0.9%
Delahoy 2021	24	3280	0.01	[0.00; 0.01]	0.4%	0.9%
Bellino 2020	4	3836	0.00	[0.00; 0.00]	0.4%	0.9%
Rodriguez Velasquez 2021	27	4676	0.01	[0.00; 0.01]	0.5%	0.9%
Sousa 2021	424	4676	0.09	[0.08; 0.10]	0.5%	0.9%
Bolanos-Almeida 2021	8	5062	0.00	[0.00; 0.00]	0.6%	0.9%
Ward 2022	8	5830	0.00	[0.00; 0.00]	0.7%	0.9%
Dash 2021	36	5988	0.01	[0.00; 0.01]	0.7%	0.9%
Gomes 2021	500	6199	0.08	[0.07; 0.09]	0.7%	0.9%
Madani 2021	352	6610	0.05	[0.05; 0.06]	0.7%	0.9%
Oliveira 2021	548	8024	0.07	[0.06; 0.07]	0.9%	0.9%
Barrera 2021	1	10437	0.00	[0.00; 0.00]	1.2%	0.9%
Musa 2021	0	11445	0.00	[0.00; 0.00]	1.3%	0.9%
Jang 2022	0	14967	0.00	[0.00; 0.00]	1.7%	0.9%
Wong-Chew 2021	244	18465	0.01	[0.01; 0.01]	2.1%	0.9%
Stokes 2020	13	20458	0.00	[0.00; 0.00]	2.3%	0.9%
Pudjiadi 2021	175	37706	0.00	[0.00; 0.01]	4.3%	0.9%
Navarro-Olivos 2021	425	48505	0.01	[0.01; 0.01]	5.5%	0.9%
Leidman 2021	178	621167	0.00	[0.00; 0.00]	70.3%	0.9%
**Common effect model**		**883925**	**0.00**	**[0.00; 0.00]**	**100.0%**	**--**
**Random effects model**			**0.01**	**[0.01; 0.01]**	**--**	**100.0%**

Heterogeneity: $I^2 = 99\%$, $\tau^2 = 0.0094$, $p = 0$

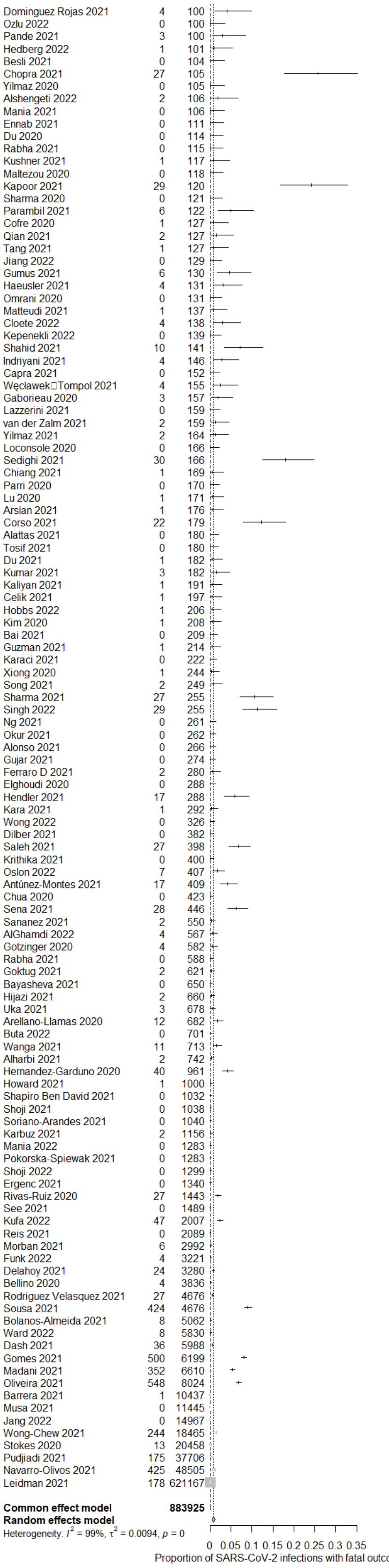

Supplement: Online Supplementary Document [file jogh-14-05003-s001.pdf]
